# CD40 agonist mitazalimab with mFOLFIRINOX in untreated metastatic pancreatic cancer: Biomarkers associated with outcomes from OPTIMIZE-1

**DOI:** 10.1016/j.xcrm.2025.102407

**Published:** 2025-10-07

**Authors:** Jean-Luc Van Laethem, Karen Geboes, Ivan Borbath, Teresa Macarulla Mercade, Aurélien Lambert, Philippe Cassier, Hans Prenen, Emmanuel Mitry, Jean-Frédéric Blanc, Lorenzo Pilla, Jaime Feliu, Mercedes Rodriguez Garrote, Roberto Antonio Pazo-Cid, Inmaculada Gallego, Karin Enell Smith, Karin Nordbladh, David Gomez Jimenez, Peter Ellmark, Yago Pico de Coaña, Sumeet Vijay Ambarkhane, Gregory L. Beatty, Eileen M. O'Reilly

**Affiliations:** 1 Gastroenterology, Hepatology and Digestive Oncology, Hopital Universitaire Bruxelles. Erasme Universite Libre de Bruxelles, Brussels, Belgium; 2 Gastroenterology Department, UZ Gent - University Hospital Ghent, Gent, Belgium; 3 Hepatogastroenterology, Cliniques Universitaires Saint-Luc (UCLouvain Saint-Luc), Woluwe-Saint-Lambert, Belgium; 4 Medical Oncology Department, Vall d'Hebron Institute of Oncology, Barcelona, Spain; 5 Institut de Cancérologie de Lorraine, Université de Lorraine, Nancy, France; 6 Medical Oncology Department, Centre Léon Bérard, Lyon, France; 7 Oncology Department, UZA - University Hospital Antwerp, Edegem, Belgium; 8 Medical Oncology Department, IPC - Institut Paoli-Calmettes, Marseille, Cedex, France; 9 Hepatology, Gastroenterology and Digestive Oncology, CHU Bordeaux - Hopital Haut-Lévêque, Bordeaux, France; 10 Department of Hepato-Gastroenterology and Gastrointestinal Oncology, Georges Pompidou European Hospital, Paris, France; 11 Department of Medical Oncology, Hospital Universitario La Paz, Madrid, Spain; 12 Medical Oncology, Hospital Universitario Ramon y Cajal, Madrid, Spain; 13 Medical Oncology Department, HospItal Universitario Miguel Servet, Zaragoza, Spain; 14 Medical Oncology, Hospital Universitario Virgen del Rocio, Seville, Spain; 15 Non-clinical, Alligator Bioscience AB, Lund, Sweden; 16 Clinical Operations, Alligator Bioscience AB, Lund, Sweden; 17 Scientific, Alligator Bioscience AB, Lund, Sweden; 18 Department of Immunotechnology, Lund University, Lund, Sweden; 19 Medical, Alligator Bioscience AB, Lund, Sweden; 20 Division of Hematology/Oncology, Perelman School of Medicine, University of Pennsylvania, Philadelphia, PA, USA; 21 Memorial Sloan Kettering Cancer Center, New York, NY, USA; 22 Instituto de Investigación Sanitaria del Hospital Universitario La Paz (IdiPAZ), Madrid, Spain; 23 Centro de Investigación Biomédica en Red de Cáncer (CIBERONC), Madrid, Spain

**Keywords:** CD40 agonist, mitazalimab, mPDAC, biomarker, immunotherapy, chemotherapy, mFOLFIRINOX, ct*KRAS*, classical mPDAC, tumor microenvironment

## Abstract

Response determinants to immunotherapy in metastatic pancreatic ductal adenocarcinoma (mPDAC) remain unclear, limiting treatment advancements. We report a single-arm phase 1b/2 study (OPTIMIZE-1) evaluating the safety and efficacy of the cluster of differentiation 40 (CD40) agonist mitazalimab combined with modified FOLFIRINOX (mFOLFIRINOX), in chemotherapy-naive patients with mPDAC. Patients receive an initial dose of mitazalimab one week before starting biweekly cycles of mFOLFIRINOX plus mitazalimab. The study meets its pre-specified primary endpoint, achieving a confirmed objective response rate (ORR) of 42.1%. Median duration of response, progression-free survival, and overall survival was 12.6 months, 7.7 months, and 14.9 months, respectively. Multi-omic analyses of tumor and blood specimens identify a baseline tumor-intrinsic gene signature related to fibrosis associated with improved survival. Additionally, mitazalimab-induced increases in activated circulating myeloid, B cell, and T cell frequencies correlate with better outcomes. These results may inform future patient stratification strategies supporting a planned randomized confirmatory trial of mitazalimab with mFOLFIRINOX in mPDAC. This study was registered at ClinicalTrials.gov (NCT04888312).

## Highlights

• Encouraging activity: ORR 42.1%, DoR 12.6 months, OS 14.9 months, and 18-month OS 36.2%
• A baseline fibrosis gene-signature associates with OS
• Mitazalimab induces intratumoral myeloid and T cell activation in objective responders
• Mitazalimab-induced immune activation correlates with better outcomes

## Graphical abstract

**Figure:**
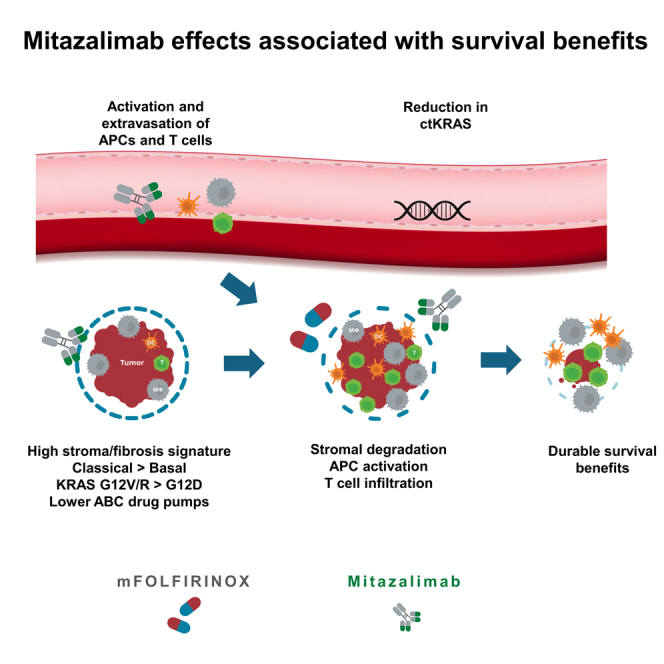

## Teaser

Van Laethem et al. report that the combination of mFOLFIRINOX and mitazalimab has manageable safety and encouraging activity. Mitazalimab-induced immune activation and a fibrosis gene signature correlate with better outcomes. These results may inform future patient stratification strategies supporting a planned randomized confirmatory trial in mPDAC.

## Introduction

Pancreatic ductal adenocarcinoma (PDAC) is a highly aggressive malignancy with a dismal prognosis and is projected to become the second leading cause of cancer-related deaths by 2030.^1^ Metastatic PDAC (mPDAC) is particularly lethal, with a 5-year survival rate of only 3%. Current standard of care regimens, including gemcitabine plus nab-paclitaxel and FOLFIRINOX, achieve objective response rates (ORRs) of 20%–40% and a median progression-free survival (PFS) of approximately 6 months^2^ The recently approved NALIRIFOX regimen provides similar outcomes.^3^

Treatment resistance in mPDAC is largely attributed to its unique tumor microenvironment (TME), characterized by a desmoplastic reaction and a dense fibrosis. This microenvironment is frequently associated with the infiltration of suppressive myeloid cells, exclusion of cytotoxic T cells, poor vascularity, and high interstitial pressures, all of which limit drug delivery and contribute to the immunosuppressive nature of the disease.^4^ Consequently, immunotherapies such as immune checkpoint inhibitors have demonstrated limited efficacy in mPDAC.^5^^,^^6^

Despite these challenges, the TME of mPDAC is plastic and has the potential to be reprogrammed for anti-tumor effects. Notably, patients with mPDAC exhibiting increased T cell infiltration into tumors have a more favorable prognosis,^7^^,^^8^^,^^9^ suggesting the potential of T cell-directed immunotherapies. One promising approach involves targeting cluster of differentiation 40 (CD40), a member of the tumor necrosis factor receptor superfamily.^10^ CD40 is expressed on the surface of B cells, monocytes, macrophages and dendritic cells (DCs) and plays a key role in bridging innate and adaptive immunity by “licensing” DCs to prime and activate T cells, thereby triggering tumor-specific T cell immunity. Preclinical models have demonstrated the potential of CD40 agonists to reverse T cell exclusion in PDAC,^11^ and clinical data have revealed similar biology.^12^ Recent studies investigating the combination of CD40 agonists with gemcitabine and nab-paclitaxel in PDAC have shown that tumor extrinsic immune signatures associate with improved outcomes,^13^^,^^14^ consistent with the potential benefit of CD40 agonism.

Preclinical models show that CD40 agonists can remodel the TME to enhance the efficacy of chemotherapy,^15^ and clinical data support this remodeling of the fibrotic reaction by CD40 agonists in PDAC.^12^ Thus, the sequencing of CD40 agonist administration and chemotherapy may be critical to treatment efficacy.^10^ We recently reported results from the OPTIMIZE-1 study, which investigated the combination of modified FOLFIRINOX (mFOLFIRINOX) with mitazalimab, a second-generation CD40 agonist, as first-line treatment for patients with mPDAC.^16^ In this study, mitazalimab was administered first as a conditioning regimen to remodel the tumor and sensitize it to chemotherapy. This treatment sequencing approach resulted in deep responses, an increase in duration of response (DoR), and prolonged overall survival (OS). Importantly, the safety profile was consistent with mFOLFIRINOX, with no signs of toxicity related to the addition of mitazalimab.

A significant challenge in developing effective immunotherapies for mPDAC is the identification of patients most likely to benefit from treatment. Currently, there are no validated prognostic biomarkers to predict benefit from mFOLFIRINOX, although *KRAS* G12 subtype and tumor molecular classification into classical and basal-like subtypes have been used to genotype tumors.^17^^,^^18^^,^^19^^,^^20^^,^^21^^,^^22^^,^^23^^,^^24^ Circulating tumor DNA (ctDNA) has also emerged as a promising longitudinal prognostic marker for patients with solid tumors.^25^^,^^26^^,^^27^^,^^28^ In this study, we report updated clinical results and investigate determinants of treatment efficacy of mitazalimab combined with mFOLFIRINOX. Our findings reveal that a baseline tumor-intrinsic gene signature comprising transcripts related to fibrosis associates with improved survival. Further, increases in frequencies of activated myeloid, B, and T cells following mitazalimab administration correlate with improved outcomes, supporting mitazalimab’s contribution to anti-tumor activity. Additional factors associated with better OS included ctDNA clearance and classical PDAC subtype. Collectively, our work defines potential patient subsets that derive the greatest benefit from this combination therapy and supports the use of mitazalimab as a therapeutic agent to enhance the efficacy of mFOLFIRINOX chemotherapy.

## Results

### Clinical study data analysis: Study design, patient characteristics, and patient treatment

This phase 1b/2 study was designed as a single-arm study to assess the safety and preliminary efficacy of mitazalimab in combination with mFOLFIRINOX in patients with treatment-naive mPDAC. Patients received mitazalimab on day 1 (priming dose), followed by a 2-week dosing regimen starting with mFOLFIRINOX on day 8 and mitazalimab on day 10. Treatment was administered until disease progression (PD), unacceptable toxicities, or consent withdrawal, whichever came first. The phase 2 primary objective was to assess the clinical activity of this combination therapy using ORR as the primary endpoint. Between September 29, 2021, and March 28, 2023, 88 chemotherapy-naive patients with mPDAC were screened for study eligibility. Of these, 70 patients fulfilled the eligibility criteria and received study treatment (see Figures S1A and S1B). Baseline demographics and clinical characteristics of these 70 treated patients are presented in Table S1. In the phase 1b dose-escalation portion, five patients received 450 μg/kg mitazalimab and six patients received 900 μg/kg. In the phase 2 portion, all patients received mitazalimab at the recommended phase 2 dose (RP2D) of 900 μg/kg mitazalimab (Figure S1A).

For the efficacy analysis, only patients who received mitazalimab at the RP2D (900 μg/kg) and had completed at least two treatment cycles at the RP2D were included in the full analysis set (FAS), regardless of whether they were enrolled in phase 1b or phase 2. Thus, the FAS comprised 57 patients: six from phase 1b (received mitazalimab at 900 μg/kg) and 51 from phase 2. The five patients from phase 1b who received the lower dose (450 μg/kg) were excluded from the efficacy analysis, as were any patients who discontinued before completing two cycles at the RP2D to ensure that efficacy outcomes reflect the activity of the dose selected for further development. In total, 65 patients were treated with at least one dose of mitazalimab, Figure S1C lists the 8 patients who discontinued before Cycle 2 along with the reasons for exclusion.

### Clinical study data analysis: Primary and secondary efficacy endpoints

**Figure 1:**
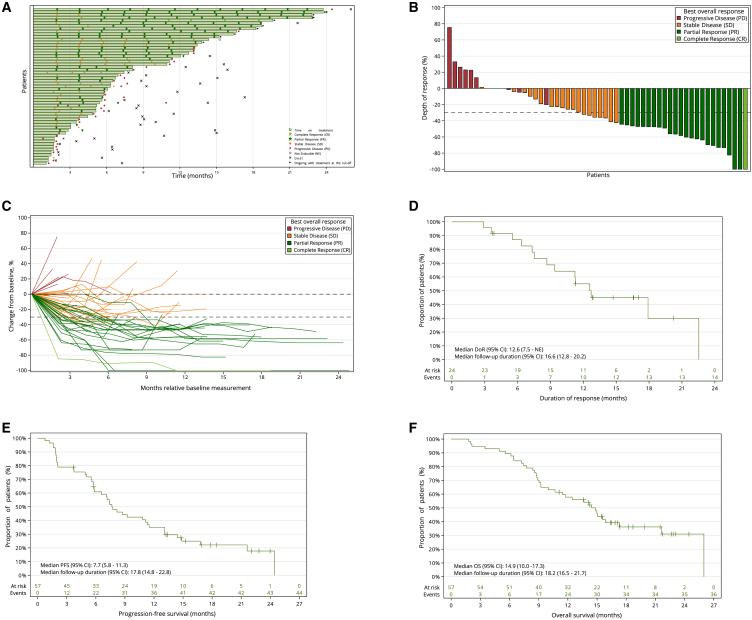
Clinical outcomes in the OPTIMIZE-1 trial (A) Swimmer plot showing the treatment durations for all 57 patients in the full analysis set. (B) Waterfall plot showing the maximum percentage change in the tumor size relative to baseline target lesions of individual patients by best overall response. (C) Spider plot showing percent change from baseline in summed tumor target lesions over time. (D) KaplanMeier (KM) curve estimates for duration of response (DoR). (E) KM curve estimates for progression-free survival (PFS). (F) KM curve estimates for overall survival (OS). CI, confidence interval.

We present an updated efficacy analysis based on data from 57 efficacy-evaluable patients (FAS) treated with 900 μg/kg mitazalimab (data cut-off May 16, 2024, median follow-up duration of 18.2 months [95% confidence interval (CI): 16.5–21.7]). At the time of data cut-off, 19 patients (33.3%) remained in the study, with nine (15.8%) still receiving treatment (Figure 1A). Forty-eight patients discontinued treatment, 39 due to PD. Of these, 32 patients (56.1%, of the 57 evaluable patients) received second-line systemic therapies (Table S2).

In the FAS, the confirmed ORR (primary endpoint) was 42.1%, comprising 24 partial responses (PRs) and one complete response (CR) (Figures 1A–1C). Including unconfirmed responses, the ORR reached 54.4%. The disease control rate (DCR), was 78.9%. Key efficacy outcomes included a median DoR of 12.6 months, a median PFS of 7.7 months (with 6- and 12-month PFS of 60.9% and 35.1%, respectively), and a median OS of 14.9 months (with 12- and 18-month OS of 57.8% and 36.2%, respectively) (Figures 1D–1F). Eighteen patients (31.6%) received treatment beyond 12 months, and seven patients (12.3%) achieved a late objective response (at approximately the 6-month time point during treatment).

Efficacy outcomes were also analyzed in the intent-to-treat (ITT) population, which included all 65 patients who received at least one dose of 900 μg/kg mitazalimab. In the ITT population, the confirmed ORR was 36.9% (47.7% unconfirmed). The DCR was 69.2%, and the median OS was 14 months. Key efficacy outcomes for both the FAS and ITT populations are shown in Table S3.

### Clinical study data analysis: Safety endpoints

Treatment-emergent adverse events (TEAEs) of grade ≥3 were consistent with the known safety profile of mFOLFIRINOX. The most common grade ≥3 TEAEs included neutropenia (26%; 18 patients), hypokalemia (16%; 11 patients), anemia (13%; nine patients), and thrombocytopenia (11%; eight patients). Five patients (7%) discontinued treatment due to TEAEs: one each for condition aggravated, fatigue, chest pain, and headache; general deterioration in physical health; pneumonia; and infusion-related reaction (pruritus). A comprehensive overview of all TEAEs, including those resulting in treatment discontinuations, TEAEs of grade ≥3, and mitazalimab-related TEAEs, is provided in Tables S4 and S5.

### Genomic alterations correlate with clinical efficacy outcomes

**Figure 2:**
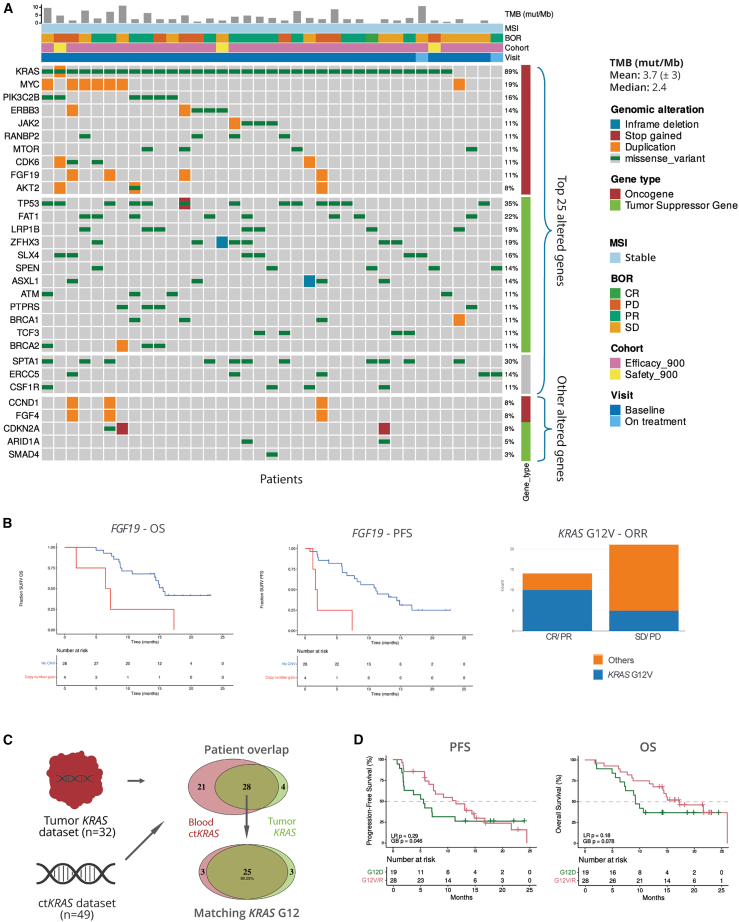
Genomic alterations associated with clinical efficacy endpoints (A) Oncoprint from the TSO500 dataset summarizing somatic genomic alterations in 37 patients who had received the recommended phase 2 dose of 900 μg/kg mitazalimab with available targeted DNA-sequencing data. The three upper panels depict the top 25 most frequently altered genes, as defined by somatic gene variants and copy number variation (CNV; duplication/deletion). The lower panel displays five frequently mutated genes according to literature.^23^^,^^24^^,^^31^^,^^32^^,^^33^ Tumor mutational burden (TMB), microsatellite instability (MSI), best overall response (BOR), cohort, and visit are included as column annotations. Genes were ordered based on their function (oncogene or tumor suppressor, as defined by oncoKB database)^34^^,^^35^, and the frequency of alteration. (B) Genomic alterations associated with overall survival (OS), objective response rate (ORR), and progression-free survival (PFS) with an unadjusted *p* value of <0.05. (C) Derivation of the KRAS subtype consensus dataset for patients in the full analysis set (FAS; *n* = 50); patient overlap between TSO500 and circulating tumor DNA datasets (top) and intersection of KRAS G12 variants among shared patients in both datasets (bottom). (D) OS and PFS comparison of patients from the FAS stratified according to KRAS G12V/R and G12D variants from the consensus dataset. CR, complete response; GB, Gehan-Breslow; LR, log rank; mut/Mb, mutation per megabase; PD, disease progression; PR, partial response; SD, stable disease; SURV, survival.

Genomic alterations, including *KRAS* status, high microsatellite instability (MSI) and tumor mutational burden (TMB), can significantly impact outcomes in PDAC.^17^^,^^18^^,^^19^^,^^29^^,^^30^ Therefore, we conducted a genomic analysis on 37 of 57 patients (65%) with evaluable baseline tumor biopsies who received 900 μg/kg of mitazalimab revealing *KRAS* as the most frequently mutated oncogene (89%) and *TP53* as the most commonly mutated tumor suppressor gene (35%) (Figure 2A; Figure S2). All evaluable patients were classified as microsatellite stable, with a median TMB of 2.4 mut/Mb. No patients were classified as TMB-high (Figure 2A).^29^

To investigate the potential of genomic alterations as prognostic biomarkers, we analyzed their association with clinical efficacy endpoints. Using a nominal *p* value threshold of 0.05 (unadjusted for multiple comparisons), 17 genomic alterations (out of 523 assessed) were found to be associated with clinical efficacy endpoints, including overall response (OR), duration of confirmed response, OS, PFS, OS rate at 12 months (OS-12), PFS rate at 12 months (PFS-12), and DCR (Tables S6 and S7). Among these, alterations in *KRAS*, *FGF19*, *ERCC5*, and *SPEN* loci were identified (Figure 2B; Table S6). Specifically, *FGF19* duplication correlated significantly with shorter OS and PFS, and reduced the likelihood of disease control (Figure 2B). The *KRAS* G12V variant was more prevalent in treatment responders (CR/PR) compared to non-responders (stable disease/PD) (Figure 2B). To further test the association of OS and PFS and *KRAS* G12 variants, we utilized a consensus *KRAS* status dataset that harmonized tumor *KRAS* DNA-sequencing and *KRAS* G12 ctDNA typing (*n* = 50, Figure 2C; Table S8). Comparative analysis of G12V/R to G12D mutations revealed that patients harboring G12D mutations exhibited a trend toward shorter OS, while PFS was significantly shorter at early time points, as determined by the Gehan-Breslow test (Figure 2D).

### Baseline molecular PDAC subtype associates with survival benefit

**Figure 3:**
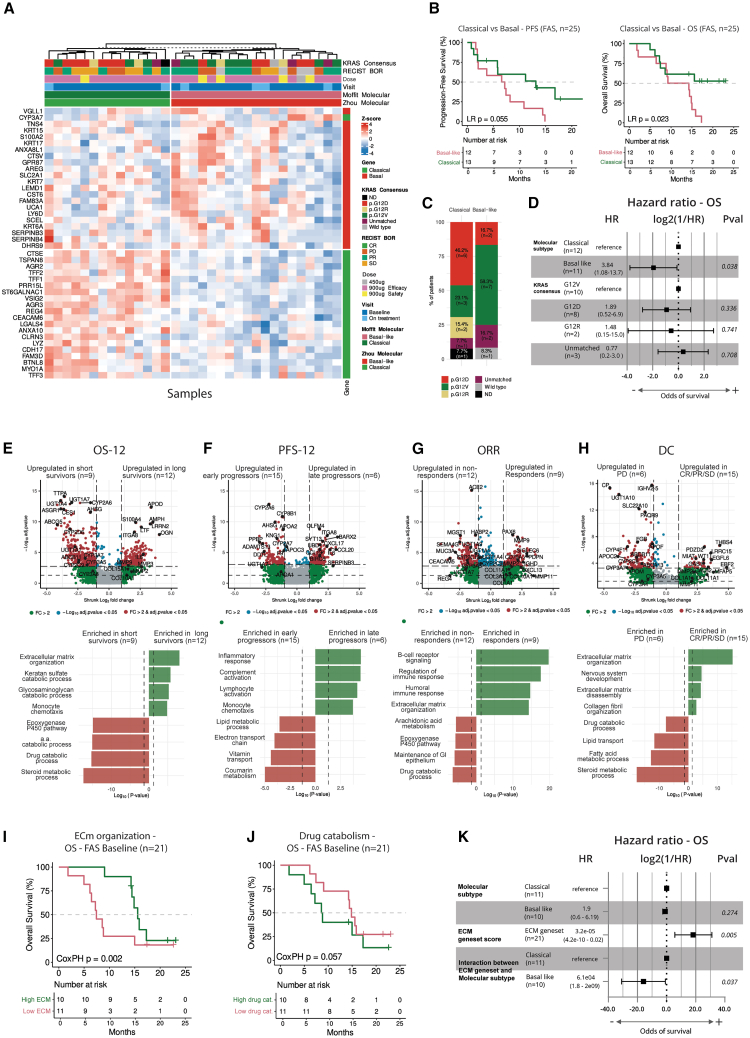
Transcriptomic profiles of patients in OPTIMIZE-1 (A) Molecular subtype classification into “classical” and “basal-like” profiles (*n* = 33 samples; *n* = 31 patients) using the gene set published by Moffit et al.^21^ (B) Overall survival (OS) and progression-free survival (PFS) differences in the full analysis set (FAS) stratified by molecular subtype (*n* = 25). (C) Overlap between KRAS G12 and molecular subtypes. (D) Forest plot derived from a multivariate cox proportional hazards (PH) model displaying the hazard ratio (HR) of OS. (E) Differentially expressed genes (DEGs) and enriched pathways based on OS at 12 months (OS-12; long survivors, post-12 months; short survivors, pre-12 months). (F) DEGs and enriched pathways-based PFS at 12 months (PFS-12; late progressors, post-12 months; early progressors, pre-12 months). (G) DEGs and enriched pathways-based on overall response (OR; responders considered complete response [CR]/partial response [PR]; non-responders considered stable disease [SD]/disease progression [PD]). (H) DEGs and enriched pathways-based on disease control (DC; CR/PR/SD vs. PD). (I) OS differences based on extracellular matrix (ECM) remodeling organization gene set scores of patients from the FAS at baseline. (J) OS differences based on drug catabolic processes gene set scores of patients from FAS at baseline. (I and J) Contributions were tested via univariate cox PH using ssGSEA gene set scores as continuous numerical data, but the scores were represented as a categorical variable by stratifying the patients according to the median (high: >median, low: <median). (K) Forest plot derived from a multivariate cox PH model displaying the HR of OS. Molecular subtype was analyzed as a categorical variable with “classical” as reference group, while the ECM Gene set was analyzed as a continuous variable. aa, amino acid; BOR, best overall response; FC, fold change; GI, gastrointestinal; LR, log rank; ND, not determined.

The molecular subtype of mPDAC has emerged as a prognostic indicator of treatment response to FOLFIRINOX.^22^^,^^36^ Thus, we studied the influence of PDAC subtype by classifying patients into classical or basal-like molecular subtype (*n* = 33 samples, *n* = 31 patients, Figure S3) using gene signatures published by Moffit et al. (Figure 3A)^21^ and Zhou et al. (Figure S4A).^20^ This analysis revealed that 48% (*n* = 15) of patients had a classical subtype and 52% (*n* = 16) had a basal-like subtype. Among the 31 patients with molecular subtype classification, 25 were included in the FAS and were stratified by molecular subtype. Patients with classical mPDAC tumors demonstrated longer OS compared to those with a basal-like subtype (Figure 3B). In the ITT population (*n* = 30), patients with the classical subtype had significantly longer OS and PFS compared to those with the basal-like subtype (Figure S4B).

We then assessed the relationship between *KRAS* status and molecular subtype by conducting a confounding factor analysis using the consensus *KRAS* G12 status dataset and molecular subtype classifications. Although the G12D *KRAS* variant was more prevalent in the classical subtype (6/13), and the G12V in the basal-like subtype (7/12), this association was not significant (Fisher’s exact test, *p* value = 0.11) (Figure 3C). We then assessed the prognostic power of both variables using a multivariate cox proportional hazards (PH) test. In the OS analysis, a basal-like subtype was associated with worse survival compared to those with classical subtype tumors (HR = 3.84, *p* value = 0.038) (Figure 3D). Similarly, *KRAS* G12D and G12R mutations were associated with worse survival compared to the G12V subtype, although these differences were not statistically significant (*p* value = 0.336 and 0.741, respectively).

### Presence of a fibrosis-related gene signature in baseline tumors associates with survival benefits

We hypothesized that biological determinants of the transcriptional profile of baseline PDAC tumors would associate with clinical efficacy. To test this, we conducted a differential gene expression analysis (DGEA) followed by pathway enrichment analysis using RNA sequencing (RNA-seq) data from baseline tumor biopsies obtained from the FAS patients (*n* = 21, see Figure S3; Table S9) Analysis of survival at 12 months (OS-12) revealed that long survivors (>12 months) exhibited overexpression of genes involved in immune-modulatory pathways (*S100A4* and *IL6*), and extracellular matrix (ECM) remodeling (*MMP2*, *MMP9*, and *COL15A1*) (Figure 3E). Conversely, short survivors (<12 months) displayed increased presence of pathways related to drug and steroid metabolic processes (*UGT1A4*, *UGT1A7*, and *UGT1A1*), primarily through the cytochrome P450 (CYP450) pathway (*CYP2A6*, *CYP3A7*, and *CYP3A5*), as well as lipid and drug efflux pumps (*ABCG5* and *ABCB11*) (Figure 3E). TME deconvolution analysis revealed that long survivors had higher baseline scores of cancer-associated fibroblasts and T cells, along with lower scores of drug and fatty acid metabolism (Figures S4C and S4D).

**Figure 4:**
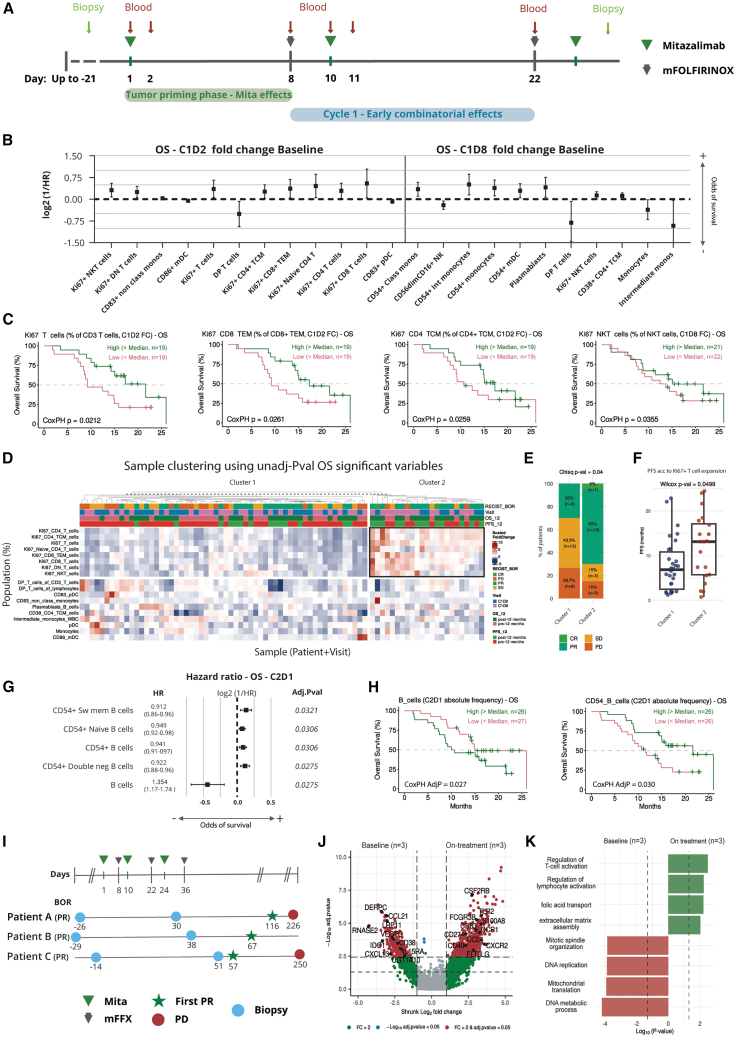
Tumor extrinsic factors associated with clinical efficacy endpoints during the first cycle of OPTIMIZE-1 (A) Dosing and sampling regimen in OPTIMIZE-1. (B) Forest plot derived from univariate cox proportional hazards (PH) models displaying fold change (FC) in immune cell frequencies during the tumor priming phase of OPTIMIZE-1 (*p* < 0.05), associated with the odds of survival (log2[−HR]). (C) Mitazalimab-induced FC in immune cell frequency associated with overall survival (OS) during the tumor-priming phase of OPTIMIZE-1 (*p* < 0.05). Sample numbers are based on data availability for each population. (D) Sample clustering based on FC in immune cell frequency, induced during the tumor-priming dose of mitazalimab, associated with OS (*p* < 0.05). Samples clustered via K-means. (E) Differences in best overall response (BOR) in patients stratified by the induction of T cell proliferation in peripheral blood, induced by the tumor-priming dose of mitazalimab. (F) Differences in progression-free survival (PFS) in patients stratified by the induction of T cell proliferation in peripheral blood, induced by the tumor-priming dose of mitazalimab. Data are represented as median ± range. (G) Forest plot derived from univariate cox PH models displaying cycle (C) 2, day (D) 1 levels of immune cell frequencies and cytokines associated with the odds of survival (log2[−HR]), during the first treatment cycle of OPTIMIZE-1 (adjusted *p* value < 0.05). (H) Immune cell frequency levels on C2D1 associated with OS (adjusted *p* value < 0.05), induced by the combination of mitazalimab and mFOLFIRINOX (mFFX) during the first treatment cycle of OPTIMIZE-1. Sample numbers are based on data availability for each population. (I) Intratumoral transcriptomic changes driven by mitazalimab in combination with mFFX during treatment. Paired tumor biopsies from three patients that achieved a partial response (PR) as BOR were analyzed. (J) Differentially expressed genes (DEGs) from paired biopsies. (K) Enriched pathways based on paired biopsies. CR, complete response; DN, double-negative; DP, double-positive; HR, hazard ratio; int, intermediate; mDC, myeloid dendritic cells; Mita, mitazalimab; NK, natural killer; NKT, natural killer T cells; non class monos, non-classical monocytes; PD, disease progression; pDC, plasmacytoid dendritic cells; SD, stable disease; TCM, T central memory; TEM, T effector memory.

We next stratified patients by PFS-12 and found that tumors from late progressors (>12 months) were enriched for genes involved in general inflammatory responses (*CXCL17*, *IL6*, and *CD40*), monocyte chemotaxis (*CCL2*, *CCL19*, and *CCL22*), and complement activation (*C1QB*, *C1QA*, and *IGHV*). Early progressors (<12 months) overexpressed genes associated with lipid and coumarin metabolic pathways (*CYP2A6*, *CYP2A7*, *UGT1A7*, *APOA1*, *APOA2*, and *APOC3*) (Figure 3F). Patients who responded to treatment or achieved disease control demonstrated overexpression of genes involved in ECM remodeling and collagen fibril organization (*COL1A1*, *COL11A1*, *COL3A1*, *MMP2*, *MMP9*, and *MMP11*) (Figures 3G and 3H). Responders particularly overexpressed genes related to regulation of immune responses and B cell biology (*CXCL13*, *SIGLEC6*, *CD79A*, and *IGHD*) (Figure 3G). Tumors from non-responders and quick progressors were characterized by an enrichment of pathways involved in drug and steroid metabolic processes via the CYP450 system (*CYP2A6*, *CYP3A7*, and *CYP3A5*), as well as drug efflux pumps and inactivating enzymes (*ABCG5*, *ABCB11*, *UGT1A4*, and *UGT1A7*) (Figures 3G and 4H).

To assess the predictive power of these processes, we generated gene set scores and tested their influence on OS using univariate cox PH analysis. A high score in the ECM organization gene set was significantly associated with longer OS, including genes involved in ECM remodeling such as *MMP2* and *MMP14* (Figure 3I; Figure S4E). Conversely, a high score in the drug catabolism gene set showed a trend toward shorter OS (Figure 3J). Finally, we evaluated the association of the ECM organization gene set and molecular subtype with OS using a multivariate cox PH test (Figure 3K). Higher ECM gene set scores were associated with a greater probability of survival. Patients with the classical tumor subtype displayed a lower HR compared to those with the basal-like tumor subtype. The interaction between ECM organization and molecular subtype was also found to be significant (Figure 3K). The ECM gene set emerges as a promising predictive biomarker for patients with mPDAC undergoing treatment with mitazalimab and mFOLFIRINOX.

### Mitazalimab-induced changes in peripheral immune cell populations associate with clinical efficacy outcomes

**Figure 5:**
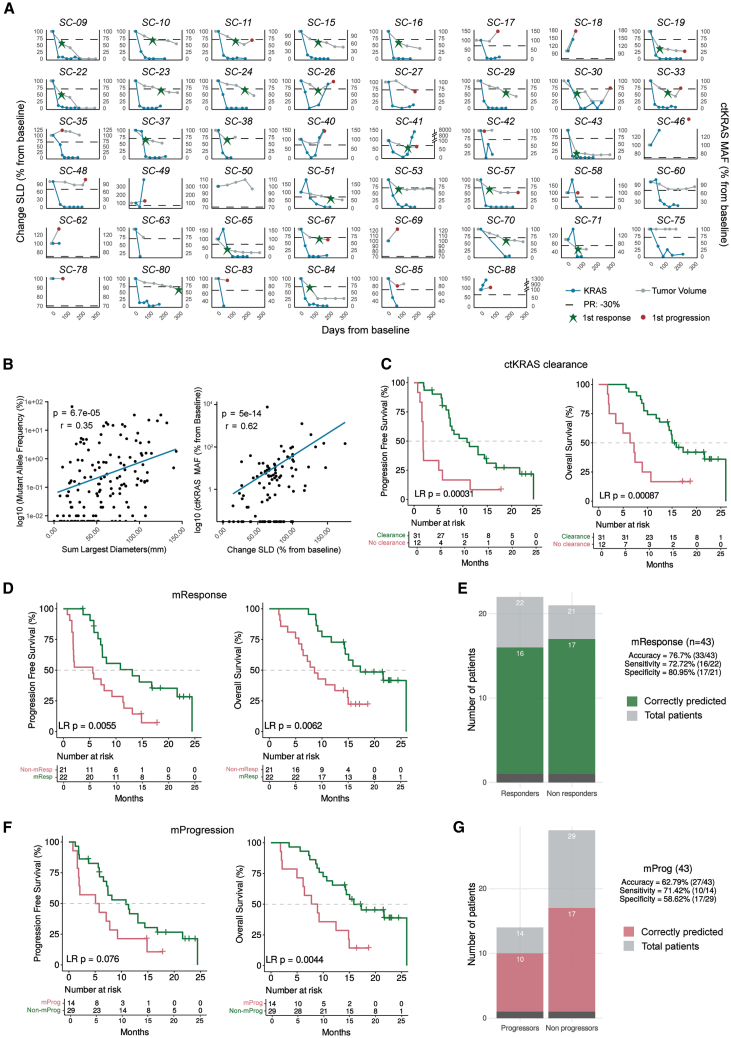
Correlation of ctDNA with tumor burden and association with clinical efficacy endpoints (A) Longitudinal changes in tumor volume, expressed as sum of longest diameters (SLD), and ctKRAS levels, displayed as % mutant allele frequency (MAF), from baseline during the first 300 days of treatment. (B) Correlation of SLD and log10 (%MAF) in absolute numbers (left), and percentage from baseline (right). Differences in PFS and OS after stratifying patients according to ctKRAS. (C) ctKRAS clearance. (D) Molecular Response. (E) Metrics of the mResponse predictor. (F) Molecular Progression. (G) Metrics of the mProgression predictor. PR, partial response.

The unique design of OPTIMIZE-1, incorporating a tumor-priming dose of mitazalimab prior to chemotherapy,^16^ allowed us to study the correlation between CD40 stimulation and survival benefits (Figure 4A, “Tumor-priming phase—Mita effects”). Analysis of mitazalimab-induced immunophenotype changes during the tumor-priming phase, measured by fold change (FC) from baseline, identified 23 immune cell populations associated with OS at a nominal *p* value threshold of 0.05 (Figure 4B). Notably, increasing percentages of proliferating T cell and NK cell populations, including Ki67^+^ T cells, Ki67^+^ CD8 T effector memory cells, Ki67^+^ CD4 T central memory cells, and Ki67^+^ natural killer T (NKT) cells, were associated with longer OS (Figure 4C). Further analysis revealed 18 immune cell populations associated with PFS at a nominal *p* value threshold of 0.05, with seven at C1D2 and 11 at C1D8. Increased FC at C1D2 of Ki67^+^ T cells and Ki67^+^ naive CD4 T cells, as well as FC at C1D8 of Ki67^+^ NK cells and plasmablasts, correlated with longer PFS (Figures 5A and 5B). A subset of patients exhibiting an expansion of proliferating Ki67^+^ T cell populations at C1D2 and C1D8 showed significantly higher ORR and longer PFS compared to those without Ki67^+^ T cell expansion in circulation (Figures 4D–4F). Overall, these findings suggest a potential direct contribution of activation and proliferation of circulating T cells and NK cells, induced by the initial tumor-priming dose of mitazalimab, to improved clinical outcomes.

Following on from this, we evaluated the “early combinatorial effects” of mitazalimab and mFOLFIRINOX on immune phenotypes, cytokines and clinical outcomes (Figure 4A) by analyzing immune phenotypic associations at the end of cycle 1 with survival benefits. At an adjusted *p* value of 0.05, increased absolute frequency levels of four CD54^+^ B cell populations and decreased total B cell frequency were associated with improved OS (Figures 4G and 4H). Additionally, six B cell subsets as well as interleukin (IL)-6 were associated with shorter PFS (Figures 5C and 5D). The observed activation of B cells and activation-induced margination of B cells are hallmarks of a CD40 agonist response and suggest that increased CD40 stimulation in response to mitazalimab supports improved survival outcomes.

### Mitazalimab + mFOLFIRINOX induced intratumoral myeloid cell activation in patients with an objective response

To assess treatment-induced changes in the TME, we performed RNA-seq analysis on paired biopsies from three patients with a PR (Figure 4I). Intratumoral transcriptomic changes following treatment were identified through DGEA comparing on-treatment and baseline samples. The analysis identified 1,207 differentially expressed genes (DEGs) (Figure 4J). After treatment with mitazalimab and mFOLFIRINOX, there was an upregulation of genes involved in myeloid cell biology (*CSF2RB*, *TLR2*, *S100A8*, *FLT3LG*, *CCR1*, and *FCGRIIIB*) and regulation of T cell responses (*CD83*, *CD274*, and *CCL4*). Concurrently, downregulation was seen in immunosuppressive genes (*IDO1*, *CXCL13*, and *IL15RA*) as well as *VEGFC*, *CCL21*, and *CD38*. Pathway enrichment analysis, conducted at a nominal *p* value threshold of 0.05, revealed significant enrichment in lymphocyte-related pathways (Figure 4K). Due to the limited number of paired biopsies obtained from the study, we conducted an additional analysis using a PDAC (KPCY) tumor bearing hCD40tg mouse model to define systemic and intratumoral effects of mitazalimab in combination with FOLFIRINOX (Figure S5E). This model closely recapitulated the clinical and systemic events observed in patients with mPDAC (Figures 5F–5H). Notably, mitazalimab-treated mice exhibited an increase in intratumoral Ki67^+^ effector CD8^+^ T cells, monocytes, and macrophages. This was accompanied by an upregulation of granzyme B (GzmB) in cytotoxic cells, including effector, CM, and naive CD8^+^ T cells, as well as NK cells (Figure S5I). Together, the data support the role of myeloid cells and T cells in the treatment response to mitazalimab when combined with mFOLFIRINOX.

### Early on-treatment changes in ctKRAS levels as a prognostic indicator

To assess circulating KRAS (ctKRAS) as a surrogate biomarker for tumor burden in FAS patients, *KRAS* mutant allele frequency (MAF) was determined in ctDNA obtained from blood samples (*n* = 46) and compared with the sum of the longest diameter (SLD) of tumor lesions over the first 300 days of treatment (Figure 5A; Figure S6). Fluctuations in %MAF of ct*KRAS* coincided with changes in % SLD, although with varying magnitudes and temporal offsets. The relationship between SLD and %MAF of ct*KRAS* was exponential, as evidenced by the strong linear relationship observed between SLD and log_10_ (%MAF), as well as between percent change in SLD and %MAF from baseline (Figure 5B; Figure S7A).

We subsequently tested the relationship between longitudinal changes in ct*KRAS* levels and clinical benefit from treatment (*n* = 43, Figure S6). Clearance of ct*KRAS* at any time point within the first 225 days was significantly associated with improved OS and PFS (Figure 5C). ct*KRAS* clearance was achieved in 72% of patients (31/43). Stratification by ct*KRAS* G12 mutation status revealed that patients with the *KRAS* G12V variant reached ct*KRAS* clearance more frequently than those with the *KRAS* G12D variant (17/20 vs. 9/18 respectively, Fisher test *p* value = 0.0354). Furthermore, ct*KRAS* clearance was significantly associated with longer OS and PFS in G12D patients (Figures S7B–S7E), while G12V patients with ctKRAS clearance showed significantly longer PFS and a trend toward longer OS (Figure S7F).

Further, patients exhibiting a molecular response (mResponse), defined as at least 90% reduction in %MAF within the first 70 days of treatment, demonstrated longer OS and PFS compared to non-mResponders (Figure 5D). Similar trends were observed when stratifying patients according to *KRAS* G12 subtype (Figures S7G and S7H). The accuracy of mResponse in predicting radiological response, as assessed by conventional computed tomography (CT) scan, was 76.7%; with a sensitivity of 72.7% and a specificity of 81.0% (Figure 5E; Table S10). On average, ct*KRAS* mResponse was detected 47.2 ± 30.8 days before radiological response as per Response Evaluation Criteria in Solid Tumors (RECIST) criteria. Conversely, patients experiencing molecular progression (mProgression), defined as at least 10% increase in ct*KRAS* %MAF from a previous measurement within the first 225 days of treatment, had significantly shorter OS and PFS compared to non-mProgressors (Figure 5F). Similar trends were observed when stratifying by *KRAS* G12 subtype (Figures S7I and S7J). The accuracy of mProgression in predicting radiological progression, as determined by conventional CT scan, was 62.8%, with a sensitivity of 71.4% and a specificity of 58.6% (Figure 5G; Table S11). On average, ct*KRAS*-based mProgression was detected 39.1 ± 44.6 days before radiological progression per RECIST criteria.

## Discussion

In this study, we report updated safety and efficacy data on mitazalimab in combination with mFOLFIRINOX in the OPTIMIZE-1 trial. The correlative analyses, including RNA sequencing of tumor tissue and cytokine/flow cytometry analysis of peripheral blood, were exploratory and hypothesis-generating. Using this multi-omic approach, we identified determinants associated with treatment outcomes. Notably, we discovered a fibrosis-related gene signature that may help identify patients with a better outcome to treatment with mitazalimab plus mFOLFIRINOX in terms of OS. Additionally, mitazalimab-induced activation of peripheral immune cell populations—including T cells, B cells, NK cells, and myeloid cells—was associated with longer survival. Findings also show that treatment triggers changes in ctDNA that both precede and associate with clinical outcomes. Collectively, these biological effects induced by mitazalimab align with the hypothesized mechanism of anti-tumor activity for a CD40 agonist when combined with chemotherapy and support the treatment approach implemented in the OPTIMIZE-1 trial.

In this analysis, based on a median follow-up of 18-month, the confirmed ORR increased from 40% at 12 months to 42% due to one additional late responder. The median DoR was 12.6 months representing a 2-fold improvement compared to FOLFIRINOX and a 50% increase over NALIRIFOX.^3^^,^^16^^,^^37^ PFS remained superior to historical benchmarks, and the mature 18-month OS rates improving to twice that of historical controls and surpassing current standard of care expectation.^37^^,^^38^^,^^39^ Importantly, no new safety signals emerged since the last update.^16^ These results suggest encouraging efficacy and a manageable safety profile of mitazalimab in combination with mFOLFIRINOX in the treatment of mPDAC; however, confirmation in a planned randomized phase 3 study is warranted to establish the benefit of this regimen.

Multiple CD40 agonists have been investigated in clinical trials for pancreatic cancer, as recently reviewed by McVey et al.^40^ These agonists can be broadly categorized based on their requirement for concurrent interaction with Fc gamma receptors (FcγR): some are FcγR-conditional (e.g., sotigalimab and mitazalimab), while others are non-conditional (e.g., selicrelimab and CDX-1140).^41^ Among the FcγR-conditional agonists, differences in structure of the Fc region further influence their clinical activity. For example, mitazalimab has a wild-type IgG1 Fc, whereas sotigalimab features a mutated Fc with enhanced affinity for FcγR2b. These structural and functional distinctions may impact both safety and efficacy profiles.^41^

The phase 2 PRINCE trial, evaluating sotigalimab in combination with chemotherapy (gemcitabine/nab-paclitaxel) in patients with mPDAC, reported negative results for the chemo/CD40 combination arm.^14^^,^^42^ The PRINCE trial differed from OPTMIZE-1 in several important aspects, including the chemotherapy backbone (gemcitabine/nab-paclitaxel vs. mFOLFIRINOX) and the sequence of agent administration (sotigalimab was given after chemotherapy in PRINCE, whereas mitazalimab was administered as a priming dose prior to the first dose of chemotherapy in OPTIMIZE-1). The priming dose of mitazalimab in OPTIMIZE-1 was specifically designed to leverage CD40-mediated degradation of tumor stroma before the initiation of chemotherapy, which may enhance drug delivery and efficacy. While direct comparisons between studies are challenging due to differences in trial design, patient populations, and dosing strategies, it is noteworthy that the development of other CD40 agonists has not advanced, largely due to limited efficacy signals in clinical trials. In contrast, the safety, efficacy, and pharmacodynamic data from OPTIMIZE-1 suggest that mitazalimab, in combination with mFOLFRINOX and with an optimized dosing schedule, may offer a more favorable therapeutic profile. These findings support the rationale for further investigation of mitazalimab in this setting and highlight the importance of both agent selection and trial design in the development of CD40-targeted therapies for pancreatic cancer.

The mutational profile of patients in OPTIMIZE-1 aligns with previously reported patterns in mPDAC.^23^^,^^24^ *KRAS* was the most frequently altered gene, with single nucleotide variants (SNVs) at position G12 being the predominant alteration, consistent with earlier studies.^23^^,^^43^ Patients with the G12V variant demonstrated increased response rates and, when combined with those carrying the G12R variant, a longer PFS compared to individuals with the G12D variant. However, no statistically significant difference in OS was observed between G12V/R variants and G12D carriers, contrasting with some reports.^17^^,^^18^^,^^19^ The relationship between *KRAS* G12 status and tumor molecular subtype (classical and basal-like) emerged as a potential confounding factor in our analysis. Although the molecular subtype classification in OPTIMIZE-1 was largely consistent with previous data, *KRAS* G12D showed partial, yet non-significant, enrichment in patients with the classical subtype. This observation differs from previous findings suggesting independence between *KRAS* G12 status and molecular subtype.^21^^,^^36^ Multivariate Cox PH analysis confirmed molecular subtype as a prognostic factor for OS, whereas *KRAS* G12 subtype was not. These findings suggest that molecular subtype exerts a stronger prognostic influence than *KRAS* G12 status, as proposed by Singh et al.,^36^ or that our analysis lacks sufficient power to detect OS differences for individual G12 variants. Furthermore, they highlight the complex role of genetic mutations and subtypes in mPDAC, stressing the need for more research on their impact on immunotherapy.

The desmoplastic stroma in PDAC plays a key role in immune exclusion and treatment resistance.^4^ In OPTIMIZE-1, a priming dose of mitazalimab was introduced prior to chemotherapy to activate CD40-expressing myeloid cells facilitating stromal degradation and enhancing the efficacy of chemotherapy.^15^^,^^44^ A key finding in this study is the identification of a baseline, fibrosis-related gene signature, associated with ECM organization and linked to survival benefits. Moreover, key genes involved in matrix remodeling, such as MMP2 and MMP14, were significantly associated with prolonged OS. This aligns with preclinical data indicating that tumors with high MMP2 and MMP14 expression are more likely to respond to CD40 agonists in combination with chemotherapy.^15^ These findings support ECM remodeling as a potential predictive biomarker for response to mitazalimab plus mFOLFIRINOX, endorsing the underlying hypothesis and rationale for the priming dose of mitazalimab. Further studies, including orthogonal methods such as multiplex immunohistochemistry (IHC) approaches, are needed to validate the predictive utility of this fibrosis-related gene signature, which represents a promising biomarker candidate. Notably, no published data are available to demonstrate that this specific fibrosis signature predicts outcomes with mFOLFIRINOX alone, yet its predictive relevance for mitazalimab cannot be definitively established without a comparator group.

In addition to identifying potential determinants of treatment response, gene expression analysis of baseline tumors revealed a distinct signature associated with poor treatment outcomes to mitazalimab in combination with mFOLFIRINOX. This signature, characterized by overexpression of steroid metabolism, drug catabolism, and epoxygenase p450 pathways, was detected in short-survivors, early progressors and non-responders. These pathways encompass multiple chemoresistance mechanisms that may lead to the inactivation^45^^,^^46^^,^^47^ or enhanced efflux^48^^,^^49^ of mFOLFIRINOX components. Collectively, they contribute to a complex network of resistance mechanisms associated with poor prognosis, independently of the mitazalimab component in the treatment regimen.

In OPTIMIZE-1, the first dose of mitazalimab is administered prior to chemotherapy. This approach contrasts prior studies wherein CD40 is delivered after the first dose of chemotherapy.^42^^,^^44^ As a consequence of the study design, OPTIMIZE-1 offers a unique opportunity to define the immunological effects induced by CD40 agonist monotherapy and their association with survival. This is relevant given that chemotherapy can negatively affect the quality and quantity of CD40-expressing myeloid cells.^50^ Previous reports have described longitudinal immune phenotype changes in OPTIMIZE-1.^16^ Herein, we show the association of these mitazalimab-induced changes with survival benefits. Notably, the frequency of activated antigen-presenting cells (APCs) and proliferating T cell and NK cell subsets triggered by mitazalimab correlates with longer OS, PFS and a higher response rate. These findings suggest that mitazalimab induces rapid activation and mobilization of APCs in the circulation, leading to the secretion of key stimulatory cytokines—interleukin (IL)-12, TNF-α, and interferon (IFN)-γ—within 24 h, which in turn drives T cell expansion in peripheral blood.^10^^,^^16^^,^^51^^,^^52^ Our results deviate from those recently published by Padrón and colleagues, who found no significant correlation between T cell proliferation, monocyte activation, and survival.^13^ In addition, we did not observe any baseline immunophenotypic signatures that associated with improved survival.^13^ These discrepancies may be due to differences in the timing and sequence of CD40 agonist administration with respect to chemotherapy and their impact in shaping anti-tumor immune responses.^10^

We identified associations between immune phenotype changes and survival following the first full treatment cycle in OPTIMIZE-1. These “early combinatorial” treatment-induced changes included increases in the relative frequency of CD54^+^ B cells and a decrease in the overall frequency of B cells, both of which were associated with improved survival. CD54, a molecule involved in leukocyte extravasation and previously used as a biomarker for APCs in immunotherapy, may indicate activation, extravasation, and migration of APCs into peripheral tissues when its frequency increases relative to the total leukocyte pool.^53^^,^^54^ Additionally, cytokine analysis revealed that patients with low IL-6 levels at C2D1 had longer PFS, consistent with findings from similar studies on other CD40 agonists.^13^^,^^50^ Notably, the mitazalimab-induced elevation in IL-6 levels observed in OPTIMIZE-1 was lower compared to those reported with other CD40 agonists such as selicrelumab, an effect attributed to the design of mitazalimab.^52^^,^^55^ This finding reinforces both the efficacy and, importantly, the favorable safety profile of mitazalimab, as IL-6-driven immunosuppression is associated with mild AEs including pyrexia, rigors, headaches, and infusion-related reactions.^16^^,^^56^ Collectively, these findings underscore that the CD40-mediated immunologic responses induced by mitazalimab are associated with improved survival.

Analysis of paired tumor biopsies revealed significant treatment-induced changes in the TME following mitazalimab and mFOLFIRINOX therapy. Notably, an upregulation of myeloid APC-associated and T cell modulatory genes was observed, indicative of successful recruitment and expansion of myeloid APCs in tumors. Concurrently, a decrease in gene set scores related to extracellular vesicle and exosome assembly and secretion was found, potentially associated with reduced tumor size. This result shows an increase in myeloid APCs following treatment with a combination of CD40 agonist and chemotherapy in mPDAC. Although the low number of paired biopsies available for this study (3) limits the interpretation of these findings, they align with the qualitative increase of macrophages reported by Padrón et al. after sotigalimab treatment and additional earlier studies in the neoadjuvant setting.^13^

ctDNA is an emerging, minimally invasive, biomarker with the potential to predict response or resistance before radiological assessment, as well as to enable more precise monitoring of ongoing treatment efficacy. Importantly, ctDNA is an established marker of chemotherapy response in pancreatic cancer.^26^^,^^27^ In OPTIMIZE-1, *KRAS* was the most frequently mutated oncogene, consistent with prior reports.^24^^,^^33^ We, and others, have previously shown that the predominant alteration is a SNV at position G12.^16^^,^^23^^,^^43^ While baseline ct*KRAS* levels did not significantly correlate with OS across all patients, stratification by *KRAS* G12 status revealed that increasing baseline ct*KRAS* MAF levels correlated with lower OS and PFS in G12V, but not G12D patients. This contrasts with results from the PRINCE and SOC cohorts reported by Till et al., who observed an association between ct*KRAS* %MAF levels and OS and PFS in patients with the G12D variant only, albeit in relatively small cohorts.^25^ Importantly, ct*KRAS* clearance following treatment was associated with longer OS and PFS, consistent with earlier studies.^25^ Patients achieving mResponse or those without mProgression showed benefits in OS and PFS, with these molecular changes preceding radiological assessments by 47 and 39 days, respectively. These observations highlight the potential of ctDNA analysis as an early predictor of treatment outcomes in mPDAC, but further studies will be needed to determine the specific contribution of mitazalimab to changes in ctDNA observed with chemoimmunotherapy in mPDAC.

In conclusion, mitazalimab, in combination with mFOLFIRINOX, continues to exhibit a manageable safety profile and promising clinical activity, with a favorable DoR contributing to a meaningful survival benefit. Through comprehensive multi-omic analyses, we have identified both tumor-intrinsic and -extrinsic biomarkers associated with improved survival. Of particular significance is the fibrosis-related gene signature, which appears to be directly linked to the expected mechanism of action of mitazalimab and holds potential as a predictive biomarker, pending further confirmatory studies. Finally, the immune phenotypic changes induced by mitazalimab align with the expected effects of a CD40 agonist, and their association with survival suggests that mitazalimab plays a role in driving the observed clinical benefit. While the identified biomarkers need to be confirmed in an independent dataset, these findings and biomarker associations provide evidence for a mitazalimab-driven contribution to the response observed in OPTIMIZE-1, supporting its further development.

### Limitations of the study

While the study findings provide valuable insights into the clinical activity and biomarker landscape of mitazalimab in combination with mFOLFIRINOX, several limitations should be acknowledged.

The single-arm study design restricts the ability to directly attribute observed clinical benefits to mitazalimab. In addition, the OPTIMIZE-1 population differed from those in the PRODIGE and MPACT trials,^37^^,^^38^ with a higher proportion of female patients (58% vs. 38%–43%) and fewer patients with liver metastases (74% vs. 85%–88%); however, ORR and OS were similar between males and females (see Table S12).

The limited number of samples, including paired biopsies, and the variability in sample sizes for the different exploratory analysis impacts the generalizability of the findings, the statistical power of subgroup analyses, and the ability to comprehensively assess intratumoral changes at an individual level. As a result, further validation of the identified biomarkers in independent cohorts is required. In addition, the deconvolution of the TME using bulk RNA-seq did not allow for detailed characterization of cell populations (e.g., T cells, myeloid cells, and fibroblasts), nor was orthogonal validation using IHC possible due to limited biopsy material. More comprehensive phenotyping and validation using additional methods will be important in future studies. Similarly, given the limited sample size, multiple comparisons, and lack of a validation cohort, the peripheral blood analyses are exploratory. Findings will need to be validated in larger, independent cohorts that include appropriate control arms.

While a retrospective control group of patients treated with mFOLFIRINOX might have been informative, the marked heterogeneity of PDAC tumor and stromal biology, as well as limited sample availability, complicates the feasibility of this approach. As a result, the subgroup analyses for OS should be considered exploratory and hypothesis-generating only, and these findings require validation in larger, independent cohorts to establish their clinical significance. The heterogeneity in biopsy timing relative to clinical response (between 6 and 86 days) could also influence the interpretation of longitudinal immune and transcriptomic changes. Importantly, both ctDNA clearance and classical versus basal molecular subtypes are established biomarkers of chemotherapy response. Thus, the contribution of mitazalimab to mFOLFIRINOX-induced changes in ctDNA remains unclear. Finally, despite the potential of ctDNA-based monitoring, challenges remain in defining its precise role in predicting clinical outcomes, underscoring the need for larger, well-controlled studies to refine the clinical utility of ctDNA as a predictive biomarker in mPDAC.

## Resource availability

### Lead contact

Further information and requests for resources and reagents should be directed to and will be fulfilled by the lead contact, Peter Ellmark (pek@alligatorbioscience.com).

### Materials availability

This study did not generate new unique reagents.

### Data and code availability

• RNA sequencing data are available in Gene Expression Omnibus at GEO: GSE291331.
• This manuscript does not report custom computer code.
• Any additional information required to reanalyze the data reported in this work paper is available from the lead contact upon request.

## Acknowledgments

This study and publication were funded by Alligator Bioscience AB. The authors thank the patients and their families and carers for their participation in OPTIMIZE-1. The authors would also like to thank all investigators and study site coordinators. Flow cytometry and cytokine associations with clinical efficacy endpoints were conducted by FIOS genomics (Edinburgh, UK). The authors would like to thank Tommy Schyman, BC Platforms, for statistical analysis. Medical writing support was provided by Kara McNair, MediComm Partners, which was sponsored by Alligator Bioscience AB in accordance with Good Publication Practice guidelines.

## Author contributions

Conceptualization, J.-L.V.L., P.E., K.E.S., S.V.A., Y.P.d.C., and D.G.J.; methodology, K.E.S., J.-L.V.L., P.E., S.V.A., Y.P.d.C., D.G.J., and G.L.B.; formal analysis, D.G.J., G.L.B., and Y.P.d.C.; investigation, E.M.O'R., G.L.B., D.G.J., J.-L.V.L., I..B, H.P., K.G., A.L., E.M., P.C., J.-F.B., L.P., J.F., M.R.G., R.A.P.-C., I.G., K.N., and T.M.M.; data curation, D.G.J.; writing – original draft, D.G.J., P.E., K.E.S., and Y.P.d.C.; writing – reviewing and editing, J.-L.V.L., K.G., I.B., T.M.M., A.L., P.C., H.P., E.M., J.-F.B., L.P., J.F., M.R.G., R.A.P.-C., I.G., K.N., E.M.O'R., D.G.J., G.L.B., P.E., K.E.S., Y.P.d.C., and S.V.A.; visualization, D.G.J.; project administration, P.E., K.N., and S.V.A.

## Declaration of interests

G.L.B. reports consulting and grant support to the University of Pennsylvania from Alligator Bioscience for the current manuscript; research grants to the University of Pennsylvania from Bristol Myers Squibb, Genmab, Incyte Corporation, and Hibercell; royalties/licenses from Novartis and Tmunity Therapeutics/Kite Pharma as the inventor of intellectual property related to CAR T cells licensed by University of Pennsylvania; consulting and advisory board fees from Molecular Partners, Shattuck Labs, EMD Serono, Pancreatic Cancer Action Network, and Alligator Bioscience; owns stock in Pfizer and Johnson & Johnson. P.C. reports institutional support from Alligator Biosciences for the current manuscript; institutional grants/contracts from Abbvie, ADCT, Adial Nortye, Amge, Astex, AstraZeneca, BMS, Boehringer Ingelheim, Blueprint, C4 Therapeutics, Debio Pharm Dalichi Sankyo, Dragonfly, Exelixis, Incyte, Iteos, Janssen, Kinnate, Lilly, Molecular Partners, Novartis, Ose Immunotherapeutics, Pierre Fabre, Relay, Roche/Genentech, Sotio, Taiho, Tango, Toray, Turningpoint, and Transgene; consulting fees from Boehringer Ingelheim, Ose Immunotherapeutics, Scenic, and Brenus; support for attending meetings from Novartis; support for participation on a DSMB/Advisory Board from BMS; and institutional support for receipt of equipment from Debio Pharm, Novartis, and Glaxo Smith Kline. J.F. reports grants/contracts from AMGEN, Roche, Astra Zeneca, Servier, and Viatris. R.A.P.-C. reports grants/contract from EU Horizon Program, Aragon Health Research Institute (IIS Aragon), and Institute de Salud Carlos III; consulting fees from Roche Astra Zeneca, BMS, Servier, Ipsen, and Astellas; payment or honoraria for lectures, presentations, speakers bureau, manuscript writing, or educational events from Astellas, Servier, Astra Zeneca, Roche, Eisai; payment for expert testimony from Servier, Astellas, Astra Zeneca; and support for attending meetings and/or travel from Servier, Astellas, Roche, Astra Zeneca, and Lilly. K.E.S., K.N., D.G.J., Y.P.d.C., P.E., and S.V.A. are employee and shareholder of Alligator Bioscience, AB. P.E. reports grant funding from Swedish Foundation for Strategic Research in his position as Adjunct Professor at Lund University. P.E., K.E.S., and D.G.J. are inventors on the patents relating to mitazalimab (patents owned by Alligator Bioscience). E.M.O.R. reports institutional grant support from Genentech/Roche, BioNTech, AstraZeneca, Arcus, Elicio, Parker Institute, NIH/NCI, Digestive Care, Break Through Cancer, Agenus, Amgen, and Rand evolution Medicines; consulting fees from Arcus, AstraZeneca, Ability Pharma, Alligator Bioscience, Agenus, BioNTech, Ipsen, Ikena, Merck, Morna Therapeutics, Novartis, Leap Therapeutics, Astellas, BMS, Revolution Medicines, Regeneron, and Tango; support for attending meetings and/or travel from BioNTech and Arcus; with other financial/non-financial interests from the American Association of Cancer Research, the American Society of Clinical Oncology, Imedex, Research to Practice, and SU2C.

## STAR★Methods

### Key resources table

**Table:** 

REAGENT or RESOURCE	SOURCE	IDENTIFIER
**Antibodies**		

APC anti-human/mouse Granzyme B Recombinant Antibody (clone QA16A02)	Biolegend	Cat.#372204 RRID: AB_2687027
Alexa Fluor™ 700 anti-mouse FOXP3 Monoclonal Antibody (clone FJK-16s)	eBioscience	Cat#56-5773-80 RRID: AB_1210557
BD Pharmingen™ PerCP-Cy™5.5 Rat Anti-Mouse CD4 (clone RM4-5)	BD Biosciences	Cat#550954 RRID: AB_393977
BD Pharmingen FITC Rat Anti-Mouse CD45 (clone 30-F11)	BD Biosciences	Cat#553080 RRID: AB_394609
BD Pharmingen PE Rat Anti-Mouse CD38 (clone 90)	BD Biosciences	Cat#553764 RRID: AB_395034
PE/Cyanine5 anti-mouse NK-1.1 (clone PK136)	Biolegend	Cat#108716 RRID: AB_493590
PE-Cyanine7 anti-mouse TCR beta Monoclonal Antibody (clone H57-597)	eBioscience	Cat#25-5961-82 RRID: AB_2573507
BD Horizon™ BV421 Rat Anti-Mouse CD8a (clone 53-6.7)	BD Biosciences	Cat#563898 RRID: AB_2738474
BD Horizon BV510 Rat Anti-Mouse CD44 (clone IM7)	BD Biosciences	Cat#563114 RRID: AB_2738011
BD Horizon BV605 Rat Anti-Mouse CD19 (clone 1D3)	BD Biosciences	Cat#563148 RRID: AB_2732057
BD Horizon BV605 Rat Anti-Mouse LY-6G (clone 1A8)	BD Biosciences	Cat#563005 RRID: AB_2737946
BD Horizon BV650 Rat Anti-Mouse CD62L (clone MEL-14A)	BD Biosciences	Cat#564108 RRID: AB_2738597
BD Horizon BV711 Rat Anti-Mouse CD279 (PD-1) (clone 29F.1A12)	BD Biosciences	Cat#568563 RRID: Not available
BV786 Rat Anti-Mouse Ki-67 Monoclonal Antibody (clone SolA15)	eBioscience	Cat#417-5698-82 RRID: AB_10854564
BD Horizon Viability stain FVS620	BD Biosciences	Cat#564996 RRID: AB_2869636
BD Pharmingen FITC Mouse Anti-Human HLA-DR (clone TU36)	BD Biosciences	Cat#555560 RRID: AB_395942
BD Pharmingen PE Mouse Anti-Human CD83 (clone HB15e)	BD Biosciences	Cat#550634 RRID: N/A
PerCP/Cyanine5.5 anti-human CD27 Antibody (clone O323)	Biolegend	Cat#302819 RRID: AB_893295
BD Pharmingen™ PE-Cy™7 Mouse Anti-Human CD86 (clone 2331)	BD Biosciences	Cat#561128 RRID: AB_10563077
BD Pharmingen™ APC Mouse Anti-Human CD54 (clone HA58)	BD Biosciences	Cat#561899 RRID: AB_398667
BD™ APC-H7 Mouse Anti-Human CD38 (clone HB7)	BD Biosciences	Cat#656646 RRID:N/A
BD Horizon™ BV421 Mouse Anti-Human IgD (clone IA6-2)	BD Biosciences	Cat#6565940 RRID: AB_11153121
BD Horizon™ BV605 Mouse Anti-Human CD45 (clone HI30)	BD Biosciences	Cat#6564047 RRID: AB_2744403
BD Horizon™ BV786 Mouse Anti-Human CD19 (clone SJ25C1)	BD Biosciences	Cat#563326 RRID: AB_2744314
BD Pharmingen™ PerCP-Cy™5.5 Mouse Anti-Human CD16 (clone 3G8)	BD Biosciences	Cat#560717 RRID: AB_1727434
BD Pharmingen™ APC-H7 Mouse anti-Human CD14 (clone MφP9)	BD Biosciences	Cat#560180 RRID: AB_1645464
BD Horizon™ BV421 Mouse Anti-Human CD11c (clone B-ly6)	BD Biosciences	Cat#562561 RRID: AB_2737656
BD Horizon™ BV510 Mouse Anti-Human CD3 (clone UCHT1)	BD Biosciences	Cat#563109 RRID: AB_2732053
Brilliant Violet 510™ anti-human CD19 Antibody (clone HIB19)	Biolegend	Cat#302242 RRID: AB_2561668
BD Horizon™ BV510 Mouse Anti-Human CD56 (clone NCAM16.2)	BD Biosciences	Cat#563041 RRID: AB_2732786
BD Horizon™ BV711 Mouse Anti-Human CD123 (clone 9F5)	BD Biosciences	Cat#563161 RRID: AB_2738038
Brilliant Violet 785™ anti-human CD141 (Thrombomodulin) Antibody (clone M80)	Biolegend	Cat#344115 RRID: AB_2572195
BD™ CD45RA FITC (clone L48)	BD Biosciences	Cat#335039 RRID: N/A
BD™ CD56 FITC (clone NCAM16.2)	BD Biosciences	Cat#345811 RRID: N/A
BD™ PE Mouse Anti-Human CD38 (clone HB7)	BD Biosciences	Cat#347687 RRID: AB_400341
CD3 Monoclonal Antibody (SK7), PerCP-Cyanine5.5, eBioscience™ (clone SK7)	eBioscience	Cat#45-0036-42 RRID: AB_1518742
BD™ PE-Cy™7 Mouse Anti-Human CD8 (clone SK1)	BD Biosciences	Cat#335787 RRID: N/A
BD Pharmingen™ Alexa Fluor® 647 Mouse anti-Human CD279 (PD-1) (clone EH12.1)	BD Biosciences	Cat#560838 RRID: AB_2033988
APC/Fire™ 750 anti-human CD197 (CCR7) Antibody (clone G043H7)	Biolegend	Cat#353245 RRID: AB_2750146
BD Horizon™ BV421 Mouse Anti-Ki-67 (clone B56)	BD Biosciences	Cat#562899 RRID: AB_2686897
BD Horizon™ V500 Mouse Anti-Human CD4 (clone RPA-T4)	BD Biosciences	Cat#560768 RRID: AB_1937323
BD Horizon™ BV605 Mouse Anti-Human CD45 (clone HI30)	BD Biosciences	Cat#564048 RRID: AB_2744403

**Biological samples**		

Tumor biopsies	OPTIMIZE-1 study (NCT04888312)	Not available due to data protection laws to ensure confidentiality of patient information
Whole blood samples	OPTIMIZE-1 study (NCT04888312)	Not available due to data protection laws to ensure confidentiality of patient information

**Chemicals, peptides, and recombinant proteins**		

FACS ™ lysing solution	BD Biosciences	Cat#349202 RRID: AB_2868862
FACS Permeabilizing Solution 2	BD Biosciences	Cat#340973 RRID: NA

**Critical commercial assays**		

AllPrep DNA/RNA FFPE Kit – Nucleic Acid Extraction	Qiagen	Cat#80234 RRID: NA
Qubit™ dsDNA HS Assay Kit	Invitrogen	Cat#Q32854 RRID: NA
TruSight Oncology 500 DNA Automation Kit	Illumina	Cat#20045504 RRID: NA
Agilent DNA 1000 Kit	Agilent	Cat#5067-1504 RRID: NA
QIAamp Circulating Nucleic Acid Kit	Qiagen	Cat#55114 RRID: NA
QX100/QX200 ddPCR	Bio-Rad	RRID: SCR_019714
Eukaryote Total RNA Nano Series II Assay	Agilent	RRID: NA
KAPA RNA HyperPrep Kit with RiboErase (HMR) Globin	Roche	08308241702 RRID: NA
Pre-validated multiplex MSD cytokine and chemokine assays (proinflammatory panel 1 and chemokine panel 1)	Mesoscale Diagnostics	RRID: NA
Custom-made validated FC panels	Cerba Research	RRID: NA
2 x 100 bp S2 kit flow cell	Illumina	RRID: NA

**Deposited data**		

RNA sequencing data	Gene Expression Omnibus	GEO: GSE291331

**Experimental models: Cell lines**		

KPCY tumor cells (clone 2838c, KRAS^G12D^, Trp53^R172H^)	Kerafast	RRID:CVCL_YM18

**Experimental models: Organisms/strains**		

Human CD40 transgenic mouse	In-house (Alligator Bioscience AB)	N/A

**Software and algorithms**		

QuantaSoft™ Software (Regulatory Edition 1864011)	Bio-Rad	RRID: NA
FACSLyric™	BD Biosciences	RRID: NA
FACSuite™ software (Version 1.4)	BD Biosciences	RRID: NA
Illumina’s BCL Convert (Version 3.10)	Illumina	RRID: NA
bcl2fastq2 Conversion Software (Version 2.20)	Illumina	RRID: SCR_015058
R (Version 4.3.0)	R development core team	RRID: SCR_001905
OncoKB™ data package^34^^,^^35^	Memorial Sloan Kettering Cancer Center	RRID: SCR_014782
TSO500 Local App (Version 2.2)	Illumina	RRID: NA
DRAGEN MIS pipeline (Version TBC)	Illumina	RRID: NA
DRAGEN RNA pipeline (Version 4.0)	Illumina	RRID: NA
Ensembl Variant Effect Predictor (Release TBC)	Ensembl	RRID: SCR_007931
survminer package (Version 0.4.9)	Rdocumentation	RRID: SCR_021094
survival package (Version 3.5–5)	Rdocumentation	RRID: SCR_026244
BaseSpace Sequence Hub	Illumina	RRID: SCR_011881
PCA tools package (Version 2.14.0)	Biooconductor	RRID: SCR_025593
Limma (Version 3.58.1)	Biooconductor	RRID: SCR_010943
ComplexHeatmap package (Version 2.18.0)	Biooconductor	RRID: SCR_017270
DESeq2 (Version 1.42.1)	Biooconductor	RRID: SCR_015687
GO_Biological_Process_2018 database (Version 3.2)	Gene Ontology	RRID: SCR_002811
Immuno-Oncology Biological Research (IOBR) package (Version 0.99.9)	GitHub	RRID: SCR_025619

**Other**		

Covaris E220evolution focused-ultrasonicator	Covaris	500429 RRID: SCR_019817
Agilent 2100 Bioanalyzer	Agilent	RRID: SCR_018043
NovaSeq 6000 instrument	Illumina	RRID: SCR_016387
Qubit®4 Fluorometer	Thermo Fisher Scientific	Cat#Q33226 RRID: SCR_018095

### Experimental models and study participant details

#### Human participants

Eligible patients were aged 18 years or older with a histologically confirmed diagnosis of previously untreated mPDAC, an Eastern Cooperative Oncology Group performance status of 0 or 1 and measurable disease according to modified RECIST version 1.1.^57^ All patients or their representatives provided written informed consent before enrollment. OPTIMIZE-1 is an open-label, phase 1b/2 study being conducted in 14 centers across Europe in accordance with the study protocol and amendments and was approved by the local Institutional Review Boards and independent ethics committees at each of the participating centers. The study is being conducted in accordance with the Declaration of Helsinki and Good Clinical Practice guidelines as defined by the International Conference on Harmonisation.

### Method details

#### Study design

Details of study design, patients and treatments in OPTIMIZE-1 have been reported previously.^16^ The primary outcome of phase 1b was determination of the RP2D of mitazalimab in combination with mFOLFIRINOX by examining the incidence of dose-limiting toxicities (DLTs). The primary outcome of phase 2 was the ORR of mitazalimab in combination with mFOLFIRINOX, defined as the proportion of patients achieving a confirmed CR or PR as per the RECIST v1.1 guideline.^57^ Key secondary objectives applicable to both phases of the study included assessment of efficacy and other clinical outcomes. BoR was defined as the best response (CR, PR, SD, and PD) observed at any time during the study. DoR was defined as the number of days from the initial response (CR or PR) to progressive disease or death due to underlying disease, whichever occurred first, and was assessed in all patients who achieved a confirmed response. Duration of SD was defined as the number of days from first dose of mitazalimab to progressive disease or death, whichever occurred first. DCR was defined as the proportion of patients achieving CR, PR or SD et each visit. Time to next anti-cancer therapy was defined as the number of days from the first dose of mitazalimab to initiation of subsequent treatment. Safety, and tolerability was evaluated by recording the type, frequency. and severity of AEs, which were coded using MedDRA version 24.0, and graded according to the National Cancer Institute Common Terminology Criteria for Adverse Events (version 5.0). Survival outcomes, including PFS and OS, were also assessed.

#### OPTIMIZE-1: Patient inclusion and exclusion

Patients were required to meet several hematological and clinical chemistry laboratory criteria. Key exclusion criteria included other types of non-ductal tumor of the pancreas (including endocrine tumors or acinar cell adenocarcinoma, cyst adenocarcinoma and ampullary carcinoma), other current cancer or history of cancer (other than *in situ* cervical cancer or basal cell or squamous cell carcinoma treated with local excision only) in the three years prior to study enrollment and known central nervous system metastases or carcinomatous meningitis. The complete eligibility criteria, including exclusion criteria specific to mFOLFIRINOX treatment, are provided in Data S1/Methods S1. Patients fulfilling all eligibility criteria were registered onto the study using an Interactive Web Response System automated patient registration system. Patient registration was controlled by accrual limits assigned to the active cohort.

#### OPTIMIZE-1: Patient treatment

In phase 1b, mitazalimab was administered intravenously (IV) during a 2-h rate-controlled infusion at 450 μg/kg and 900 μg/kg, in combination with mFOLFIRINOX (85 mg/m^2^ oxaliplatin IV over two hours, 400 mg/m^2^ leucovorin over two hours, 150 mg/m^2^ irinotecan over 90 min [starting 30 min after the start of the leucovorin infusion] followed by 2400 mg/m^2^ 5-FU over 46–48 h) to determine the RP2D. During the first 21-day treatment cycle, mitazalimab was administered as a priming dose on Study Days 1 (priming dose) and 10 and mFOLFIRINOX infusion was started on Study Day 8. During subsequent 14-day treatment cycles, mFOLFIRINOX was administered on Study Day 1 and mitazalimab on Study Day 3 of each cycle.

Dose escalation in phase 1b followed a Bayesian optimal interval design requiring at least three patients evaluable for a DLT at each dose level. The DLT evaluation period was defined as the time from the first dose of mitazalimab (Study Day 1) until Day 21 in the first treatment cycle (Cycle 1). Detailed descriptions of DLT criteria can be found in Data S1/Methods S1. A minimum of six patients were required to be evaluated at the RP2D. When the last patient had completed the DLT evaluation period in phase 1b, collected data were reviewed by a Data Review Committee, and a decision was made on continuation into phase 2 of the study.

The RP2D of mitazalimab, in combination with mFOLFIRINOX, was administered to all patients in the phase 2 study, with a planned treatment duration of up to 12 cycles. Upon completion of all 12 treatment cycles, at the Investigator’s discretion, a patient deriving clinical benefit (without confirmed PD) was permitted to continue until PD, unacceptable toxicity, withdrawal of consent or until any other treatment discontinuation or study withdrawal criteria are met, whichever came first.

#### OPTIMIZE-1: Efficacy assessments

Anti-tumor activity was evaluated by assessing CT scan of chest/abdomen/pelvis and other body areas, if required, according to local practice with consistent imaging used throughout per patient. Scans were performed according to RECIST v1.1 guidelines at screening and every four cycles thereafter. Additional CT scans were permitted at the Investigator’s discretion.

#### OPTIMIZE-1: Safety assessments

Any AEs considered by Investigators as related to mFOLFIRINOX permitted a dose adjustment according to standard practice for mFOLFIRINOX. The dose of mitazalimab was reduced by 50% where an AE possibly, probably, or definitely related to mitazalimab resulted in more than two weeks of treatment delay. Only one dose reduction was allowed. Any AE considered serious or >Grade 2 and possibly, probably, or definitely related to mitazalimab resulted in mitazalimab being withheld until resolution of the AE to the greater of the baseline grade or ≤ Grade 1, or less. In such cases mitazalimab was permitted to be resumed at the Investigator’s discretion either at the previous dose or, if not already dose-reduced, a 50% dose reduction; the Investigator was also permitted to forgo rechallenge. Study treatment was discontinued if there was evidence of PD, unacceptable toxicity, withdrawal of consent or meeting other criteria for study drug termination.

#### Sample collection and handling

##### Tumor biopsies

Tumor biopsies were collected from patients in the FAS of OPTIMIZE-1 (patients that received 900 μg/kg of mitazalimab for two or more cycles), ITT set (all patients who received 900 μg/kg dose of mitazalimab), and safety set (all patients who received any study treatment, including 450 μg/kg of mitazalimab) at baseline and C2D10 or at another unscheduled time point if not collected at Cycle 2. Samples were collected using an 18-gauge needle (inner diameter = 0.838 mm, inner surface = 0.55 mm^2^). After collection, tumor biopsies underwent formalin fixation followed by paraffin embedding (FFPE) and were either stored as FFPE blocks or FFPE tissue slides.

Subsequently, one slide from each patient was subjected to H&E staining, followed by pathologist evaluation. FFPE samples that had a tumor cell content below 20%, or detectable necrotic or healthy adjacent tissue, were subjected to macrodissection to enrich for tumor cell areas ensuring tumor purity during downstream procedures and analyses. No macrodissection was performed when tumor cell islets were dispersed, or the tumor area was less than 5mm^2^.

##### Whole blood samples

Whole blood samples were collected longitudinally according to the schedule of assessments) in the OPTIMIZE-1 study protocol (Data S1/Methods S1). Streck tubes were used for ct*KRAS* subtype analysis and tumor burden assessment, while ethylenediamine tetraacetic acid (EDTA) tubes were used for immunophenotyping.

#### Genomic profiling of tumor biopsies

##### DNA extraction, library preparation and sequencing

Total DNA was extracted from FFPE slides using the AllPrep DNA/RNA FFPE Kit (50) (Qiagen, Hilden, Germany, Cat# 80234). Extracted DNA was quantified with the Qubit dsDNA HS assay (Invitrogen, Massachusetts, USA, Cat# Q32854). DNA samples meeting the input requirement (manufacturer-recommended input of 40ng, with very low DNA yield adjusted to 14 ng) were fragmented with the Covaris E220evolution focused-ultrasonicator (Covaris, Massachusetts, USA, Cat#500429, RRID: SCR_019817). Mechanical fragmentation was followed by 3′ and 5′ end repair. Samples were then used as templates for cDNA library generation using the TruSight Oncology 500 (TSO500) High-Throughput DNA Library Preparation Kit (Illumina, California, USA, Cat# 20045504). Library concentration was determined using the Qubit dsDNA HS Assay Kit; size distribution and average fragment length were evaluated using the Agilent DNA 1000 assay (Agilent, California, USA, Cat# 5067-1504) on the Agilent 2100 Bioanalyzer (Agilent, RRID: SCR_018043). Libraries were sequenced on a NovaSeq 6000 instrument using the 2 x 100 bp S2 kit flow cell (Illumina) to achieve a mean target coverage of ≥150x.

##### Sample filtering of TSO500 dataset

The TSO500 dataset derivation can be found in Figure S2. Following QC, 37 samples were retained for demographic analysis. From these, 35 baseline samples with RECIST response data were used for associations with RECIST response. Finally, 32 baseline samples from the FAS with survival data were tested for association with clinical variables. Three patients with unscheduled visits according to eCRF records were recategorized by comparing the first mitazalimab dose date and the sample collection date. Details of these analyses can be found in the quantification and statistical analysis section.

#### Transcriptome profiling of tumor biopsies

##### RNA extraction, library preparation and sequencing

Total RNA was extracted from FFPE slides using the AllPrep DNA/RNA FFPE Kit (50). RNA QC was performed via electrophoresis using Eukaryote Total RNA Nano Series II assay in a 2100 Bioanalyzer instrument. RNA samples meeting the size range requirements (40 ng input RNA) were subjected to library preparation using the KAPA RNA HyperPrep Kit with RiboErase (HMR) Globin (Roche, Basel, Switzerland, Cat# 08308241702). Derived cDNA library concentration was determined using the Qubit dsDNA HS Assay Kit; size distribution and average fragment length, and RIN value were evaluated on Agilent 2100 bioanalyzer. cDNA libraries were divided into two batches based on a 240bp library size threshold. Libraries were sequenced in a NovaSeq 6000 instrument using NovaSeq 6000 S2 (Batch 1) and S1 (Batch 2) flow cells at the desired sequencing depth (50 M reads per library, 2 X 100 bp paired end reads). Three samples were sequenced in both batches to ensure proper batch normalization in the event that sequencing batch contributed to variance in the dataset.

##### Sample filtering of RNA-seq dataset

As for the TSO500 dataset derivation, the 40 unique sequenced samples, the remaining 40 samples were subjected to gene and sample filtering (see Figure S3). Gene counts >10 in 20 samples were considered detected, and four samples with <15000 (<75%) detected genes were excluded from analysis, yielding 36 QC’d samples (Figure S3). Of note, the four excluded samples featured either low library concentration or low insert fragment size. Three patients with unscheduled visits according to eCRF records were recategorized by comparing the first mitazalimab dose date and the sample collection date.

##### Circulating KRAS subtype analysis from blood samples

The ct*KRAS* G12 subtype analysis on blood samples was performed by Saga Diagnostics (Lund, Sweden), developed to detect the three most frequent G12 alleles in PDAC; *KRAS* G12V/D/R variants. DNA was extracted from plasma from whole blood using the QIAamp Circulating Nucleic Acid Kit (Qiagen, Cat# 5511). Following extraction, DNA concentrations were measured using a Qubit 4 Fluorometer (Thermo Fisher Scientific, Massachusetts, USA, Cat# Q33226, RRID: SCR_018095) and results were generated using the QX100/QX200 ddPCR (Bio-Rad Laboratories, Veenendaal, the Netherlands, RRID: SCR_019714), and analyzed using the QuantaSoft Software that accompanies the QX100/QX200 systems.

A clinical sample was considered *KRAS* G12V/D/R mutant positive if the measured MAF was higher than the determined limit of blank of the assay and if there were at least two mutant positive droplets across the test reactions. Sample results that fail either criterion may still be called mutant positive on a case-by-case basis with strong supporting evidence and expert interpretation.

##### Cytokine and chemokine assessment

Cytokine and chemokine levels in serum were analyzed at Cerba Research using Mesoscale Discovery (MSD; Mesoscale Diagnostics, Maryland, USA) and pre-validated multiplex MSD cytokine and chemokine assays (proinflammatory panel 1 and chemokine panel 1) which included the following cytokines and chemokines: IL-8, IL-10, IL-12p70, TNF-α, IFN-γ, MCP-1, IP-10, MIP-1 alpha and MIP-1 beta.

##### Immunophenotyping assessment

Immunophenotyping and activation status were analyzed in whole blood samples using custom-made validated flow cytometry panels at Cerba Research. Details of the immunophenotypes in both the APC and T/NK cell panels can be found in Table S13.

Sample preparation and FC analysis (using custom-made validated FC panels at Cerba Research) were conducted according to validated assay protocols within 72 h after collection of whole blood samples. All incubation steps were carried out at room temperature and in the dark. Whole blood was lysed with FACS lysing solution (BD Biosciences, Cat# 349202, RRID: AB_2868862). For stainings, 100 μL of anti-coagulated whole blood was transferred to each assay tube and incubated with the appropriate antibody cocktail mixture. For intracellular stainings, after the surface staining procedure samples were centrifuged, the supernatant was discarded, and the cells were resuspended in the FACS Permeabilizing Solution 2 (BD Biosciences, Cat# 340973). Samples were then incubated with the intracellular staining antibody cocktail. After washing and resuspension all samples were analyzed within 1 h after sample preparation. Data acquisition was done using FACSLyric (BD Biosciences, New Jersey, USA). Analysis of the raw data was performed with FACSuite software, version 1.4.

### Quantification and statistical analysis

#### Clinical data analysis

The study employed a Simon’s two stage design to statistically evaluate whether the addition of mitazalimab to mFOLFIRINOX improved the ORR compared to a historical control rate of 30% (ORR with chemotherapy alone). The formal hypotheses were.

(1) Null hypothesis (H_0_): ORR ≤30% (no improvement over historical control)
(2) Alternative hypothesis (H_1_): ORR >30% (superior to historical control)

For sample size estimation, a target ORR of 45% was assumed to reflect the anticipated improvement with mitazalimab. The study was powered at 80% with a one-side type I error rate of 5%. The safety set (*n* = 70) includes all patients who received any dose of mitazalimab. The ITT set (*n* = 65) includes all patients who received a dose of 900 μg/kg mitazalimab. The FAS (*n* = 57) is all patients who received the combination of mitazalimab at the RP2D (900 μg/kg) and mFOLFIRINOX for at least two treatment cycles.

Full details of the statistical analyses of the clinical data have been reported previously and are described in Data S1/Methods S1.

Median DoR, PFS, and OS were estimated using Kaplan-Meier methods with 95% CIs. ORR is accompanied by one-sided 90% CIs. No formal statistical hypothesis was defined for phase 1b; patients were pooled with those on the same dose regimen in phase 2 for statistical analyses and data summaries.

#### Analysis of genomic profiling of tumor biopsies

##### TSO500 data analysis

Raw sequencing data (binary base call [BCL] files) were stored in BaseSpace Sequence Hub, demultiplexed, and converted to FASTQ files using bcl2fastq2 Conversion Software (Illumina, RRID: SCR_015058). The FASTQ files were transferred to the TruSight Oncology 500 Local App (Illumina) for analysis. The sequences in the FASTQ files were aligned to the GRCh37 (hg19) reference genome using the Isaac Aligner, generating Binary Alignment Map (BAM) files. BAM files were then processed by the TSO500 Local App’s DNA Analysis Methods. Specifically, copy number variant (CNV) calling utilized the CRAFT algorithm, and structural variant detection employed the Manta algorithm. TMB was calculated by counting all coding SNVs and indels with variant allele frequency ≥5% and variant allele count ≥3. MSI status was determined using 130 microsatellite loci. All analyses were performed using default parameters as recommended by Illumina for the TSO500 assay.

##### Demographic analyses

Data analysis and visualization were performed in R (version 4.3.0, RRID: SCR_001905), running in a virtual environment from docker. Genes were classified into oncogene or tumor suppressor gene based on their annotation using the “oncoKBdata” package (https://github.com/waldronlab/oncoKBData).^34^^,^^35^ The frequency of the top 25 altered genes was compared to current literature.^23^^,^^24^^,^^31^^,^^32^^,^^33^

##### Small variant mutation

The small variant mutation dataset was generated from single combined variant output files (per sample). Individual variants were annotated using the Ensembl Variant Effect Predictor software to gain further information on the variants such as predicted functional effects and allele frequency in various populations. To remove potential germline mutations, the data were subsequently filtered to exclude variants with MAF >0.01 in any of the 1000 Genomes, exome sequencing project (ESP) or Genome Aggregation Database (gnomAD) populations. Finally, only somatic variants with strong functional consequences (Ensembl IMPACT rating [Ensembl IMPACT rating] of moderate or high) were selected. In contrast, analysis of DNA damage repair genes *BRCA1*, *BRCA2* and *PALB2* was performed by excluding variants with MAF <0.01 in any of the 1000 Genomes, ESP or gnomAD populations. Finally, only germline variants with strong functional consequences (Ensembl IMPACT rating high) were selected.

##### Copy number variation

CNV data, where copy number losses and gains were identified, were extracted from the CNV call format (VCF) files. Only CNVs that passed the quality assessment (FILTER = PASS) were used in the statistical analyses. Only copy number gains were retained.

##### Tumor mutational burden

Samples were classified as having a high or low TMB based on their median TMB value (2.65 mutations/Mb). No distinction was performed between synonymous and non-synonymous mutations.

##### Microsatellite instability status

MSI status data were obtained from the TSO500 Local App’s pipeline. The percentage of unstable MSI sites to total assessed MSI sites is reported as a sample-level microsatellite score. Samples were categorized into MS-stable (MSS; <20%) and MSI (≥20%) based on the DRAGEN pipeline recommendations (Microsatellite Instability [illumina.com]) for solid samples. All samples with usable MSI sites were MS-stable; and thus, no statistical analysis could be performed based on microsatellite status.

##### Associations of genomic alterations in tumor biopsies with clinical variables

Associations of genomic alterations to clinical variables were performed by FIOS genomics (Edinburgh, UK).

##### Association with categorical clinical variables (OS rate at 12 months, PFS rate at 12 months, ORR, DC)

The presence of a specific variant, gene mutation (as a binary variable), and a gene CNV (as a binary variable) were assessed for association with OS-12, PFS-12, ORR and disease control using Fisher’s exact test. OS-12 segregated patients into still alive after 12 months (post-12 months) or had died before or at 12 months (pre-12 months). PFS-12 segregated the patients into progression-free after 12 months (post-12 months) or showed PD before or at 12 months (pre-12 months). TMB status was assessed for association with OS-12, PFS-12, ORR and disease control using a *t*-test.

##### Association with continuous clinical variables (overall survival, progression-free survival, duration of response)

The presence of a specific variant, gene mutation (as a binary variable), and a gene CNV (as a binary variable) were assessed for association with each of the three survival variables, OS (‘SURV 0S’), PFS (‘SURV PFS’) and duration of confirmed response (‘SURV CRSD’) using univariate Cox PH regression. Genes altered in at least three samples were included in the statistical analyses.

TMB status was assessed for association with OS (‘SURV 0S’), PFS (‘SURV PFS’) and duration of confirmed response (‘SURV CRSD’) using univariate Cox PH regression.

##### Association of circulating KRAS with continuous clinical variables

OS and PFS differences between ct*KRAS* G12 subtypes were tested using both the log rank and Gehan-Breslow tests.

#### Correlation between circulating KRAS and tumor volume

Data analysis and visualization were performed in-house using R (version 4.3.0). Raw data from the ct*KRAS* subtype analysis assay was retrieved. The %MAF was defined as the fraction of *KRAS* G12V/D/R concentration divided by the total concentration, expressed as a percentage.

*KRAS* G12 V/D/R % MAF information from blood ctDNA was used in the analyses. The three *KRAS* G12 Wt patients, and the eight *KRAS* undetermined patients were filtered out for subsequent analyses. The %MAF was standardized on a per patient basis, using longitudinal on-treatment %MAF divided by the %MAF at baseline. Similarly, tumor volume as indicated by SLD was standardized on a per patient basis, using longitudinal on-treatment SLD divided by the SLD at baseline, expressed as a percentage. Standardized and %SLD was matched to the closest %MAF measure for each patient (10.67 days difference on average ±6.03 days). Correlation between %MAF and %SLD was visualized using a scatterplot and statistically tested using Pearson’s correlation test. Standardized data from patients with three or more datapoints were used to generate patient-specific exponential models. These models were used to calculate appropriate mResponse and mProgression thresholds, equivalent to those used in RECIST based on conventional CT scan tumor measures. The thresholds were further validated using the ”surv_cutpoint” function of the survminer package (version 0.4.9., RRID: SCR_021094).

##### Association of circulating KRAS, circulating KRAS clearance, mResponse, mProgression with clinical efficacy endpoints

Patients carrying a *KRAS* G12 V/D/R mutation with available baseline ct*KRAS* data were included in the associations between ct*KRAS* baseline levels and clinical efficacy endpoints. From this set, only patients with two or more on-treatment ct*KRAS* datapoints, including at baseline and at least one on-treatment datapoint, were included in the associations between ct*KRAS* clearance, mResponse, or mProgression, and clinical efficacy endpoints. The threshold for ct*KRAS* clearance was defined as the mean of MAF from *KRAS* G12 Wt patients plus three standard deviations. To define mResponse, reductions in %MAF from baseline were analyzed for correlation with clinical response. Previously described exponential models were used to estimate a %MAF threshold equivalent to the conventional 30% reduction in %SLD, as defined by RECIST v.1.1^57^ (Figure 5B). A reduction of 30% in SLD translated into a reduction of 82.5% (±21.0) in %MAF. Thus, mResponse was defined as the reduction of at least 90% from baseline in ct*KRAS* % MAF within the first 70 days of treatment.

Survival was visualized using the KM method and reported as median with 95% CI. OS between groups was compared using a log rank test using the survival package (version 3.5–5, RRID: SCR_026244). Assessment of the impact of continuous variables on survival parameters was performed via cox PH using the survival package.

#### Analysis of transcriptome profiling of tumor biopsies

##### RNA-seq data analysis

Raw sequencing data (BCL files) were stored in BaseSpace, demultiplexed and converted to FASTQ files (Illumina’s BCL Convert version 3.10.10). The FASTQ files were transferred to BaseSpace Sequence Hub (Illumina, RRID: SCR_011881) for further analysis. After aligning FASTQ files to the GRCh37 (hg19) reference genome, quantification of reference genes and transcripts were performed using the DRAGEN RNA pipeline (Illumina version 4.0).

Data analysis and visualization were performed in R (version 4.3.0).

##### Confounding factor analysis for RNA seq

RNA-seq was performed in two batches and a batch-effect analysis was performed. Clinical and technical covariates in metadata were evaluated in confounding factor analysis. Pairs of numerical variables were evaluated by Pearson correlation. Pairs of categorical covariates were evaluated by either Chi-square or Fisher’s exact test (depending on number of samples per group). Pairs of categorical and numerical covariates were evaluated using analysis of variance (ANOVA). P-value was adjusted using the false discovery rate (FDR) method (see Figure S8A). Contribution of clinical and technical covariates to PC variance was performed using the “eigencorplot” function from the PCA tools package (version 2.14.0) (Figure S8B).

Given that sequencing batch did not contribute to variance in the first 10 PCs, the three re-sequenced samples were addressed. Thus, for samples that were sequenced twice, batch 1 counterparts were removed, while batch 2 counterparts were kept (Figure S3). Technical covariates that were significantly associated with the first two PCs and not confounded with clinical efficacy endpoints (namely “hospital”, “Biopsy_site”, “Macrodissection”, “Sample type”, “HE_AREA”) were tested for regression using ”removebatcheffect” function in Limma (version 3.58.1, Bioconductor, RRID: SCR_010943).^58^ Using VST counts, the regression of “Biopsy_site” and “Hospital” removed the effect of the technical covariates while maximizing the associations of clinical efficacy endpoints to the first 10 PCs (Figure S8B).

##### Molecular subtype classification (classical vs. basal-like)

Thirty-three (33) unique QC’d samples (from 31 patients; Figure S3) were subjected to molecular classification into classical and basal-like subtypes using previously published gene-sets.^20^^,^^21^ Briefly, VST counts of genes in each gene set were subjected to *Z* score transformation and PCA visualization. Next, samples were clustered via K-means clustering in the PCA space and visualized in heatmap format using the ComplexHeatmap package (version 2.18.0, Bioconductor, RRID: SCR_017270).^59^

##### Differential gene expression and pathway enrichment analyses

Exploratory data analysis using clinical efficacy endpoints was performed only in the FAS, which includes samples from the efficacy cohort (*n* = 27, including 21 baseline samples and six on-treatment samples). Filtered gene counts were subjected to normalization (via median of ratios method) and DGEA (accounting for batch effects in covariates Hospital and Biopsy_site) using DESeq2 (version 1.42.1, Bioconductor, RRID: SCR_015687) with the normal shrinkage estimator. P-values were adjusted using FDR (BH correction). DEGs were visualized using “EnhancedVolcano“ package (version 1.20.0; https://github.com/kevinblighe/EnhancedVolcano, Bioconductor, RRID: SCR_018931). DEGs, defined by *p*-value <0.05 and absolute log2 FC >1, were extracted, and used for pathway enrichment analysis employing the enrichR package to query the GO_Biological_Process_2018 database (version 3.2, RRID: SCR_002811).^60^ Pathways were considered enriched if their adjusted *p*-value threshold was <0.05.

##### Gene set scoring

Filtered transcripts per million (TPM) counts were normalized via a linear model for microarray data (LIMMA) package using the clinical variable under study, and accounting for the batch effects of “Hospital” and “Biopsy_site” covariates. Normalized TPM counts were then used for TME deconvolution using the function “deconvo_tme” from the Immuno-Oncology Biological Research (IOBR) package (version 0.99.9, GitHub, RRID: SCR_025619), either using the MCP-counter or EPIC methods. Deconvolved cell scores were then statistically compared between groups. First, distribution of the data was evaluated by Shapiro-Wilk test, and differences between groups were tested using either t-test or Wilcoxon rank-sum test depending on normality. Gene signatures were evaluated via the “calculate_sig_score” function in the IOBR package using the ssGSEA method. In both cases, *p*-values were adjusted using the BH adjustment.

#### Data analysis of immune cells and cytokines

Filtered FC data and cytokine data were used for data analysis of immune cells and cytokines. Levels of the main cell populations depicted in this report, along with all cytokines except IL-12p70, were selected for further analysis, inclusive of C1D1 through C2D1. For a given cell population or cytokine, levels at a given timepoint were compared to the rest of timepoints, through either an unpaired t-test or unpaired Mann Whitney test, depending on the distribution of the data. Multiple comparisons were adjusted using the FDR method.

#### Additional resources

This study is registered with ClinicalTrials.gov (NCT04888312).

## Supplemental information

Document S1. Figures S1–S8, Tables S1–S13, Data S1, and Methods S1

Document S2. Article plus supplemental information
