# Supplementary material for: CD40 agonist mitazalimab with mFOLFIRINOX in untreated metastatic pancreatic cancer: Biomarkers associated with outcomes from OPTIMIZE-1
Source: Cell Rep Med. 2025 Oct 7;6(10):102407. doi: 10.1016/j.xcrm.2025.102407 (PMC12629792; doi:10.1016/j.xcrm.2025.102407)
Supplement: Document S2. Article plus supplemental information [file mmc2.pdf]

Cell Reports Medicine

Article

CD40 agonist mitazalimab with mFOLFIRINOX in untreated metastatic pancreatic cancer: Biomarkers associated with outcomes from OPTIMIZE-1

Graphical abstract

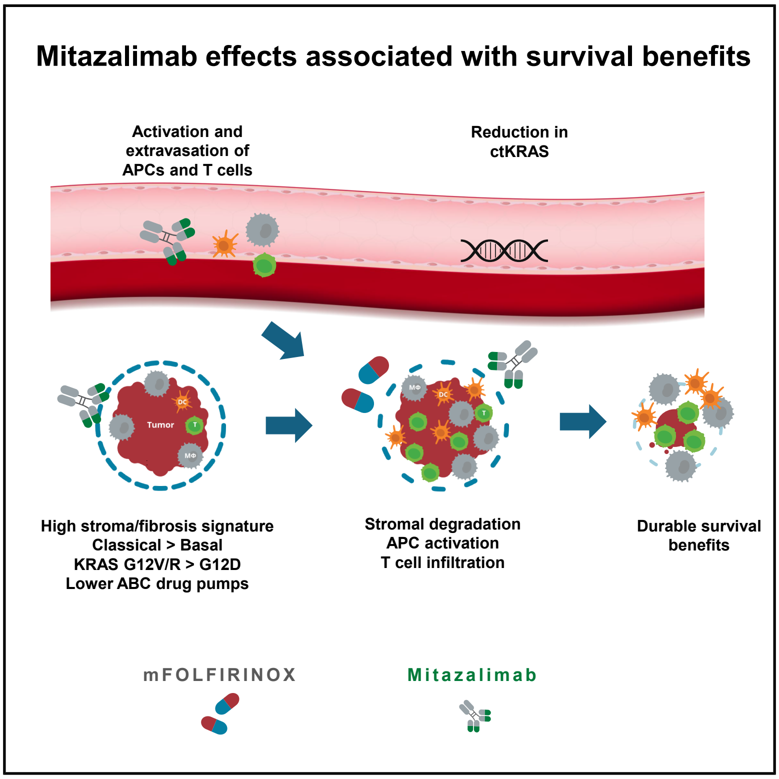

Highlights

- Encouraging activity: ORR 42.1%, DoR 12.6 months, OS 14.9 months, and 18-month OS 36.2%
- A baseline fibrosis gene-signature associates with OS
- Mitazalimab induces intratumoral myeloid and T cell activation in objective responders
- Mitazalimab-induced immune activation correlates with better outcomes

Authors

Jean-Luc Van Laethem, Karen Geboes, Ivan Borbath, ..., Sumeet Vijay Ambarkhane, Gregory L. Beatty, Eileen M. O'Reilly

Correspondence

pek@alligatorbioscience.com (P.E.), gregory.beatty@pennmedicine.upenn.edu (G.L.B.), oreillye@mskcc.org (E.M.O.)

In brief

Van Laethem et al. report that the combination of mFOLFIRINOX and mitazalimab has manageable safety and encouraging activity. Mitazalimab-induced immune activation and a fibrosis gene signature correlate with better outcomes. These results may inform future patient stratification strategies supporting a planned randomized confirmatory trial in mPDAC.

Van Laethem et al., 2025, Cell Reports Medicine 6, 102407
October 21, 2025 © 2025 The Author(s). Published by Elsevier Inc.
https://doi.org/10.1016/j.xcrm.2025.102407

CellPress

Cell Reports Medicine

CellPress
OPEN ACCESS

Article

CD40 agonist mitazalimab with mFOLFIRINOX in untreated metastatic pancreatic cancer: Biomarkers associated with outcomes from OPTIMIZE-1

Jean-Luc Van Laethem,[1] Karen Geboes,[2] Ivan Borbath,[3] Teresa Macarulla Mercade,[4] Aurélien Lambert,[5] Philippe Cassier,[6] Hans Prenen,[7] Emmanuel Mitry,[8] Jean-Frédéric Blanc,[9] Lorenzo Pilla,[10] Jaime Feliu,[11,22,23] Mercedes Rodriguez Garrote,[12] Roberto Antonio Pazo-Cid,[13] Inmaculada Gallego,[14] Karin Enell Smith,[15] Karin Nordbladh,[16] David Gomez Jimenez,[15] Peter Ellmark,[17,18,25,*] Yago Pico de Coaña,[19] Sumeet Vijay Ambarkhane,[19] Gregory L. Beatty,[20,24,*] and Eileen M. O'Reilly[21,24,*]

[1]Gastroenterology, Hepatology and Digestive Oncology, Hopital Universitaire Bruxelles. Erasme Universite Libre de Bruxelles, Brussels, Belgium
[2]Gastroenterology Department, UZ Gent - University Hospital Ghent, Gent, Belgium
[3]Hepatogastroenterology, Cliniques Universitaires Saint-Luc (UCLouvain Saint-Luc), Woluwe-Saint-Lambert, Belgium
[4]Medical Oncology Department, Vall d'Hebron Institute of Oncology, Barcelona, Spain
[5]Institut de Cancérologie de Lorraine, Université de Lorraine, Nancy, France
[6]Medical Oncology Department, Centre Léon Bérard, Lyon, France
[7]Oncology Department, UZA - University Hospital Antwerp, Edegem, Belgium
[8]Medical Oncology Department, IPC - Institut Paoli-Calmettes, Marseille, Cedex, France
[9]Hepatology, Gastroenterology and Digestive Oncology, CHU Bordeaux - Hopital Haut-Lévêque, Bordeaux, France
[10]Department of Hepato-Gastroenterology and Gastrointestinal Oncology, Georges Pompidou European Hospital, Paris, France
[11]Department of Medical Oncology, Hospital Universitario La Paz, Madrid, Spain
[12]Medical Oncology, Hospital Universitario Ramon y Cajal, Madrid, Spain
[13]Medical Oncology Department, HospItal Universitario Miguel Servet, Zaragoza, Spain
[14]Medical Oncology, Hospital Universitario Virgen del Rocio, Seville, Spain
[15]Non-clinical, Alligator Bioscience AB, Lund, Sweden
[16]Clinical Operations, Alligator Bioscience AB, Lund, Sweden
[17]Scientific, Alligator Bioscience AB, Lund, Sweden
[18]Department of Immunotechnology, Lund University, Lund, Sweden
[19]Medical, Alligator Bioscience AB, Lund, Sweden
[20]Division of Hematology/Oncology, Perelman School of Medicine, University of Pennsylvania, Philadelphia, PA, USA
[21]Memorial Sloan Kettering Cancer Center, New York, NY, USA
[22]Instituto de Investigación Sanitaria del Hospital Universitario La Paz (IdiPAZ), Madrid, Spain
[23]Centro de Investigación Biomédica en Red de Cáncer (CIBERONC), Madrid, Spain
[24]These authors contributed equally
[25]Lead contact
*Correspondence: pek@alligatorbioscience.com (P.E.), gregory.beatty@pennmedicine.upenn.edu (G.L.B.), oreillye@mskcc.org (E.M.O.)
https://doi.org/10.1016/j.xcrm.2025.102407

SUMMARY

Response determinants to immunotherapy in metastatic pancreatic ductal adenocarcinoma (mPDAC) remain unclear, limiting treatment advancements. We report a single-arm phase 1b/2 study (OPTIMIZE-1) evaluating the safety and efficacy of the cluster of differentiation 40 (CD40) agonist mitazalimab combined with modified FOLFIRINOX (mFOLFIRINOX), in chemotherapy-naive patients with mPDAC. Patients receive an initial dose of mitazalimab one week before starting biweekly cycles of mFOLFIRINOX plus mitazalimab. The study meets its pre-specified primary endpoint, achieving a confirmed objective response rate (ORR) of 42.1%. Median duration of response, progression-free survival, and overall survival was 12.6 months, 7.7 months, and 14.9 months, respectively. Multi-omic analyses of tumor and blood specimens identify a baseline tumor-intrinsic gene signature related to fibrosis associated with improved survival. Additionally, mitazalimab-induced increases in activated circulating myeloid, B cell, and T cell frequencies correlate with better outcomes. These results may inform future patient stratification strategies supporting a planned randomized confirmatory trial of mitazalimab with mFOLFIRINOX in mPDAC. This study was registered at ClinicalTrials.gov (NCT04888312).

© 2025 The Author(s). Published by Elsevier Inc.
This is an open access article under the CC BY license (http://creativecommons.org/licenses/by/4.0/).

INTRODUCTION

Pancreatic ductal adenocarcinoma (PDAC) is a highly aggressive malignancy with a dismal prognosis and is projected to become the second leading cause of cancer-related deaths by 2030.[1] Metastatic PDAC (mPDAC) is particularly lethal, with a 5-year survival rate of only 3%. Current standard of care regimens, including gemcitabine plus nab-paclitaxel and FOLFIRINOX, achieve objective response rates (ORRs) of 20%–40% and a median progression-free survival (PFS) of approximately 6 months[2] The recently approved NALIRIFOX regimen provides similar outcomes.[3]

Treatment resistance in mPDAC is largely attributed to its unique tumor microenvironment (TME), characterized by a desmoplastic reaction and a dense fibrosis. This microenvironment is frequently associated with the infiltration of suppressive myeloid cells, exclusion of cytotoxic T cells, poor vascularity, and high interstitial pressures, all of which limit drug delivery and contribute to the immunosuppressive nature of the disease.[4] Consequently, immunotherapies such as immune checkpoint inhibitors have demonstrated limited efficacy in mPDAC.[5,6]

Despite these challenges, the TME of mPDAC is plastic and has the potential to be reprogrammed for anti-tumor effects. Notably, patients with mPDAC exhibiting increased T cell infiltration into tumors have a more favorable prognosis,[7–9] suggesting the potential of T cell-directed immunotherapies. One promising approach involves targeting cluster of differentiation 40 (CD40), a member of the tumor necrosis factor receptor superfamily.[10] CD40 is expressed on the surface of B cells, monocytes, macrophages and dendritic cells (DCs) and plays a key role in bridging innate and adaptive immunity by "licensing" DCs to prime and activate T cells, thereby triggering tumor-specific T cell immunity. Preclinical models have demonstrated the potential of CD40 agonists to reverse T cell exclusion in PDAC,[11] and clinical data have revealed similar biology.[12] Recent studies investigating the combination of CD40 agonists with gemcitabine and nab-paclitaxel in PDAC have shown that tumor extrinsic immune signatures associate with improved outcomes,[13,14] consistent with the potential benefit of CD40 agonism.

Preclinical models show that CD40 agonists can remodel the TME to enhance the efficacy of chemotherapy,[15] and clinical data support this remodeling of the fibrotic reaction by CD40 agonists in PDAC.[12] Thus, the sequencing of CD40 agonist administration and chemotherapy may be critical to treatment efficacy.[10] We recently reported results from the OPTIMIZE-1 study, which investigated the combination of modified FOLFIRINOX (mFOLFIRINOX) with mitazalimab, a second-generation CD40 agonist, as first-line treatment for patients with mPDAC.[16] In this study, mitazalimab was administered first as a conditioning regimen to remodel the tumor and sensitize it to chemotherapy. This treatment sequencing approach resulted in deep responses, an increase in duration of response (DoR), and prolonged overall survival (OS). Importantly, the safety profile was consistent with mFOLFIRINOX, with no signs of toxicity related to the addition of mitazalimab.

A significant challenge in developing effective immunotherapies for mPDAC is the identification of patients most likely to benefit from treatment. Currently, there are no validated prognostic biomarkers to predict benefit from mFOLFIRINOX, although *KRAS* G12 subtype and tumor molecular classification into classical and basal-like subtypes have been used to genotype tumors.[17–24] Circulating tumor DNA (ctDNA) has also emerged as a promising longitudinal prognostic marker for patients with solid tumors.[25–28] In this study, we report updated clinical results and investigate determinants of treatment efficacy of mitazalimab combined with mFOLFIRINOX. Our findings reveal that a baseline tumor-intrinsic gene signature comprising transcripts related to fibrosis associates with improved survival. Further, increases in frequencies of activated myeloid, B, and T cells following mitazalimab administration correlate with improved outcomes, supporting mitazalimab's contribution to anti-tumor activity. Additional factors associated with better OS included ctDNA clearance and classical PDAC subtype. Collectively, our work defines potential patient subsets that derive the greatest benefit from this combination therapy and supports the use of mitazalimab as a therapeutic agent to enhance the efficacy of mFOLFIRINOX chemotherapy.

RESULTS

Clinical study data analysis: Study design, patient characteristics, and patient treatment

This phase 1b/2 study was designed as a single-arm study to assess the safety and preliminary efficacy of mitazalimab in combination with mFOLFIRINOX in patients with treatment-naive mPDAC. Patients received mitazalimab on day 1 (priming dose), followed by a 2-week dosing regimen starting with mFOLFIRINOX on day 8 and mitazalimab on day 10. Treatment was administered until disease progression (PD), unacceptable toxicities, or consent withdrawal, whichever came first. The phase 2 primary objective was to assess the clinical activity of this combination therapy using ORR as the primary endpoint. Between September 29, 2021, and March 28, 2023, 88 chemotherapy-naive patients with mPDAC were screened for study eligibility. Of these, 70 patients fulfilled the eligibility criteria and received study treatment (see Figures S1A and S1B). Baseline demographics and clinical characteristics of these 70 treated patients are presented in Table S1. In the phase 1b dose-escalation portion, five patients received 450 μg/kg mitazalimab and six patients received 900 μg/kg. In the phase 2 portion, all patients received mitazalimab at the recommended phase 2 dose (RP2D) of 900 μg/kg mitazalimab (Figure S1A).

For the efficacy analysis, only patients who received mitazalimab at the RP2D (900 μg/kg) and had completed at least two treatment cycles at the RP2D were included in the full analysis set (FAS), regardless of whether they were enrolled in phase 1b or phase 2. Thus, the FAS comprised 57 patients: six from phase 1b (received mitazalimab at 900 μg/kg) and 51 from phase 2. The five patients from phase 1b who received the lower dose (450 μg/kg) were excluded from the efficacy analysis, as were any patients who discontinued before completing two cycles at the RP2D to ensure that efficacy outcomes reflect the activity of the dose selected for further development. In total, 65 patients were treated with at least one dose of mitazalimab, Figure S1C

CellPress
OPEN ACCESS

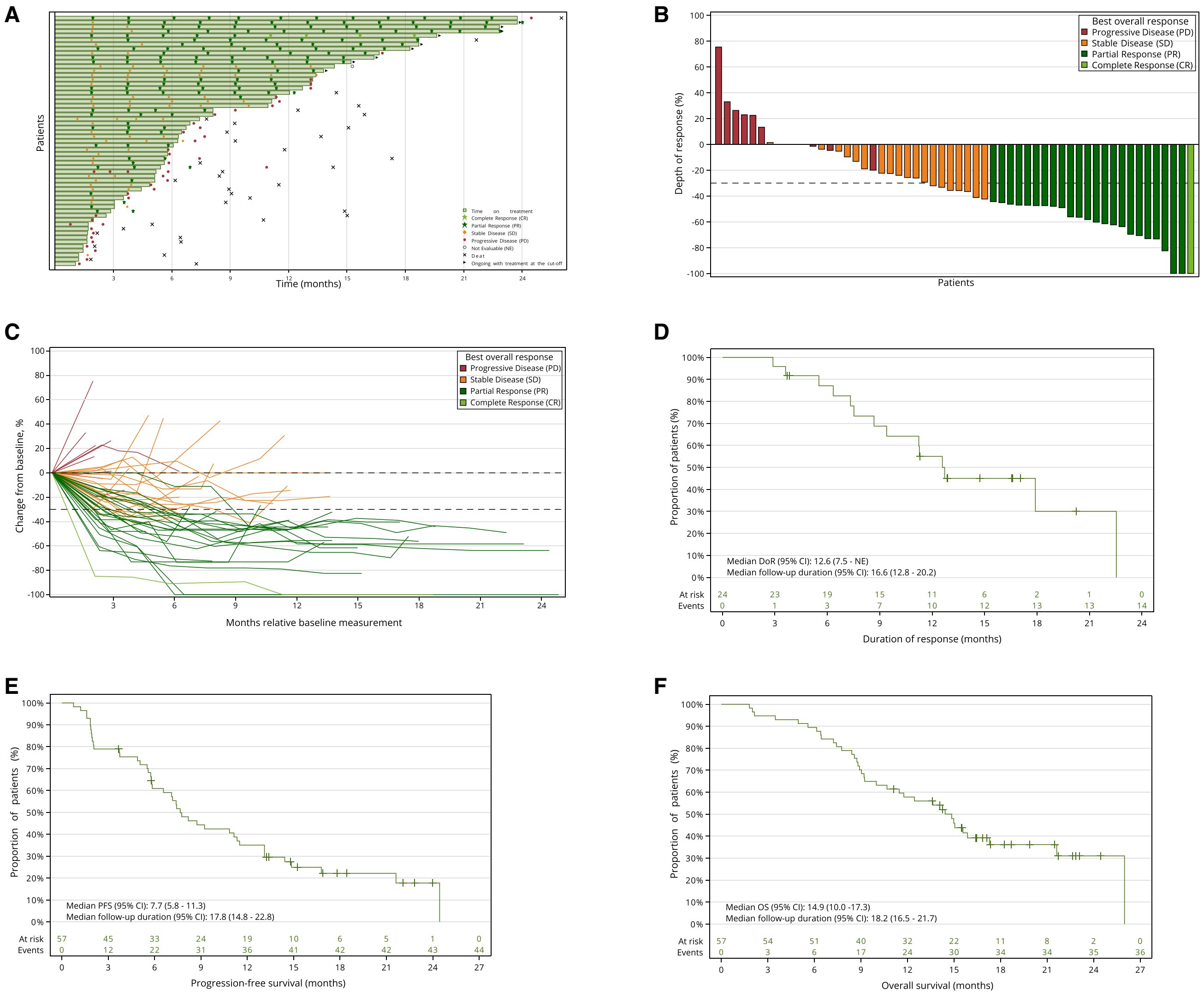

Figure 1. Clinical outcomes in the OPTIMIZE-1 trial

(A) Swimmer plot showing the treatment durations for all 57 patients in the full analysis set.
(B) Waterfall plot showing the maximum percentage change in the tumor size relative to baseline target lesions of individual patients by best overall response.
(C) Spider plot showing percent change from baseline in summed tumor target lesions over time.
(D) KaplanMeier (KM) curve estimates for duration of response (DoR).
(E) KM curve estimates for progression-free survival (PFS).
(F) KM curve estimates for overall survival (OS).
CI, confidence interval.

lists the 8 patients who discontinued before Cycle 2 along with the reasons for exclusion.

Clinical study data analysis: Primary and secondary efficacy endpoints

We present an updated efficacy analysis based on data from 57 efficacy-evaluable patients (FAS) treated with 900 μg/kg mitazalimab (data cut-off May 16, 2024, median follow-up duration of 18.2 months [95% confidence interval (CI): 16.5–21.7]). At the time of data cut-off, 19 patients (33.3%) remained in the study, with nine (15.8%) still receiving treatment (Figure 1A). Forty-eight patients discontinued treatment, 39 due to PD. Of these, 32 patients (56.1%, of the 57 evaluable patients) received second-line systemic therapies (Table S2).

In the FAS, the confirmed ORR (primary endpoint) was 42.1%, comprising 24 partial responses (PRs) and one complete response (CR) (Figures 1A–1C). Including unconfirmed responses, the ORR reached 54.4%. The disease control rate (DCR), was 78.9%. Key efficacy outcomes included a median DoR of 12.6 months, a median PFS of 7.7 months (with 6- and 12-month PFS of 60.9% and 35.1%, respectively), and a median OS of 14.9 months (with 12- and 18-month OS of 57.8% and 36.2%, respectively) (Figures 1D–1F). Eighteen patients (31.6%) received treatment beyond 12 months, and seven

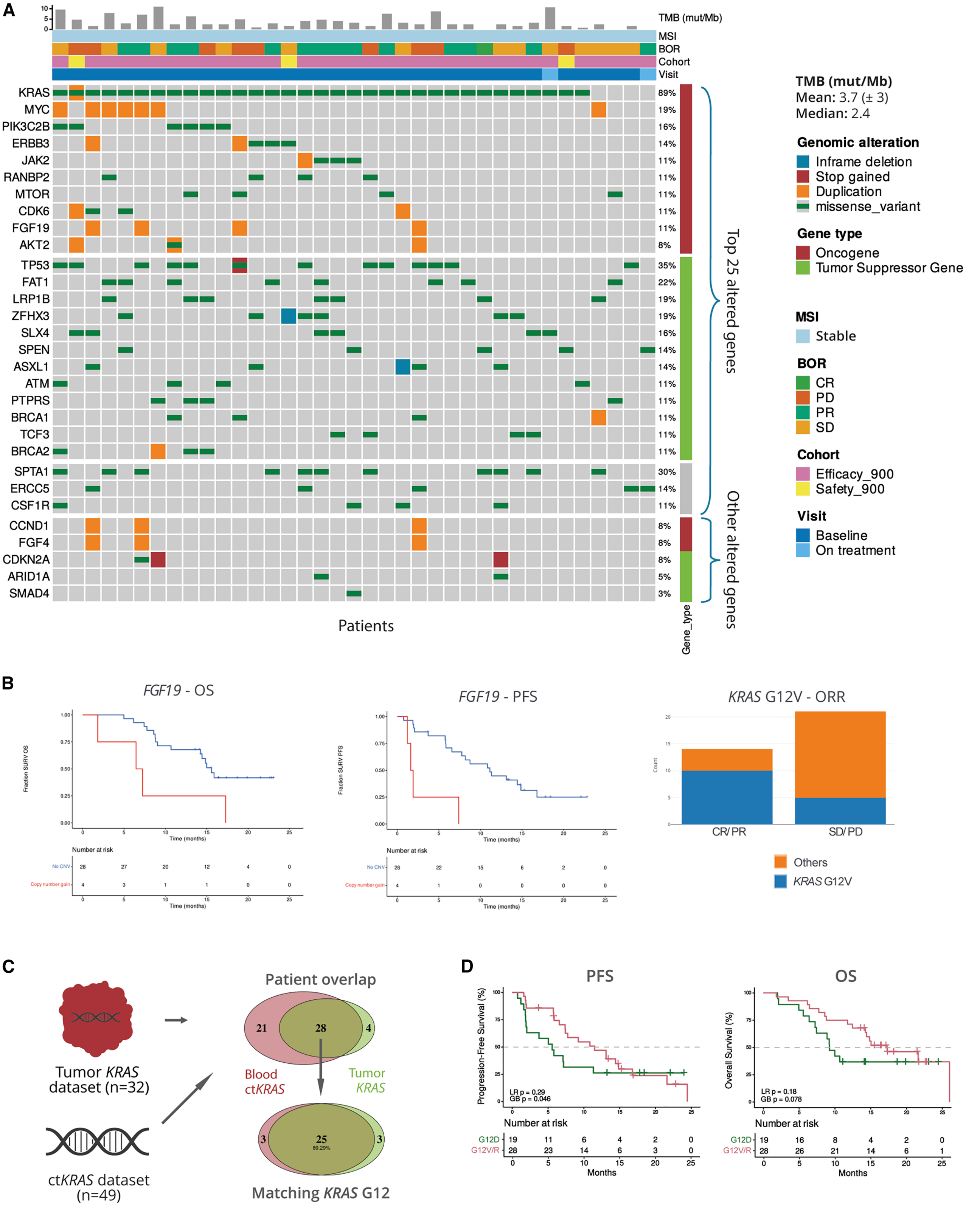

(legend on next page)

CellPress
OPEN ACCESS

patients (12.3%) achieved a late objective response (at approximately the 6-month time point during treatment).

Efficacy outcomes were also analyzed in the intent-to-treat (ITT) population, which included all 65 patients who received at least one dose of 900 μg/kg mitazalimab. In the ITT population, the confirmed ORR was 36.9% (47.7% unconfirmed). The DCR was 69.2%, and the median OS was 14 months. Key efficacy outcomes for both the FAS and ITT populations are shown in Table S3.

Clinical study data analysis: Safety endpoints

Treatment-emergent adverse events (TEAEs) of grade ≥3 were consistent with the known safety profile of mFOLFIRINOX. The most common grade ≥3 TEAEs included neutropenia (26%; 18 patients), hypokalemia (16%; 11 patients), anemia (13%; nine patients), and thrombocytopenia (11%; eight patients). Five patients (7%) discontinued treatment due to TEAEs: one each for condition aggravated, fatigue, chest pain, and headache; general deterioration in physical health; pneumonia; and infusion-related reaction (pruritus). A comprehensive overview of all TEAEs, including those resulting in treatment discontinuations, TEAEs of grade ≥3, and mitazalimab-related TEAEs, is provided in Tables S4 and S5.

Genomic alterations correlate with clinical efficacy outcomes

Genomic alterations, including *KRAS* status, high microsatellite instability (MSI) and tumor mutational burden (TMB), can significantly impact outcomes in PDAC.[17–19,29,30] Therefore, we conducted a genomic analysis on 37 of 57 patients (65%) with evaluable baseline tumor biopsies who received 900 μg/kg of mitazalimab revealing *KRAS* as the most frequently mutated oncogene (89%) and *TP53* as the most commonly mutated tumor suppressor gene (35%) (Figure 2A; Figure S2). All evaluable patients were classified as microsatellite stable, with a median TMB of 2.4 mut/Mb. No patients were classified as TMB-high (Figure 2A).[29]

To investigate the potential of genomic alterations as prognostic biomarkers, we analyzed their association with clinical efficacy endpoints. Using a nominal p value threshold of 0.05 (unadjusted for multiple comparisons), 17 genomic alterations (out of 523 assessed) were found to be associated with clinical efficacy endpoints, including overall response (OR), duration of confirmed response, OS, PFS, OS rate at 12 months (OS-12), PFS rate at 12 months (PFS-12), and DCR (Tables S6 and S7). Among these, alterations in *KRAS*, *FGF19*, *ERCC5*, and *SPEN* loci were identified (Figure 2B; Table S6). Specifically, *FGF19* duplication correlated significantly with shorter OS and PFS, and reduced the likelihood of disease control (Figure 2B). The *KRAS* G12V variant was more prevalent in treatment responders (CR/PR) compared to non-responders (stable disease/PD) (Figure 2B). To further test the association of OS and PFS and *KRAS* G12 variants, we utilized a consensus *KRAS* status dataset that harmonized tumor *KRAS* DNA-sequencing and *KRAS* G12 ctDNA typing (n = 50, Figure 2C; Table S8). Comparative analysis of G12V/R to G12D mutations revealed that patients harboring G12D mutations exhibited a trend toward shorter OS, while PFS was significantly shorter at early time points, as determined by the Gehan-Breslow test (Figure 2D).

Baseline molecular PDAC subtype associates with survival benefit

The molecular subtype of mPDAC has emerged as a prognostic indicator of treatment response to FOLFIRINOX.[22,36] Thus, we studied the influence of PDAC subtype by classifying patients into classical or basal-like molecular subtype (n = 33 samples, n = 31 patients, Figure S3) using gene signatures published by Moffit et al. (Figure 3A)[21] and Zhou et al. (Figure S4A).[20] This analysis revealed that 48% (n = 15) of patients had a classical subtype and 52% (n = 16) had a basal-like subtype. Among the 31 patients with molecular subtype classification, 25 were included in the FAS and were stratified by molecular subtype. Patients with classical mPDAC tumors demonstrated longer OS compared to those with a basal-like subtype (Figure 3B). In the ITT population (n = 30), patients with the classical subtype had significantly longer OS and PFS compared to those with the basal-like subtype (Figure S4B).

We then assessed the relationship between *KRAS* status and molecular subtype by conducting a confounding factor analysis using the consensus *KRAS* G12 status dataset and molecular subtype classifications. Although the G12D *KRAS* variant was more prevalent in the classical subtype (6/13), and the G12V in the basal-like subtype (7/12), this association was not significant (Fisher's exact test, p value = 0.11) (Figure 3C). We then assessed the prognostic power of both variables using a multivariate cox proportional hazards (PH) test. In the OS analysis, a basal-like subtype was associated with worse survival compared to those with classical subtype tumors (HR = 3.84, p value = 0.038) (Figure 3D). Similarly, *KRAS* G12D and G12R mutations were associated with worse survival compared to

Figure 2. Genomic alterations associated with clinical efficacy endpoints

(A) Oncoprint from the TSO500 dataset summarizing somatic genomic alterations in 37 patients who had received the recommended phase 2 dose of 900 μg/kg mitazalimab with available targeted DNA-sequencing data. The three upper panels depict the top 25 most frequently altered genes, as defined by somatic gene variants and copy number variation (CNV; duplication/deletion). The lower panel displays five frequently mutated genes according to literature.[23,24,31–33] Tumor mutational burden (TMB), microsatellite instability (MSI), best overall response (BOR), cohort, and visit are included as column annotations. Genes were ordered based on their function (oncogene or tumor suppressor, as defined by oncoKB database)[34,35], and the frequency of alteration.

(B) Genomic alterations associated with overall survival (OS), objective response rate (ORR), and progression-free survival (PFS) with an unadjusted p value of <0.05.

(C) Derivation of the KRAS subtype consensus dataset for patients in the full analysis set (FAS; n = 50); patient overlap between TSO500 and circulating tumor DNA datasets (top) and intersection of KRAS G12 variants among shared patients in both datasets (bottom).

(D) OS and PFS comparison of patients from the FAS stratified according to KRAS G12V/R and G12D variants from the consensus dataset.

CR, complete response; GB, Gehan-Breslow; LR, log rank; mut/Mb, mutation per megabase; PD, disease progression; PR, partial response; SD, stable disease; SURV, survival.

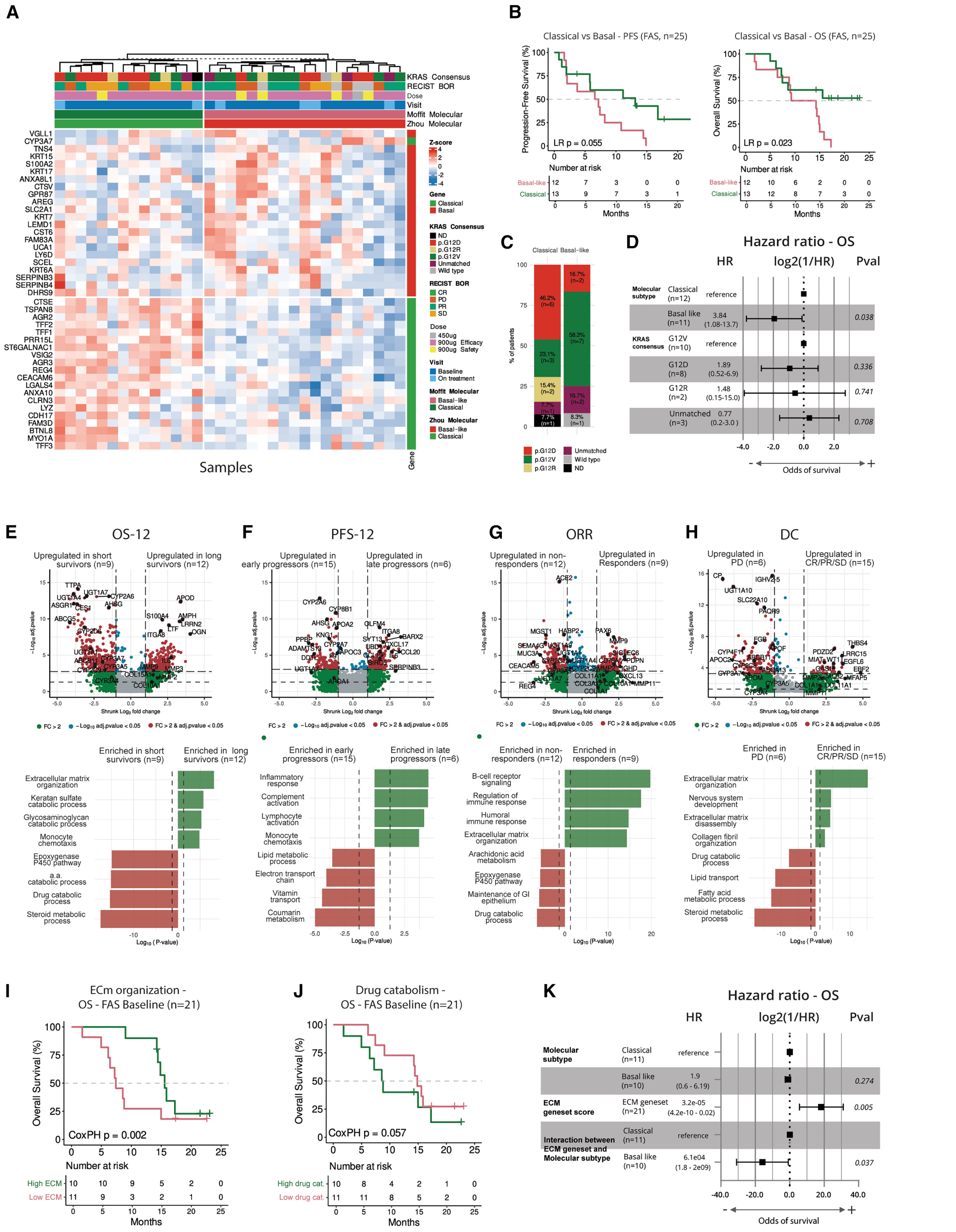

(legend on next page)

the G12V subtype, although these differences were not statistically significant (p value = 0.336 and 0.741, respectively).

Presence of a fibrosis-related gene signature in baseline tumors associates with survival benefits

We hypothesized that biological determinants of the transcriptional profile of baseline PDAC tumors would associate with clinical efficacy. To test this, we conducted a differential gene expression analysis (DGEA) followed by pathway enrichment analysis using RNA sequencing (RNA-seq) data from baseline tumor biopsies obtained from the FAS patients (n = 21, see Figure S3; Table S9) Analysis of survival at 12 months (OS-12) revealed that long survivors (>12 months) exhibited overexpression of genes involved in immune-modulatory pathways (*S100A4* and *IL6*), and extracellular matrix (ECM) remodeling (*MMP2*, *MMP9*, and *COL15A1*) (Figure 3E). Conversely, short survivors (<12 months) displayed increased presence of pathways related to drug and steroid metabolic processes (*UGT1A4*, *UGT1A7*, and *UGT1A1*), primarily through the cytochrome P450 (CYP450) pathway (*CYP2A6*, *CYP3A7*, and *CYP3A5*), as well as lipid and drug efflux pumps (*ABCG5* and *ABCB11*) (Figure 3E). TME deconvolution analysis revealed that long survivors had higher baseline scores of cancer-associated fibroblasts and T cells, along with lower scores of drug and fatty acid metabolism (Figures S4C and S4D).

We next stratified patients by PFS-12 and found that tumors from late progressors (>12 months) were enriched for genes involved in general inflammatory responses (*CXCL17*, *IL6*, and *CD40*), monocyte chemotaxis (*CCL2*, *CCL19*, and *CCL22*), and complement activation (*C1QB*, *C1QA*, and *IGHV*). Early progressors (<12 months) overexpressed genes associated with lipid and coumarin metabolic pathways (*CYP2A6*, *CYP2A7*, *UGT1A7*, *APOA1*, *APOA2*, and *APOC3*) (Figure 3F). Patients who responded to treatment or achieved disease control demonstrated overexpression of genes involved in ECM remodeling and collagen fibril organization (*COL1A1*, *COL11A1*, *COL3A1*, *MMP2*, *MMP9*, and *MMP11*) (Figures 3G and 3H). Responders particularly overexpressed genes related to regulation of immune responses and B cell biology (*CXCL13*, *SIGLEC6*, *CD79A*, and *IGHD*) (Figure 3G). Tumors from non-responders and quick progressors were characterized by an enrichment of pathways involved in drug and steroid metabolic processes via the CYP450 system (*CYP2A6*, *CYP3A7*, and *CYP3A5*), as well as drug efflux pumps and inactivating enzymes (*ABCG5*, *ABCB11*, *UGT1A4*, and *UGT1A7*) (Figures 3G and 4H).

To assess the predictive power of these processes, we generated gene set scores and tested their influence on OS using univariate cox PH analysis. A high score in the ECM organization gene set was significantly associated with longer OS, including genes involved in ECM remodeling such as *MMP2* and *MMP14* (Figure 3I; Figure S4E). Conversely, a high score in the drug catabolism gene set showed a trend toward shorter OS (Figure 3J). Finally, we evaluated the association of the ECM organization gene set and molecular subtype with OS using a multivariate cox PH test (Figure 3K). Higher ECM gene set scores were associated with a greater probability of survival. Patients with the classical tumor subtype displayed a lower HR compared to those with the basal-like tumor subtype. The interaction between ECM organization and molecular subtype was also found to be significant (Figure 3K). The ECM gene set emerges as a promising predictive biomarker for patients with mPDAC undergoing treatment with mitazalimab and mFOLFIRINOX.

Mitazalimab-induced changes in peripheral immune cell populations associate with clinical efficacy outcomes

The unique design of OPTIMIZE-1, incorporating a tumor-priming dose of mitazalimab prior to chemotherapy,[16] allowed us to study the correlation between CD40 stimulation and survival benefits (Figure 4A, "Tumor-priming phase—Mita effects"). Analysis of mitazalimab-induced immunophenotype changes during the tumor-priming phase, measured by fold change (FC) from baseline, identified 23 immune cell populations associated with OS at a nominal p value threshold of 0.05 (Figure 4B). Notably, increasing percentages of proliferating T cell and NK cell populations, including Ki67^{+} T cells, Ki67^{+} CD8 T effector memory cells, Ki67^{+} CD4 T central memory cells, and Ki67^{+} natural killer T (NKT) cells, were associated with longer OS (Figure 4C). Further analysis revealed 18 immune cell populations associated with PFS at a nominal p value threshold of 0.05, with seven at C1D2 and 11 at C1D8. Increased FC at C1D2 of Ki67^{+} T cells and Ki67^{+} naive CD4 T cells, as well as FC at C1D8 of Ki67^{+} NK cells and plasmablasts, correlated

Figure 3. Transcriptomic profiles of patients in OPTIMIZE-1

(A) Molecular subtype classification into "classical" and "basal-like" profiles (n = 33 samples; n = 31 patients) using the gene set published by Moffit et al.[21]

(B) Overall survival (OS) and progression-free survival (PFS) differences in the full analysis set (FAS) stratified by molecular subtype (n = 25).

(C) Overlap between KRAS G12 and molecular subtypes.

(D) Forest plot derived from a multivariate cox proportional hazards (PH) model displaying the hazard ratio (HR) of OS.

(E) Differentially expressed genes (DEGs) and enriched pathways based on OS at 12 months (OS-12; long survivors, post-12 months; short survivors, pre-12 months).

(F) DEGs and enriched pathways-based PFS at 12 months (PFS-12; late progressors, post-12 months; early progressors, pre-12 months).

(G) DEGs and enriched pathways-based on overall response (OR; responders considered complete response [CR]/partial response [PR]; non-responders considered stable disease [SD]/disease progression [PD]).

(H) DEGs and enriched pathways-based on disease control (DC; CR/PR/SD vs. PD).

(I) OS differences based on extracellular matrix (ECM) remodeling organization gene set scores of patients from the FAS at baseline.

(J) OS differences based on drug catabolic processes gene set scores of patients from FAS at baseline.

(I and J) Contributions were tested via univariate cox PH using ssGSEA gene set scores as continuous numerical data, but the scores were represented as a categorical variable by stratifying the patients according to the median (high: >median, low: <median).

(K) Forest plot derived from a multivariate cox PH model displaying the HR of OS. Molecular subtype was analyzed as a categorical variable with "classical" as reference group, while the ECM Gene set was analyzed as a continuous variable.

aa, amino acid; BOR, best overall response; FC, fold change; GI, gastrointestinal; LR, log rank; ND, not determined.

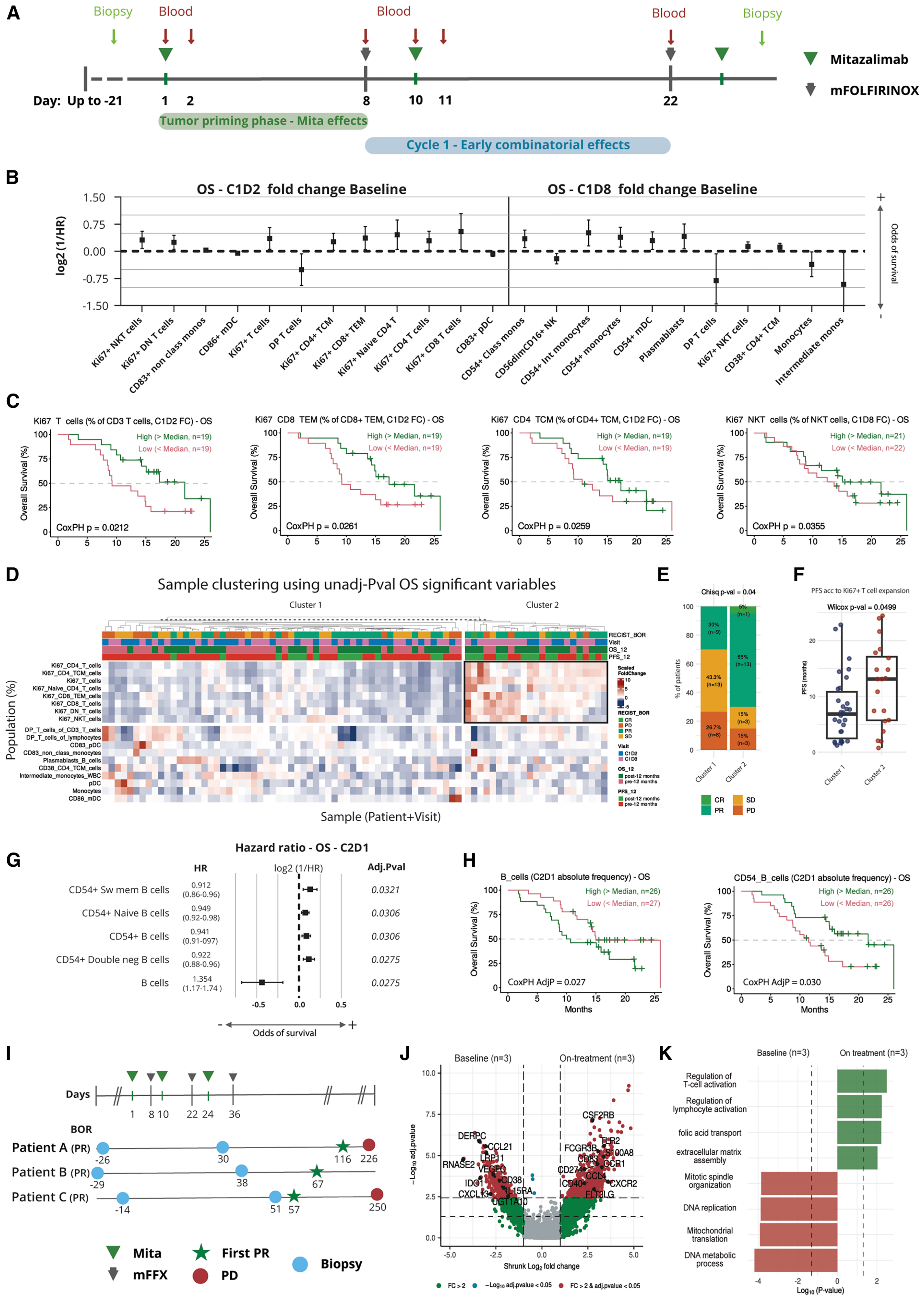

(legend on next page)

CellPress
OPEN ACCESS

with longer PFS (Figures 5A and 5B). A subset of patients exhibiting an expansion of proliferating Ki67^{+} T cell populations at C1D2 and C1D8 showed significantly higher ORR and longer PFS compared to those without Ki67^{+} T cell expansion in circulation (Figures 4D–4F). Overall, these findings suggest a potential direct contribution of activation and proliferation of circulating T cells and NK cells, induced by the initial tumor-priming dose of mitazalimab, to improved clinical outcomes.

Following on from this, we evaluated the "early combinatorial effects" of mitazalimab and mFOLFIRINOX on immune phenotypes, cytokines and clinical outcomes (Figure 4A) by analyzing immune phenotypic associations at the end of cycle 1 with survival benefits. At an adjusted *p* value of 0.05, increased absolute frequency levels of four CD54^{+} B cell populations and decreased total B cell frequency were associated with improved OS (Figures 4G and 4H). Additionally, six B cell subsets as well as interleukin (IL)-6 were associated with shorter PFS (Figures 5C and 5D). The observed activation of B cells and activation-induced margination of B cells are hallmarks of a CD40 agonist response and suggest that increased CD40 stimulation in response to mitazalimab supports improved survival outcomes.

Mitazalimab + mFOLFIRINOX induced intratumoral myeloid cell activation in patients with an objective response

To assess treatment-induced changes in the TME, we performed RNA-seq analysis on paired biopsies from three patients with a PR (Figure 4I). Intratumoral transcriptomic changes following treatment were identified through DGEA comparing on-treatment and baseline samples. The analysis identified 1,207 differentially expressed genes (DEGs) (Figure 4J). After treatment with mitazalimab and mFOLFIRINOX, there was an upregulation of genes involved in myeloid cell biology (*CSF2RB*, *TLR2*, *S100A8*, *FLT3LG*, *CCR1*, and *FCGRIIIB*) and regulation of T cell responses (*CD83*, *CD274*, and *CCL4*). Concurrently, downregulation was seen in immunosuppressive genes (*IDO1*, *CXCL13*, and *IL15RA*) as well as *VEGFC*, *CCL21*, and *CD38*. Pathway enrichment analysis, conducted at a nominal *p* value threshold of 0.05, revealed significant enrichment in lymphocyte-related pathways (Figure 4K). Due to the limited number of paired biopsies obtained from the study, we conducted an additional analysis using a PDAC (KPCY) tumor bearing hCD40tg mouse model to define systemic and intratumoral effects of mitazalimab in combination with FOLFIRINOX (Figure S5E). This model closely recapitulated the clinical and systemic events observed in patients with mPDAC (Figures 5F–5H). Notably, mitazalimab-treated mice exhibited an increase in intratumoral Ki67^{+} effector CD8^{+} T cells, monocytes, and macrophages. This was accompanied by an upregulation of granzyme B (GzmB) in cytotoxic cells, including effector, CM, and naive CD8^{+} T cells, as well as NK cells (Figure S5I). Together, the data support the role of myeloid cells and T cells in the treatment response to mitazalimab when combined with mFOLFIRINOX.

Early on-treatment changes in ctKRAS levels as a prognostic indicator

To assess circulating KRAS (ctKRAS) as a surrogate biomarker for tumor burden in FAS patients, *KRAS* mutant allele frequency (MAF) was determined in ctDNA obtained from blood samples (n = 46) and compared with the sum of the longest diameter (SLD) of tumor lesions over the first 300 days of treatment (Figure 5A; Figure S6). Fluctuations in %MAF of ct*KRAS* coincided with changes in % SLD, although with varying magnitudes and temporal offsets. The relationship between SLD and %MAF of ct*KRAS* was exponential, as evidenced by the strong linear relationship observed between SLD and $\log_{10}$ (%MAF), as well as between percent change in SLD and %MAF from baseline (Figure 5B; Figure S7A).

We subsequently tested the relationship between longitudinal changes in ct*KRAS* levels and clinical benefit from treatment (n = 43, Figure S6). Clearance of ct*KRAS* at any time point within the first 225 days was significantly associated with improved OS

Figure 4. Tumor extrinsic factors associated with clinical efficacy endpoints during the first cycle of OPTIMIZE-1
(A) Dosing and sampling regimen in OPTIMIZE-1.
(B) Forest plot derived from univariate cox proportional hazards (PH) models displaying fold change (FC) in immune cell frequencies during the tumor priming phase of OPTIMIZE-1 (p < 0.05), associated with the odds of survival (log2[−HR]).
(C) Mitazalimab-induced FC in immune cell frequency associated with overall survival (OS) during the tumor-priming phase of OPTIMIZE-1 (p < 0.05). Sample numbers are based on data availability for each population.
(D) Sample clustering based on FC in immune cell frequency, induced during the tumor-priming dose of mitazalimab, associated with OS (p < 0.05). Samples clustered via K-means.
(E) Differences in best overall response (BOR) in patients stratified by the induction of T cell proliferation in peripheral blood, induced by the tumor-priming dose of mitazalimab.
(F) Differences in progression-free survival (PFS) in patients stratified by the induction of T cell proliferation in peripheral blood, induced by the tumor-priming dose of mitazalimab. Data are represented as median ± range.
(G) Forest plot derived from univariate cox PH models displaying cycle (C) 2, day (D) 1 levels of immune cell frequencies and cytokines associated with the odds of survival (log2[−HR]), during the first treatment cycle of OPTIMIZE-1 (adjusted p value < 0.05).
(H) Immune cell frequency levels on C2D1 associated with OS (adjusted p value < 0.05), induced by the combination of mitazalimab and mFOLFIRINOX (mFFX) during the first treatment cycle of OPTIMIZE-1. Sample numbers are based on data availability for each population.
(I) Intratumoral transcriptomic changes driven by mitazalimab in combination with mFFX during treatment. Paired tumor biopsies from three patients that achieved a partial response (PR) as BOR were analyzed.
(J) Differentially expressed genes (DEGs) from paired biopsies.
(K) Enriched pathways based on paired biopsies.
CR, complete response; DN, double-negative; DP, double-positive; HR, hazard ratio; int, intermediate; mDC, myeloid dendritic cells; Mita, mitazalimab; NK, natural killer; NKT, natural killer T cells; non class monos, non-classical monocytes; PD, disease progression; pDC, plasmacytoid dendritic cells; SD, stable disease; TCM, T central memory; TEM, T effector memory.

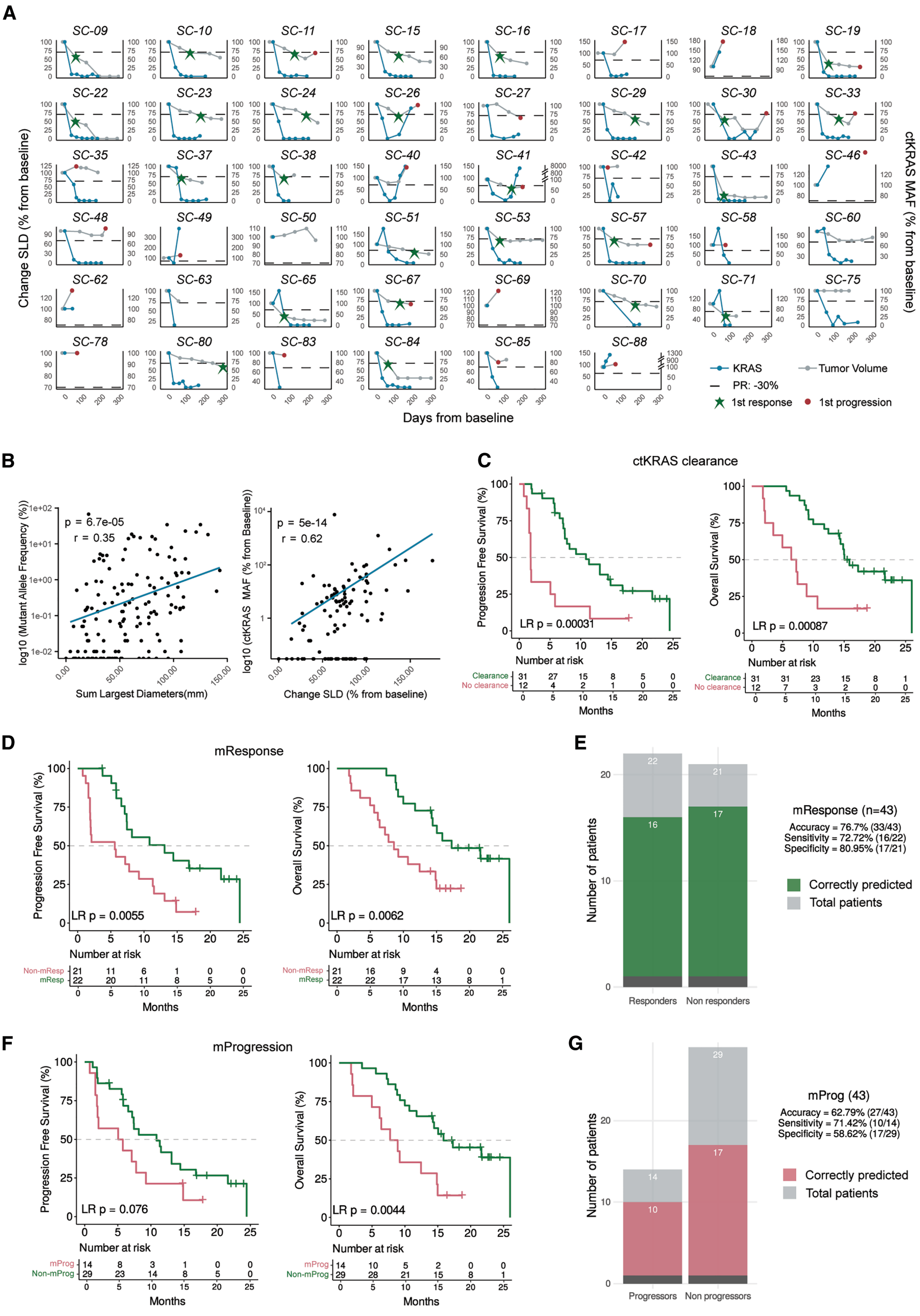

(legend on next page)

(legend on next page)

and PFS (Figure 5C). ct*KRAS* clearance was achieved in 72% of patients (31/43). Stratification by ct*KRAS* G12 mutation status revealed that patients with the *KRAS* G12V variant reached ct*KRAS* clearance more frequently than those with the *KRAS* G12D variant (17/20 vs. 9/18 respectively, Fisher test p value = 0.0354). Furthermore, ct*KRAS* clearance was significantly associated with longer OS and PFS in G12D patients (Figures S7B–S7E), while G12V patients with ctKRAS clearance showed significantly longer PFS and a trend toward longer OS (Figure S7F).

Further, patients exhibiting a molecular response (mResponse), defined as at least 90% reduction in %MAF within the first 70 days of treatment, demonstrated longer OS and PFS compared to non-mResponders (Figure 5D). Similar trends were observed when stratifying patients according to *KRAS* G12 subtype (Figures S7G and S7H). The accuracy of mResponse in predicting radiological response, as assessed by conventional computed tomography (CT) scan, was 76.7%; with a sensitivity of 72.7% and a specificity of 81.0% (Figure 5E; Table S10). On average, ct*KRAS* mResponse was detected 47.2 ± 30.8 days before radiological response as per Response Evaluation Criteria in Solid Tumors (RECIST) criteria. Conversely, patients experiencing molecular progression (mProgression), defined as at least 10% increase in ct*KRAS* %MAF from a previous measurement within the first 225 days of treatment, had significantly shorter OS and PFS compared to non-mProgressors (Figure 5F). Similar trends were observed when stratifying by *KRAS* G12 subtype (Figures S7I and S7J). The accuracy of mProgression in predicting radiological progression, as determined by conventional CT scan, was 62.8%, with a sensitivity of 71.4% and a specificity of 58.6% (Figure 5G; Table S11). On average, ct*KRAS*-based mProgression was detected 39.1 ± 44.6 days before radiological progression per RECIST criteria.

DISCUSSION

In this study, we report updated safety and efficacy data on mitazalimab in combination with mFOLFIRINOX in the OPTIMIZE-1 trial. The correlative analyses, including RNA sequencing of tumor tissue and cytokine/flow cytometry analysis of peripheral blood, were exploratory and hypothesis-generating. Using this multi-omic approach, we identified determinants associated with treatment outcomes. Notably, we discovered a fibrosis-related gene signature that may help identify patients with a better outcome to treatment with mitazalimab plus mFOLFIRINOX in terms of OS. Additionally, mitazalimab-induced activation of peripheral immune cell populations—including T cells, B cells, NK cells, and myeloid cells—was associated with longer survival. Findings also show that treatment triggers changes in ctDNA that both precede and associate with clinical outcomes. Collectively, these biological effects induced by mitazalimab align with the hypothesized mechanism of anti-tumor activity for a CD40 agonist when combined with chemotherapy and support the treatment approach implemented in the OPTIMIZE-1 trial.

In this analysis, based on a median follow-up of 18-month, the confirmed ORR increased from 40% at 12 months to 42% due to one additional late responder. The median DoR was 12.6 months representing a 2-fold improvement compared to FOLFIRINOX and a 50% increase over NALIRIFOX.[3,16,37] PFS remained superior to historical benchmarks, and the mature 18-month OS rates improving to twice that of historical controls and surpassing current standard of care expectation.[37–39] Importantly, no new safety signals emerged since the last update.[16] These results suggest encouraging efficacy and a manageable safety profile of mitazalimab in combination with mFOLFIRINOX in the treatment of mPDAC; however, confirmation in a planned randomized phase 3 study is warranted to establish the benefit of this regimen.

Multiple CD40 agonists have been investigated in clinical trials for pancreatic cancer, as recently reviewed by McVey et al.[40] These agonists can be broadly categorized based on their requirement for concurrent interaction with Fc gamma receptors (FcγR): some are FcγR-conditional (e.g., sotigalimab and mitazalimab), while others are non-conditional (e.g., selicrelimab and CDX-1140).[41] Among the FcγR-conditional agonists, differences in structure of the Fc region further influence their clinical activity. For example, mitazalimab has a wild-type IgG1 Fc, whereas sotigalimab features a mutated Fc with enhanced affinity for FcγR2b. These structural and functional distinctions may impact both safety and efficacy profiles.[41]

The phase 2 PRINCE trial, evaluating sotigalimab in combination with chemotherapy (gemcitabine/nab-paclitaxel) in patients with mPDAC, reported negative results for the chemo/CD40 combination arm.[14,42] The PRINCE trial differed from OPTMIZE-1 in several important aspects, including the chemotherapy backbone (gemcitabine/nab-paclitaxel vs. mFOLFIRINOX) and the sequence of agent administration (sotigalimab was given after chemotherapy in PRINCE, whereas mitazalimab was administered as a priming dose prior to the first dose of chemotherapy in OPTIMIZE-1). The priming dose of mitazalimab in OPTIMIZE-1 was specifically designed to leverage CD40-mediated degradation of tumor stroma before the initiation of chemotherapy, which may enhance drug delivery and efficacy. While direct comparisons between studies are challenging due

Figure 5. Correlation of ctDNA with tumor burden and association with clinical efficacy endpoints

(A) Longitudinal changes in tumor volume, expressed as sum of longest diameters (SLD), and ctKRAS levels, displayed as % mutant allele frequency (MAF), from baseline during the first 300 days of treatment.

(B) Correlation of SLD and log10 (%MAF) in absolute numbers (left), and percentage from baseline (right). Differences in PFS and OS after stratifying patients according to ctKRAS.

(C) ctKRAS clearance.

(D) Molecular Response.

(E) Metrics of the mResponse predictor.

(F) Molecular Progression.

(G) Metrics of the mProgression predictor.

PR, partial response.

to differences in trial design, patient populations, and dosing strategies, it is noteworthy that the development of other CD40 agonists has not advanced, largely due to limited efficacy signals in clinical trials. In contrast, the safety, efficacy, and pharmacodynamic data from OPTIMIZE-1 suggest that mitazalimab, in combination with mFOLFIRINOX and with an optimized dosing schedule, may offer a more favorable therapeutic profile. These findings support the rationale for further investigation of mitazalimab in this setting and highlight the importance of both agent selection and trial design in the development of CD40-targeted therapies for pancreatic cancer.

The mutational profile of patients in OPTIMIZE-1 aligns with previously reported patterns in mPDAC.[23,24] *KRAS* was the most frequently altered gene, with single nucleotide variants (SNVs) at position G12 being the predominant alteration, consistent with earlier studies.[23,43] Patients with the G12V variant demonstrated increased response rates and, when combined with those carrying the G12R variant, a longer PFS compared to individuals with the G12D variant. However, no statistically significant difference in OS was observed between G12V/R variants and G12D carriers, contrasting with some reports.[17–19] The relationship between *KRAS* G12 status and tumor molecular subtype (classical and basal-like) emerged as a potential confounding factor in our analysis. Although the molecular subtype classification in OPTIMIZE-1 was largely consistent with previous data, *KRAS* G12D showed partial, yet non-significant, enrichment in patients with the classical subtype. This observation differs from previous findings suggesting independence between *KRAS* G12 status and molecular subtype.[21,36] Multivariate Cox PH analysis confirmed molecular subtype as a prognostic factor for OS, whereas *KRAS* G12 subtype was not. These findings suggest that molecular subtype exerts a stronger prognostic influence than *KRAS* G12 status, as proposed by Singh et al.,[36] or that our analysis lacks sufficient power to detect OS differences for individual G12 variants. Furthermore, they highlight the complex role of genetic mutations and subtypes in mPDAC, stressing the need for more research on their impact on immunotherapy.

The desmoplastic stroma in PDAC plays a key role in immune exclusion and treatment resistance.[4] In OPTIMIZE-1, a priming dose of mitazalimab was introduced prior to chemotherapy to activate CD40-expressing myeloid cells facilitating stromal degradation and enhancing the efficacy of chemotherapy.[15,44] A key finding in this study is the identification of a baseline, fibrosis-related gene signature, associated with ECM organization and linked to survival benefits. Moreover, key genes involved in matrix remodeling, such as MMP2 and MMP14, were significantly associated with prolonged OS. This aligns with preclinical data indicating that tumors with high MMP2 and MMP14 expression are more likely to respond to CD40 agonists in combination with chemotherapy.[15] These findings support ECM remodeling as a potential predictive biomarker for response to mitazalimab plus mFOLFIRINOX, endorsing the underlying hypothesis and rationale for the priming dose of mitazalimab. Further studies, including orthogonal methods such as multiplex immunohistochemistry (IHC) approaches, are needed to validate the predictive utility of this fibrosis-related gene signature, which represents a promising biomarker candidate. Notably, no published data are available to demonstrate that this specific fibrosis signature predicts outcomes with mFOLFIRINOX alone, yet its predictive relevance for mitazalimab cannot be definitively established without a comparator group.

In addition to identifying potential determinants of treatment response, gene expression analysis of baseline tumors revealed a distinct signature associated with poor treatment outcomes to mitazalimab in combination with mFOLFIRINOX. This signature, characterized by overexpression of steroid metabolism, drug catabolism, and epoxygenase p450 pathways, was detected in short-survivors, early progressors and non-responders. These pathways encompass multiple chemoresistance mechanisms that may lead to the inactivation[45–47] or enhanced efflux[48,49] of mFOLFIRINOX components. Collectively, they contribute to a complex network of resistance mechanisms associated with poor prognosis, independently of the mitazalimab component in the treatment regimen.

In OPTIMIZE-1, the first dose of mitazalimab is administered prior to chemotherapy. This approach contrasts prior studies wherein CD40 is delivered after the first dose of chemotherapy.[42,44] As a consequence of the study design, OPTIMIZE-1 offers a unique opportunity to define the immunological effects induced by CD40 agonist monotherapy and their association with survival. This is relevant given that chemotherapy can negatively affect the quality and quantity of CD40-expressing myeloid cells.[50] Previous reports have described longitudinal immune phenotype changes in OPTIMIZE-1.[16] Herein, we show the association of these mitazalimab-induced changes with survival benefits. Notably, the frequency of activated antigen-presenting cells (APCs) and proliferating T cell and NK cell subsets triggered by mitazalimab correlates with longer OS, PFS and a higher response rate. These findings suggest that mitazalimab induces rapid activation and mobilization of APCs in the circulation, leading to the secretion of key stimulatory cytokines—interleukin (IL)-12, TNF-α, and interferon (IFN)-γ—within 24 h, which in turn drives T cell expansion in peripheral blood.[10,16,51,52] Our results deviate from those recently published by Padrón and colleagues, who found no significant correlation between T cell proliferation, monocyte activation, and survival.[13] In addition, we did not observe any baseline immunophenotypic signatures that associated with improved survival.[13] These discrepancies may be due to differences in the timing and sequence of CD40 agonist administration with respect to chemotherapy and their impact in shaping anti-tumor immune responses.[10]

We identified associations between immune phenotype changes and survival following the first full treatment cycle in OPTIMIZE-1. These "early combinatorial" treatment-induced changes included increases in the relative frequency of $CD54^+$ B cells and a decrease in the overall frequency of B cells, both of which were associated with improved survival. CD54, a molecule involved in leukocyte extravasation and previously used as a biomarker for APCs in immunotherapy, may indicate activation, extravasation, and migration of APCs into peripheral tissues when its frequency increases relative to the total leukocyte pool.[53,54] Additionally, cytokine analysis revealed that patients with low IL-6 levels at C2D1 had longer PFS, consistent with findings from similar studies on other CD40 agonists.[13,50] Notably, the mitazalimab-induced elevation in IL-6 levels

observed in OPTIMIZE-1 was lower compared to those reported with other CD40 agonists such as selicrelumab, an effect attributed to the design of mitazalimab.[52,55] This finding reinforces both the efficacy and, importantly, the favorable safety profile of mitazalimab, as IL-6-driven immunosuppression is associated with mild AEs including pyrexia, rigors, headaches, and infusion-related reactions.[16,56] Collectively, these findings underscore that the CD40-mediated immunologic responses induced by mitazalimab are associated with improved survival.

Analysis of paired tumor biopsies revealed significant treatment-induced changes in the TME following mitazalimab and mFOLFIRINOX therapy. Notably, an upregulation of myeloid APC-associated and T cell modulatory genes was observed, indicative of successful recruitment and expansion of myeloid APCs in tumors. Concurrently, a decrease in gene set scores related to extracellular vesicle and exosome assembly and secretion was found, potentially associated with reduced tumor size. This result shows an increase in myeloid APCs following treatment with a combination of CD40 agonist and chemotherapy in mPDAC. Although the low number of paired biopsies available for this study (3) limits the interpretation of these findings, they align with the qualitative increase of macrophages reported by Padrón et al. after sotigalimab treatment and additional earlier studies in the neoadjuvant setting.[13]

ctDNA is an emerging, minimally invasive, biomarker with the potential to predict response or resistance before radiological assessment, as well as to enable more precise monitoring of ongoing treatment efficacy. Importantly, ctDNA is an established marker of chemotherapy response in pancreatic cancer.[26,27] In OPTIMIZE-1, *KRAS* was the most frequently mutated oncogene, consistent with prior reports.[24,33] We, and others, have previously shown that the predominant alteration is a SNV at position G12.[16,23,43] While baseline ct*KRAS* levels did not significantly correlate with OS across all patients, stratification by *KRAS* G12 status revealed that increasing baseline ct*KRAS* MAF levels correlated with lower OS and PFS in G12V, but not G12D patients. This contrasts with results from the PRINCE and SOC cohorts reported by Till et al., who observed an association between ct*KRAS* %MAF levels and OS and PFS in patients with the G12D variant only, albeit in relatively small cohorts.[25] Importantly, ct*KRAS* clearance following treatment was associated with longer OS and PFS, consistent with earlier studies.[25] Patients achieving mResponse or those without mProgression showed benefits in OS and PFS, with these molecular changes preceding radiological assessments by 47 and 39 days, respectively. These observations highlight the potential of ctDNA analysis as an early predictor of treatment outcomes in mPDAC, but further studies will be needed to determine the specific contribution of mitazalimab to changes in ctDNA observed with chemoimmunotherapy in mPDAC.

In conclusion, mitazalimab, in combination with mFOLFIRINOX, continues to exhibit a manageable safety profile and promising clinical activity, with a favorable DoR contributing to a meaningful survival benefit. Through comprehensive multi-omic analyses, we have identified both tumor-intrinsic and -extrinsic biomarkers associated with improved survival. Of particular significance is the fibrosis-related gene signature, which appears to be directly linked to the expected mechanism of action of mitazalimab and holds potential as a predictive biomarker, pending further confirmatory studies. Finally, the immune phenotypic changes induced by mitazalimab align with the expected effects of a CD40 agonist, and their association with survival suggests that mitazalimab plays a role in driving the observed clinical benefit. While the identified biomarkers need to be confirmed in an independent dataset, these findings and biomarker associations provide evidence for a mitazalimab-driven contribution to the response observed in OPTIMIZE-1, supporting its further development.

Limitations of the study

While the study findings provide valuable insights into the clinical activity and biomarker landscape of mitazalimab in combination with mFOLFIRINOX, several limitations should be acknowledged.

The single-arm study design restricts the ability to directly attribute observed clinical benefits to mitazalimab. In addition, the OPTIMIZE-1 population differed from those in the PRODIGE and MPACT trials,[37,38] with a higher proportion of female patients (58% vs. 38%–43%) and fewer patients with liver metastases (74% vs. 85%–88%); however, ORR and OS were similar between males and females (see Table S12).

The limited number of samples, including paired biopsies, and the variability in sample sizes for the different exploratory analysis impacts the generalizability of the findings, the statistical power of subgroup analyses, and the ability to comprehensively assess intratumoral changes at an individual level. As a result, further validation of the identified biomarkers in independent cohorts is required. In addition, the deconvolution of the TME using bulk RNA-seq did not allow for detailed characterization of cell populations (e.g., T cells, myeloid cells, and fibroblasts), nor was orthogonal validation using IHC possible due to limited biopsy material. More comprehensive phenotyping and validation using additional methods will be important in future studies. Similarly, given the limited sample size, multiple comparisons, and lack of a validation cohort, the peripheral blood analyses are exploratory. Findings will need to be validated in larger, independent cohorts that include appropriate control arms.

While a retrospective control group of patients treated with mFOLFIRINOX might have been informative, the marked heterogeneity of PDAC tumor and stromal biology, as well as limited sample availability, complicates the feasibility of this approach. As a result, the subgroup analyses for OS should be considered exploratory and hypothesis-generating only, and these findings require validation in larger, independent cohorts to establish their clinical significance. The heterogeneity in biopsy timing relative to clinical response (between 6 and 86 days) could also influence the interpretation of longitudinal immune and transcriptomic changes. Importantly, both ctDNA clearance and classical versus basal molecular subtypes are established biomarkers of chemotherapy response. Thus, the contribution of mitazalimab to mFOLFIRINOX-induced changes in ctDNA remains unclear. Finally, despite the potential of ctDNA-based monitoring, challenges remain in defining its precise role in predicting clinical outcomes, underscoring the need for larger, well-controlled studies to refine the clinical utility of ctDNA as a predictive biomarker in mPDAC.

RESOURCE AVAILABILITY

Lead contact

Further information and requests for resources and reagents should be directed to and will be fulfilled by the lead contact, Peter Ellmark (pek@alligatorbioscience.com).

Materials availability

This study did not generate new unique reagents.

Data and code availability

- RNA sequencing data are available in Gene Expression Omnibus at GEO: GSE291331.
- This manuscript does not report custom computer code.
- Any additional information required to reanalyze the data reported in this work paper is available from the lead contact upon request.

ACKNOWLEDGMENTS

This study and publication were funded by Alligator Bioscience AB. The authors thank the patients and their families and carers for their participation in OPTIMIZE-1. The authors would also like to thank all investigators and study site coordinators. Flow cytometry and cytokine associations with clinical efficacy endpoints were conducted by FIOS genomics (Edinburgh, UK). The authors would like to thank Tommy Schyman, BC Platforms, for statistical analysis. Medical writing support was provided by Kara McNair, MediComm Partners, which was sponsored by Alligator Bioscience AB in accordance with Good Publication Practice guidelines.

AUTHOR CONTRIBUTIONS

Conceptualization, J.-L.V.L., P.E., K.E.S., S.V.A., Y.P.d.C., and D.G.J.; methodology, K.E.S., J.-L.V.L., P.E., S.V.A., Y.P.d.C., D.G.J., and G.L.B.; formal analysis, D.G.J., G.L.B., and Y.P.d.C.; investigation, E.M.O'R., G.L.B., D.G.J., J.-L.V.L., I..B, H.P., K.G., A.L., E.M., P.C., J.-F.B., L.P., J.F., M.R.G., R.A.P.-C., I.G., K.N., and T.M.M.; data curation, D.G.J.; writing – original draft, D.G.J., P. E., K.E.S., and Y.P.d.C.; writing – reviewing and editing, J.-L.V.L., K.G., I.B., T. M.M., A.L., P.C., H.P., E.M., J.-F.B., L.P., J.F., M.R.G., R.A.P.-C., I.G., K.N., E. M.O'R., D.G.J., G.L.B., P.E., K.E.S., Y.P.d.C., and S.V.A.; visualization, D.G.J.; project administration, P.E., K.N., and S.V.A.

DECLARATION OF INTERESTS

G.L.B. reports consulting and grant support to the University of Pennsylvania from Alligator Bioscience for the current manuscript; research grants to the University of Pennsylvania from Bristol Myers Squibb, Genmab, Incyte Corporation, and Hibercell; royalties/licenses from Novartis and Tmunity Therapeutics/Kite Pharma as the inventor of intellectual property related to CAR T cells licensed by University of Pennsylvania; consulting and advisory board fees from Molecular Partners, Shattuck Labs, EMD Serono, Pancreatic Cancer Action Network, and Alligator Bioscience; owns stock in Pfizer and Johnson & Johnson. P.C. reports institutional support from Alligator Biosciences for the current manuscript; institutional grants/contracts from Abbvie, ADCT, Adial Nortye, Amge, Astex, AstraZeneca, BMS, Boehringer Ingelheim, Blueprint, C4 Therapeutics, Debio Pharm Dalichi Sankyo, Dragonfly, Exelixis, Incyte, Iteos, Janssen, Kinnate, Lilly, Molecular Partners, Novartis, Ose Immunotherapeutics, Pierre Fabre, Relay, Roche/Genentech, Sotio, Taiho, Tango, Toray, Turningpoint, and Transgene; consulting fees from Boehringer Ingelheim, Ose Immunotherapeutics, Scenic, and Brenus; support for attending meetings from Novartis; support for participation on a DSMB/Advisory Board from BMS; and institutional support for receipt of equipment from Debio Pharm, Novartis, and Glaxo Smith Kline. J.F. reports grants/contracts from AMGEN, Roche, Astra Zeneca, Servier, and Viatris. R.A.P.-C. reports grants/contract from EU Horizon Program, Aragon Health Research Institute (IIS Aragon), and Institute de Salud Carlos III; consulting fees from Roche Astra Zeneca, BMS, Servier, Ipsen, and Astellas; payment or honoraria for lectures, presentations, speakers bureau, manuscript writing, or educational events from Astellas, Servier, Astra Zeneca, Roche, Eisai; payment for expert testimony from Servier, Astellas, Astra Zeneca; and support for attending meetings and/or travel from Servier, Astellas, Roche, Astra Zeneca, and Lilly. K.E.S., K.N., D.G.J., Y.P.d.C., P.E., and S.V.A. are employee and shareholder of Alligator Bioscience, AB. P.E. reports grant funding from Swedish Foundation for Strategic Research in his position as Adjunct Professor at Lund University. P.E., K.E.S., and D.G.J. are inventors on the patents relating to mitazalimab (patents owned by Alligator Bioscience). E.M.O.R. reports institutional grant support from Genentech/Roche, BioNTech, AstraZeneca, Arcus, Elicio, Parker Institute, NIH/NCI, Digestive Care, Break Through Cancer, Agenus, Amgen, and Rand evolution Medicines; consulting fees from Arcus, AstraZeneca, Ability Pharma, Alligator Bioscience, Agenus, BioNTech, Ipsen, Ikena, Merck, Morna Therapeutics, Novartis, Leap Therapeutics, Astellas, BMS, Revolution Medicines, Regeneron, and Tango; support for attending meetings and/or travel from BioNTech and Arcus; with other financial/non-financial interests from the American Association of Cancer Research, the American Society of Clinical Oncology, Imedex, Research to Practice, and SU2C.

STAR★METHODS

Detailed methods are provided in the online version of this paper and include the following:

- KEY RESOURCES TABLE
- EXPERIMENTAL MODELS AND STUDY PARTICIPANT DETAILS
  - Human participants
- METHOD DETAILS
  - Study design
  - OPTIMIZE-1: Patient inclusion and exclusion
  - OPTIMIZE-1: Patient treatment
  - OPTIMIZE-1: Efficacy assessments
  - OPTIMIZE-1: Safety assessments
  - Sample collection and handling
  - Genomic profiling of tumor biopsies
  - Transcriptome profiling of tumor biopsies
- QUANTIFICATION AND STATISTICAL ANALYSIS
  - Clinical data analysis
  - Analysis of genomic profiling of tumor biopsies
  - Correlation between circulating KRAS and tumor volume
  - Analysis of transcriptome profiling of tumor biopsies
  - Data analysis of immune cells and cytokines
  - Additional resources

SUPPLEMENTAL INFORMATION

Supplemental information can be found online at https://doi.org/10.1016/j.xcrm.2025.102407.

Received: February 21, 2025
Revised: May 28, 2025
Accepted: September 11, 2025
Published: October 7, 2025

REFERENCES

1. Park, W., Chawla, A., and O'Reilly, E.M. (2021). Pancreatic Cancer: A Review. JAMA *326*, 851–862. https://doi.org/10.1001/jama.2021.13027.
2. Cockrum, P., Dennen, S., Brown, A., Briggs, J., and Paluri, R. (2025). Real-world clinical outcomes and economic burden of metastatic pancreatic ductal adenocarcinoma: a systematic review. Future Oncol. *21*, 241–260. https://doi.org/10.1080/14796694.2024.2435253.
3. Wainberg, Z.A., Melisi, D., Macarulla, T., Pazo Cid, R., Chandana, S.R., De La Fouchardière, C., Dean, A., Kiss, I., Lee, W.J., Goetze, T.O., et al. (2023). NALIRIFOX versus nab-paclitaxel and gemcitabine in treatment-naive patients with metastatic pancreatic ductal adenocarcinoma (NAPOLI 3): a randomised, open-label, phase 3 trial. Lancet *402*, 1272–1281. https://doi.org/10.1016/s0140-6736(23)01366-1.

CellPress
OPEN ACCESS

4. Balachandran, V.P., Beatty, G.L., and Dougan, S.K. (2019). Broadening the Impact of Immunotherapy to Pancreatic Cancer: Challenges and Opportunities. Gastroenterology *156*, 2056–2072.

5. Deicher, A., Andersson, R., Tingstedt, B., Lindell, G., Bauden, M., and Ansari, D. (2018). Targeting dendritic cells in pancreatic ductal adenocarcinoma. Cancer Cell Int. *18*, 85. https://doi.org/10.1186/s12935-018-0585-0.

6. Yao, W., Maitra, A., and Ying, H. (2020). Recent insights into the biology of pancreatic cancer. EBioMedicine *53*, 102655. https://doi.org/10.1016/j.ebiom.2020.102655.

7. Balachandran, V.P., Łuksza, M., Zhao, J.N., Makarov, V., Moral, J.A., Remark, R., Herbst, B., Askan, G., Bhanot, U., Senbabaoglu, Y., et al. (2017). Identification of unique neoantigen qualities in long-term survivors of pancreatic cancer. Nature *551*, 512–516.

8. Wattenberg, M.M., Colby, S., Garrido-Laguna, I., Xue, Y., Chang, R., Delman, D., Lee, J., Affolter, K., Mulvihill, S.J., Beg, M.S., et al. (2024). Intra-tumoral cell neighborhoods coordinate outcomes in pancreatic ductal adenocarcinoma. Gastroenterology *166*, 1114–1129. https://doi.org/10.1053/j.gastro.2024.01.013.

9. Sivakumar, S., Jainarayanan, A., Arbe-Barnes, E., Sharma, P.K., Leathlobhair, M.N., Amin, S., Reiss, D.J., Heij, L., Hegde, S., Magen, A., et al. (2025). Distinct immune cell infiltration patterns in pancreatic ductal adenocarcinoma (PDAC) exhibit divergent immune cell selection and immunosuppressive mechanisms. Nat. Commun. *16*, 1397. https://doi.org/10.1038/s41467-024-55424-2.

10. Beatty, G.L., Li, Y., and Long, K.B. (2017). Cancer immunotherapy: activating innate and adaptive immunity through CD40 agonists. Expert Rev. Anticancer Ther. *17*, 175–186. https://doi.org/10.1080/14737140.2017.1270208.

11. Beatty, G.L., Winograd, R., Evans, R.A., Long, K.B., Luque, S.L., Lee, J.W., Clendenin, C., Gladney, W.L., Knoblock, D.M., Guirnalda, P.D., and Vonderheide, R.H. (2015). Exclusion of T Cells From Pancreatic Carcinomas in Mice Is Regulated by Ly6C(low) F4/80(+) Extratumoral Macrophages. Gastroenterology *149*, 201–210. https://doi.org/10.1053/j.gastro.2015.04.010.

12. Byrne, K.T., Betts, C.B., Mick, R., Sivagnanam, S., Bajor, D.L., Laheru, D.A., Chiorean, E.G., O'Hara, M.H., Liudahl, S.M., Newcomb, C., et al. (2021). Neoadjuvant Selicrelumab, an Agonist CD40 Antibody, Induces Changes in the Tumor Microenvironment in Patients with Resectable Pancreatic Cancer. Clin. Cancer Res. *27*, 4574–4586. https://doi.org/10.1158/1078-0432.CCR-21-1047.

13. Padron, L.J., Maurer, D.M., and O'Hara, M.H. (2022). Sotigalimab and/or nivolumab with chemotherapy in first-line metastatic pancreatic cancer: clinical and immunologic analyses from the randomized phase 2 PRINCE trial. Nat Med *28*, 1167–1177. https://doi.org/10.1038/s41591-022-01829-9.

14. Weiss, S.A., Sznol, M., Shaheen, M., Berciano-Guerrero, M.Á., Couselo, E.M., Rodríguez-Abreu, D., Boni, V., Schuchter, L.M., Gonzalez-Cao, M., Arance, A., et al. (2024). A Phase II Trial of the CD40 Agonistic Antibody Sotigalimab (APX005M) in Combination with Nivolumab in Subjects with Metastatic Melanoma with Confirmed Disease Progression on Anti-PD-1 Therapy. Clin. Cancer Res. *30*, 74–81. https://doi.org/10.1158/1078-0432.CCR-23-0475.

15. Long, K.B., Gladney, W.L., Tooker, G.M., Graham, K., Fraietta, J.A., and Beatty, G.L. (2016). IFNγ and CCL2 Cooperate to Redirect Tumor-Infiltrating Monocytes to Degrade Fibrosis and Enhance Chemotherapy Efficacy in Pancreatic Carcinoma. Cancer Discov. *6*, 400–413. https://doi.org/10.1158/2159-8290.Cd-15-1032.

16. Van Laethem, J.-L., Borbath, I., Prenen, H., Geboes, K.P., Lambert, A., Mitry, E., Cassier, P.A., Blanc, J.F., Pilla, L., Batlle, J.F., et al. (2024). Combining CD40 agonist mitazalimab with mFOLFIRINOX in previously untreated metastatic pancreatic ductal adenocarcinoma (OPTIMIZE-1): a single-arm, multicentre phase 1b/2 study. Lancet Oncol. *25*, 853–864. https://doi.org/10.1016/S1470-2045(24)00263-8.

17. Hendifar, A.E., Blais, E.M., Ng, C., Thach, D., Gong, J., Sohal, D., Chung, V., Sahai, V., Fountzilas, C., Mikhail, S., et al. (2020). Comprehensive analysis of KRAS variants in patients (pts) with pancreatic cancer (PDAC): Clinical/molecular correlations and real-world outcomes across standard therapies. J. Clin. Oncol. *38*, 4641. https://doi.org/10.1200/JCO.2020.38.15_suppl.4641.

18. Bournet, B., Muscari, F., Buscail, C., Assenat, E., Barthet, M., Hammel, P., Selves, J., Guimbaud, R., Cordelier, P., and Buscail, L. (2016). KRAS G12D Mutation Subtype Is A Prognostic Factor for Advanced Pancreatic Adenocarcinoma. Clin. Transl. Gastroenterol. *7*, e157. https://doi.org/10.1038/ctg.2016.18.

19. Ardalan, B., Ciner, A.T., and Baca, Y. (2023). Not all treated KRAS-mutant pancreatic adenocarcinomas are equal: KRAS G12D and survival outcome. J. Clin. Oncol. *41*, 4020.

20. Zhou, X., An, J., Kurilov, R., Brors, B., Hu, K., Peccerella, T., Roessler, S., Pfütze, K., Schulz, A., Wolf, S., et al. (2023). Persister cell phenotypes contribute to poor patient outcomes after neoadjuvant chemotherapy in PDAC. Nat. Cancer *4*, 1362–1381. https://doi.org/10.1038/s43018-023-00628-6.

21. Moffitt, R.A., Marayati, R., Flate, E.L., Volmar, K.E., Loeza, S.G.H., Hoadley, K.A., Rashid, N.U., Williams, L.A., Eaton, S.C., Chung, A.H., et al. (2015). Virtual microdissection identifies distinct tumor- and stroma-specific subtypes of pancreatic ductal adenocarcinoma. Nat. Genet. *47*, 1168–1178.

22. O'Kane, G.M., Grünwald, B.T., Jang, G.H., Masoomian, M., Picardo, S., Grant, R.C., Denroche, R.E., Zhang, A., Wang, Y., Lam, B., et al. (2020). GATA6 Expression Distinguishes Classical and Basal-like Subtypes in Advanced Pancreatic Cancer. Clin. Cancer Res. *26*, 4901–4910. https://doi.org/10.1158/1078-0432.CCR-19-3724.

23. Raphael, B.J., Hruban, R.H., Aguirre, A.J., Moffitt, R.A., Yeh, J.J., Stewart, C., Robertson, A.G., Cherniack, A.D., Gupta, M., Getz, G., et al. (2017). Integrated Genomic Characterization of Pancreatic Ductal Adenocarcinoma. Cancer Cell *32*, 185–203.e13. https://doi.org/10.1016/j.ccell.2017.07.007.

24. Bailey, P., Chang, D.K., Nones, K., Johns, A.L., Patch, A.M., Gingras, M.C., Miller, D.K., Christ, A.N., Bruxner, T.J.C., Quinn, M.C., et al. (2016). Genomic analyses identify molecular subtypes of pancreatic cancer. Nature *531*, 47–52. https://doi.org/10.1038/nature16965.

25. Till, J.E., McDaniel, L., Chang, C., Long, Q., Pfeiffer, S.M., Lyman, J.P., Padrón, L.J., Maurer, D.M., Yu, J.X., Spencer, C.N., et al. (2024). Circulating KRAS G12D but not G12V is associated with survival in metastatic pancreatic ductal adenocarcinoma. Nat. Commun. *15*, 5763. https://doi.org/10.1038/s41467-024-49915-5.

26. Toledo, R.D.A., Calahorro García, A.M., Mirallas, O., Moreno, A., Galvao, V., Alonso, G., Vieito, M., Brana, I., Oberoi, A., Lostes-Bardaji, M.J., et al. (2024). Prognostic and predictive value of ultrasensitive ctDNA monitoring in a metastatic pan-cancer cohort treated with immune checkpoint inhibitors in the context of phase 1 clinical trials. J. Clin. Oncol. *42*, 2510. https://doi.org/10.1200/JCO.2024.42.16_suppl.2510.

27. Raja, R., Kuziora, M., Brohawn, P.Z., Higgs, B.W., Gupta, A., Dennis, P. A., and Ranade, K. (2018). Early Reduction in ctDNA Predicts Survival in Patients with Lung and Bladder Cancer Treated with Durvalumab. Clin. Cancer Res. *24*, 6212–6222. https://doi.org/10.1158/1078-0432.Ccr-18-0386.

28. Kim, M.K., Woo, S.M., Park, B., Yoon, K.A., Kim, Y.H., Joo, J., Lee, W.J., Han, S.S., Park, S.J., and Kong, S.Y. (2018). Prognostic Implications of Multiplex Detection of KRAS Mutations in Cell-Free DNA from Patients with Pancreatic Ductal Adenocarcinoma. Clin. Chem. *64*, 726–734. https://doi.org/10.1373/clinchem.2017.283721.

29. Karamitopoulou, E., Andreou, A., Wenning, A.S., Gloor, B., and Perren, A. (2022). High tumor mutational burden (TMB) identifies a microsatellite stable pancreatic cancer subset with prolonged survival and strong

anti-tumor immunity. Eur. J. Cancer *169*, 64–73. https://doi.org/10.1016/j.ejca.2022.03.033.

30. Palmeri, M., Mehnert, J., Silk, A.W., Jabbour, S.K., Ganesan, S., Popli, P., Riedlinger, G., Stephenson, R., de Meritens, A.B., Leiser, A., et al. (2022). Real-world application of tumor mutational burden-high (TMB-high) and microsatellite instability (MSI) confirms their utility as immunotherapy biomarkers. ESMO Open *7*, 100336. https://doi.org/10.1016/j.esmoop.2021.100336.
31. Waddell, N., Pajic, M., Patch, A.M., Chang, D.K., Kassahn, K.S., Bailey, P., Johns, A.L., Miller, D., Nones, K., Quek, K., et al. (2015). Whole genomes redefine the mutational landscape of pancreatic cancer. Nature *518*, 495–501. https://doi.org/10.1038/nature14169.
32. Witkiewicz, A.K., McMillan, E.A., Balaji, U., Baek, G., Lin, W.C., Mansour, J., Mollaee, M., Wagner, K.U., Koduru, P., Yopp, A., et al. (2015). Whole-exome sequencing of pancreatic cancer defines genetic diversity and therapeutic targets. Nat. Commun. *6*, 6744. https://doi.org/10.1038/ncomms7744.
33. Biankin, A.V., Waddell, N., Kassahn, K.S., Gingras, M.C., Muthuswamy, L.B., Johns, A.L., Miller, D.K., Wilson, P.J., Patch, A.M., Wu, J., et al. (2012). Pancreatic cancer genomes reveal aberrations in axon guidance pathway genes. Nature *491*, 399–405. https://doi.org/10.1038/nature11547.
34. Chakravarty, D., Gao, J., Phillips, S.M., Kundra, R., Zhang, H., Wang, J., Rudolph, J.E., Yaeger, R., Soumerai, T., Nissan, M.H., et al. (2017). OncoKB: A Precision Oncology Knowledge Base. JCO Precis. Oncol. *1*, 1–16. https://doi.org/10.1200/po.17.00011.
35. Suehnholz, S.P., Nissan, M.H., Zhang, H., Kundra, R., Nandakumar, S., Lu, C., Carrero, S., Dhaneshwar, A., Fernandez, N., Xu, B.W., et al. (2024). Quantifying the Expanding Landscape of Clinical Actionability for Patients with Cancer. Cancer Discov. *14*, 49–65. https://doi.org/10.1158/2159-8290.Cd-23-0467.
36. Singh, H., Xiu, J., Kapner, K.S., Yuan, C., Narayan, R.R., Oberley, M., Farrell, A., Surana, R., Huffman, B.M., Perez, K., et al. (2024). Clinical and Genomic Features of Classical and Basal Transcriptional Subtypes in Pancreatic Cancer. Clin. Cancer Res. *30*, 4932–4942. https://doi.org/10.1158/1078-0432.Ccr-24-1164.
37. Conroy, T., Desseigne, F., Ychou, M., Bouché, O., Guimbaud, R., Bécouarn, Y., Adenis, A., Raoul, J.L., Gourgou-Bourgade, S., de la Fouchardière, C., et al. (2011). FOLFIRINOX versus gemcitabine for metastatic pancreatic cancer. N. Engl. J. Med. *364*, 1817–1825.
38. Von Hoff, D.D., Ervin, T., Arena, F.P., Chiorean, E.G., Infante, J., Moore, M., Seay, T., Tjulandin, S.A., Ma, W.W., Saleh, M.N., et al. (2013). Increased survival in pancreatic cancer with nab-paclitaxel plus gemcitabine. N. Engl. J. Med. *369*, 1691–1703.
39. Conroy, T., Hammel, P., Hebbar, M., Ben Abdelghani, M., Wei, A.C., Raoul, J.L., Choné, L., Francois, E., Artru, P., Biagi, J.J., et al. (2018). FOLFIRINOX or Gemcitabine as Adjuvant Therapy for Pancreatic Cancer. N. Engl. J. Med. *379*, 2395–2406. https://doi.org/10.1056/NEJMoa1809775.
40. McVey, J.C., and Beatty, G.L. (2025). Facts and hopes of CD40 agonists as a cancer immunotherapy. Clin. Cancer Res. *31*, 2079–2087. https://doi.org/10.1158/1078-0432.CCR-24-1660.
41. Enell Smith, K., Deronic, A., Hägerbrand, K., Norlén, P., and Ellmark, P. (2021). Rationale and clinical development of CD40 agonistic antibodies for cancer immunotherapy. Expet Opin. Biol. Ther. *17*, 1–12. https://doi.org/10.1080/14712598.2021.1934446.
42. O'Hara, M.H., O'Reilly, E.M., Varadhachary, G., Wolff, R.A., Wainberg, Z.A., Ko, A.H., Fisher, G., Rahma, O., Lyman, J.P., Cabanski, C.R., et al. (2021). CD40 agonistic monoclonal antibody APX005M (sotigalimab) and chemotherapy, with or without nivolumab, for the treatment of metastatic pancreatic adenocarcinoma: an open-label, multicentre, phase 1b study. Lancet Oncol. *22*, 118–131. https://doi.org/10.1016/S1470-2045(20)30532-5.
43. Yousef, A., Yousef, M., Chowdhury, S., Abdilleh, K., Knafl, M., Edelkamp, P., Alfaro-Munoz, K., Chacko, R., Peterson, J., Smaglo, B.G., et al. (2024). Impact of KRAS mutations and co-mutations on clinical outcomes in pancreatic ductal adenocarcinoma. npj Precis. Oncol. *8*, 27. https://doi.org/10.1038/s41698-024-00505-0.
44. Beatty, G.L., Chiorean, E.G., Fishman, M.P., Saboury, B., Teitelbaum, U.R., Sun, W., Huhn, R.D., Song, W., Li, D., Sharp, L.L., et al. (2011). CD40 agonists alter tumor stroma and show efficacy against pancreatic carcinoma in mice and humans. Science *331*, 1612–1616. https://doi.org/10.1126/science.1198443.
45. Mathijssen, R.H.J., van Alphen, R.J., and Verweij, J. (2001). Clinical Pharmacokinetics and Metabolism of Irinotecan (CPT-11). Clin. Cancer Res. *7*, 2182–2194.
46. Santos, A., Zanetta, S., Cresteil, T., Deroussent, A., Pein, F., Raymond, E., Vernillet, L., Risse, M.L., Boige, V., Gouyette, A., and Vassal, G. (2000). Metabolism of irinotecan (CPT-11) by CYP3A4 and CYP3A5 in humans. Clin. Cancer Res. *6*, 2012–2020.
47. Rivory, L.P., and Robert, J. (1995). Identification and kinetics of a beta-glucuronide metabolite of SN-38 in human plasma after administration of the camptothecin derivative irinotecan. Cancer Chemother. Pharmacol. *36*, 176–179. https://doi.org/10.1007/bf00689205.
48. Wang, T., Chen, Z., Zhu, Y., Pan, Q., Liu, Y., Qi, X., Jin, L., Jin, J., Ma, X., and Hua, D. (2015). Inhibition of transient receptor potential channel 5 reverses 5-Fluorouracil resistance in human colorectal cancer cells. J. Biol. Chem. *290*, 448–456. https://doi.org/10.1074/jbc.M114.590364.
49. Adamska, A., and Falasca, M. (2018). ATP-binding cassette transporters in progression and clinical outcome of pancreatic cancer: What is the way forward? World J. Gastroenterol. *24*, 3222–3238. https://doi.org/10.3748/wjg.v24.i29.3222.
50. Wattenberg, M.M., Herrera, V.M., Giannone, M.A., Gladney, W.L., Carpenter, E.L., and Beatty, G.L. (2021). Systemic inflammation is a determinant of outcomes to CD40 agonist-based therapy in pancreatic cancer patients. JCI Insight *6*, e145389. https://doi.org/10.1172/jci.insight.145389.
51. Stone, M.L., Lee, J., Herrera, V.M., Graham, K., Lee, J.W., Huffman, A., Coho, H., Tooker, E., Myers, M.I., Giannone, M., et al. (2021). TNF blockade uncouples toxicity from antitumor efficacy induced with CD40 chemoimmunotherapy. JCI Insight *6*, e146314. https://doi.org/10.1172/jci.insight.146314.
52. Beatty, G.L., Torigian, D.A., Chiorean, E.G., Saboury, B., Brothers, A., Alavi, A., Troxel, A.B., Sun, W., Teitelbaum, U.R., Vonderheide, R.H., and O'Dwyer, P.J. (2013). A phase I study of an agonist CD40 monoclonal antibody (CP-870,893) in combination with gemcitabine in patients with advanced pancreatic ductal adenocarcinoma. Clin. Cancer Res. *19*, 6286–6295.
53. Wee, H., Oh, H.M., Jo, J.H., and Jun, C.D. (2009). ICAM-1/LFA-1 interaction contributes to the induction of endothelial cell-cell separation: implication for enhanced leukocyte diapedesis. Exp. Mol. Med. *41*, 341–348. https://doi.org/10.3858/emm.2009.41.5.038.
54. Sheikh, N.A., and Jones, L.A. (2008). CD54 is a surrogate marker of antigen presenting cell activation. Cancer Immunol. Immunother. *57*, 1381–1390. https://doi.org/10.1007/s00262-008-0474-9.
55. Mangsbo, S.M., Broos, S., Fletcher, E., Veitonmäki, N., Furebring, C., Dahlén, E., Norlén, P., Lindstedt, M., Tötterman, T.H., and Ellmark, P. (2015). The human agonistic CD40 antibody ADC-1013 eradicates bladder tumors and generates T-cell-dependent tumor immunity. Clin. Cancer Res. *21*, 1115–1126. https://doi.org/10.1158/1078-0432.Ccr-14-0913.
56. Velazquez-Salinas, L., Verdugo-Rodriguez, A., Rodriguez, L.L., and Borca, M.V. (2019). The Role of Interleukin 6 During Viral Infections. Front. Microbiol. *10*, 1057. https://doi.org/10.3389/fmicb.2019.01057.
57. Eisenhauer, E.A., Therasse, P., Bogaerts, J., Schwartz, L.H., Sargent, D., Ford, R., Dancey, J., Arbuck, S., Gwyther, S., Mooney, M., et al. (2009).

New response evaluation criteria in solid tumours: revised RECIST guideline (version 1.1). Eur. J. Cancer *45*, 228–247. https://doi.org/10.1016/j.ejca.2008.10.026.

58. Ritchie, M.E., Phipson, B., Wu, D., Hu, Y., Law, C.W., Shi, W., and Smyth, G.K. (2015). limma powers differential expression analyses for RNA-sequencing and microarray studies. Nucleic Acids Res. *43*, e47. https://doi.org/10.1093/nar/gkv007.

59. Gu, Z. (2022). Complex heatmap visualization. Imeta *1*, e43. https://doi.org/10.1002/imt2.43.

60. Xie, Z., Bailey, A., Kuleshov, M.V., Clarke, D.J.B., Evangelista, J.E., Jenkins, S.L., Lachmann, A., Wojciechowicz, M.L., Kropiwnicki, E., Jagodnik, K.M., et al. (2021). Gene Set Knowledge Discovery with Enrichr. Curr. Protoc. *1*, e90. https://doi.org/10.1002/cpz1.90.

STAR★METHODS

KEY RESOURCES TABLE

REAGENT or RESOURCE	SOURCE	IDENTIFIER
Antibodies		
APC anti-human/mouse Granzyme B Recombinant Antibody (clone QA16A02)	Biolegend	Cat.#372204<br>RRID: AB_2687027
Alexa Fluor™ 700 anti-mouse FOXP3 Monoclonal Antibody (clone FJK-16s)	eBioscience	Cat#56-5773-80<br>RRID: AB_1210557
BD Pharmingen™ PerCP-Cy™5.5 Rat Anti-Mouse CD4 (clone RM4-5)	BD Biosciences	Cat#550954<br>RRID: AB_393977
BD Pharmingen FITC Rat Anti-Mouse CD45 (clone 30-F11)	BD Biosciences	Cat#553080<br>RRID: AB_394609
BD Pharmingen PE Rat Anti-Mouse CD38 (clone 90)	BD Biosciences	Cat#553764<br>RRID: AB_395034
PE/Cyanine5 anti-mouse NK-1.1 (clone PK136)	Biolegend	Cat#108716<br>RRID: AB_493590
PE-Cyanine7 anti-mouse TCR beta Monoclonal Antibody (clone H57-597)	eBioscience	Cat#25-5961-82<br>RRID: AB_2573507
BD Horizon™ BV421 Rat Anti-Mouse CD8a (clone 53-6.7)	BD Biosciences	Cat#563898<br>RRID: AB_2738474
BD Horizon BV510 Rat Anti-Mouse CD44 (clone IM7)	BD Biosciences	Cat#563114<br>RRID: AB_2738011
BD Horizon BV605 Rat Anti-Mouse CD19 (clone 1D3)	BD Biosciences	Cat#563148<br>RRID: AB_2732057
BD Horizon BV605 Rat Anti-Mouse LY-6G (clone 1A8)	BD Biosciences	Cat#563005<br>RRID: AB_2737946
BD Horizon BV650 Rat Anti-Mouse CD62L (clone MEL-14A)	BD Biosciences	Cat#564108<br>RRID: AB_2738597
BD Horizon BV711 Rat Anti-Mouse CD279 (PD-1) (clone 29F.1A12)	BD Biosciences	Cat#568563<br>RRID: Not available
BV786 Rat Anti-Mouse Ki-67 Monoclonal Antibody (clone SolA15)	eBioscience	Cat#417-5698-82<br>RRID: AB_10854564
BD Horizon Viability stain FVS620	BD Biosciences	Cat#564996<br>RRID: AB_2869636
BD Pharmingen FITC Mouse Anti-Human HLA-DR (clone TU36)	BD Biosciences	Cat#555560<br>RRID: AB_395942
BD Pharmingen PE Mouse Anti-Human CD83 (clone HB15e)	BD Biosciences	Cat#550634<br>RRID: N/A
PerCP/Cyanine5.5 anti-human CD27 Antibody (clone O323)	Biolegend	Cat#302819<br>RRID: AB_893295
BD Pharmingen™ PE-Cy™7 Mouse Anti-Human CD86 (clone 2331)	BD Biosciences	Cat#561128<br>RRID: AB_10563077
BD Pharmingen™ APC Mouse Anti-Human CD54 (clone HA58)	BD Biosciences	Cat#561899<br>RRID: AB_398667
BD™ APC-H7 Mouse Anti-Human CD38 (clone HB7)	BD Biosciences	Cat#656646<br>RRID:N/A
BD Horizon™ BV421 Mouse Anti-Human IgD (clone IA6-2)	BD Biosciences	Cat#6565940<br>RRID: AB_11153121
BD Horizon™ BV605 Mouse Anti-Human CD45 (clone HI30)	BD Biosciences	Cat#6564047<br>RRID: AB_2744403
BD Horizon™ BV786 Mouse Anti-Human CD19 (clone SJ25C1)	BD Biosciences	Cat#563326<br>RRID: AB_2744314

(Continued on next page)

CellPress
OPEN ACCESS

Continued

REAGENT or RESOURCE	SOURCE	IDENTIFIER
BD Pharmingen™ PerCP-Cy™5.5 Mouse Anti-Human CD16 (clone 3G8)	BD Biosciences	Cat#560717<br>RRID: AB_1727434
BD Pharmingen™ APC-H7 Mouse anti-Human CD14 (clone MφP9)	BD Biosciences	Cat#560180<br>RRID: AB_1645464
BD Horizon™ BV421 Mouse Anti-Human CD11c (clone B-ly6)	BD Biosciences	Cat#562561<br>RRID: AB_2737656
BD Horizon™ BV510 Mouse Anti-Human CD3 (clone UCHT1)	BD Biosciences	Cat#563109<br>RRID: AB_2732053
Brilliant Violet 510™ anti-human CD19 Antibody (clone HIB19)	Biolegend	Cat#302242<br>RRID: AB_2561668
BD Horizon™ BV510 Mouse Anti-Human CD56 (clone NCAM16.2)	BD Biosciences	Cat#563041<br>RRID: AB_2732786
BD Horizon™ BV711 Mouse Anti-Human CD123 (clone 9F5)	BD Biosciences	Cat#563161<br>RRID: AB_2738038
Brilliant Violet 785™ anti-human CD141 (Thrombomodulin) Antibody (clone M80)	Biolegend	Cat#344115<br>RRID: AB_2572195
BD™ CD45RA FITC (clone L48)	BD Biosciences	Cat#335039<br>RRID: N/A
BD™ CD56 FITC (clone NCAM16.2)	BD Biosciences	Cat#345811<br>RRID: N/A
BD™ PE Mouse Anti-Human CD38 (clone HB7)	BD Biosciences	Cat#347687<br>RRID: AB_400341
CD3 Monoclonal Antibody (SK7), PerCP-Cyanine5.5, eBioscience™ (clone SK7)	eBioscience	Cat#45-0036-42<br>RRID: AB_1518742
BD™ PE-Cy™7 Mouse Anti-Human CD8 (clone SK1)	BD Biosciences	Cat#335787<br>RRID: N/A
BD Pharmingen™ Alexa Fluor® 647 Mouse anti-Human CD279 (PD-1) (clone EH12.1)	BD Biosciences	Cat#560838<br>RRID: AB_2033988
APC/Fire™ 750 anti-human CD197 (CCR7) Antibody (clone G043H7)	Biolegend	Cat#353245<br>RRID: AB_2750146
BD Horizon™ BV421 Mouse Anti-Ki-67 (clone B56)	BD Biosciences	Cat#562899<br>RRID: AB_2686897
BD Horizon™ V500 Mouse Anti-Human CD4 (clone RPA-T4)	BD Biosciences	Cat#560768<br>RRID: AB_1937323
BD Horizon™ BV605 Mouse Anti-Human CD45 (clone HI30)	BD Biosciences	Cat#564048<br>RRID: AB_2744403
**Biological samples**		
Tumor biopsies	OPTIMIZE-1 study (NCT04888312)	Not available due to data protection laws to ensure confidentiality of patient information
Whole blood samples	OPTIMIZE-1 study (NCT04888312)	Not available due to data protection laws to ensure confidentiality of patient information
**Chemicals, peptides, and recombinant proteins**		
FACS ™ lysing solution	BD Biosciences	Cat#349202<br>RRID: AB_2868862
FACS Permeabilizing Solution 2	BD Biosciences	Cat#340973<br>RRID: NA
**Critical commercial assays**		
AllPrep DNA/RNA FFPE Kit – Nucleic Acid Extraction	Qiagen	Cat#80234<br>RRID: NA
Qubit™ dsDNA HS Assay Kit	Invitrogen	Cat#Q32854<br>RRID: NA

(*Continued on next page*)

Continued		
REAGENT or RESOURCE	SOURCE	IDENTIFIER
TruSight Oncology 500 DNA Automation Kit	Illumina	Cat#20045504<br>RRID: NA
Agilent DNA 1000 Kit	Agilent	Cat#5067-1504<br>RRID: NA
QIAamp Circulating Nucleic Acid Kit	Qiagen	Cat#55114<br>RRID: NA
QX100/QX200 ddPCR	Bio-Rad	RRID: SCR_019714
Eukaryote Total RNA Nano Series II Assay	Agilent	RRID: NA
KAPA RNA HyperPrep Kit with RiboErase (HMR) Globin	Roche	08308241702<br>RRID: NA
Pre-validated multiplex MSD cytokine and chemokine assays (proinflammatory panel 1 and chemokine panel 1)	Mesoscale Diagnostics	RRID: NA
Custom-made validated FC panels	Cerba Research	RRID: NA
2 x 100 bp S2 kit flow cell	Illumina	RRID: NA
Deposited data		
RNA sequencing data	Gene Expression Omnibus	GEO: GSE291331
Experimental models: Cell lines		
KPCY tumor cells (clone 2838c, KRAS^{G12D}, Trp53^{R172H})	Kerafast	RRID:CVCL_YM18
Experimental models: Organisms/strains		
Human CD40 transgenic mouse	In-house (Alligator Bioscience AB)	N/A
Software and algorithms		
QuantaSoft^{TM} Software (Regulatory Edition 1864011)	Bio-Rad	RRID: NA
FACSLyric^{TM}	BD Biosciences	RRID: NA
FACSuite^{TM} software (Version 1.4)	BD Biosciences	RRID: NA
Illumina's BCL Convert (Version 3.10)	Illumina	RRID: NA
bcl2fastq2 Conversion Software (Version 2.20)	Illumina	RRID: SCR_015058
R (Version 4.3.0)	R development core team	RRID: SCR_001905
OncoKB^{TM} data package[34,35]	Memorial Sloan Kettering Cancer Center	RRID: SCR_014782
TSO500 Local App (Version 2.2)	Illumina	RRID: NA
DRAGEN MIS pipeline (Version TBC)	Illumina	RRID: NA
DRAGEN RNA pipeline (Version 4.0)	Illumina	RRID: NA
Ensembl Variant Effect Predictor (Release TBC)	Ensembl	RRID: SCR_007931
survminer package (Version 0.4.9)	Rdocumentation	RRID: SCR_021094
survival package (Version 3.5–5)	Rdocumentation	RRID: SCR_026244
BaseSpace Sequence Hub	Illumina	RRID: SCR_011881
PCA tools package (Version 2.14.0)	Biooconductor	RRID: SCR_025593
Limma (Version 3.58.1)	Biooconductor	RRID: SCR_010943
ComplexHeatmap package (Version 2.18.0)	Biooconductor	RRID: SCR_017270
DESeq2 (Version 1.42.1)	Biooconductor	RRID: SCR_015687
GO_Biological_Process_2018 database (Version 3.2)	Gene Ontology	RRID: SCR_002811
Immuno-Oncology Biological Research (IOBR) package (Version 0.99.9)	GitHub	RRID: SCR_025619

(*Continued on next page*)

CellPress
OPEN ACCESS

Continued

REAGENT or RESOURCE	SOURCE	IDENTIFIER
Other		
Covaris E220evolution focused-ultrasonicator	Covaris	500429<br>RRID: SCR_019817
Agilent 2100 Bioanalyzer	Agilent	RRID: SCR_018043
NovaSeq 6000 instrument	Illumina	RRID: SCR_016387
Qubit®4 Fluorometer	Thermo Fisher Scientific	Cat#Q33226<br>RRID: SCR_018095

EXPERIMENTAL MODELS AND STUDY PARTICIPANT DETAILS

Human participants

Eligible patients were aged 18 years or older with a histologically confirmed diagnosis of previously untreated mPDAC, an Eastern Cooperative Oncology Group performance status of 0 or 1 and measurable disease according to modified RECIST version 1.1.[57] All patients or their representatives provided written informed consent before enrollment. OPTIMIZE-1 is an open-label, phase 1b/2 study being conducted in 14 centers across Europe in accordance with the study protocol and amendments and was approved by the local Institutional Review Boards and independent ethics committees at each of the participating centers. The study is being conducted in accordance with the Declaration of Helsinki and Good Clinical Practice guidelines as defined by the International Conference on Harmonisation.

METHOD DETAILS

Study design

Details of study design, patients and treatments in OPTIMIZE-1 have been reported previously.[16] The primary outcome of phase 1b was determination of the RP2D of mitazalimab in combination with mFOLFIRINOX by examining the incidence of dose-limiting toxicities (DLTs). The primary outcome of phase 2 was the ORR of mitazalimab in combination with mFOLFIRINOX, defined as the proportion of patients achieving a confirmed CR or PR as per the RECIST v1.1 guideline.[57] Key secondary objectives applicable to both phases of the study included assessment of efficacy and other clinical outcomes. BoR was defined as the best response (CR, PR, SD, and PD) observed at any time during the study. DoR was defined as the number of days from the initial response (CR or PR) to progressive disease or death due to underlying disease, whichever occurred first, and was assessed in all patients who achieved a confirmed response. Duration of SD was defined as the number of days from first dose of mitazalimab to progressive disease or death, whichever occurred first. DCR was defined as the proportion of patients achieving CR, PR or SD et each visit. Time to next anti-cancer therapy was defined as the number of days from the first dose of mitazalimab to initiation of subsequent treatment. Safety, and tolerability was evaluated by recording the type, frequency. and severity of AEs, which were coded using MedDRA version 24.0, and graded according to the National Cancer Institute Common Terminology Criteria for Adverse Events (version 5.0). Survival outcomes, including PFS and OS, were also assessed.

OPTIMIZE-1: Patient inclusion and exclusion

Patients were required to meet several hematological and clinical chemistry laboratory criteria. Key exclusion criteria included other types of non-ductal tumor of the pancreas (including endocrine tumors or acinar cell adenocarcinoma, cyst adenocarcinoma and ampullary carcinoma), other current cancer or history of cancer (other than *in situ* cervical cancer or basal cell or squamous cell carcinoma treated with local excision only) in the three years prior to study enrollment and known central nervous system metastases or carcinomatous meningitis. The complete eligibility criteria, including exclusion criteria specific to mFOLFIRINOX treatment, are provided in Data S1/Methods S1. Patients fulfilling all eligibility criteria were registered onto the study using an Interactive Web Response System automated patient registration system. Patient registration was controlled by accrual limits assigned to the active cohort.

OPTIMIZE-1: Patient treatment

In phase 1b, mitazalimab was administered intravenously (IV) during a 2-h rate-controlled infusion at 450 μg/kg and 900 μg/kg, in combination with mFOLFIRINOX (85 mg/m^2 oxaliplatin IV over two hours, 400 mg/m^2 leucovorin over two hours, 150 mg/m^2 irinotecan over 90 min [starting 30 min after the start of the leucovorin infusion] followed by 2400 mg/m^2 5-FU over 46–48 h) to determine the RP2D. During the first 21-day treatment cycle, mitazalimab was administered as a priming dose on Study Days 1 (priming dose) and 10 and mFOLFIRINOX infusion was started on Study Day 8. During subsequent 14-day treatment cycles, mFOLFIRINOX was administered on Study Day 1 and mitazalimab on Study Day 3 of each cycle.

Dose escalation in phase 1b followed a Bayesian optimal interval design requiring at least three patients evaluable for a DLT at each dose level. The DLT evaluation period was defined as the time from the first dose of mitazalimab (Study Day 1) until Day 21 in the first treatment cycle (Cycle 1). Detailed descriptions of DLT criteria can be found in Data S1/Methods S1. A minimum of six patients were required to be evaluated at the RP2D. When the last patient had completed the DLT evaluation period in phase 1b, collected data were reviewed by a Data Review Committee, and a decision was made on continuation into phase 2 of the study.

The RP2D of mitazalimab, in combination with mFOLFIRINOX, was administered to all patients in the phase 2 study, with a planned treatment duration of up to 12 cycles. Upon completion of all 12 treatment cycles, at the Investigator's discretion, a patient deriving clinical benefit (without confirmed PD) was permitted to continue until PD, unacceptable toxicity, withdrawal of consent or until any other treatment discontinuation or study withdrawal criteria are met, whichever came first.

OPTIMIZE-1: Efficacy assessments

Anti-tumor activity was evaluated by assessing CT scan of chest/abdomen/pelvis and other body areas, if required, according to local practice with consistent imaging used throughout per patient. Scans were performed according to RECIST v1.1 guidelines at screening and every four cycles thereafter. Additional CT scans were permitted at the Investigator's discretion.

OPTIMIZE-1: Safety assessments

Any AEs considered by Investigators as related to mFOLFIRINOX permitted a dose adjustment according to standard practice for mFOLFIRINOX. The dose of mitazalimab was reduced by 50% where an AE possibly, probably, or definitely related to mitazalimab resulted in more than two weeks of treatment delay. Only one dose reduction was allowed. Any AE considered serious or >Grade 2 and possibly, probably, or definitely related to mitazalimab resulted in mitazalimab being withheld until resolution of the AE to the greater of the baseline grade or $\leq$ Grade 1, or less. In such cases mitazalimab was permitted to be resumed at the Investigator's discretion either at the previous dose or, if not already dose-reduced, a 50% dose reduction; the Investigator was also permitted to forgo rechallenge. Study treatment was discontinued if there was evidence of PD, unacceptable toxicity, withdrawal of consent or meeting other criteria for study drug termination.

Sample collection and handling

Tumor biopsies

Tumor biopsies were collected from patients in the FAS of OPTIMIZE-1 (patients that received 900 μg/kg of mitazalimab for two or more cycles), ITT set (all patients who received 900 μg/kg dose of mitazalimab), and safety set (all patients who received any study treatment, including 450 μg/kg of mitazalimab) at baseline and C2D10 or at another unscheduled time point if not collected at Cycle 2. Samples were collected using an 18-gauge needle (inner diameter = 0.838 mm, inner surface = 0.55 mm^2). After collection, tumor biopsies underwent formalin fixation followed by paraffin embedding (FFPE) and were either stored as FFPE blocks or FFPE tissue slides.

Subsequently, one slide from each patient was subjected to H&E staining, followed by pathologist evaluation. FFPE samples that had a tumor cell content below 20%, or detectable necrotic or healthy adjacent tissue, were subjected to macrodissection to enrich for tumor cell areas ensuring tumor purity during downstream procedures and analyses. No macrodissection was performed when tumor cell islets were dispersed, or the tumor area was less than 5mm^2.

Whole blood samples

Whole blood samples were collected longitudinally according to the schedule of assessments) in the OPTIMIZE-1 study protocol (Data S1/Methods S1). Streck tubes were used for ct*KRAS* subtype analysis and tumor burden assessment, while ethylenediamine tetraacetic acid (EDTA) tubes were used for immunophenotyping.

Genomic profiling of tumor biopsies

DNA extraction, library preparation and sequencing

Total DNA was extracted from FFPE slides using the AllPrep DNA/RNA FFPE Kit (50) (Qiagen, Hilden, Germany, Cat# 80234). Extracted DNA was quantified with the Qubit dsDNA HS assay (Invitrogen, Massachusetts, USA, Cat# Q32854). DNA samples meeting the input requirement (manufacturer-recommended input of 40ng, with very low DNA yield adjusted to 14 ng) were fragmented with the Covaris E220evolution focused-ultrasonicator (Covaris, Massachusetts, USA, Cat#500429, RRID: SCR_019817). Mechanical fragmentation was followed by 3′ and 5′ end repair. Samples were then used as templates for cDNA library generation using the TruSight Oncology 500 (TSO500) High-Throughput DNA Library Preparation Kit (Illumina, California, USA, Cat# 20045504). Library concentration was determined using the Qubit dsDNA HS Assay Kit; size distribution and average fragment length were evaluated using the Agilent DNA 1000 assay (Agilent, California, USA, Cat# 5067-1504) on the Agilent 2100 Bioanalyzer (Agilent, RRID: SCR_018043). Libraries were sequenced on a NovaSeq 6000 instrument using the 2 x 100 bp S2 kit flow cell (Illumina) to achieve a mean target coverage of $\geq$150x.

Sample filtering of TSO500 dataset

The TSO500 dataset derivation can be found in Figure S2. Following QC, 37 samples were retained for demographic analysis. From these, 35 baseline samples with RECIST response data were used for associations with RECIST response. Finally, 32 baseline samples from the FAS with survival data were tested for association with clinical variables. Three patients with unscheduled visits

according to eCRF records were recategorized by comparing the first mitazalimab dose date and the sample collection date. Details of these analyses can be found in the quantification and statistical analysis section.

Transcriptome profiling of tumor biopsies

RNA extraction, library preparation and sequencing

Total RNA was extracted from FFPE slides using the AllPrep DNA/RNA FFPE Kit (50). RNA QC was performed via electrophoresis using Eukaryote Total RNA Nano Series II assay in a 2100 Bioanalyzer instrument. RNA samples meeting the size range requirements (40 ng input RNA) were subjected to library preparation using the KAPA RNA HyperPrep Kit with RiboErase (HMR) Globin (Roche, Basel, Switzerland, Cat# 08308241702). Derived cDNA library concentration was determined using the Qubit dsDNA HS Assay Kit; size distribution and average fragment length, and RIN value were evaluated on Agilent 2100 bioanalyzer. cDNA libraries were divided into two batches based on a 240bp library size threshold. Libraries were sequenced in a NovaSeq 6000 instrument using NovaSeq 6000 S2 (Batch 1) and S1 (Batch 2) flow cells at the desired sequencing depth (50 M reads per library, 2 X 100 bp paired end reads). Three samples were sequenced in both batches to ensure proper batch normalization in the event that sequencing batch contributed to variance in the dataset.

Sample filtering of RNA-seq dataset

As for the TSO500 dataset derivation, the 40 unique sequenced samples, the remaining 40 samples were subjected to gene and sample filtering (see Figure S3). Gene counts >10 in 20 samples were considered detected, and four samples with <15000 (<75%) detected genes were excluded from analysis, yielding 36 QC'd samples (Figure S3). Of note, the four excluded samples featured either low library concentration or low insert fragment size. Three patients with unscheduled visits according to eCRF records were recategorized by comparing the first mitazalimab dose date and the sample collection date.

Circulating KRAS subtype analysis from blood samples

The ct*KRAS* G12 subtype analysis on blood samples was performed by Saga Diagnostics (Lund, Sweden), developed to detect the three most frequent G12 alleles in PDAC; *KRAS* G12V/D/R variants. DNA was extracted from plasma from whole blood using the QIAamp Circulating Nucleic Acid Kit (Qiagen, Cat# 5511). Following extraction, DNA concentrations were measured using a Qubit 4 Fluorometer (Thermo Fisher Scientific, Massachusetts, USA, Cat# Q33226, RRID: SCR_018095) and results were generated using the QX100/QX200 ddPCR (Bio-Rad Laboratories, Veenendaal, the Netherlands, RRID: SCR_019714), and analyzed using the QuantaSoft Software that accompanies the QX100/QX200 systems.

A clinical sample was considered *KRAS* G12V/D/R mutant positive if the measured MAF was higher than the determined limit of blank of the assay and if there were at least two mutant positive droplets across the test reactions. Sample results that fail either criterion may still be called mutant positive on a case-by-case basis with strong supporting evidence and expert interpretation.

Cytokine and chemokine assessment

Cytokine and chemokine levels in serum were analyzed at Cerba Research using Mesoscale Discovery (MSD; Mesoscale Diagnostics, Maryland, USA) and pre-validated multiplex MSD cytokine and chemokine assays (proinflammatory panel 1 and chemokine panel 1) which included the following cytokines and chemokines: IL-8, IL-10, IL-12p70, TNF-α, IFN-γ, MCP-1, IP-10, MIP-1 alpha and MIP-1 beta.

Immunophenotyping assessment

Immunophenotyping and activation status were analyzed in whole blood samples using custom-made validated flow cytometry panels at Cerba Research. Details of the immunophenotypes in both the APC and T/NK cell panels can be found in Table S13.

Sample preparation and FC analysis (using custom-made validated FC panels at Cerba Research) were conducted according to validated assay protocols within 72 h after collection of whole blood samples. All incubation steps were carried out at room temperature and in the dark. Whole blood was lysed with FACS lysing solution (BD Biosciences, Cat# 349202, RRID: AB_2868862). For stainings, 100 μL of anti-coagulated whole blood was transferred to each assay tube and incubated with the appropriate antibody cocktail mixture. For intracellular stainings, after the surface staining procedure samples were centrifuged, the supernatant was discarded, and the cells were resuspended in the FACS Permeabilizing Solution 2 (BD Biosciences, Cat# 340973). Samples were then incubated with the intracellular staining antibody cocktail. After washing and resuspension all samples were analyzed within 1 h after sample preparation. Data acquisition was done using FACSLyric (BD Biosciences, New Jersey, USA). Analysis of the raw data was performed with FACSuite software, version 1.4.

QUANTIFICATION AND STATISTICAL ANALYSIS

Clinical data analysis

The study employed a Simon's two stage design to statistically evaluate whether the addition of mitazalimab to mFOLFIRINOX improved the ORR compared to a historical control rate of 30% (ORR with chemotherapy alone). The formal hypotheses were.

(1) Null hypothesis (H_0): ORR ≤30% (no improvement over historical control)
(2) Alternative hypothesis (H_1): ORR >30% (superior to historical control)

For sample size estimation, a target ORR of 45% was assumed to reflect the anticipated improvement with mitazalimab. The study was powered at 80% with a one-side type I error rate of 5%. The safety set (n = 70) includes all patients who received any dose of mitazalimab. The ITT set (n = 65) includes all patients who received a dose of 900 μg/kg mitazalimab. The FAS (n = 57) is all patients who received the combination of mitazalimab at the RP2D (900 μg/kg) and mFOLFIRINOX for at least two treatment cycles.

Full details of the statistical analyses of the clinical data have been reported previously and are described in Data S1/Methods S1.

Median DoR, PFS, and OS were estimated using Kaplan-Meier methods with 95% CIs. ORR is accompanied by one-sided 90% CIs. No formal statistical hypothesis was defined for phase 1b; patients were pooled with those on the same dose regimen in phase 2 for statistical analyses and data summaries.

Analysis of genomic profiling of tumor biopsies

TSO500 data analysis

Raw sequencing data (binary base call [BCL] files) were stored in BaseSpace Sequence Hub, demultiplexed, and converted to FASTQ files using bcl2fastq2 Conversion Software (Illumina, RRID: SCR_015058). The FASTQ files were transferred to the TruSight Oncology 500 Local App (Illumina) for analysis. The sequences in the FASTQ files were aligned to the GRCh37 (hg19) reference genome using the Isaac Aligner, generating Binary Alignment Map (BAM) files. BAM files were then processed by the TSO500 Local App's DNA Analysis Methods. Specifically, copy number variant (CNV) calling utilized the CRAFT algorithm, and structural variant detection employed the Manta algorithm. TMB was calculated by counting all coding SNVs and indels with variant allele frequency ≥5% and variant allele count ≥3. MSI status was determined using 130 microsatellite loci. All analyses were performed using default parameters as recommended by Illumina for the TSO500 assay.

Demographic analyses

Data analysis and visualization were performed in R (version 4.3.0, RRID: SCR_001905), running in a virtual environment from docker. Genes were classified into oncogene or tumor suppressor gene based on their annotation using the "oncoKBdata" package (https://github.com/waldronlab/oncoKBData).[34,35] The frequency of the top 25 altered genes was compared to current literature.[23,24,31–33]

Small variant mutation

The small variant mutation dataset was generated from single combined variant output files (per sample). Individual variants were annotated using the Ensembl Variant Effect Predictor software to gain further information on the variants such as predicted functional effects and allele frequency in various populations. To remove potential germline mutations, the data were subsequently filtered to exclude variants with MAF >0.01 in any of the 1000 Genomes, exome sequencing project (ESP) or Genome Aggregation Database (gnomAD) populations. Finally, only somatic variants with strong functional consequences (Ensembl IMPACT rating [Ensembl IMPACT rating] of moderate or high) were selected. In contrast, analysis of DNA damage repair genes *BRCA1*, *BRCA2* and *PALB2* was performed by excluding variants with MAF <0.01 in any of the 1000 Genomes, ESP or gnomAD populations. Finally, only germline variants with strong functional consequences (Ensembl IMPACT rating high) were selected.

Copy number variation

CNV data, where copy number losses and gains were identified, were extracted from the CNV call format (VCF) files. Only CNVs that passed the quality assessment (FILTER = PASS) were used in the statistical analyses. Only copy number gains were retained.

Tumor mutational burden

Samples were classified as having a high or low TMB based on their median TMB value (2.65 mutations/Mb). No distinction was performed between synonymous and non-synonymous mutations.

Microsatellite instability status

MSI status data were obtained from the TSO500 Local App's pipeline. The percentage of unstable MSI sites to total assessed MSI sites is reported as a sample-level microsatellite score. Samples were categorized into MS-stable (MSS; <20%) and MSI (≥20%) based on the DRAGEN pipeline recommendations (Microsatellite Instability [illumina.com]) for solid samples. All samples with usable MSI sites were MS-stable; and thus, no statistical analysis could be performed based on microsatellite status.

Associations of genomic alterations in tumor biopsies with clinical variables

Associations of genomic alterations to clinical variables were performed by FIOS genomics (Edinburgh, UK).

Association with categorical clinical variables (OS rate at 12 months, PFS rate at 12 months, ORR, DC)

The presence of a specific variant, gene mutation (as a binary variable), and a gene CNV (as a binary variable) were assessed for association with OS-12, PFS-12, ORR and disease control using Fisher's exact test. OS-12 segregated patients into still alive after 12 months (post-12 months) or had died before or at 12 months (pre-12 months). PFS-12 segregated the patients into progression-free after 12 months (post-12 months) or showed PD before or at 12 months (pre-12 months). TMB status was assessed for association with OS-12, PFS-12, ORR and disease control using a t-test.

Association with continuous clinical variables (overall survival, progression-free survival, duration of response)

The presence of a specific variant, gene mutation (as a binary variable), and a gene CNV (as a binary variable) were assessed for association with each of the three survival variables, OS ('SURV 0S'), PFS ('SURV PFS') and duration of confirmed response ('SURV CRSD') using univariate Cox PH regression. Genes altered in at least three samples were included in the statistical analyses.

TMB status was assessed for association with OS ('SURV 0S'), PFS ('SURV PFS') and duration of confirmed response ('SURV CRSD') using univariate Cox PH regression.

Association of circulating KRAS with continuous clinical variables

OS and PFS differences between ct*KRAS* G12 subtypes were tested using both the log rank and Gehan-Breslow tests.

Correlation between circulating KRAS and tumor volume

Data analysis and visualization were performed in-house using R (version 4.3.0). Raw data from the ct*KRAS* subtype analysis assay was retrieved. The %MAF was defined as the fraction of *KRAS* G12V/D/R concentration divided by the total concentration, expressed as a percentage.

KRAS G12 V/D/R % MAF information from blood ctDNA was used in the analyses. The three *KRAS* G12 Wt patients, and the eight *KRAS* undetermined patients were filtered out for subsequent analyses. The %MAF was standardized on a per patient basis, using longitudinal on-treatment %MAF divided by the %MAF at baseline. Similarly, tumor volume as indicated by SLD was standardized on a per patient basis, using longitudinal on-treatment SLD divided by the SLD at baseline, expressed as a percentage. Standardized and %SLD was matched to the closest %MAF measure for each patient (10.67 days difference on average ±6.03 days). Correlation between %MAF and %SLD was visualized using a scatterplot and statistically tested using Pearson's correlation test. Standardized data from patients with three or more datapoints were used to generate patient-specific exponential models. These models were used to calculate appropriate mResponse and mProgression thresholds, equivalent to those used in RECIST based on conventional CT scan tumor measures. The thresholds were further validated using the "surv_cutpoint" function of the survminer package (version 0.4.9., RRID: SCR_021094).

Association of circulating KRAS, circulating KRAS clearance, mResponse, mProgression with clinical efficacy endpoints

Patients carrying a *KRAS* G12 V/D/R mutation with available baseline ct*KRAS* data were included in the associations between ct*KRAS* baseline levels and clinical efficacy endpoints. From this set, only patients with two or more on-treatment ct*KRAS* datapoints, including at baseline and at least one on-treatment datapoint, were included in the associations between ct*KRAS* clearance, mResponse, or mProgression, and clinical efficacy endpoints. The threshold for ct*KRAS* clearance was defined as the mean of MAF from *KRAS* G12 Wt patients plus three standard deviations. To define mResponse, reductions in %MAF from baseline were analyzed for correlation with clinical response. Previously described exponential models were used to estimate a %MAF threshold equivalent to the conventional 30% reduction in %SLD, as defined by RECIST v.1.1[57] (Figure 5B). A reduction of 30% in SLD translated into a reduction of 82.5% (±21.0) in %MAF. Thus, mResponse was defined as the reduction of at least 90% from baseline in ct*KRAS* % MAF within the first 70 days of treatment.

Survival was visualized using the KM method and reported as median with 95% CI. OS between groups was compared using a log rank test using the survival package (version 3.5–5, RRID: SCR_026244). Assessment of the impact of continuous variables on survival parameters was performed via cox PH using the survival package.

Analysis of transcriptome profiling of tumor biopsies

RNA-seq data analysis

Raw sequencing data (BCL files) were stored in BaseSpace, demultiplexed and converted to FASTQ files (Illumina's BCL Convert version 3.10.10). The FASTQ files were transferred to BaseSpace Sequence Hub (Illumina, RRID: SCR_011881) for further analysis. After aligning FASTQ files to the GRCh37 (hg19) reference genome, quantification of reference genes and transcripts were performed using the DRAGEN RNA pipeline (Illumina version 4.0).

Data analysis and visualization were performed in R (version 4.3.0).

Confounding factor analysis for RNA seq

RNA-seq was performed in two batches and a batch-effect analysis was performed. Clinical and technical covariates in metadata were evaluated in confounding factor analysis. Pairs of numerical variables were evaluated by Pearson correlation. Pairs of categorical covariates were evaluated by either Chi-square or Fisher's exact test (depending on number of samples per group). Pairs of categorical and numerical covariates were evaluated using analysis of variance (ANOVA). P-value was adjusted using the false discovery rate (FDR) method (see Figure S8A). Contribution of clinical and technical covariates to PC variance was performed using the "eigencorplot" function from the PCA tools package (version 2.14.0) (Figure S8B).

Given that sequencing batch did not contribute to variance in the first 10 PCs, the three re-sequenced samples were addressed. Thus, for samples that were sequenced twice, batch 1 counterparts were removed, while batch 2 counterparts were kept (Figure S3). Technical covariates that were significantly associated with the first two PCs and not confounded with clinical efficacy endpoints (namely "hospital", "Biopsy_site", "Macrodissection", "Sample type", "HE_AREA") were tested for regression using "removebatcheffect" function in Limma (version 3.58.1, Bioconductor, RRID: SCR_010943).[58] Using VST counts, the regression of "Biopsy_site" and "Hospital" removed the effect of the technical covariates while maximizing the associations of clinical efficacy endpoints to the first 10 PCs (Figure S8B).

Molecular subtype classification (classical vs. basal-like)

Thirty-three (33) unique QC'd samples (from 31 patients; Figure S3) were subjected to molecular classification into classical and basal-like subtypes using previously published gene-sets.[20,21] Briefly, VST counts of genes in each gene set were subjected to *Z* score transformation and PCA visualization. Next, samples were clustered via K-means clustering in the PCA space and visualized in heatmap format using the ComplexHeatmap package (version 2.18.0, Bioconductor, RRID: SCR_017270).[59]

Differential gene expression and pathway enrichment analyses

Exploratory data analysis using clinical efficacy endpoints was performed only in the FAS, which includes samples from the efficacy cohort (n = 27, including 21 baseline samples and six on-treatment samples). Filtered gene counts were subjected to normalization (via median of ratios method) and DGEA (accounting for batch effects in covariates Hospital and Biopsy_site) using DESeq2 (version 1.42.1, Bioconductor, RRID: SCR_015687) with the normal shrinkage estimator. P-values were adjusted using FDR (BH correction). DEGs were visualized using "EnhancedVolcano" package (version 1.20.0; https://github.com/kevinblighe/EnhancedVolcano, Bioconductor, RRID: SCR_018931). DEGs, defined by p-value <0.05 and absolute log2 FC >1, were extracted, and used for pathway enrichment analysis employing the enrichR package to query the GO_Biological_Process_2018 database (version 3.2, RRID: SCR_002811).[60] Pathways were considered enriched if their adjusted p-value threshold was <0.05.

Gene set scoring

Filtered transcripts per million (TPM) counts were normalized via a linear model for microarray data (LIMMA) package using the clinical variable under study, and accounting for the batch effects of "Hospital" and "Biopsy_site" covariates. Normalized TPM counts were then used for TME deconvolution using the function "deconvo_tme" from the Immuno-Oncology Biological Research (IOBR) package (version 0.99.9, GitHub, RRID: SCR_025619), either using the MCP-counter or EPIC methods. Deconvolved cell scores were then statistically compared between groups. First, distribution of the data was evaluated by Shapiro-Wilk test, and differences between groups were tested using either t-test or Wilcoxon rank-sum test depending on normality. Gene signatures were evaluated via the "calculate_sig_score" function in the IOBR package using the ssGSEA method. In both cases, p-values were adjusted using the BH adjustment.

Data analysis of immune cells and cytokines

Filtered FC data and cytokine data were used for data analysis of immune cells and cytokines. Levels of the main cell populations depicted in this report, along with all cytokines except IL-12p70, were selected for further analysis, inclusive of C1D1 through C2D1. For a given cell population or cytokine, levels at a given timepoint were compared to the rest of timepoints, through either an unpaired t-test or unpaired Mann Whitney test, depending on the distribution of the data. Multiple comparisons were adjusted using the FDR method.

Additional resources

This study is registered with ClinicalTrials.gov (NCT04888312).

Cell Reports Medicine, Volume *6*

Supplemental information

CD40 agonist mitazalimab with mFOLFIRINOX in untreated metastatic pancreatic cancer: Biomarkers associated with outcomes from OPTIMIZE-1

Jean-Luc Van Laethem, Karen Geboes, Ivan Borbath, Teresa Macarulla Mercade, Aurélien Lambert, Philippe Cassier, Hans Prenen, Emmanuel Mitry, Jean-Frédéric Blanc, Lorenzo Pilla, Jaime Feliu, Mercedes Rodriguez Garrote, Roberto Antonio Pazo-Cid, Inmaculada Gallego, Karin Enell Smith, Karin Nordbladh, David Gomez Jimenez, Peter Ellmark, Yago Pico de Coaña, Sumeet Vijay Ambarkhane, Gregory L. Beatty, and Eileen M. O'Reilly

Supplemental Information

Table of Contents

Supplementary Table 1: Baseline Demographics and Clinical Characteristics of Patients included in OPTIMIZE-1 2
Supplementary Table 2: Subsequent anti-cancer therapies 4
Supplementary Table 3: Efficacy responses by Investigator assessment per RECIST v1·1 5
Supplementary Table 4: Overview of TEAEs 6
Supplementary Table 5: Overview of TEAEs of ≥Grade 3 by SOC (PT) 7
Supplementary Table 6: Genomic alterations associated with clinical efficacy endpoints 9
Supplementary Table 7: Genomic alterations measured using TSO500 10
Supplementary Table 8: KRAS G12 variant consensus dataset. 12
Supplementary Table 9: Enriched pathways and gene signatures and associated survival benefits 14
Supplementary Table 10: Metrics of the mResponse predictor 15
Supplementary Table 11: Metrics of the mProgression predictor 15
Supplementary Table 12: Subgroup analysis of clinical efficacy outcomes 16
Supplementary Table 13: Immunophenotypes of APC and T/NK cell populations 17

Supplementary Figure 1: *Trial Profile*. Related to *Figure 1 in the manuscript* 18
Supplementary Figure 2: TSO500 dataset derivation. Related to Figure 2 in the manuscript 19
Supplementary Figure 3: RNA-sequencing dataset derivation. Related to Figure 3 and 4 in the manuscript. . 20
Supplementary Figure 4: Supplementary information related to transcriptomic profiles of OPTIMIZE-1 patients. Related to Figure 3 in the manuscript 21
Supplementary Figure 5: Supplementary information on tumor extrinsic factors associated with clinical efficacy endpoints, during the first cycle of OPTIMIZE-1 supporting Figure 4 in the manuscript. 22
Supplementary Figure 6: ctKRAS dataset derivation. Related to Figure 5 in the manuscript. 24
Supplementary Figure 7: Supplementary information related to correlation of ctDNA with tumor burden and association of clinical. Related to Figure 5 in the manuscript 25
Supplementary Figure 8: Confounding co-variate analyses. Related to "Confounding factor analysis for RNA seq" section in the STAR methods 26

Data S1/Methods S1:Study Protocol 28

Supplementary Table 1: *Baseline Demographics and Clinical Characteristics of Patients included in OPTIMIZE-1*

	**FAS (N=57)**	**Safety set (N=70)**
**Age, years**		
Median (range)	62 (43-77)	62·0 (43-77)
Under 65	35 (61·4%)	40 (57·1%)
65 or above	22 (38·6%)	30 (42·9%)
**Sex***		
Female	33 (57·9%)	40 (57·1%)
Male	24 (42·1%)	30 (42·9%)
**Race***		
American Indian or Alaska Native	0 (0%)	0 (0%)
Asian	0 (0%)	0 (0%)
Black or African American	3 (5·3%)	3 (4·3%)
Native Hawaiian or Other Pacific Islander	0 (0%)	0 (0%)
Not reported	16 (28·1%)	19 (27·1%)
Other	1 (1·8%)	1 (1·4%)
Unknown	0 (0%)	0 (0%)
White	37 (64·9%)	47 (67·1%)
**Ethnicity***		
Unknown	0 (0%)	0 (0%)
Hispanic or Latino	3 (5·3%)	3 (4·3%)
Not Hispanic or Latino	35 (61·4%)	41 (58·6%)
Not reported	19 (33·3%)	26 (37·1%)
**Time from diagnosis to treatment start, days; median (IQR)**	29·5 (21·75-35)	29·0 (21–35)
**ECOG performance status**		
Grade 0	31 (54·4%)	38 (54·3%)
Grade 1	26 (45·6%)	32 (45·7%)
**Modified Glasgow Prognostic Score**		
0	29 (50·9%)	35 (50·0%)
1	25 (43·9%)	31 (44·3%)
2	3 (5·3%)	4 (5·7%)
**Prior surgery**		
Yes	2[†] (3·5%)	3 (4·3%)
No	55 (96·5%)	67 (95·7%)
**CA19-9 at baseline (n)**	48	61
<100	8 (16·7%)	9 (14·8%)
100-1000	13 (27·1%)	13 (21·3%)
>1000	27 (56·3%)	39 (63·9%)
**Neutrophil/lymphocyte ratio (n)**	56	69
<5	46 (82·1%)	57 (82·6%)
>5	10 (17·9%)	12 (17·4%)
**Primary tumor location (n)**	55	67
Head	25 (45·5%)	29 (43·3%)
Body	21 (38·2%)	23 (34·3%)
Tail	9 (16·4%)	15 (22·4%)
**Number of target lesions**		
1	12 (21·1%)	12 (17·1%)
2	19 (33·3%)	21 (30·0%)
3 or more	25 (45·6%)	37 (52·9%)
**Presence of liver metastases**		
Yes	42 (73·7%)	55 (78·6%)
No	15 (26·3%)	15 (21·4%)
**Sites of metastatic disease**		

Liver	42 (73·7%)	55 (78·6%)
Lung	9 (15·8%)	12 (17·1%)
Peritoneal	9 (15·8%)	10 (14·3%)
Lymph Node	10 (17·5%)	14 (20·0%)
Other	16 (28·1%)	20 (28·6%)
**Number of liver metastasis** (n)	42	55
1	7 (16·7%)	8 (14·5%)
2	14 (33·3%)	18 (32·7%)
3 or more	21 (50·0%)	29 (52·7%)
**Histological subtype**		
Adenocarcinoma	56 (98·2%)	68 (97·1%)
Adenosquamous carcinoma	0 (0%)	1 (1·4%)
Not known	1 (1·8%)	1 (1·4%)

*Data are based on the full analysis set (FAS) and safety set populations. Data are n (%) except where specified. *Information on sex, race, and ethnicity was collected via electronic medical records. †Choleduodenostomy and palliative excision of the primary tumor, respectively. CA19-9, carbohydrate antigen 19-9; ECOG, Eastern Cooperative Oncology Group; IQR, interquartile range; n, number of patients with non-missing value; N, number of patients in FAS or safety set population.*

Supplementary Table 2: *Subsequent anti-cancer therapies*

**Gemcitabine-based**	**5-FU-based**	**Liposomal irinotecan-based**	**Other**
Gemcitabine	FOLFIRI	Liposomal irinotecan + leucovorin + 5-FU	Investigational drug CM24 + nivolumab + paclitaxel + gemcitabine
Gemcitabine-nab paclitaxel	FOLFOX	Liposomal irinotecan; 5-FU	Paclitaxel
	FOLFIRINOX		Olaparib
	5-FU+ leucovorin; cisplatin		Bevacizumab
	5-FU + leucovorin; oxaliplatin		Dabrafenib + trametinib

5-FU, 5-fluorouracil.

Supplementary Table 3: *Efficacy responses by Investigator assessment per RECIST v1·1*

**Investigator-assessed efficacy outcomes**	**Efficacy set (N=57)**	**ITT (N=65)**
**Objective response**		
Patients with objective response (unconfirmed)	31	31
ORR (unconfirmed)*, % (90% CI)	54.4 (42.7 - 65.7)	47.7 (37.0 - 58.6)
Patients with objective response (confirmed)	24	24
ORR (confirmed)*, % (90% CI)	42.1 (31.0 - 53.9)	36.9 (26.9 - 47.8)
**Best Overall Response, n (%)**		
CR	1 (1.8)	1 (1.5)
PR	23 (40.4)	23 (35.4)
SD	21 (36.8)	21 (32.3)
PD	12 (21.1)	16 (28.1)
DCR	45 (78.9)	45 (69.2)
Not Evaluated	0	4 (6.2)
**Median DoR, months (95% CI)**	12.6 (7.5 - NE)	12.6 (7.5 - NE)
**PFS**		
Median PFS, months (95% CI)	7.7 (5.8 - 11.3)	7.7 (5.8 - 11.1)
6-month PFS rate, % (95% CI)	60.9 (46.9 - 72.3)	58.5 (47.5 - 72.1)
12-month PFS rate, % (95% CI)	35.1 (22.8 - 47.6)	33.4 (23.5 - 47.7)
18-month PFS rate, % (95% CI)	22.2 (13.1 - 37.5)	19.9 (11.6 - 34.0)
**OS**		
Median OS, months (95% CI)	14.9 (10.0 - 17.3)	14.0 (9.2 - 15.9)
6-month OS rate, % (95% CI)	89.5 (81.9 - 97.8)	84.4 (75.9 - 93.8)
12-month OS rate, % (95% CI)	57.8 (43.9 - 69.4)	54.6 (43.7 - 68.3)
18-month OS rate, % (95% CI)	36.2 (23.0 - 49.5)	33.2% (22.7 - 48.5)

**Complete or partial responses may only be claimed if the criteria for each are confirmed by a repeat assessment at least four weeks later. BOR, best overall response; CI, confidence interval; CR, complete response; DCR, disease control rate; DoR, duration of response; ITT, intent-to-treat;, NE, not evaluable; ORR, objective response rate; OS, overall survival; PD, progressive disease; PFS, progression-free survival; PR, partial response; RECIST, Response Evaluation Criteria in Solid Tumors; SD, stable disease.*

Supplementary Table 4: *Overview of TEAEs*

Total number (%) of patients with;	Mitazalimab (450mg/kg) (N=5)	Mitazalimab (900 mg/kg) (N=65)	Safety set (N=70)
any TEAE	5 (100.0)	64 (98.5)	69 (98.6)
any TEAE related to mitazalimab	4 (80.0)	57 (87.7)	61 (87.1)
any TEAE related to mFOLFIRINOX	5 (100.0)	61 (93.8)	
any TEAE of ≥Grade 3	3 (60.0)	52 (80.0)	55 (78.6)
any TEAE leading to discontinuation of study treatment	1 (20.0)	4 (6.2)	5 (7.1)
any SAE	2 (40.0)	28 (43.1)	30 (42.9)
any SAE leading to death	0 (0.0)	0 (0.0)	0 (0.0)
any SAE related to mitazalimab	0 (0.0)	8 (12.3)	8 (11.4)
any SAE related to mFOLFIRINOX	0 (0.0)	15 (23.1)	15 (21.4)
any AESI, overall	0 (0.0)	20 (30.8)	20 (28.6)
or AESI by category:			
IRR Grade 2 or higher	0 (0.0)	13 (20.0)	13 (18.6)
CRS Grade 2 or higher	0 (0.0)	0 (0.0)	0 (0.0)
AST and/or ALT elevation >5 x ULN	0 (0.0)	5 (7.7)	5 (7.1)
Bilirubin elevation >1.5 x ULN	0 (0.0)	3 (4.6)	3 (4.3)

Adverse events (AEs) were collected from signing of informed consent until the last administration of any study treatment. Treatment-emergent is defined as occurring or worsening after the first infusion of mitazalimab and up to 28 days after end of any study treatment. AESI, adverse event of special interest; ALT, alanine aminotransferase; AST, aspartate aminotransferase; CRS, cytokine release syndrome; IRR, infusion-related reaction; mFOLFIRINOX, modified FOLFIRINOX; N, number of patients; SAE, serious adverse event; TEAE, treatment-emergent adverse event; ULN, upper limit of normal.

Supplementary Table 5: *Overview of TEAEs of ≥Grade 3 by SOC (PT)*

**Total number (%) of patients with;**	**Mitazalimab (450mg/kg) (N=5)**	**Mitazalimab (900 mg/kg) (N=65)**	**Safety set (N=70)**
**Any TEAE ≥Grade 3 by SOC (PT)***			
**Blood and lymphatic system disorder**	0 (0.0)	29 (44.6)	29 (41.4)
Anaemia	0 (0.0)	9 (13.8)	9 (12.9)
Febrile Neutropenia	0 (0.0)	1 (1.5)	1 (1.4)
Neutropenia	0 (0.0)	18 (27.7)	18 (25.7)
Thrombocytopenia	0 (0.0)	8 (12.3)	8 (11.4)
**Cardiac Disorders**	0 (0.0)	2 (3.1)	2 (2.9)
Pericardial effusion	0 (0.0)	1 (1.5)	1 (1.4)
Supraventricular tachycardia	0 (0.0)	1 (1.5)	1 (1.4)
**Gastrointestinal disorders**	1 (20.0)	18 (27.7)	19 (27.1)
Abdominal pain	1 (20.0)	2 (3.1)	3 (4.3)
Abdominal pain upper	0 (0.0)	1 (1.5)	1 (1.4)
Ascites	1 (20.0)	0 (0.0)	1 (1.4)
Diarrhoea	0 (0.0)	6 (9.2)	6 (8.6)
Duodenal obstruction	0 (0.0)	1 (1.5)	1 (1.4)
Dysphagia	1 (20.0)	1 (1.5)	2 (2.9)
Gastrointestinal motility disorder	0 (0.0)	1 (1.5)	1 (1.4)
Haemorrhoids	0 (0.0)	1 (1.5)	1 (1.4)
Nausea	1 (20.0)	3 (4.6)	4 (5.7)
Obstruction gastric	0 (0.0)	2 (3.1)	2 (2.9)
Small intestinal obstruction	0 (0.0)	1 (1.5)	1 (1.4)
Stomatitis	0 (0.0)	1 (1.5)	1 (1.4)
Vomiting	0 (0.0)	3 (4.6)	3 (4.3)
**General disorders and administration site conditions**	1 (20.0)	10 (15.4)	11 (15.7)
Asthenia	1 (20.0)	3 (4.6)	4 (5.7)
Condition aggravated	1 (20.0)	1 (1.5)	2 (2.9)
Fatigue	0 (0.0)	7 (10.8)	7 (10.0)
**Hepatobilliary disorders**	0 (0.0)	3 (4.6)	3 (4.3)
Biliary obstruction	0 (0.0)	0 (0.0)	0 (0.0)
Cholangitis	0 (0.0)	3 (4.6)	3 (4.3)
**Infection and infestations**	0 (0.0)	11 (16.9)	11 (15.7)
Abdominal infection	0 (0.0)	2 (3.1)	2 (2.9)
Balanoposthitis infective	0 (0.0)	1 (1.5)	1 (1.4)
Bronchitis	0 (0.0)	1 (1.5)	1 (1.4)
COVID-19	0 (0.0)	1 (1.5)	1 (1.4)
Device-related infection	0 (0.0)	2 (3.1)	2 (2.9)
Oral fungal infection	0 (0.0)	1 (1.5)	1 (1.4)
Pneumonia	0 (0.0)	2 (3.1)	2 (2.9)
Urinary tract infection	0 (0.0)	1 (1.5)	1 (1.4)
**Injury, poisoning and procedural complications**	1 (20.0)	1 (1.5)	2 (2.9)
Fall	1 (20.0)	0 (0.0)	1 (1.4)
Femoral neck fracture	0 (0.0)	1 (1.5)	1 (1.4)
**Investigations**	0 (0.0)	6 (9.2)	6 (8.6)
Alanine aminotransferase increased	0 (0.0)	2 (3.1)	2 (2.9)
Alanine aminotransferase increased	0 (0.0)	2 (3.1)	2 (2.9)
Gamma-glutamyltransferase increased	0 (0.0)	1 (1.5)	1 (1.4)
Lipase increased	0 (0.0)	1 (1.5)	1 (1.4)
Platelet count decreased	0 (0.0)	1 (1.5)	1 (1.4)
Weight decreased	0 (0.0)	1 (1.5)	1 (1.4)
**Metabolism and nutrition disorders**	2 (40.0)	16 (24.6)	18 (25.7)
Decreased appetite	1 (20.0)	1 (1.5)	2 (2.9)
Hypoalbuminaemia	1 (20.0)	1 (1.5)	2 (2.9)
Hypokalaemia	1 (20.0)	10 (15.4)	11 (15.7)

Hypomagnesaemia	0 (0.0)	2 (3.1)	2 (2.9)
Hyponatraemia	0 (0.0)	1 (1.5)	1 (1.4)
Malnutrition	1 (20.0)	2 (3.1)	3 (4.3)
**Musculoskeletal and connective tissue disorders**	0 (0.0)	1 (1.5)	1 (1.4)
Back pain	0 (0.0)	1 (1.5)	1 (1.4)
**Neoplasms benign, malignant and unspecified (including cysts and polyps)**	1 (20.0)	0 (0.0)	1 (1.4)
Non-small cell lung cancer	1 (20.0)	0 (0.0)	1 (1.4)
**Nervous system disorders**	1 (20.0)	11 (16.9)	12 (17.1)
Headache	0 (0.0)	1 (1.5)	1 (1.4)
Peripheral neuropathy	1 (20.0)	5 (7.7)	6 (8.6)
Peripheral sensory neuropathy	0 (0.0)	1 (1.5)	1 (1.4)
Polyneuropathy	0 (0.0)	3 (4.6)	3 (4.3)
Syncope	0 (0.0)	1 (1.5)	1 (1.4)
**Psychiatric disorders**	0 (0.0)	2 (3.1)	2 (2.9)
Depression	0 (0.0)	1 (1.5)	1 (1.4)
Suicide attempt	0 (0.0)	1 (1.5)	1 (1.4)
**Renal and urinary disorders**	0 (0.0)	1 (1.5)	1 (1.4)
Acute kidney injury	0 (0.0)	1 (1.5)	1 (1.4)
**Respiratory, thoracic and mediastinal disorders**	0 (0.0)	2 (3.1)	2 (2.9)
Pleural effusion	0 (0.0)	1 (1.5)	1 (1.4)
Pulmonary embolism	0 (0.0)	2 (3.1)	2 (2.9)

*Adverse events (AEs) were collected from signing of informed consent until the last administration of any study treatment. Treatment-emergent is defined as occurring or worsening after the first infusion of mitazalimab and up to 28 days after end of any study treatment. MedDRA, Medical Dictionary for Regulatory Activities; N, number of patients; PT, preferred term; SOC, system organ class; TEAE, treatment-emergent adverse event; v, version. *PT coded with MedDRA v24·0.*

Supplementary Table 6: *Genomic alterations associated with clinical efficacy endpoints*

Gene ID	Symbol	OR	HR	Effect	P-value	Adjusted p-value	Clinical parameter	Genetic alteration	Variant ID
**ENSG00000133703**	*KRAS*	0.134	N/A	-2.897	0.01	0.24	OR	Gene variant	chr12:25398284_C/A
**ENSG00000138376**	*BARD1*	0.166		-2.592	0.04	0.42	DoR	Gene variant	chr2:215632255_CA/TG
**ENSG00000134899**	*ERCC5*	N/A	6.203	2.633	0	0.26	OS	Gene mutant	N/A
**ENSG00000171940**	*ZNF217*		4.632	2.212	0.02	0.74	OS	Gene mutant	
**ENSG00000134899**	*ERCC5*		5.644	2.497	0.01	0.34	PFS	Gene mutant	
**ENSG00000153201**	*RANBP2*		4.789	2.26	0.01	0.34	PFS	Gene mutant	
**ENSG00000197299**	*BLM*		5.45	2.446	0.02	0.34	PFS	Gene mutant	
**ENSG00000065526**	*SPEN*		0.216	-2.208	0.04	0.69	PFS	Gene mutant	
**ENSG00000134899**	*ERCC5*	11.907	N/A	3.574	0.01	0.92	OS12	Gene mutant	
**ENSG00000065526**	*SPEN*	0.098		-3.355	0.01	0.43	PFS12	Gene mutant	
**ENSG00000096968**	*JAK2*	0.048		-4.368	0.02	0.66	OR	Gene mutant	
**ENSG00000065526**	*SPEN*	0.097		-3.368	0.03	0.66	OR	Gene mutant	
**ENSG00000162344**	*FGF19*	N/A	3.591	1.844	0.03	0.10	OS	CNV gain	
**ENSG00000162344**	*FGF19*		6.736	2.752	0	0.01	PFS	CNV gain	
**ENSG00000075388**	*FGF4*		4.775	2.256	0.02	0.03	PFS	CNV gain	
**ENSG00000110092**	*CCND1*		4.775	2.256	0.02	0.03	PFS	CNV gain	
**ENSG00000162344**	*FGF19*	11.332	N/A	3.502	0.04	0.20	DC	CNV gain	

CNV, copy number variant; DC, disease control; DoR, duration of response; HR, hazard ratio; ID, identification; N/A, not applicable; OR, overall response; OS, overall survival; OS12, overall survival at 12 months; PFS, progression-free survival; PFS12, progression-free survival at 12 months.

Supplementary Table 7: Genomic alterations measured using TSO500

ABL1	BMPR1A	CSF1R	ERCC5	FLI1	HIST1H3I	KDR	MRE11A	PAX3	PTCH1	SDHD	TCF7L2
ABL2	BRAF	CSF3R	ERG	FLT1	HIST1H3J	KEAP1	MSH2	PAX5	PTEN	SETBP1	TERC
ACVR1	BRCA1	CSNK1A1	ERRFI1	FLT3	HIST2H3A*	KEL	MSH3	PAX7	PTPN11	SETD2	TERT
ACVR1B	BRCA2	CTCF	ESR1	FLT4	HIST2H3C	KIF5B	MSH6	PAX8	PTPRD	SF3B1	TET1
AKT1	BRD4	CTLA4	ETS1	FOXA1	HIST2H3D	KIT	MST1*	PBRM1	PTPRS	SH2B3	TET2
AKT2	BRIP1	CTNNA1	ETV1	FOXL2	HIST3H3	KLF4	MST1R	PDCD1	PTPRT	SH2D1A	TFE3
AKT3	BTG1	CTNNB1	ETV4	FOXO1	HLA-A	KLHL6	MTOR	PDCD1LG2	QKI	SHQ1	TFRC
ALK	BTK	CUL3	ETV5	FOXP1	HLA-B	KMT2B	MUTYH	PDGFRA	RAB35	SLIT2	TGFBR1
ALOX12B	C11orf30	CUX1	ETV6	FRS2	HLA-C	KMT2C	MYB*	PDGFRB	RAC1	SLX4	TGFBR2
ANKRD11	CALR	CXCR4	EWSR1	FUBP1	HNF1A	KMT2D	MYC	PDK1	RAD21	SMAD2	TMEM127
ANKRD26	CARD11	CYLD	EZH2	FYN	HNRNPK	KRAS	MYCL1	PDPK1*	RAD50	SMAD3	TMPRSS2
APC	CASP8	DAXX	FAM123B	GABRA6	HOXB13	LAMP1	MYCN	PGR	RAD51	SMAD4	TNFAIP3
AR	CBFB	DCUN1D1	FAM175A	GATA1	HRAS	LATS1	MYD88	PHF6	RAD51B	SMARCA4	TNFRSF14
ARAF	CBL	DDR2	FAM46C	GATA2	HSD3B1	LATS2	MYOD1	PHOX2B	RAD51C	SMARCB1	TOP1
ARFRP1	CCND1	DDX41	FANCA	GATA3	HSP90AA1	LMO1	NAB2	PIK3C2B	RAD51D	SMARCD1	TOP2A
ARID1A	CCND2	DHX15	FANCC	GATA4	ICOSLG*	LRP1B	NBN	PIK3C2G	RAD52	SMC1A	TP53
ARID1B	CCND3	DICER1	FANCD2	GATA6	ID3	LYN	NCOA3	PIK3C3	RAD54L	SMC3	TP63
ARID2	CCNE1	DIS3	FANCE	GEN1	IDH1	LZTR1	NCOR1*	PIK3CA	RAF1	SMO*	TRAF2
ARID5B	CD274	DNAJB1	FANCF	GID4	IDH2	MAGI2*	NEGR1	PIK3CB	RANBP2	SNCAIP	TRAF7
ASXL1	CD276	DNMT1	FANCG	GLI1	IFNGR1	MALT1	NF1*	PIK3CD	RARA	SOCS1	TSC1
ASXL2	CD74	DNMT3A	FANCI	GNA11	IGF1	MAP2K1*	NF2	PIK3CG	RASA1	SOX10	TSC2
ATM	CD79A	DNMT3B	FANCL	GNA13	IGF1R	MAP2K2	NFE2L2	PIK3R1	RB1	SOX17	TSHR
ATR	CD79B	DOT1L	FAS	GNAQ*	IGF2	MAP2K4	NFKBIA	PIK3R2	RBM10	SOX2	U2AF1
ATRX	CDC73	E2F3	FAT1	GNAS*	IKBKE	MAP3K1	NKX2-1	PIK3R3	RECQL4	SOX9	VEGFA
AURKA	CDH1	EED	FBXW7	GPR124	IKZF1	MAP3K13	NKX3-1	PIM1	REL	SPEN	VHL
AURKB	CDK12	EGFL7	FGF1	GPS2	IL10	MAP3K14	NOTCH1	PLCG2	RET	SPOP	VTCN1
AXIN1	CDK4	EGFR	FGF10	GREM1	IL7R	MAP3K4	NOTCH2*	PLK2	RFWD2	SPTA1	WISP3
AXIN2	CDK6	EIF1AX	FGF14	GRIN2A	INHA	MAPK1	NOTCH3*	PMAIP1	RHEB	SRC	WT1
AXL	CDK8	EIF4A2	FGF19	GRM3	INHBA	MAPK3	NOTCH4	PMS1	RHOA	SRSF2	XIAP

B2M	CDKN1A	EIF4E	FGF2*	GSK3B	INPP4A	MAX	NPM1*	PMS2	RICTOR	STAG1	XPO1
BAP1	CDKN1B	EML4	FGF23	H3F3A	INPP4B	MCL1	NRAS	PNRC1	RIT1	STAG2	XRCC2
BARD1	CDKN2A	EP300	FGF3	H3F3B	INSR	MDC1	NRG1	POLD1	RNF43	STAT3	YAP1
BBC3	CDKN2B	EPCAM	FGF4	H3F3C	IRF2	MDM2	NSD1	POLE	ROS1	STAT4	YES1
BCL10	CDKN2C	EPHA3	FGF5	HGF	IRF4	MDM4	NTRK1	PPARG	RPS6KA4	STAT5A*	ZBTB2
BCL2	CEBPA*	EPHA5	FGF6	HIST1H1C	IRS1	MED12	NTRK2	PPM1D	RPS6KB1	STAT5B*	ZBTB7A
BCL2L1	CENPA	EPHA7	FGF7*	HIST1H2BD	IRS2	MEF2B	NTRK3	PPP2R1A	RPS6KB2	STK11	ZFHX3
BCL2L11*	CHD2	EPHB1	FGF8	HIST1H3A	JAK1	MEN1	NUP93	PPP2R2A	RPTOR	STK40	ZNF217
BCL2L2	CHD4	ERBB2	FGF9	HIST1H3B	JAK2	MET	NUTM1	PPP6C	RUNX1	SUFU	ZNF703
BCL6	CHEK1	ERBB3	FGFR1	HIST1H3C	JAK3	MGA	PAK1	PRDM1	RUNX1T1	SUZ12*	ZRSR2
BCOR	CHEK2	ERBB4	FGFR2*	HIST1H3D	JUN	MITF	PAK3	PREX2	RYBP	SYK	
BCORL1	CIC	ERCC1	FGFR3	HIST1H3E	KAT6A	MLH1	PAK7	PRKAR1A	SDHA	TAF1	
BCR	CREBBP	ERCC2	FGFR4	HIST1H3F	KDM5A	MLL	PALB2	PRKCI	SDHAF2	TBX3	
BIRC3	CRKL	ERCC3	FH	HIST1H3G	KDM5C	MLLT3*	PARK2	PRKDC	SDHB	TCEB1	
BLM	CRLF2	ERCC4	FLCN	HIST1H3H	KDM6A	MPL	PARP1	PRSS8	SDHC*	TCF3	

Derived fromhttps://www.illumina.com/content/dam/illumina/gcs/assembled-assets/marketing-literature/trusight-oncology-500-data-sheet-m-gl-00173/trusight-oncology-500-and-ht-data-sheet-m-gl-00173.pdf

Supplementary Table 8: *KRAS G12 variant consensus dataset.*

**Tumor *KRAS* status (n=32)**	**ct*KRAS* status (n=49)**	**Consensus *KRAS* status (n=50/N=57)**
	p.G12R	p.G12R
	p.G12V	p.G12V
p.G12V	p.G12V	p.G12V
	G12 - Wt	G12 - Wt
	p.G12D	p.G12D
p.G12V	p.G12V	p.G12V
	p.G12D	p.G12D
p.G12V	p.G12V	p.G12V
	p.G12R	p.G12R
	G12 - Wt	G12 - Wt
p.G12V	p.G12V	p.G12V
p.G12D	p.G12D	p.G12D
p.G12V	p.G12V	p.G12V
	p.G12D	p.G12D
	p.G12V	p.G12V
p.G12V	p.G12V	p.G12V
	p.G12V	p.G12V
	Not detectable *KRAS*	Not detectable *KRAS*
p.G12V	p.G12V	p.G12V
p.G12V	p.G12V	p.G12V
	p.G12V	p.G12V
p.G12V	p.G12V	p.G12V
	p.G12D	p.G12D
p.G12R	p.G12R	p.G12R
p.G12D	p.G12D	p.G12D
p.G12D	p.G12D	p.G12D
	p.G12D	p.G12D
p.G12V	Not detectable *KRAS*	p.G12V
p.G12D	p.G12D	p.G12D
p.G12D	p.G12D	p.G12D
p.G12I	p.G12D	Unmatched
	p.G12D	p.G12D
	p.G12V	p.G12V
p.G12R	Not detectable *KRAS*	p.G12R
p.G12V	p.G12D(/V) multiallelic	Unmatched
p.G12D	p.G12V(/D) multiallelic	Unmatched
p.G12R	Not detectable *KRAS*	p.G12R
	p.G12V	p.G12V
p.G12D	p.G12D	p.G12D
	p.G12D	p.G12D
	Not detectable *KRAS*	Not detectable *KRAS*
p.G12V	p.G12V	p.G12V

p.G12D	p.G12D	p.G12D
p.G12V	p.G12V	p.G12V
p.G12V	p.G12V	p.G12V
p.G12V	p.G12V	p.G12V
	Not detectable *KRAS*	Not detectable *KRAS*
p.G12R	p.G12R	p.G12R
	Not detectable *KRAS*	Not detectable *KRAS*
	p.G12D	p.G12D
p.G12D	p.G12D	p.G12D
p.G12D	Not detectable *KRAS*	p.G12D
p.G12D	p.G12D	p.G12D
	p.G12R	p.G12R
	p.G12V	p.G12V
	G12 - Wt	G12 - Wt
	p.G12D	p.G12D

The tumor KRAS dataset includes somatic variant with an allele frequency <0.01 according to gnomAD, and a sequencing depth >50 counts. The ctKRAS dataset, derived from blood, includes G12 variant information with a mutant allele frequency (MAF) higher than the determined limit of blank (LoB) of the assay and at least two mutant positive droplets across the test reactions. In the consensus KRAS dataset, three patients were categorized as "unmatched": two multiallelic patients carried both G12V and D mutations (minor allele indicated in brackets), and one patient presented a disagreement between ctKRAS and tumor KRAS datasets. Only patients from the full analysis set (FAS) set were included. N, number of patients with detectable G12 KRAS; N, number of patients in the FAS; wt, wild type.

Supplementary Table 9: *Enriched pathways and gene signatures and associated survival benefits*

Clinical Endpoint	Patient Response	Gene Expression Profile	Enriched Pathway
**OS-12**	Long survivor (>12 months)	*S100A4*, *IL6*	Immune modulatory pathway
		ITGA8, *LRRN2*	Cell adhesion
		MMP2, *MMP3*, *MMP9*, *COL15A1*, *COL10A1*	Extracellular matrix remodeling
	Short survivors (<12 months)	*CYP2A6, CYP3A7, CYP3A5, CYP2D6, CYP2C9*	Drug and steroid metabolic processes (cytochrome P450 pathway)
		ABCG5, *ABCB11*	Drug and steroid metabolic processes (lipid and drug pump)
		UGT1A4, *UGT1A7*, *UGT1A1*	Drug and steroid metabolic processes (drug inactivating enzymes)
**PFS-12**	Late progressors (>12 months)	*CXCL17*, *IL6*, *OLFM4*, *SERPINB3*, *CD40*	General inflammatory response
		CCL22, *CCL19*, *CCL20*, *CCL2*	Monocyte chemotaxis
		C1QB, *C1QA* and *IGHV* transcripts	Complement activation
	Early progressors (<12 months)	*CYP2A7*, *CYP2A6*, *CYP2D6*, *UGT1A7*	Coumarin metabolism
		APOA2, *APOA1*, *APOC3*	Lipid metabolic processes
**OR/DC**	Responded to treatment/controlled the disease	*COL1A1, COL11A1, COL3A1, MMP2, MMP9 and MMP11*	ECM remodeling, disassembly, and collagen fibril organization
	Responders	*CXCL13, SIGLEC6*	Immune responses
		CD79A, IGHD	B-cell biology
	Non-responders (including quick progressors)	*CYP2A6, CYP3A7, CYP3A5, CYP2D6, CYP2C9*	Drug and steroid metabolic processes (cytochrome P450 pathway)

DC, disease control; ECM, extracellular matrix; OR, overall response; OS-12, overall survival at 12 months; PFS12, progression-free survival at 12 months.

Supplementary Table 10: *Metrics of the mResponse predictor*

		RECIST v1.1		
		Responders	Non responders	Total
Molecular Response	Responders	16	4	20
	Non responders	6	17	23
	Total	22	21	43

Supplementary Table 11: *Metrics of the mProgression predictor*

		RECIST v1.1		
		Progressors	Non Progressors	Total
Molecular Progression	Progressors	10	12	21
	Non progressors	4	17	21
	Total	14	29	43

Supplementary Table 12: *Subgroup analysis of clinical efficacy outcomes*

**Subgroup**		**ORR (95 %CI)**	**PFS (95 %CI)**	**OS (95 %CI)**
**Gender**	Female (n=33)	42.4 (27.8 - 58.1)	7.4 (5.5 - 10.8)	14.3 (9.0 - 15.9)
	Male (n=24)	41.7 (24.6 - 60.3)	11.3 (5.7 - 14.4)	15.0 (8.8 - NE)
**Presence of liver metastasis (baseline)**	Yes (n=42)	42.9 (29.8 - 56.7)	7.1 (5.1 - 9.2)	13.6 (9.2 - 17.3)
	No (n=15)	40.0 (19.1- 64)	13.1 (5.6 - NE)	15.6 (7.8 - NE)

CI, confidence interval; NE, not evaluable; ORR, objective response rate; OS, overall survival; PFS, progression-free survival.

Supplementary Table 13: *Immunophenotypes of APC and T/NK cell populations*

**APC panel**	
**Population**	**Immunophenotype**
B cells	CD45+CD19+
Naïve B cells	CD45+CD19+IgD+CD27-
Class switched memory B cells	CD45+CD19+IgD-CD27+
Plasmablasts	CD45+CD19+IgD-CD27+CD38++
Non switched memory B cells	CD45+CD19+IgD+CD27+
Double neg B cells	CD45+CD19+IgD-CD27-
Monocytes	CD45+Lin-CD14low-highCD16low-med
Classical monocytes	CD45+Lin-CD14+CD16-
Intermediate monocytes	CD45+Lin-CD14+CD16+
Non-classical monocytes	CD45+Lin-CD14dimCD16+
pDC	CD45+Lin-CD14-CD16-HLA-DR+CD123+CD11c-
mDC	CD45+Lin-CD14-CD16-HLA-DR+CD123-CD11c+
cDC1	CD45+Lin-CD14-CD16-HLA-DR+CD123-CD11c+CD141+
**T/NK cell panel**	
**Population**	**Immunophenotype**
T cells	CD45+CD3+
CD8 T cells	CD45+CD3+CD8+CD4-
Naïve CD8 T cells	CD45+CD3+CD8+CD4-CD45RA+CCR7+
CD8+ TCM cells	CD45+CD3+CD8+CD4-CD45RA-CCR7+
CD8+ TEM cells	CD45+CD3+CD8+CD4-CD45RA-CCR7-
CD8+ TEMRA cells	CD45+CD3+CD8+CD4-CD45RA+CCR7-
CD4 T cells	CD45+CD3+CD8-CD4+
Naïve CD4 T cells	CD45+CD3+CD8-CD4+CD45RA+CCR7+
CD4+ TCM cells	CD45+CD3+CD8-CD4+CD45RA-CCR7+
CD4+ TEM cells	CD45+CD3+CD8-CD4+CD45RA-CCR7-
CD4+ TEMRA cells	CD45+CD3+CD8-CD4+CD45RA+CCR7-
DN T cells	CD45+CD3+CD8-CD4-
DP T cells	CD45+CD3+CD8+CD4+
NKT cells	CD45+CD3+CD56+
NK cells	CD45+CD3-CD56+
CD56bright CD16- NK cells	CD45+CD3-CD56++CD16-
CD56dimCD16+ NK cells	CD45+CD3-CD56+CD16+

APC, antigen presenting cell; CD, cluster of differentiation; cDC, conventional type 1 dendritic cell; DN, double negative; DP, double positive; HLA, human leukocyte antigens; Ig, immunoglobulin; Lin, lineage marker negative; mDC, myeloid dendritic cell; NK, natural killer; pDC, plasmacytoid dendritic cell; TCM, central memory T cells; TEM, effector memory T cells; TEMRA, terminally differentiated effector memory T cells.

(A)

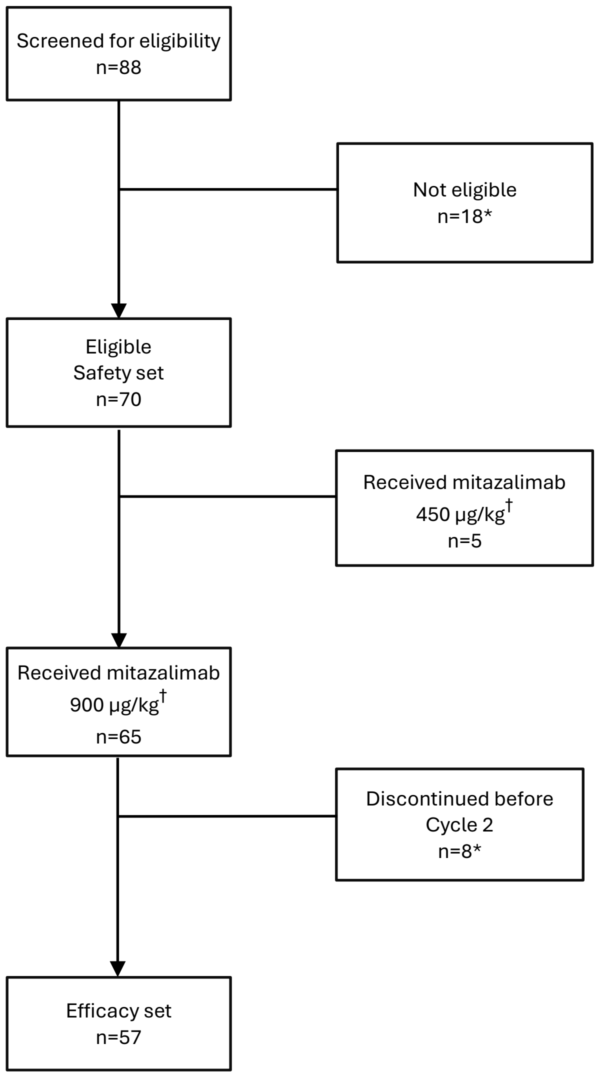

(B)

Subject No.	Reason for ineligibility
1	Unacceptable clinical chemistry laboratory values
2	Unwilling to comply with all study procedures
3	Unacceptable clinical chemistry laboratory values
4	QTc >450 msec
5	Platelets ≥100 x 10^9/L; intercurrent illness
6	Has complete dihydropyrimidine dehydrogenase deficiency
7	Unacceptable clinical chemistry laboratory values
8	Positive pregnancy test
9	Unacceptable clinical chemistry laboratory values
10	Unacceptable clinical chemistry laboratory values
11	Unacceptable clinical chemistry laboratory values
12	Contraindications to study treatment
13	Unacceptable clinical chemistry laboratory values
14	Unwilling to comply with all study procedures
15	Has complete dihydropyrimidine dehydrogenase deficiency
16	Patient died due to disease progression before treatment start
17	ECOG >1; life expectancy <3 months, known CNS metastases
18	Contraindications to study treatment

(C)

Subject No.	Days on treatment	Reason for discontinuation
1	10	Unacceptable toxicity (DLT, headache)
2	1	Administrative/PI decision
3	7	Progressive disease
4	8	Consent withdrawn
5	10	Progressive disease
6	10	Clinical deterioration
7	1	Progressive disease
8	10	Unacceptable toxicity (IRR, erythematous pruritic skin reaction)

Supplementary Figure 1: *Trial Profile*. Related to *Figure 1 in the manuscript*

(A) CONSORT trial profile.
(B) Reasons for ineligibility.
(C) Reasons for study discontinuation.
**Details in (B) and (C) for ineligibility and discontinuation, respectively. †In combination with modified FOLFIRINOX. CNS, central nervous system; DLT, dose-limiting toxicity; ECOG, Eastern Cooperative Oncology Group; IRR, infusion-related reaction; PI, Principal Investigator.*

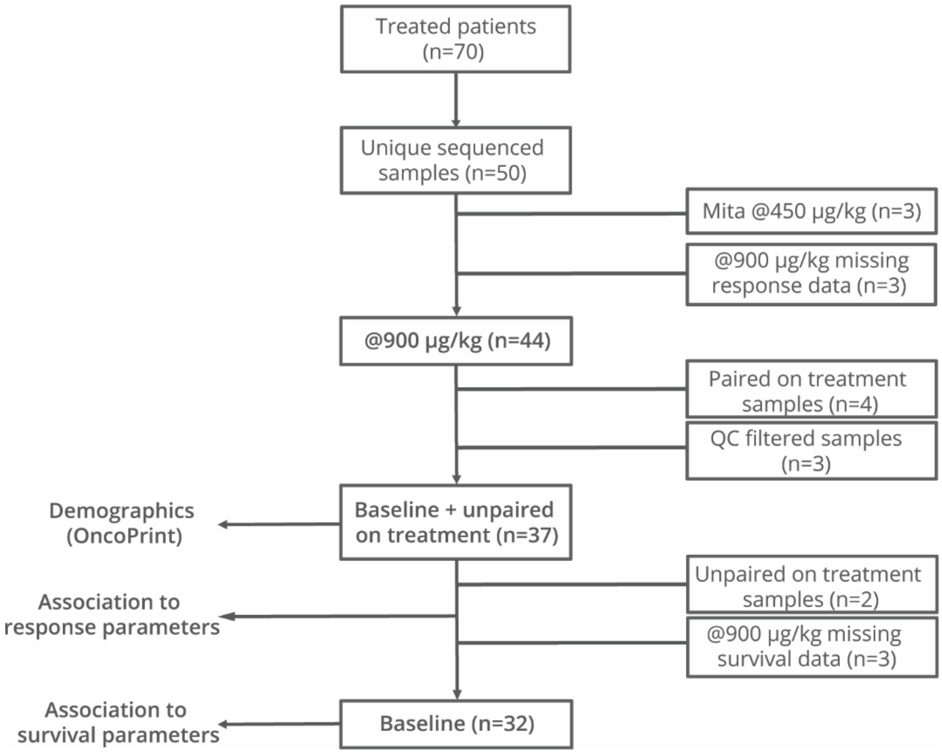

Supplementary Figure 2: *TSO500 dataset derivation. Related to Figure 2 in the manuscript*

Mita, mitazalimab; QC, quality control.

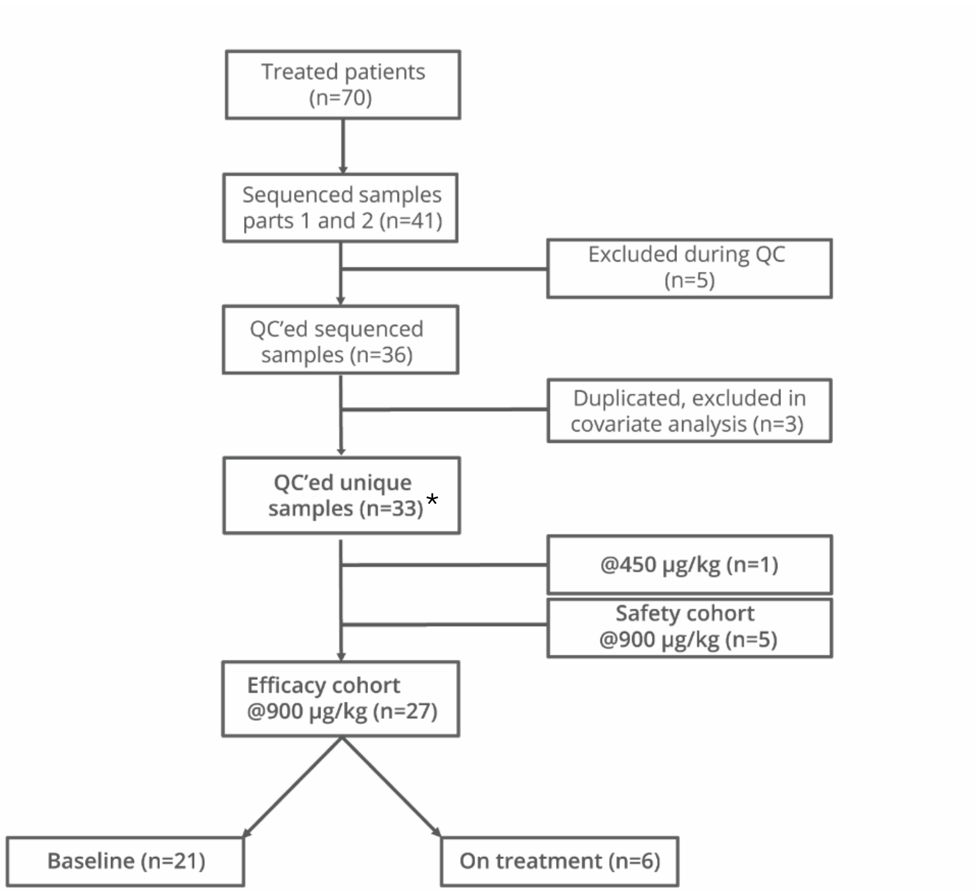

Supplementary Figure 3: *RNA-sequencing dataset derivation. Related to Figure 3 and 4 in the manuscript.*

**33 samples from 31 patients. QC, quality control.*

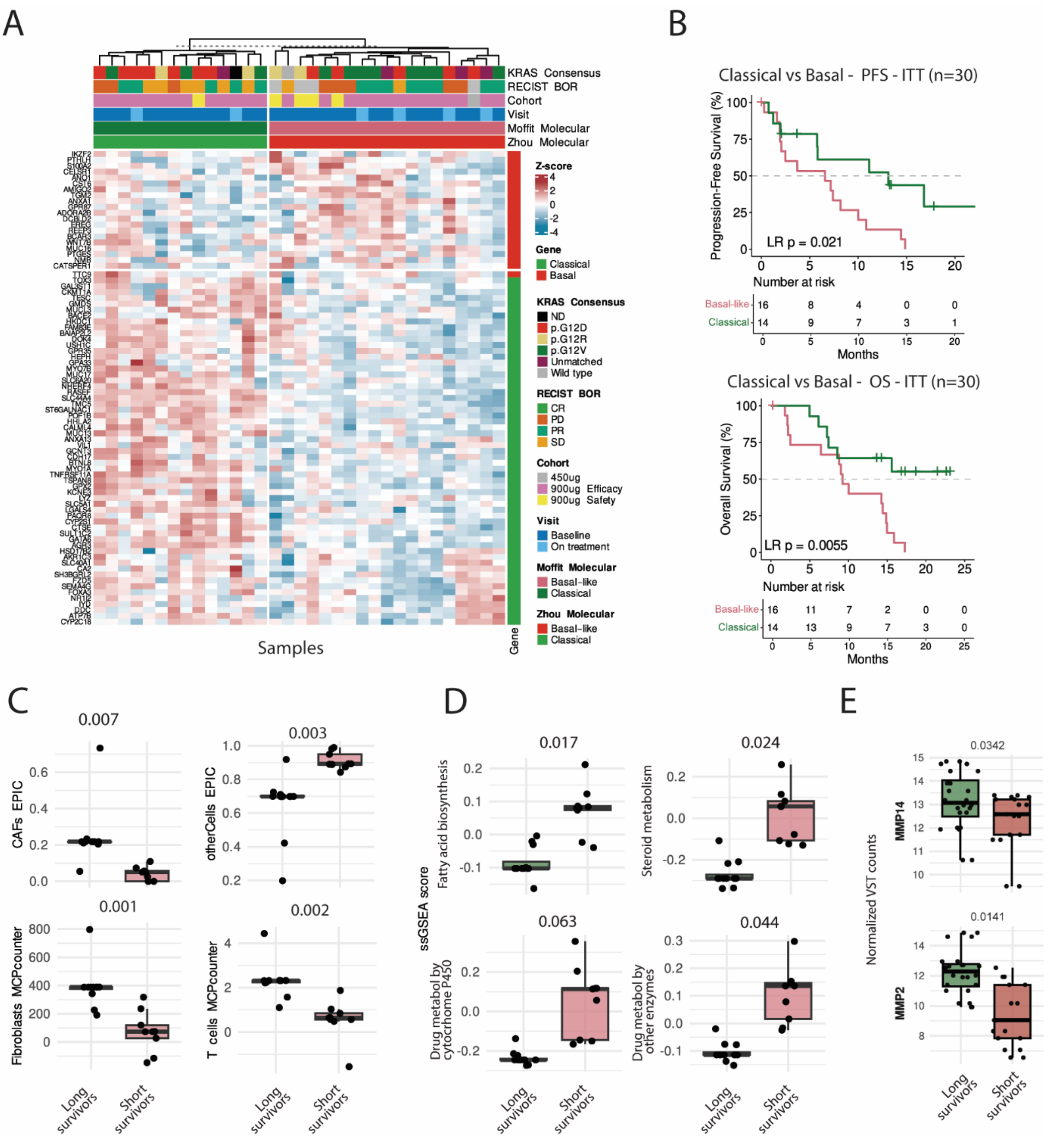

Supplementary Figure 4: *Supplementary information related to transcriptomic profiles of OPTIMIZE-1 patients. Related to Figure 3 in the manuscript*

A. Molecular subtype classification into "classical" and "basal-like" profiles (n=33 samples, 31 patients) using the gene set published by Zhou et al. B. Overall survival (OS) and progression-free survival (PFS) differences in patients from the intention to treat (ITT) set, stratified by molecular subtype (n=30). C. Tumor microenvironment (TME) deconvolution scores from patients in the full analysis set (FAS) set stratified by overall survival at 12 months (OS-12). D. TME metabolic pathway scores, of publicly available gene sets, from patients in the FAS set stratified by OS-12. E. normalized expression of matrix metallopeptidase (MMP)14 and MMP2 (selected MMP genes involved in fibrosis degradation). OS-12 (overall survival at 12 months; long survivors: post-12 months; short survivors: pre-12 months). BOR, best overall response; CAFs, cancer-associated fibroblasts; CR, complete response; EPIC, estimating the proportion of immune and cancer cells; LR, log rank; MCP, microenvironment cell population; PD, disease progression; PR, partial response; SD, stable disease; VST, variance stabilizing transformation.*Zhou X, An J, Kurilov R, et al. Persister cell phenotypes contribute to poor patient outcomes after neoadjuvant chemotherapy in PDAC.* Nat Cancer. *Sep 2023;4(9):1362-1381. doi:10.1038/s43018-023-00628-6*

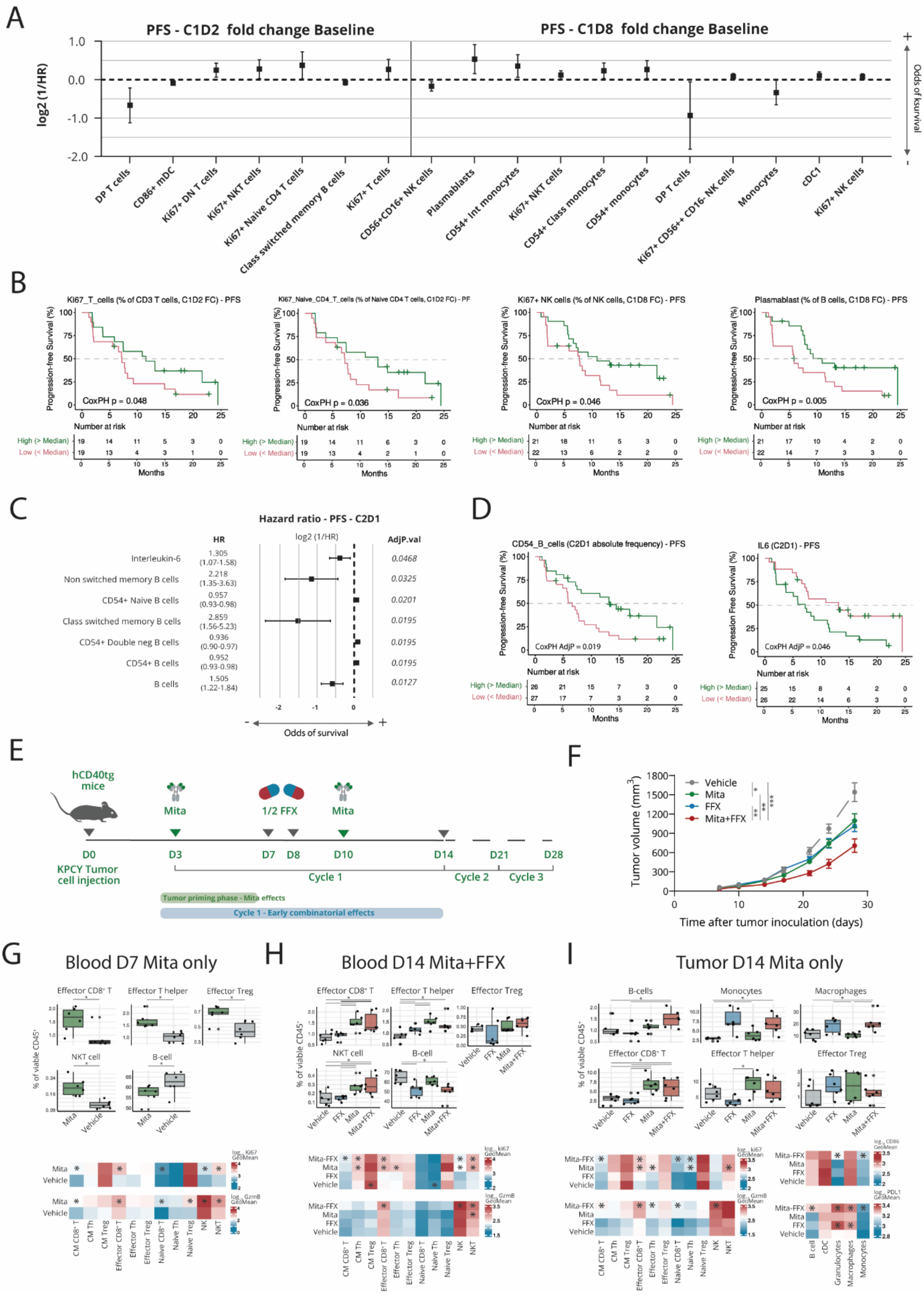

Supplementary Figure 5: *Supplementary information on tumor extrinsic factors associated with clinical efficacy endpoints, during the first cycle of OPTIMIZE-1 supporting Figure 4 in the manuscript.*

*A. and B. Mitazalimab (mita)-induced fold changes in immune cell frequency associated with progression-free survival (PFS), during the tumor priming phase of OPTIMIZE-1 (P-value<0.05). C. and D. Immune cell frequency levels associated with overall survival (OS) (adjusted p-value<0.05), induced by the combination of mitazalimab and mFOLFIRINOX during the first treatment cycle of OPTIMIZE-1. E. Dosing and sampling regimen used during in vivo experiments of hCD40tg KCPY tumor model treated with mitazalimab in combination with FOLFIRINOX (FFX). F. Anti-tumor efficacy of mice treated with mitazalimab, FFX or mitazalimab and FFX. Differences in tumor volume were calculated at day 21 using the Mann-Whitney test. * p<0.05, ** p<0.01 and, *** p<0.005. G., H. and I. Changes in immune cell frequency and activation markers levels (GeoMean: geometric mean) induced by treatment and identified in G. Blood after 96 hours of the first dose of mitazalimab (tumor priming dose), H. Blood after 96 hours of the second dose of mitazalimab (first cycle), and I. tumor tissue after 96 hours of the second dose of mitazalimab (first cycle). AdjP, adjusted P value; C, cycle; CD, cluster of differentiation; cDC, classical dendritic cell; CM, central memory; D, day; DN, double negative; DP, double positive; GzmB: granzyme B; HR, hazard ratio; IL, interleukin; Int, intermediate; mDC, myeloid dendritic cell; NK, natural killer; NKT, natural killer T-cell; PH, proportional hazards; Th, helper T-cell; Treg, regulatory T-cell.*

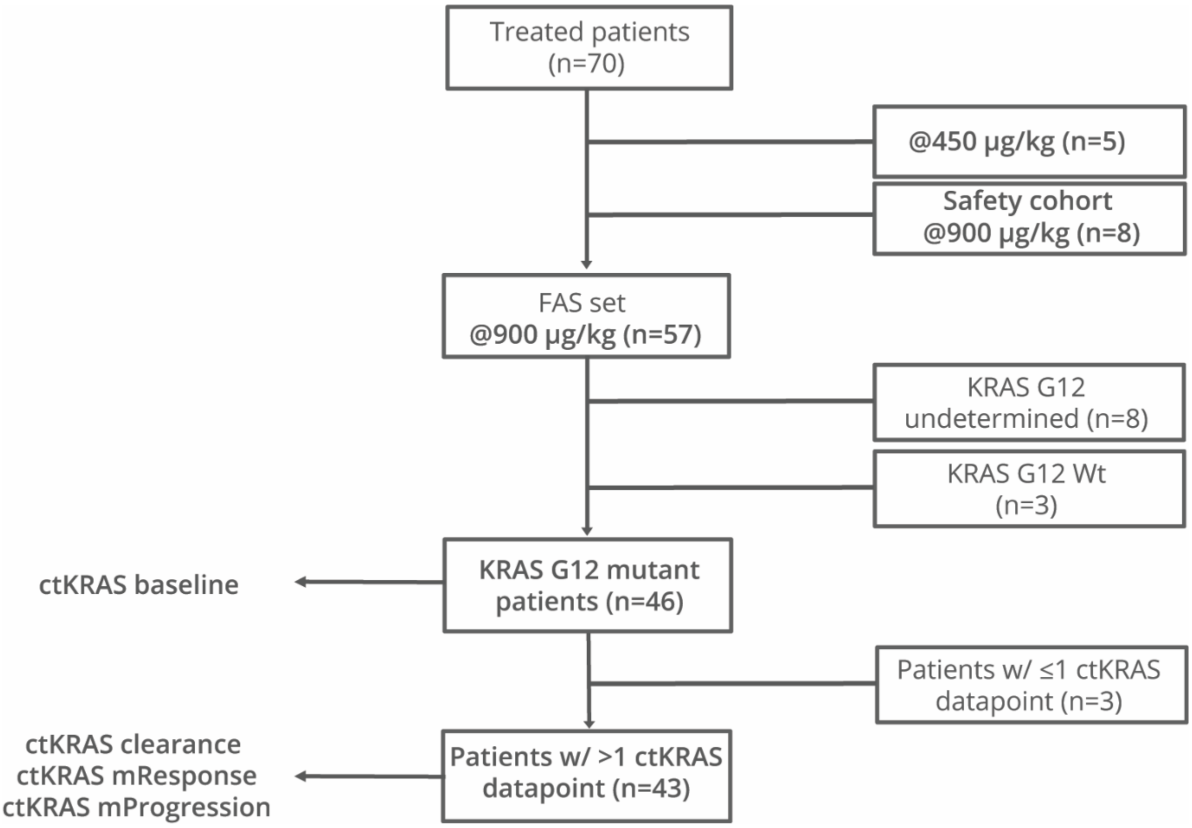

Supplementary Figure 6: *ctKRAS dataset derivation. Related to Figure 5 in the manuscript.*

FAS, full analysis set; Wt, wild type.

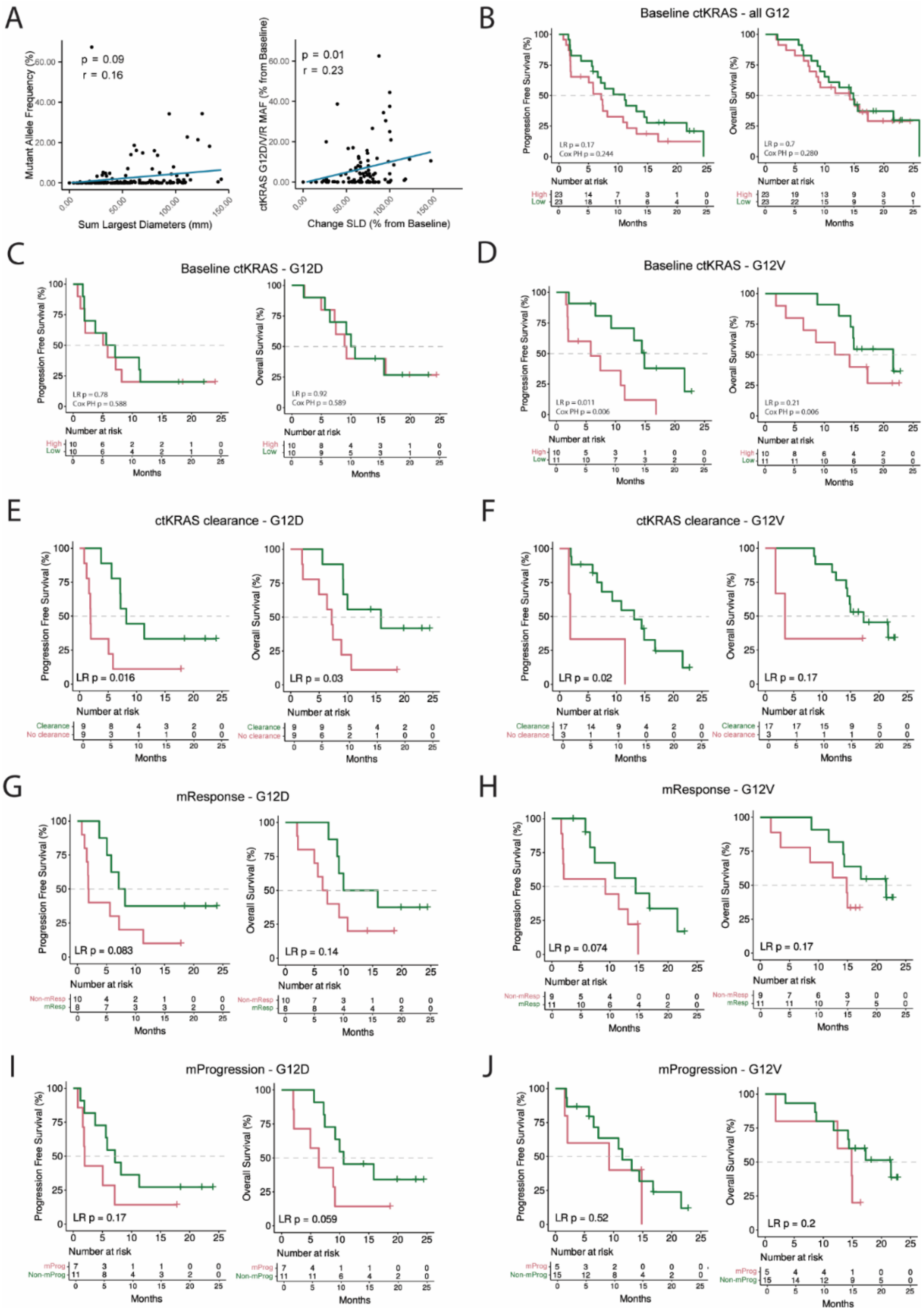

***Supplementary Figure 7:** Supplementary information related to correlation of ctDNA with tumor burden and association of clinical. Related to Figure 5 in the manuscript*

A. Correlation of sum of largest diameters (SLD) and % mutation allele frequency (MAF) in absolute numbers (left), and percentage from baseline (right). B. Differences in progression-free survival (PFS) and overall survival (OS) after stratifying patients according to ctKRAS levels at baseline. Differences in PFS and OS after stratifying G12D patients according to C. ctKRAS levels at baseline. E. ctKRAS clearance G. molecular response (mResponse), and I. molecular progression (mProgression). Differences in PFS and OS after stratifying G12V patients according to D. ctKRAS levels at baseline. F. ctKRAS clearance, H. mResponse, and J. mProgression. LR, log rank; PH, proportional hazards.

A

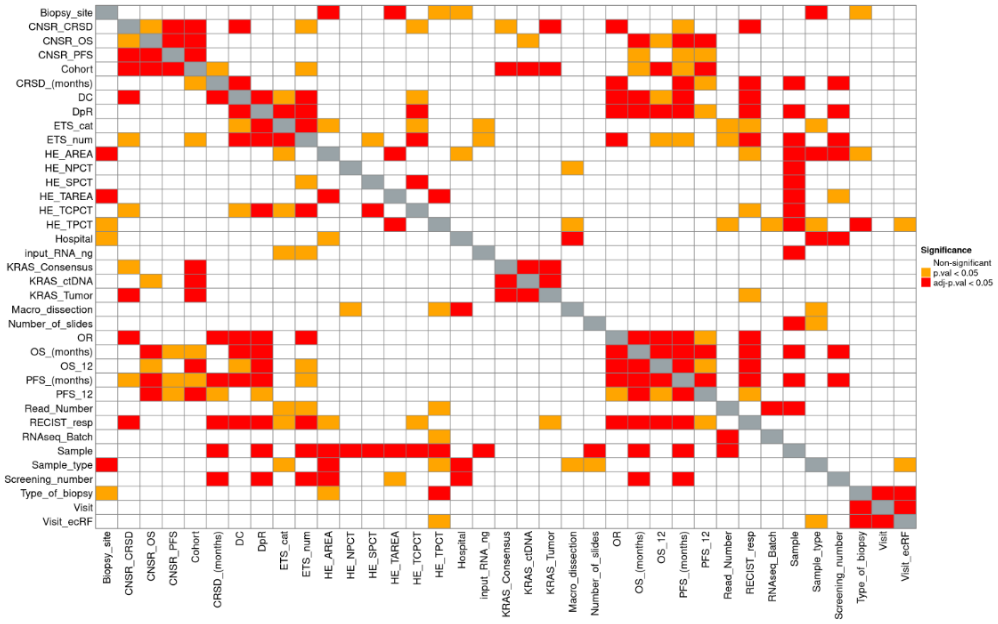

B

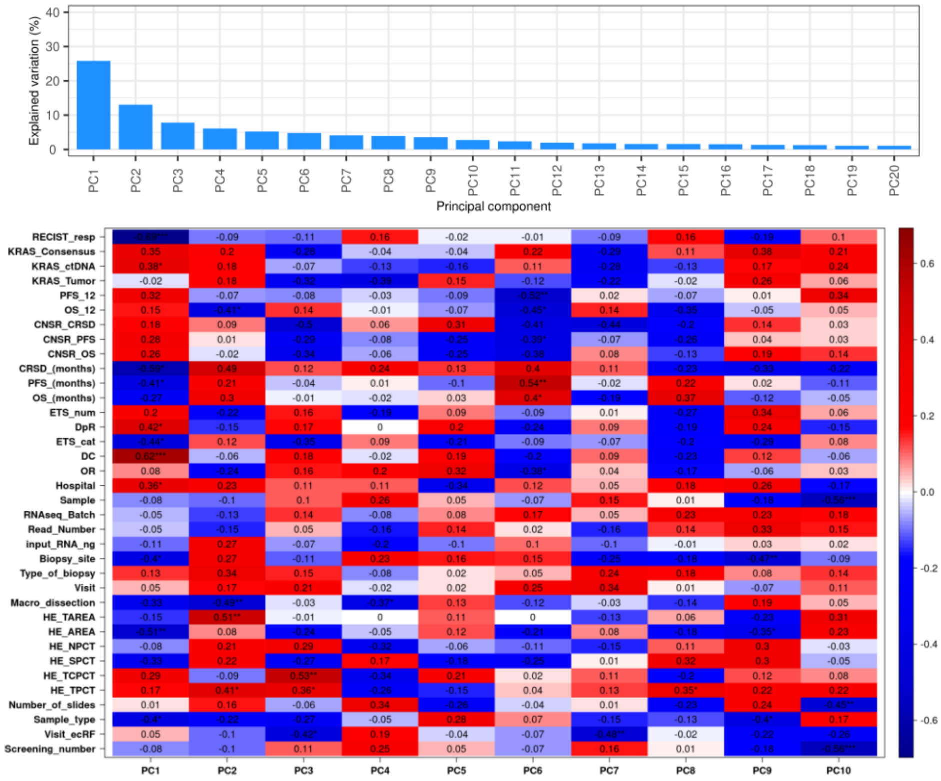

Supplementary Figure 8: *Confounding co-variate analyses. Related to "Confounding factor analysis for RNA seq" section in the STAR methods*

Thirty-three (33) unique quality controlled (QC'd) samples from the 900 µg/kg efficacy (n=27), 900 µg/kg safety (n=5) and 450 µg/kg (n=1) cohorts were included in these analyses A. Correlation between co-variates in the metadata. Pairs of significantly confounded covariates are indicated in orange (unadjusted p-value) and red (false discovery rate-

*adjusted). B. Correlation between covariates and first 10 principal components (PC) indicating Pearson correlation coefficient. PC component contribution to the dataset variance are indicated on top. *p<0.05, **p<0.005, ***p<0.0005.*

Data S1/Methods S1:Study Protocol

An open-label phase 1b/2 study assessing the safety and efficacy of mitazalimab in combination with chemotherapy in patients with metastatic pancreatic ductal adenocarcinoma

Acronym: OPTIMIZE-1
Study No: A-20-1013-C-03

EudraCT No: 2020-005182-14
Sponsor: Alligator Bioscience AB Medicon Village
SE-223 81 Lund
Sweden
Tel. +46 (0)46 540 8200
Coordinating Investigator: Professor Jean-Luc van Laethem
Hospital Erasme Route de Lennik 808 1070 Bruxelles Belgium

Medical Monitor: Theradex (Europe) Ltd (Theradex) 2nd Floor, The Pinnacle
Station Way, Crawley West Sussex, RH10 1JH United Kingdom
Tel. +44 (0)1293 510 319
Protocol Version/Date: 6.0 / 05 OCT 2

STATEMENT OF COMPLIANCE

This study will be conducted in compliance with this Clinical Study Protocol, the International Council for Harmonization (ICH) Guideline for Good Clinical Practice (GCP)(Doc. Ref. ICHE6(R2) EMA/CHMP/ICH/135/1995) and applicable regulatory requirements, and in accordance with the terms of the Declaration of Helsinki (2013) as well as with the European Medicines Agency (EMA) ‘Guideline on strategies to identify and mitigate risks for first-in-human and early clinical trials with investigational medicinal products’ (Doc. Ref. EMEA/CHMP/SWP/28367/07 Rev 1). In case of an update in any of the guidelines this will, if applicable, be included in the next Amendment of the Protocol.

INVESTIGATOR SIGNATURE PAGE

1. I have carefully read this Clinical Study Protocol entitled ‘An open-label phase 1b/2 study assessing the safety and efficacy of mitazalimab in combination with chemotherapy in patients with metastatic pancreatic ductal adenocarcinoma, and agree that it contains all the necessary information required to conduct the study. I agree to conduct this study as outlined in the protocol.
2. I understand that this study will not be initiated without approval of the appropriate Ethics Committee (EC), and that all administrative requirements of the governing body of the Institution will be complied with fully.
3. Informed written consent will be obtained from all participating patients in accordance with institutional guidelines, Food and Drug Administration (FDA) requirements as specified in Title 21 Code of Federal Regulations (CFR) Part 50, the European Union Directive 2001/20/EC and its associated Detailed Guidance, European Union GCP Directive 2005/28/EC, European Union General Data Protection Regulation (GDPR) 2016/679, the ICH GCP, Section 4.8, and the terms of the Declaration of Helsinki (2013).
4. I will enroll patients who meet the protocol criteria for entry.
5. I understand that my signature on each completed electronic Case Report Form (eCRF) indicates that I have carefully reviewed the complete set of eCRFs and accept full responsibility for the contents thereof.
6. I understand that the information presented in this Clinical Study Protocol is confidential, and I hereby assure that no information based on the conduct of the study will be released without prior consent from the Sponsor unless this requirement is superseded by the FDA, a Competent Authority of the European Union or another Regulatory Authority.

Investigator Name: __ Institution Name: ___ Institution Address: _

____

____

Telephone: ______________________________

Signature: ______ Date: _

VERSION HISTORY
Version 6.0/05 OCT 2022 (Amendment 5)

Version 5.0/15 DEC 2021 (Amendment 4)

Version 4.0/21 MAY 2021 (Amendment 3)

Version 3.0 / 12 MAR 2021 (Amendment 2)

Version 2.0 / 9 FEB 2021 (Amendment 1)

Version 1.0 / 27 NOV 2020 (Original)

SYNOPSIS

GENERAL INFORMATION

Study title: An open-label phase 1b/2 study assessing the safety and efficacy of mitazalimab in combination with chemotherapy in patients with metastatic pancreatic ductal adenocarcinoma
Region: Europe

Estimated number of sites: 6 sites for Part 1 and up to 15 sites for Part 2

Number of patients planned: Up to 80 patients

Patient population: Patients, at least 18 years of age, diagnosed with previously untreated metastatic pancreatic ductal adenocarcinoma

INVESTIGATIONAL MEDICINAL PRODUCT, DOSAGE AND ADMINISTRATION

Mitazalimab will be assessed in combination with the chemotherapy modified FOLFIRINOX (mFOLFIRINOX). Mitazalimab and mFOLFIRINOX will be administered by intravenous infusions following a 14-day cycle schedule, except for the first treatment cycle of 21 days where mitazalimab is administered at Day 1 and 10 and infusion of mFOLFIRINOX start Day 8.
Mitazalimab: An agonistic human monoclonal (IgG1) antibody targeting CD40, developed for cancer immunotherapy. In Part 1 (Phase 1b) of the study, the dose of mitazalimab will be escalated from a starting dose of 450 µg/kg up to 900 µg/kg (2 dose levels to be evaluated) to obtain the recommended phase 2 dose (RP2D) of mitazalimab in combination with mFOLFIRINOX. In Part 2 of the study, the RP2D of mitazalimab will be administered in combination with mFOLFIRINOX to all patients.
The chemotherapy is administered as background treatment as it is standard of care for first line treatment of advanced pancreatic cancer recommended by the European Society of Medical Oncology (ESMO) and the National Comprehensive Cancer Network (NCCN). Therefore, mFOLFIRINOX and gemcitabine plus nab-paclitaxel are classified as Non-Investigational Medicinal Products.
mFOLFIRINOX: The mFOLFIRINOX treatment will consist of oxaliplatin (85 mg/m2), leucovorin (400 mg/m2), irinotecan (150 mg/m2) and 5-fluorouracil (5-FU, 2400 mg/m2).
In case of supply issues with leucovorin, or to adhere to local standard of care practice, other approved similar folinates may be used under the supervision of an experienced clinician.
In case mFOLFIRINOX is not found safe and tolerable together with the lowest dose of mitazalimab, mFOLFIRINOX will be exchanged to gemcitabine plus nab-paclitaxel.

Gemcitabine plus nab-paclitaxel (if applicable): Gemcitabine in combination with nab-paclitaxel is approved in Europe for the first line treatment of adult patients with metastatic adenocarcinoma of the pancreas. Gemcitabine plus nab-paclitaxel will only be administered in this study in case mFOLFIRINOX is not found safe and tolerable in combination with mitazalimab at the lowest dose-level.

STUDY DESIGN

A phase 1b/2, open-label, multicenter study designed to evaluate the safety, tolerability, and efficacy of mitazalimab in combination with chemotherapy in patients with metastatic pancreatic ductal adenocarcinoma.
The study consists of 2 parts:

Part 1 (Phase 1b): Two dose levels of mitazalimab in combination with mFOLFIRINOX will be evaluated to determine a tolerable mitazalimab dose (RP2D) for Part 2 of the study. Part 1 follows a Bayesian optimal interval (BOIN) design with at least 3 patients enrolled at each dose level. As a minimum, 6 patients will be evaluated at the RP2D in Part 1. If the lowest dose of mitazalimab (450 µg/kg) in combination with mFOLFIRINOX is not found safe and tolerable, the mFOLFIRINOX treatment will be exchanged to gemcitabine plus nab- paclitaxel.
Part 2 (Phase 2): Up to 60 enrolled patients will be administered mitazalimab and mFOLFIRINOX (or gemcitabine plus nab-paclitaxel) to explore the clinical efficacy of mitazalimab in combination with chemotherapy in Part 2 of the study. A Simon's two-stage design with an interim analysis for stop for futility will be performed when 23 patients in total (including patients from both Part 1 and Part 2) are evaluable for objective response rate (ORR).

DURATION OF TREATMENT

The patients may receive treatment with mitazalimab and mFOLFIRINOX for 12 treatment cycles (~6 months; one 21-day treatment period/dose-limiting toxicity (DLT) evaluation period (Cycle 1) followed by eleven 14-day treatment cycles).
The patients may receive treatment with mitazalimab and gemcitabine plus nab-paclitaxel for a maximum of 6 treatment cycles (~6 months; one 35-day treatment period (Cycle 1) whereof the first 28 days will be the DLT evaluation period followed by five 28-day treatment cycles) (if applicable).
Patients may continue study treatment within above defined limits until progressive disease (PD), or clear clinical deterioration, according to Investigator's judgment, as long as the patients are tolerating the treatment and agree to continue. After study treatment completion the patients

perform an End of treatment visit and continue to a treatment follow up period assessing disease and survival status.
Upon completion of 12 cycles (mitazalimab and mFOLFIRINOX), the Investigator will review and assess each patient´s health and determine the best treatment options available. If the Investigator deems that a patient has shown clinical benefit from the combination treatment with mitazalimab and mFOLFIRINOX and recommends the patient to continue with this combination, the patient can continue study treatment.
The post-treatment follow-up period will continue for up to 2 years after last patient in (LPI) or until disease progression or clinical deterioration if study treatment continues past the 2 years after LPI.

OBJECTIVES AND ENDPOINTS PART 1

OBJECTIVES	ENDPOINTS
**Primary**	
To determine the recommended Phase 2 dose (RP2D) of mitazalimab in combination with chemotherapy	• Incidence of DLTs
**Secondary**	
To assess the safety and tolerability of mitazalimab in combination with chemotherapy	• Type, frequency and severity of adverse events (AEs)
To assess the immunogenicity of mitazalimab	• Detection and characterization of anti-drug antibody (ADA) titers in serum
To assess pharmacokinetics (PK) of mitazalimab after single and repeated administrations	• PK parameters will include C_{max}, T_{max}, and $AUC_{(0-T)}$. Additional parameters may be calculated depending on data obtained
To assess the clinical activity of mitazalimab in combination with chemotherapy (i.e., anti-tumor activity as per RECIST v. 1.1 guideline)	• Objective response rate (ORR)<br>• Best Overall Response (BOR), with response categories CR, PR, SD and PD<br>• Duration of response (DoR)<br>• Duration of SD<br>• Disease control rate<br>• Time to next anti-cancer therapy
To assess survival outcomes following repeated administrations of mitazalimab in combination with chemotherapy	• Progression-free survival (PFS)<br>• Overall survival (OS)
**Exploratory**	
To characterize the impact of mitazalimab in combination with chemotherapy on the immune response both systemically and in the tumor microenvironment	• Change in cytokines and chemokines<br>• Quantification of changes in innate and adaptive immune cell populations and immune cell activation status<br>• Change in gene expression and immune signatures, assessing differentially expressed genes and gene signatures for specific immune cells
To characterize the impact on CA19-9 and ctDNA of mitazalimab in combination with chemotherapy	• Change in CA19-9<br>• Change in ctDNA and differentially expressed genes of interest

PART 2

OBJECTIVES	ENDPOINTS
**Primary**	
To assess the clinical activity of mitazalimab in combination with chemotherapy (i.e., anti-tumor activity as per RECIST v. 1.1 guideline)	• Objective response rate (ORR)
**Secondary**	
To assess the clinical activity of mitazalimab in combination with chemotherapy (i.e., anti-tumor activity as per RECIST v. 1.1 guideline)	• Best Overall Response (BOR), with response categories CR, PR, SD and PD<br>• Duration of response (DoR)<br>• Duration of SD<br>• Disease control rate<br>• Time to next anti-cancer therapy
To assess survival outcomes following repeated administrations of mitazalimab in combination with chemotherapy	• Progression-free survival (PFS)<br>• Overall survival (OS)
To assess the safety and tolerability of mitazalimab in combination with chemotherapy	• Type, frequency and severity of AEs
To assess the immunogenicity of mitazalimab	• Detection and characterization of anti-drug antibody (ADA) titers in serum
To assess PK of mitazalimab after single and repeated administrations	• PK parameters will include Cmax, Tmax, and AUC(0-T). Additional parameters may be calculated depending on data obtained
**Exploratory**	
To characterize the impact of mitazalimab in combination with chemotherapy on the immune response both systemically and in the tumor microenvironment	• Change in cytokines and chemokines<br>• Quantification of changes in innate and adaptive immune cell populations and immune cell activation status<br>• Change in gene expression and immune signatures, assessing differentially expressed genes and gene signatures for specific immune cells
To characterize the impact on CA19-9 and ctDNA of mitazalimab in combination with chemotherapy	• Change in CA19-9<br>• Change in ctDNA and differentially expressed genes of interest

ASSESSMENTS

Assessments include demographics, medical history (including previous anti-cancer treatments), height and weight, vital signs (blood pressure, pulse rate, oxygen saturation and body temperature), physical examination, ECG, ECOG performance status, and clinical laboratory tests (clinical chemistry, hematology, urinalysis), concomitant medication and collection of AEs. Blood samples will be taken for analysis of pharmacokinetics, pharmacodynamics, and immunogenicity analyses.
Anti-tumor activity will be evaluated by assessing CT scans according to RECIST v. 1.1 guideline.

A post-treatment follow-up period will include assessment of disease/survival status, subsequent cancer-related therapies/procedures, and anti-tumor activity (CT-scan). The post-treatment follow-up period will continue for up to 2 years after last patient in (LPI) or until disease progression or clinical deterioration if study treatment continues past the 2 years after LPI.

STATISTICS

Statistical methodology

No formal statistical hypothesis is defined for Part 1 (Phase 1b).

For Part 2, the primary endpoint ORR will be compared between mitazalimab and historical control using a one-sided exact binomial test performed on the 10% significance level. All secondary and exploratory endpoints will be summarized using appropriate descriptive statistics. Patients in Part 1 who are on the same dose regimen as patients in Part 2 will be pooled together with patients enrolled in Part 2 for statistical analyses and data summaries.

Sample size

In Part 1, it is estimated that at least 9 patients will be enrolled for the 2 dose levels planned, 3 patients at the lower level and 3+3 patients at the higher dose level. The actual number of patients in Part 1 will be dependent on data obtained during the study.
For Part 2, the sample size is based on extended Simon's two-stage design including an interim analysis for futility and efficacy, and assuming a 15% drop-out rate however only the futility stop will be implemented. A total of 23 patients at RP2D (Part 1 + Part 2) with data evaluable for ORR will be included in the futility. To achieve this number, an estimate of 27 patients needs to be enrolled. An additional 37 patients may be enrolled if the study continues, depending on the outcome of the interim analysis, to include a total of 54 evaluable patients.

LIST OF ABBREVIATIONS AND TERMS

Abbreviation/term Definition

ADA	Anti-drug antibody (i.e., antibodies against mitazalimab)
ADCC	Antibody-dependent cellular cytotoxicity
AE	Adverse event
AESI	Adverse event of special interest
ALT	Alanine aminotransferase
aPTT	activated partial thromboplastin time
AST	Aspartate aminotransferase
AUC	Area under the serum concentration-time curve
AR	Adverse reaction
BOR	Best overall response
BSA	Body surface area
Cmax	Maximum serum concentration
CA 19-9	Carbohydrate antigen 19-9
CD40	Cluster of differentiation 40
CL	Clearance
CNS	Central nervous system
CR	Complete response
CRO	Contract Research Organization
CT	Computed tomography
CTCAE	Common Terminology Criteria for Adverse Events
ctDNA	Circulating tumor DNA
DC	Dendritic cells
DLT	Dose-limiting toxicity
DoR	Duration of response
DPD	Dihydropyrimidine dehydrogenase
DRC	Data Review Committee
EC	Ethics Committee
ECG	Electrocardiogram
eCRF	electronic Case Report Form
ECOG	Eastern Cooperative Oncology Group
EMA	European Medicines Agency
ESMO	European Society for Medical Oncology
EVCTM	EudraVigilance Clinical Trial Module
FAS	Full Analysis Set
FDA	Food and Drug Administration (US)
5-FU	5-Fluorouracil

G-CSF	Granulocyte Colony-Stimulating Factor
GDPR	General Data Protection Regulation (EU) 2016/679
Gem-NabPac	Gemcitabine plus nab-paclitaxel
GLP	Good Laboratory Practice
ICD-O	International Classification of Diseases for Oncology
ICF	Informed Consent Form
ICH	International Conference on Harmonization
IFNγ	Interferon gamma
IL-2 (IL-6, IL-8 etc)	Interleukin-2 (Interleukin-6, Interleukin-8 etc)
IMP	Investigational medicinal product
IMPD	Investigational Medicinal Product Dossier
IP-10	Interferon-gamma inducible Protein 10
ISF	Investigator Site File
IV	Intravenous
IWRS	Interactive Web Response System
LPI	Last patient in
MIP-1B	Macrophage Inflammatory Protein 1B
mFOLFIRINOX	modified FOLFIRINOX
MCP-1	Monocyte Chemoattractant Protein-1
MRI	Magnetic Resonance Imaging
MTD	Maximum Tolerated Dose
NCCN	National Comprehensive Cancer Network
ORR	Objective Response Rate
OS	Overall survival
PD	Progressive disease
PFS	Progression-free survival
PK	Pharmacokinetics
PO	Per oral
PP	Per Protocol
PR	Partial response
RSI	Reference safety information
SAE	Serious adverse event
SAR	Serious adverse reaction
SD	Stable disease
SOC	System Organ Class
SUSAR	Suspected Unexpected Serious Adverse Reaction
Tmax	Time to Cmax
TMF	Trial Master File

1 VISIT ASSESSMENT SCHEDULE

The visit assessments tables below are applicable when administering mitazalimab in combination with mFOLFIRINOX and include all assessments to be performed during the study. Table 1 lists the study assessments to be performed during screening and Treatment cycle 1. Table 2 lists the assessments to be performed during Treatment cycle 2 – 12, and at the End of treatment. Table 3 lists the assessments to be performed during cycle 13 and subsequent cycles and the End of treatment for patients where a decision has been taken to continue treatment with mitazalimab and mFOLFIRINOX beyond cycle 12. End of treatment is defined as discontinuation of mitazalimab treatment. The assessments to be performed during the post-treatment follow-up period are listed in Table 4. The timing of the different assessments is indicated with an “X”.

A number of pre- and post-medications must be administered with specific timing in relation to administration (up to 3 days prior to infusion) of mitazalimab and mFOLFIRINOX, respectively. Details are not shown in the tables but are provided in Section 8.9.1 and 8.9.3.

The assessment schedules are applicable for both Part 1 and Part 2 of the study.

Visit assessment schedules to be used when administering mitazalimab in combination with gemcitabine plus nab-paclitaxel are found in APPENDIX 3.

Table 1 Assessment schedule during screening period and Treatment cycle 1

**Activity/Visit**	**Screening**	**Cycle 1 (21-day treatment cycle)**													**Unscheduled visit[1]**
**Day**	**-21 to -1**	**1**			**2**	**8±1**		**9±1**	**10±1**	**10±13**			**11±1**	**17±4**	
**Time in relation to study drug**		**Pre**	**Mitazalimab**	**Post[2]**	**24±2h[2]**	**Pre**	**mFOLFIRINOX[3]**			**Pre**	**Mitazalimab[3]**	**Post[2]**	**24±2h[2]**		
**Informed Consent** (Section 14.1)	X[4]														
**Eligibility criteria** (Section 7.1 and 7.2)	X														
**Demographics** (Section 10.1)	X														
**Medical history** (Section 10.2)	X														
**Body weight** (Section 10.3)	X[12]	X[5]													X
**Height** (Section 10.3)	X														
**Vital signs** (Section 10.4)	X	X		1h ±10min<br>2h±10min<br>4h±15min		X[5]						1h ±10min<br>2h±10min			X
**Physical examination** (Section 10.5)	X	X[5]				X									X
**12-lead ECG** (Section 10.6)	X	X[5]		1h ±10min								1h ±10min			X

				4h±15min											
**ECOG** (Section 10.7)	X	X5													X
**Treatment discontinuation criteria** (Section 9.1)		X6				X6				X6					X6
**Pre- and post-medication**		**Note**: Pre-medications are to be given up to 3 days prior to administration of study treatment. For detailed information of timing of pre-and post-medications, please see Section 8.9.1 and 8.9.3.													
**Mitazalimab administration** (Section 8.3)			X7								X7				
**mFOLFIRINOX administration** (Section 8.9.2.1)															
Oxaliplatin							X								
Irinotecan							X								
Leucovorin13							X								
5-FU							X	X	X						

**Activity/Visit**	**Screening**	**Cycle 1 (21-day treatment cycle)**													**Unscheduled visit1**
**Day**	**-21 to -1**	**1**			**2**	**8±1**		**9±1**	**10±1**	**10±13**			**11±1**	**17±4**	
**Time in relation to study drug**		**Pre**	**Mitazalimab**	**Post2**	**24±2h2**	**Pre**	**mFOLFIRINOX3**			**Pre**	**Mitazalimab3**	**Post2**	**24±2h2**		
**Adverse events** (Section 11.2)	Continuous from signing ICF until 28 days after last dose of study drug														
**Concomitant medication** (Section 8.9.7)	Continuous from signing of ICF until End of treatment visit														
**Concomitant medical procedures** (Section 10.8)	Continuous from signing of ICF until End of treatment visit														
**Clinical laboratory tests** (Section 10.9)															
Pregnancy test	X	X8													X
DPD	X11														
Clinical chemistry, hematology and coagulation	X	X5				X5				X			X	X	X
Urinalysis	X	X5				X5									X
**Pharmacokinetics** (Section 10.11)		X5		5min, 1h±10min 2h±10min 4h±20min	X	X				X		5min, 1h±10min 2h±10min 4h±20min	X	X	X
**Immunogenicity** (Section 10.10)		X5													X

**CT scan/ Tumor response evaluation** (Section 10.12)	X9														X
**CA19-9** (Section 10.13.1)	X	X5													X
**Immunophenotyping** (Section 10.13.1)		X5			X	X				X			X		X
**Cytokines and chemokines** (Section 10.13.1)		X5		1h±10min<br>4h±20min	X	X				X		1h±10min<br>4h±20min	X		X
**Whole blood RNA** (Section 10.13.1)		X5		1h±10min<br>4h±20min	X	X				X		1h±10min<br>4h±20min	X		
**Whole blood DNA** (Section 10.13.1)	X														
**Tumor biopsy** (Section 10.13.2)	X10														

1. The assessments to be performed during unscheduled visits should be based on Investigator's judgement.
2. Assessments to be performed at specified time points after End of infusion. The End of infusion is defined as when the infusion of mitazalimab is completed/stopped (i.e., before rinsing if applicable). For example, 4h post-dose means 4 hours after end of infusion.
3. mFOLFIRINOX should be administered during 3 consecutive days. Mitazalimab should be administered on the same day as the day mFOLFIRINOX administration has ended or the day after.
4. Informed consent must be obtained prior to performing any screening assessments
5. The assessment may be performed within 72 hours (up to 3 days) prior to administration of study treatment. If the screening assessment was taken Day -1 to -3 prior to dosing, it does not need to be repeated.
6. If any of the treatment discontinuation criteria apply, the patient should be discontinued from treatment and the End of treatment visit should be performed, see End of treatment visit in Table 2.
7. If the infusion of mitazalimab is interrupted due to an AE, a PK sample and a sample for immunogenicity should be taken at the time of interruption, or as soon as is feasible considering the patient safety. An immunogenicity sample does not need to be taken if it is first administration of mitazalimab that is interrupted.
8. The result of the pregnancy test must be available prior to dosing.
9. The CT scan at screening may be obtained 28 days prior to first dose of mitazalimab.
10. The baseline biopsy may be collected up to 28 days before first dose of mitazalimab. If a fresh biopsy cannot be taken during the screening period, archival biopsy material (most recent) could be used.
11. The DPD laboratory test may be collected up to 28 days before the first dose of mitazalimab.
12. Body weight assessed before first dose of mitazalimab will be used for calculation of dose throughout study as long as the body weight does not change more than 10% from baseline.
13. In case of supply issues with leucovorin, or to adhere to local standard of care practice, other approved similar folinates may be used under the supervision of an experienced clinician.

Table 2 Assessment schedule for Treatment cycle 2 - 12

**Activity/Visit**	**Cycle 2 - 12 (14-day treatment cycles)**								**Unscheduled visit1**	**End of treatment visit2**
**Cycle day**	**13**		**2**	**3**	**3+1**			**10±4**		
**Time in relation to study drug**	**Pre**	**mFOLFIRINOX4**			**Pre**	**Mitazalimab4**	**Post5**			**28 ±4 days after last dose**
**Body weight** (Section 10.3)	X6								X	
**Vital signs** (Section 10.4)	X				X		1h±10min		X	X
**Physical examination** (Section 10.5)	X6				X6				X	X
**12-lead ECG** (Section 10.6)							1h±10min12		X	X
**ECOG** (Section 10.7)	X6								X	X
**Treatment discontinuation criteria** (Section 9.1)	X7				X7				X7	
**Pre- and post-medication**	**Note**: Pre-medications are to be given up to 3 days prior to administration of study treatment. For detailed information of timing of pre-and post-medications, see Section 8.9.1 and 8.9.3.									
**mFOLFIRINOX administration** (Section 8.9.2.1)										
Oxaliplatin		X								
Irinotecan		X								
Leucovorin13		X								
5-FU		X	X	X						
**Mitazalimab administration**						X8				

(Section 8.3)										
**Adverse events** (Section 11.2)	Continuous from signing ICF until 28 days after last dose of study treatment									
**Concomitant medication** (Section 8.9.7)	Continuous from signing of ICF until End of treatment visit									
**Concomitant medical Procedures** (Section 10.8)	Continuous from signing of ICF until End of treatment visit									
**Clinical laboratory tests** (Section 10.8)										
Pregnancy test	X9								X	X

**Activity/Visit**	**Cycle 2 - 12 (14-day treatment cycles)**								**Unscheduled visit1**	**End of treatment visit2**
**Cycle day**	**13**		**2**	**3**	**3+1**			**10±4**		
**Time in relation to study drug**	**Pre**	**mFOLFIRINOX4**			**Pre**	**Mitazalimab4**	**Post5**			**28 ±4 days after last dose**
Clinical chemistry, hematology and coagulation	X6				X6				X	X
Urinalysis	X6								X	X
**Pharmacokinetics** (Section 10.11)	X (Cycle 2, 4, 6, 8, 10, 12 only)						1h±10min (Cycle 2, 4, 6, 8, 10, 12 only)		X	X
**Immunogenicity** (Section 10.10)	X (Cycle 2, 4, 6, 8, 10, 12 only)								X	X
**CT scan/Tumor response evaluation** (Section 10.12)								X (Cycle 4, 8, 12)		X14
**CA19-9** (Section 10.13.1)	X								X	X14
**Immunophenotyping** (Section 10.13.1)	X (Cycle 2, 4, 6, 8, 10, 12 only)								X	
**Cytokines and chemokines** (Section 10.13.1)	X (Cycle 2, 4, 6, 8, 10, 12 only)								X	

**Whole blood RNA** (Section 10.13.1)	X (Cycle 2, 4, 6, 8, 10, 12 only)									
**Whole blood DNA** (Section 10.13.1)	X (Cycle 2, 4, 6, 8, 10, 12 only)									
**Tumor biopsy** (Section 10.13.2)								X10 (Cycle 2)	X10, 11	X10, 11

1. The assessments to be performed during the unscheduled visits should be based on Investigator's judgement.
2. For patients who discontinue mitazalimab treatment before or at 12 cycles the End of treatment visit is completed and the patient will enter the post-treatment follow-up period (please refer to Table 4), or if any of the study withdrawal criteria in Section 9.3 apply, the patient will be withdrawn from the study and have the End of treatment visit and End of study visit assessments performed (please refer to the End of study visit in Table 4).
3. Day 1 must be 14±1 days after the start of the last mFOLFIRINOX administration.
4. mFOLFIRINOX should be administered during 3 consecutive days. Mitazalimab should be administered on the same day as the day mFOLFIRINOX administration has ended or the day after.
5. Assessments to be performed at specified time after End of infusion. The End of infusion is defined as when the infusion of IMP is completed/stopped (i.e,. before rinsing if applicable). For example, 1h post-dose means 1 hour after end of infusion.
6. The assessment may be performed within 72 hours (i.e., up to 3 days) prior to administration of study treatment.
7. If any of the treatment discontinuation criteria apply, the patient should be discontinued from treatment. and the End of treatment visit should be performed.
8. If the infusion of mitazalimab is interrupted due to an AE, a PK sample and a sample for immunogenicity should be taken at the time of interruption, or as soon as is feasible considering patient safety.
9. The result of the pregnancy test must be available prior to dosing.
10. Biopsy to be taken only if baseline biopsy (fresh or archival) was obtained at screening. Biopsy can be collected after cycle 2, if not possible at Cycle 2.
11. Biopsy not to be taken if already obtained in Cycle 2.
12. Assessment to be obtained in Cycle 2 and 3 only
13. In case of supply issues with leucovorin, or to adhere to local standard of care practice, other approved similar folinates may be used under the supervision of an experienced clinician.
14. Assessment to be performed only if there is no prior confirmatory CT scan or clinical or radiological progression and no new anti-cancer therapy started prior to the visit

Table 3 Assessment schedule for Treatment cycle 13 and subsequent cycles (Only for patients considered to benefit from continued treatment)

**Activity/Visit**	**Cycle 13 and subsequent cycles (14-day treatment cycles)**								**Unscheduled visit1**	**End of treatment visit2**
**Cycle day**	**13**		**2**	**3**	**3+1**			**10±4**		
**Time in relation to study drug**	**Pre**	**mFOLFIRINOX4**			**Pre**	**Mitazalimab4**	**Post5**			**28 ±4 days after last dose**
**Informed Consent** (Section 14.1)	X									
**Treatment discontinuation criteria** (Section 9.1)	X6				X6				X6	
**Pre- and post-medication**	**Note**: Pre-medications are to be given up to 3 days prior to administration of study treatment. For detailed information of timing of pre-and post-medications, see Section 8.9.1 and 8.9.3.									
**mFOLFIRINOX administration** (Section 8.9.2.1)										
Oxaliplatin		X								
Irinotecan		X								
Leucovorin7		X								
5-FU		X	X	X						
**Mitazalimab administration** (Section 8.3)						X				
**Adverse events** (Section 11.2)	Continuous from signing ICF until 28 days after last dose of study treatment									
**Concomitant medication** (Section 8.9.7)	Continuous from signing of ICF until End of treatment visit									

**Concomitant medical Procedures** (Section 10.8)	Continuous from signing of ICF until End of treatment visit									
**CT scan/Tumor response evaluation** (Section 10.12)								X (Cycle 16, 20, 24 etc)		X8

1. The assessments to be performed during the unscheduled visits should be based on Investigator's judgement.
2. For patients who discontinue mitazalimab treatment the End of treatment visit is completed, and the patient will enter the post-treatment follow-up period (please refer to Table 4). The post-treatment follow-up period will continue for up to 2 years after last patient in (LPI) or until disease progression or clinical deterioration if study treatment continues past the 2 years after LPI.
3. Day 1 must be 14±1 days after the start of the last mFOLFIRINOX administration.
4. mFOLFIRINOX should be administered during 3 consecutive days. Mitazalimab should be administered on the same day as the day mFOLFIRINOX administration has ended or the day after. After Cycle 12, the mFOLFIRINOX regimen can be adapted as per routine clinical practice to include its individual components based on the investigator's judgement.
5. Assessments to be performed at specified time after End of infusion. The End of infusion is defined as when the infusion of IMP is completed/stopped (i.e,. before rinsing if applicable). For example, 1h post-dose means 1 hour after end of infusion.
6. If any of the treatment discontinuation criteria apply, the patient should be discontinued from treatment.
7. In case of supply issues with leucovorin, or to adhere to local standard of care practice, other approved similar folinates may be used under the supervision of an experienced clinician.
8. Assessment to be performed only if there is no prior confirmatory CT scan or clinical or radiological progression and no new anti-cancer therapy started prior to the visit

Table 4 Assessment schedule for Post-treatment follow-up period/End of study visit (i.e. upon discontinuation of mitazalimab treatment)

**Activity/Visit**	**Post-treatment follow-up/End of study visit**
**Time after End of treatment visit**	**3 months, and thereafter every 3rd month until end of study** ±14 days
**Disease/Survival status** (Section 10.14)	X1
**Subsequent cancer-related therapy** (Section 10.15)	X1
**CT scan/Tumor response evaluation** (Section 10.12)	X
**Study withdrawal criteria** (Section 9.3)	X2,3

1. Disease/Survival status and subsequent cancer-related therapy may be followed up via the patient's medical records (as allowed by local regulations) or phone contact.
2. If any of the study withdrawal criteria in Section 9.3 apply, the patient will be withdrawn from the study
3. End of study visit will be performed 2 years after last patient in (LPI) or at time of disease progression or clinical deterioration if study treatment continues past the 2 years after LPI, i.e., there will be a variation of duration of the treatment follow up period for individual patients.

2 GENERAL INFORMATION

2.1 Sponsor

Alligator Bioscience AB Medicon Village
SE-223 81 Lund
Sweden
Tel. +46 (0)46 540 82 00

2.2 Protocol number and title of the study

Protocol No.: A-20-1013-C-03

Title: An open-label phase 1b/2 study assessing the safety and efficacy of mitazalimab in combination with chemotherapy in patients with metastatic pancreatic ductal adenocarcinoma

2.3 Contract Research Organization (CRO)

Theradex (Europe) Ltd (Theradex) 2nd Floor, The Pinnacle
Station Way, Crawley West Sussex, RH10 1JH United Kingdom
Tel. +44 (0)1293 510 319

2.4 Medical Monitor (CRO)

Dr. Jack Welch
Theradex (Europe) Ltd (Theradex) 2nd Floor, The Pinnacle
Station Way, Crawley West Sussex, RH10 1JH United Kingdom
Tel. +44 (0)7394563680

2.5 Signature authorization

The Clinical Operations Service provider Theradex will act as the Sponsor's representative.

2.6 Investigators and Institutions

The study will be conducted in Europe and planned countries are France and Belgium. Additional countries may be added.
In Part 1, 6 sites are expected to participate in the study. The number of sites could be increased to up to 15 sites for Part 2, dependent upon recruitment.

2.7 Characteristics of a well-conducted study

The following characteristics of an adequate and well-conducted study will be implemented:

1. The Investigators will be well qualified by scientific training and experience.
2. Detailed eCRFs will be completed for every patient.
3. Requirements for institutional ethics review as set forth by the appropriate Ethics Committee (EC), Title 21 CFR Part 56, the European Union Directive 2001/20/EC and its associated Detailed Guidance, European Union GCP Directive 2005/28/EC, the ICH GCP, Sections 3 and 4, and the terms of the Declaration of Helsinki (2013), will be followed.
4. Requirements for informed consent in accordance with institutional guidelines, FDA requirements as specified in Title 21 CFR Part 50, the European Union Directive 2001/20/EC and its associated Detailed Guidance, European Union GCP Directive 2005/28/EC, European Union GDPR 2016/679, the ICH GCP, Section 4.8, and the terms of the Declaration of Helsinki (2013), will be followed.
5. Safety data will be recorded and evaluated.
6. Routine monitoring visits will be conducted by the Sponsor's representative (Theradex) to ensure data accuracy.
7. Drug accountability will be strictly maintained.
8. This study will be conducted according to ICH GCP, the Clinical Study Protocol and applicable regulatory requirements.

3 BACKGROUND INFORMATION

3.1 Introduction to pancreatic ductal adenocarcinoma

Pancreatic ductal adenocarcinoma (PDAC) is a type of exocrine pancreatic cancer. It is the most common type of pancreatic cancer with 95 out of 100 (95%) of all pancreatic cancers. Pancreatic cancer is the fourth leading cause of cancer death in both the United States and the European Union [1]. The incidence of pancreatic cancer is highest in Europe (7.7 per 100,000 people) and North America (7.6 per 100,000 people) followed by Oceania (6.4 per 100,000 people) [2]. In the United States, estimated new cases in 2020 are 57,600 and estimated deaths are 47,0501. The incidence is slightly higher for men than women.

The five-year life expectancy for pancreatic cancer is about 5%, a number which has not changed over the last two decades. Surgical resection is the only curative modality, but at best a fifth of patients are considered operable, and even in these cases, the five-year survival is on the order of 20%. More than three-quarters of patients present with advanced disease, about half have distant metastases at diagnosis, with the remaining quarter having inoperable local disease. In time, most of these locally advanced cancers will also metastasize [3].

Consequently, there is a need for effective treatment of metastatic pancreatic cancer. Until the last decade, the standard first-line treatment for metastatic pancreatic cancer was gemcitabine, which yielded a median overall survival (mOS) of about 6 months. A major advance came in 2011, with the introduction of the FOLFIRINOX regimen, which yielded an objective response rate (ORR) of 31% and mOS of 11.1 months compared to gemcitabine monotherapy with an ORR of 9.4% and mOS of 6.8 months in the randomized study PRODIGE 4 with 342 patients enrolled [4]. Two years later, the MPACT study comparing gemcitabine plus nab-paclitaxel to gemcitabine also demonstrated superior activity of the combination with an ORR of 23% and mOS of 8.5 months relative to gemcitabine monotherapy with an ORR 7% and mOS 6.7 months [5].

3.2 Mitazalimab, a CD40 agonist

Mitazalimab (also known as JNJ-64457107 and ADC-1013) is an agonistic, human monoclonal (IgG1) antibody targeting CD40. The agent has been investigated for the treatment of advanced stage solid tumors in two Phase 1 studies: A-14-1013-C-01 (EudraCT No. 2014-004556-56) and JNJ-64457107CAN1001 (EudraCT No. 2016-000969-23).

---

1 Cancer facts and figures, American Cancer Society, 2020

3.3 Introduction to CD40

CD40 is a co-stimulatory receptor belonging to the tumor necrosis factor receptor (TNFR) superfamily [6]. CD40 is expressed in a multitude of cell types and can be detected on the surface of antigen presenting cells (APCs), including dendritic cells (DCs), B cells, and macrophages. In addition, CD40 is expressed on granulocytes, endothelial cells, smooth muscle cells, fibroblasts, and epithelial cells [6-8]. CD40 is also present on the membranes of a wide range of malignant cells, including non-Hodgkin and Hodgkin lymphomas, myeloma, and some carcinomas including those of the nasopharynx, bladder, cervix, kidney, and ovary [6, 9]. CD40 interacts with a single ligand, CD40L (or CD154), a transmembrane protein that is expressed by activated T cells, B cells, platelets, mast cells, macrophages, basophils, natural killer (NK) cells, and non-hematopoietic cells (smooth muscle cells, endothelial cells, and epithelial cells) [6, 7].

The molecular consequences of CD40 signaling depend on the cell type expressing CD40 and the microenvironment in which the CD40 signal is provided [10]. CD40 ligation and cross-linking is required for the adaptive immune response through the 'licensing' of APCs and especially DCs by inducing the up-regulation of costimulatory receptors and major histocompatibility complex molecules as well as the production of pro-inflammatory cytokines. Thus, CD40 is involved in the functional maturation of APCs and consequently the activation of antigen-specific T lymphocytes [11-13]. CD40 also plays a role in humoral immunity by activating resting B lymphocytes and by increasing their antigen-presenting function [10, 14]. Moreover, CD40 is involved in the induction of innate immunity through stimulation of cytotoxic myeloid cells such as NK cells, macrophages, and granulocytes [10, 14, 15].

3.4 Summary of non-clinical data

The CD40 agonistic properties of mitazalimab have been validated *in vitro* in human monocyte-derived DC cultures. Ligation of CD40 by mitazalimab leads to up-regulation of activation markers on the surface of DCs, such as CD80 and CD86, and the release of cytokines such as IL-12. Mitazalimab has also been demonstrated to polarize tumor associated macrophages (TAMs) isolated from human prostate and ovarian tumor samples, from a more immune-suppressive phenotype into a more immune-inflammatory phenotype by upregulation of e.g., CD83. The agonistic effect of mitazalimab is critically dependent on the binding of the Fc-portion of the antibody to Fcγ-receptors (FcγR). The ability of mitazalimab to activate DCs (i.e., upregulate CD86) was significantly decreased when an aglycosylated variant of mitazalimab which contained a N297Q mutation in the γ chain and thus that does not bind to FcγR was used. The CD40 agonistic effect was again restored upon cross-linking of this aglycosylated mitazalimab variant with an anti- human IgG, conclusively demonstrating that mitazalimab is dependent on FcγR-cross-linking for optimal activity. Mitazalimab showed immune-mediated and antibody-dependent cell-mediated cytotoxicity (ADCC)-dependent anti-tumor efficacy *in vivo* in human tumors transplanted into NSG mice and human CD40-transgenic (hCD40tg) mice. Moreover, mitazalimab induced a T-cell dependent and tumor-specific immunological memory to bladder cancer cells in hCD40tg mice,

with immunity to tumor re-challenge for at least 5 months. Mitazalimab has also demonstrated a synergisitically enhanced effect on tumor growth and survival when combined with other immunotherapies such as immune checkpoint inhibitors (e.g., PD-1), vaccination or chemotherapy (e.g., mFOLFIRINOX) in experimental tumor models in hCD40tg mice.
For additional and detailed information of non-clinical data, see the mitazalimab Investigator's Brochure.

3.5 Summary of clinical data

3.5.1 Mitazalimab single-agent studies

3.5.1.1 First-in-human study with intratumoral administration of mitazalimab

Study A-14-1013-C-01 (NCT02379741) was a first-in-human, multicenter, non-randomized, open-label, multiple ascending dose escalation, phase-1 study of mitazalimab (ADC-1013) in patients with advanced stage solid malignancies.
Mitazalimab was administered as an intratumoral bolus injection into the same tumor lesion (intratumoral administration) every 14 days in 18 patients and as an intravenous (IV) infusion over 2 hours every 14 days in 5 patients.
No maximum tolerated dose (MTD) was identified. Increases in liver enzymes or bilirubin were observed in 9 of 18 patients given the intratumoral administration and 3 of 5 patients given IV administration. Only two of the patients had normal liver enzymes and bilirubin values at baseline. Two dose-limiting toxicities (DLTs) were observed, one patient with grade 3 abdominal pain and one patient with grade 3 cholecystitis, both received the intratumoral administration. The best response was stable disease (SD) in one patient with renal cell carcinoma (400 µg/kg intratumoral). No patients with pancreatic cancer were enrolled in this study.

3.5.1.2 Dose escalation study with intravenous administration of mitazalimab

Study 64457107CAN1001 (NCT02829099) was a multicenter, non-randomized, open-label, ascending dose escalation phase 1 study with mitazalimab (JNJ-64457107) conducted in patients with advanced stage solid tumors. Mitazalimab was administered every 14 days as an IV infusion at doses ranging from 75 µg/kg to 2000 µg/kg with corticosteroid included in the premedication, and up to 1200 µg/kg without corticosteroid.
In total, 95 patients were exposed to mitazalimab. One patient remains on treatment. No MTD was identified. Two patients experienced a DLT, one patient with headache lasting 5 days and one patient had grade 3 liver enzyme elevation together with grade 2 bilirubin elevation. Seven pancreatic cancer patients were enrolled in the study, one of whom experienced SD for more than 6 months. A partial response (PR) was observed in a patient with renal cell cancer, who received fourteen cycles (i.e., 28 doses) at the 1200 µg/kg dose level and was on study for 9.2 months [16].

3.5.1.3 Pharmacodynamic activity

The pharmacodynamic biomarker data in Study 644577107CAN1001 were studied after the first mitazalimab administration and are consistent with CD40-mediated immune cell activation. Following IV administration of mitazalimab, margination of B cells was observed, with dose-dependent B-cell recovery, at all doses tested. NK cells and T cells also decreased in the peripheral blood following infusion of mitazalimab at all doses tested, with the exception of the lowest dose (75 µg/kg). The levels of both cell types were fully recovered by study Day 8.

CD40 receptor occupancy was assessed on B cells and shown to be dose-dependent. CD40 remained engaged through Day 8 post mitazalimab administration.

Peripheral levels of MCP-1, IP-10 and MIP-1B chemokines peaked 1-4 hours post-infusion, consistent with myeloid cell activation. Other chemokines (such as MIP-1α and IL-8) and cytokines (such as IFN-γ, TNF-α and IL12p70) were also observed, but to a lesser extent. IL-6 levels, which can be highly induced in subjects with cytokine release syndrome, were not elevated following infusion of mitazalimab.

The pattern of biomarker changes was consistent with the proposed mechanism of mitazalimab as a CD40 agonist.

3.5.2 CD40 agonists administered to patients with pancreatic cancer

3.5.2.1 Selicrelumab

Selicrelumab (CP-870.893, RO7009789) is a CD40 agonistic monoclonal antibody, with an IgG2 format that activates CD40 independently from cross-linking with Fcγ receptors [17]. It is a general belief with agonistic CD40 antibodies that systemic delivery with cross-linking independent antibodies may increase the risk for toxicities which can reduce the therapeutic window [18]. Selicrelumab has been evaluated in combination with gemcitabine in patients with previously untreated pancreatic cancer [19]. The combination was well tolerated up to 0.2 mg/kg. One DLT, a cerebrovascular accident, occurred at the 0.2 mg/kg dose level. Four patients out of 22 had a PR, no complete responses (CR) were observed (response rate 19%). Cytokine release syndrome in relation to the selicrelumab infusion was observed in 20 of the 22 patients, one event was grade 3, all other events were grade 1 or 2. Immune activation with an increase in inflammatory cytokines, increase in expression of co-stimulatory molecules on B cells, and transient depletion of B cells were observed in all patients. Liver enzyme elevations occurred in approximately 2/3 of the patients, all were grade 1-2, while hyperbilirubinemia occurred in a few patients.

3.5.2.2 APX005M

APX005M is a CD40 agonistic monoclonal antibody, with a mutated IgG1 format for improved binding to FcγR2b, which depends on cross-linking with FcγRs for its function. APX005M has a profile similar to mitazalimab, with the aim to improve efficacy and safety compared with previous CD40 antibodies. APX-005M is in clinical development for several malignancies.

Early clinical data with APX005M in combination with gemcitabine plus nab-paclitaxel with or without nivolumab in patients with previously untreated metastatic pancreatic cancer were presented in 2019, see Table 5. Gemcitabine plus nab-paclitaxel was given 3 times, nivolumab was given twice and APX005M once in each 28-days cycle. Immune profiling of the PBMCs demonstrated remodeling of the myeloid compartment in response to treatment, with rapid activation of DCs in most patients. Analysis of circulating mutant KRAS DNA showed marked and rapid decrease with therapy. Preliminary efficacy data from 24 patients showed encouraging data for the combinations and the randomized study continues [20].

Table 5 Preliminary efficacy data for APX005M in combination with gemcitabine plus nab-paclitaxel (Gem-NabPac) with or without nivolimab (Nivo)

	Gem-NabPac+ APX005M 0.1 mg/kg (N=6)	Gem-NabPac+ APX005M 0.3 mg/kg (N=6)	Gem-NabPac/ Nivo+APX005M 0.1 mg/kg (N=6)	Gem-NabPac/ Nivo+APX005M 0.3 mg/kg (N=6)	Total (N=24)
**Complete Response**	0	0	0	0	0
**Partial Response**	3 (50%)	2 (33%)	4 (67%)	4 (67%)	13 (54%)
**Stable Disease**	3 (50%)	3 (50%)	1 (17%)	2 (33%)	9 (38%)
**Progressive Disease**	0	1 (17%)	0	0	1 (4%)
**Not Evaluable**	0	0	1 (17%)	0	1 (4%)

3.6 Scientific rationale

3.6.1 Combination of mitazalimab with chemotherapy in patients with pancreatic ductal adenocarcinoma

Combination chemotherapy for metastatic pancreatic cancer has been pushed to the brink of tolerability, but survival outcomes remain poor. The next advances in this line of therapy will most likely not come from intensification of cytotoxic chemotherapy but through addition of agents that synergize with existing therapies to achieve better anti-cancer effect, particularly agents that engage the immune system.

Pancreatic cancer is classified as immunologically “cold” compared to other tumors characterized by immune infiltrates. The desmoplastic stroma that forms around pancreatic cancer, in addition to functioning as a physical barrier to chemotherapy, is host to tumor fibroblasts and suppressive myeloid cells that dampen the immune response in the tumor microenvironment [21]. Moreover, because pancreatic cancer harbors relatively few nonsynonymous mutations compared to other

cancers, the tumors are characterized by low expression of tumor neoantigens. These two factors contribute to the lack of activity seen with checkpoint inhibitors in pancreatic cancer. Unlike cancers where checkpoint inhibitors have been effective in restoring T-cell function that has been suppressed through binding of receptors such as CTLA-4 and PD-1, there seems to be a lack of initial engagement in pancreatic cancer. For checkpoint inhibitors to become useful in this type of cancer, something has to “light the fuse” [22].
CD40 agonists fit this niche, with potential to augment the response to chemotherapy agents and to spark an effective immune response. Studies performed in murine models of pancreatic cancer have demonstrated that administration of CD40 agonists after chemotherapy results in cancer cell death, tumor shrinkage, and extended survival [23]. Studies have demonstrated that response to CD40 agonists requires the presence of Batf3+ DCs, CD8+ T cells, and INF-γ signaling, but other elements of the “inflammasome” such as TLR4, STING, and myeloid cell interactions are not involved. The interpretation is that antigen released by immunogenic cell death due to chemotherapy is being taken up by stimulated DCs, and that through cytokine signaling, effector T cells are activated resulting in anti-cancer effects [24]. The second and subsequent administrations of mitazalimab will be given 2 days after the start of each mFOLFIRINOX combination. In case of shift to gemcitabine plus nab-paclitaxel the second and subsequent administrations of mitazalimab will be given 2 days after start of the first and third gemcitabine plus nab-paclitaxel in each cycle. This window is based on synergy in mouse models [23, 24] and clinical precedence [18, 19].

Other than inducing a T-cell dependent anti-tumor immune response, CD40 agonists have been found to re-direct tumor infiltrating macrophages from the immunosuppressive M2 type to the tumoricidal M1 type [25, 26]. In pancreatic tumor models, CD40 activation and re-direction of macrophages resulted in degradation of fibrosis in the stroma surrounding the tumor which enhanced the efficacy of the chemotherapeutic agents [27]. An effect which may foster development of a more immunogenic microenvironment in the tumor. Hence, in the current clinical study a mitazalimab infusion will be administered prior to initiation of chemotherapy to push the macrophages towards the M1 subtype in order to enhance the sensitivity of the tumor for the chemotherapy. The first dose of mitazalimab will be given one week before the start of the first chemotherapy. The interval has been chosen based on the half-life of mitazalimab and the data from in vivo mouse model where hepatotoxicity was observed when a CD40 agonist was given just 2 days before gemcitabine [28].
The two dose levels chosen for mitazalimab in this study, 450 µg/kg and 900 µg/kg, are planned to be administered in combination with mFOLFIRINOX in Part 1. No MTD of mitazalimab was identified in the phase 1 dose escalation study 64457107CAN1001 (NCT02829099). In that study, binding of mitazalimab appeared to be saturated at 1200 and 2000 µg/kg. However, maximal biological effect, identified as cytokine and chemokine responses as well as immune cell responses in the circulation, occurred at doses below 1200 µg/kg. Since the classical dose-response

relationship does not necessarily apply to agonistic antibodies [29], the clinically relevant doses were therefore chosen as 450 µg/kg and 900 µg/kg.

3.7 Potential risks associated with mitazalimab treatment and chemotherapy and mitigations

Described below are selected toxicities for study treatment, including mitazalimab and chemotherapies, that may be employed in this protocol as well as mitigation guidelines. This information should be considered to supplement dose modification guidelines to mFOLFIRINOX and gemcitabine plus nab-paclitaxel in APPENDIX 2 and APPENDIX 3, respectively, and guidance provided in the respective summary of product characteristics (SmPCs) for the marketed products and the IB for mitazalimab.

Infusion-related reactions/cytokine release syndrome:

Stimulation of CD40 is associated with increased cytokines in the blood as well as upregulation of costimulatory receptors as a result of activation of antigen presenting cells. Hence, cytokine release symptoms are expected in relation to mitazalimab exposure. To mitigate severe symptoms, medication with antihistamine, acetaminophen and leukotriene inhibitor in the days before and after mitazalimab infusion are included. For more details on premedications to be given in connection with mitazalimab infusion, please see Section 8.6 Handling of infusion-related reactions. A rate-controlled infusion, with a low initial rate that can be increased if tolerated, is also mandated to mitigate severe symptoms.

Hypersensitivity reactions with either mFOLFIRINOX or gemcitabine/nab-paclitaxel are not common. If minor symptoms such as flushing, skin reactions, dyspnea, hypotension, or tachycardia occur, the infusion can be temporarily interrupted. However, severe reactions, such as hypotension requiring treatment, dyspnea requiring bronchodilators, angioedema, or generalized urticaria, require immediate discontinuation of study drug administration and aggressive symptomatic therapy. Patients who develop a severe hypersensitivity reaction should not be re- challenged, see Section 8.6 for handling of infusion related reactions.

Hepatobiliary toxicity:

Both mitazalimab and the chemotherapy included in the treatment are associated with parameters indicative of hepatic injury with elevation of liver enzymes and/or bilirubin increase. The patients will be monitored with regular blood tests to surveille these laboratory parameters. If hepatobiliary toxicity is observed, study treatment should be held to allow evaluation of alternative causes, e.g., biliary obstruction/stent malfunction (see APPENDIX 7 Drug induced liver injury (DILI)). Depending on the degree and duration of hepatic injury, dose modifications of one or more of the

chemotherapy components can be indicated (see APPENDIX 2 for mFOLFIRINOX and APPENDIX 3 for gemcitabine plus nab-paclitaxel modifications, as applicable).

Cytopenia:

Mitazalimab is associated with transient decrease of B cells in peripheral blood, likely due to redistribution from blood to tissue after administration of mitazalimab. However, this has not been associated with long term decreases or decline in immunoglobulin levels.
Treatment with chemotherapy is often associated with cytopenia. Depending on the cell lineage, affected patients may suffer from different symptoms. Neutropenia is associated with an increased risk for infections, which may be severe and even fatal. Thrombocytopenia may lead to increased risk for bleeding. Anemia can cause fatigue, dyspnea and tachycardia. The patients will be monitored with regular blood tests to surveille these laboratory parameters. For dose modifications of the chemotherapy due to hematopoietic toxicity, refer to APPENDIX 2 for mFOLFIRINOX and APPENDIX 3 for gemcitabine plus nab-paclitaxel.
Patients treated with mFOLFIRINOX may receive primary prophylaxis with G-CSF. If the gemcitabine plus nab-paclitaxel chemotherapy regimen is used, use of G-CSF is permitted, but its use is at investigator discretion. High grade neutropenia and febrile neutropenia should be managed according to institutional standards, e.g., with regard to antibiotic coverage.
Blood component transfusion and administration of erythropoietin are also authorized at investigator discretion.

Non-neutropenic sepsis risk:

Patients treated with gemcitabine plus nab-paclitaxel are at increased risk for non-neutropenic sepsis. In the phase 3 study at the first occurrence of fever ≥38.5°C (regardless of neutrophil count), institution of ciprofloxacin (500 mg orally, twice daily) - or amoxicillin/clavulanate (Augmentin®, 500 mg orally, 2-3 times daily) in patients with allergy to fluoroquinolones - should be initiated. On their first visit, patients should be provided with enough ciprofloxacin (or the alternative antibiotic) for use at home, and they should be instructed to begin taking it when they first record a temperature of ≥38.5°C. They should also immediately contact their physician for guidance on where to go for blood counts to be evaluated for sepsis as soon as possible. Hospitalization or evaluation in the emergency room may be required depending on the clinical presentation.

Diarrhea:

In general, chemotherapy-induced diarrhea results from the death of rapidly dividing enterocytes and consequent loss of absorptive function coupled with inflammation and an altered gastro- intestinal osmotic gradient resulting in secretory loss of fluids and electrolytes. Treatment is generally supportive and in severe or persistent cases can require hospitalization, with administration of parental fluids. When coincident with neutropenia, patients are at elevated risk

for infectious complications and coverage with broad-spectrum antibiotics according to institutional standard practices may be considered.
In particular, the irinotecan component of mFOLFIRINOX causes both acute (within 24 hours) and delayed (2 to 14-day post-administration) diarrhea, see APPENDIX 2.

Mucositis:

Cytotoxic chemotherapy targets rapidly dividing cells such as the gastrointestinal epithelium including the oral mucosa, resulting in tissue damage and inflammation, mucositis. This can be severe enough to limit intake and compromise nutrition, so symptomatic supportive care and nutritional monitoring is critical. Of the components in mFOLFIRINOX, 5-fluorouracil (5-FU) is the strongest contributor to mucositis.

Neuropathy:

Both taxane and platinum-containing chemotherapy are associated with nerve damage attributed primarily to altered microtubular transport interfering with axonal function. Typically, this affects peripheral neurons, more distant than proximal and more often sensory than motor. The sensory neuropathy may begin with paraesthesia but can progress to impair activities of daily living. The toxicity is cumulative over time and may persist or even worsen after dose modification.
Predisposing factors include medical history of peripheral neuropathy, diabetes, advanced age or prior exposure to neurotoxic drugs. The main contributor to neuropathy in the gemcitabine/nab-paclitaxel regimen is the paclitaxel component, whereas oxaliplatin is responsible for most of the neurotoxicity in the mFOLFIRINOX regimen.
In addition to this generalized peripheral neuropathy, a specific acute neuropathy, laryngopharyngeal dysesthesia, occurs infrequently in patients within hours of treatment with oxaliplatin. This can manifest as an uncomfortable sensation in the area of the laryngopharynx and patients can experience dyspnea and anxiety.
For patients with a history of oxaliplatin-associated laryngospasm, it is recommended to administer a tranquilizer prior to oxaliplatin infusion and to infuse over six hours.

Hand and foot syndrome:

Palmar-plantar erythrodysesthesia is characterized by redness, swelling, and pain on the palms of the hand and/or soles of the feet; occasionally, blistering may occur. The main contributor to this relatively common dermal toxicity for the mFOLFIRINOX regimen is the 5-FU component, whereas paclitaxel is the main contributor for the gemcitabine plus nab-paclitaxel regimen. Treatment is supportive.

Nausea:

mFOLFIRINOX is considered to be a moderately emetogenic regimen, and prophylaxis with anti-emetics is recommended per investigator's discretion and institutional standard. Gemcitabine plus nab-paclitaxel is considered to be of low emetogenic potential. Note, that use of corticosteroids should be restricted when given as prophylaxis, see Section 8.9.2.
Nausea as a symptom for infusion-related reaction has been observed with mitazalimab therapy and anti-emetics are part of the pre- and post-medication in relation to mitazalimab administration, see Section 8.9.1.

Pulmonary Toxicity:

Interstitial pneumonitis was seen four times more frequently in patients treated with gemcitabine/nab-paclitaxel than in the gemcitabine-alone control arm of the phase 3 study that established this regimen for metastatic pancreatic cancer. During study participation, patients should be carefully monitored for signs and symptoms of pneumonitis (i.e., episodes of transient or repeated dyspnea with unproductive persistent cough or fever) and, if observed, immediate clinical evaluation and timely institution of appropriate management (emphasizing the need for corticosteroids if an infectious process has been ruled out as well as appropriate ventilation and oxygen support when required). Study drug administration should be permanently discontinued upon making a diagnosis of interstitial pneumonitis.

Extravasation

If chemotherapy leaks into tissue surrounding the intended blood vessel, severe local tissue necrosis can occur; this has been reported for irinotecan and oxaliplatin. If this occurs, stop the infusion immediately. Leaving the needle or catheter in place, aspirate as much of the agent as possible. Apply ice to the infiltrated area for 15-20 minutes every 4 to 6 hours for a 72-hour period. Apply local corticotherapy. Follow the site closely and do not hesitate to require a surgical consultation in case of doubt.

3.8 Benefit-risk assessment

As reviewed above, the vast majority of patients with pancreatic cancer cannot be cured but will at some point develop metastatic disease. The two best options for first line chemotherapy in fit patients are the FOLFIRINOX or gemcitabine plus nab-paclitaxel regimens, which yield median overall survivals of about 11 and 8.5 months, respectively. However, both regimens cause substantial toxicity, so any prospect for building on these therapies will need to introduce minimal additional toxicity.
Adding antibody-based therapies directed at immune stimulation is an attractive strategy for patients with pancreatic cancer. Unlike most cytotoxic drugs, antibodies are highly specific and while antibodies as a class are associated with some adverse events such as infusion related

reactions, for the most part, antibody-related toxicities do not overlap with cytotoxic drug toxicities. As an immunologically "cold" tumor with poor immune cell infiltration, the host immune response contributes less to anti-tumor response in pancreatic cancer than other cancers. While traditionally chemotherapy may have reached the point of diminishing returns, the potential for immune activation is largely untapped at this point.

The main mode of action of mitazalimab is activation and induction of differentiation of dendritic cells, leading to increased tumor antigen presentation and elaboration of cytokines which transactivate cytotoxic T cells. This initial step is attenuated in pancreatic cancer tumors, likely related to low tumor mutational burden and relatively few tumor neoantigens. However, this observation raises the possibility of synergistically coupling tumor antigen release due to treatment with cytotoxic chemotherapy with mitazalimab treatment to capitalize on the presence of tumor antigens liberated by cell killing. A second mode of action of mitazalimab is direct stimulation of tumor macrophages and it has been shown in animal models that treatment with CD40 agonist antibodies alone (i.e., without the addition of chemotherapy) results in pancreatic cancer tumor regression mediated by activated tumor macrophages.

However, the present study builds upon the primary mode of action of mitazalimab, targeting antigen presentation and transactivation of the adaptive immune system. CD40 agonist antibodies given after chemotherapy in animal models of pancreatic cancer have demonstrated tumor regression and improved survival [24]. More importantly, other CD40 agonist antibodies have entered clinical trials in combination with chemotherapy, and preliminary data by APX-005M has demonstrated improved tumor response rate for combination therapy versus gemcitabine-nab- paclitaxel monotherapy in patients with pancreatic cancer [20].

This proof-of-mechanism speaks to the credibility of the current approach. However, due to structural differences, each CD40 agonist antibody will have its own safety profile alone and in combination with chemotherapy. Mitazalimab, administered both intratumorally and intravenously as monotherapy has been previously studied in two phase 1 clinical trials, which demonstrated an acceptable safety profile and identified the doses chosen in the current study.

The intention of this study is to maximize clinical benefit by combining mitazalimab with the most effective chemotherapy regimen available, namely the mFOLFIRINOX combinations. The higher objective response rate for FOLFIRINOX likely translates to greater cell kill and antigen release. Additionally, since the median Overall Survival with mFOLFIRINOX is greater than that of Gemcitabine/nab-paclitaxel, the best chance of extending survival with the combination may lie with the FOLFIRINOX combination.

However, this protocol recognizes that there is a risk that the combination of mitazalimab and chemotherapy may elicit emergent toxicities, including toxicities not observed with either mitazalimab or the chemotherapy regimen alone. Additionally, the protocol is designed to monitor for signals of additive or synergistic hepatotoxicity, as liver transaminase elevation has been seen to some degree with mitazalimab and both chemotherapy regimens independently. Drug-induced

liver injury is an adverse event of special interest in this protocol, and aggressive management guidelines are provided to mitigate this risk.

Any combinational toxicity that is encountered could lead to worse clinical outcome than would be experienced by patients taking the standard chemotherapeutic regimen alone because such toxicities could undermine the dose-density of already effective regimens, e.g., by requiring dose modification or delay of the regimen or some component of the regimen.

Part 1 of this study focuses on finding a dose combination with an acceptable risk profile, starting with mFOLFIRINOX, but if necessary for safety reasons, also considering the gemcitabine plus nab-paclitaxel combination. Part 2 of the protocol continues this risk assessment, but is also designed to provide a preliminary assessment of anti-tumor activity. Part 2 uses a two-stage design to minimize the number of patients exposed to drug, while this assessment is made, thereby limiting risk.

The current study enrolls adult patients diagnosed with metastatic pancreatic cancer. This patient population has a very poor prognosis, and a need for new therapeutic options. The potential benefit of combining mitazalimab with standard first-line chemotherapy is expected to outweigh the treatment-related risks, and overall, these combinations are believed to have an acceptable risk/benefit profile.

4 OBJECTIVES AND ENDPOINTS

4.1 Objectives and endpoints Part 1 (Phase 1b)

OBJECTIVES	ENDPOINTS
**Primary**	
To determine the recommended Phase 2 dose (RP2D) of mitazalimab in combination with chemotherapy	• Incidence of DLTs
**Secondary**	
To assess the safety and tolerability of mitazalimab in combination with chemotherapy	• Type, frequency and severity of adverse events (AEs)
To assess the immunogenicity of mitazalimab	• Detection and characterization of anti-drug antibody (ADA) titers in serum
To assess pharmacokinetics (PK) of mitazalimab after single and repeated administrations	• PK parameters will include Cmax, Tmax, and AUC(0-T). Additional parameters may be calculated depending on data obtained
To assess the clinical activity of mitazalimab in combination with chemotherapy (i.e., anti-tumor activity as per RECIST v. 1.1 guideline)	• Objective response rate (ORR)<br>• Best Overall Response (BOR), with response categories CR, PR, SD, and PD<br>• Duration of response (DoR)<br>• Duration of SD<br>• Disease control rate<br>• Time to next anti-cancer therapy
To assess survival outcomes following repeated administrations of mitazalimab in combination with chemotherapy	• Progression-free survival (PFS)<br>• Overall survival (OS)
**Exploratory**	
To characterize the impact of mitazalimab in combination with chemotherapy on the immune response both systemically and in the tumor microenvironment	• Change in cytokines and chemokines<br>• Quantification of changes in innate and adaptive immune cell populations and immune cell activation status<br>• Change in gene expression and immune signatures, assessing differentially expressed genes and gene signatures for specific immune cells
To characterize the impact on CA19-9 and ctDNA of mitazalimab in combination with chemotherapy	• Change in CA19-9<br>• Change in ctDNA and differentially expressed genes of interest

4.2 Objectives and endpoints Part 2 (Phase 2)

OBJECTIVES	ENDPOINTS
**Primary**	
To assess the clinical activity of mitazalimab in combination with chemotherapy (i.e., anti-tumor activity as per RECIST v. 1.1 guideline)	• Objective response rate (ORR)
**Secondary**	
To assess the clinical activity of mitazalimab in combination with chemotherapy (i.e., anti-tumor activity as per RECIST v. 1.1 guideline)	• Best Overall Response (BOR), with response categories CR, PR, SD, and PD<br>• Duration of response (DoR)<br>• Duration of SD<br>• Disease control rate<br>• Time to next anti-cancer therapy
To assess survival outcomes following repeated administrations of mitazalimab in combination with chemotherapy	• Progression-free survival (PFS)<br>• Overall survival (OS)
To assess the safety and tolerability of mitazalimab in combination with chemotherapy	• Type, frequency and severity of AEs
To assess the immunogenicity of mitazalimab	• Detection and characterization of anti-drug antibody (ADA) titers in serum
To assess pharmacokinetics (PK) of mitazalimab after single and repeated administrations	• PK parameters will include Cmax, Tmax, and AUC(0-T). Additional parameters may be calculated depending on data obtained
**Exploratory**	
To characterize the impact of mitazalimab in combination with chemotherapy on the immune response both systemically and in the tumor microenvironment	• Change in cytokines and chemokines<br>• Quantification of changes in innate and adaptive immune cell populations and immune cell activation status<br>• Change in gene expression and immune signatures, assessing differentially expressed genes and gene signatures for specific immune cells
To characterize the impact on CA19-9 and ctDNA of mitazalimab in combination with chemotherapy	• Change in CA19-9<br>• Change in ctDNA and differentially expressed genes of interest

5 STUDY DESIGN

5.1 Study overview

This is a phase 1b/2, open-label, multicenter study designed to evaluate the safety, tolerability, and efficacy of intravenously administered mitazalimab in combination with the chemotherapy mFOLFIRINOX (or gemcitabine plus nab-paclitaxel). A total of up to 80 patients diagnosed with metastatic pancreatic ductal adenocarcinoma are planned to be enrolled in the study. For eligibility criteria see Section 7 STUDY POPULATION.

The study consists of two parts as illustrated in Figure 1 Study overview.

Part 1 (Phase 1b) of the study is a safety run-in with a dose escalation of mitazalimab in combination with mFOLFIRINOX to determine a safe and tolerable dose (i.e., the RP2D) of mitazalimab in combination with mFOLFIRINOX for Part 2. Part 1 follows a Bayesian optimal interval (BOIN) design with at least 3 patients enrolled at each dose level and at least 6 patients to be evaluated at the RP2D.

In **Part 2 (Phase 2)** of the study, patients will be administered the RP2D of mitazalimab obtained in Part 1 to assess clinical efficacy and further safety of mitazalimab in combination with mFOLFIRINOX.

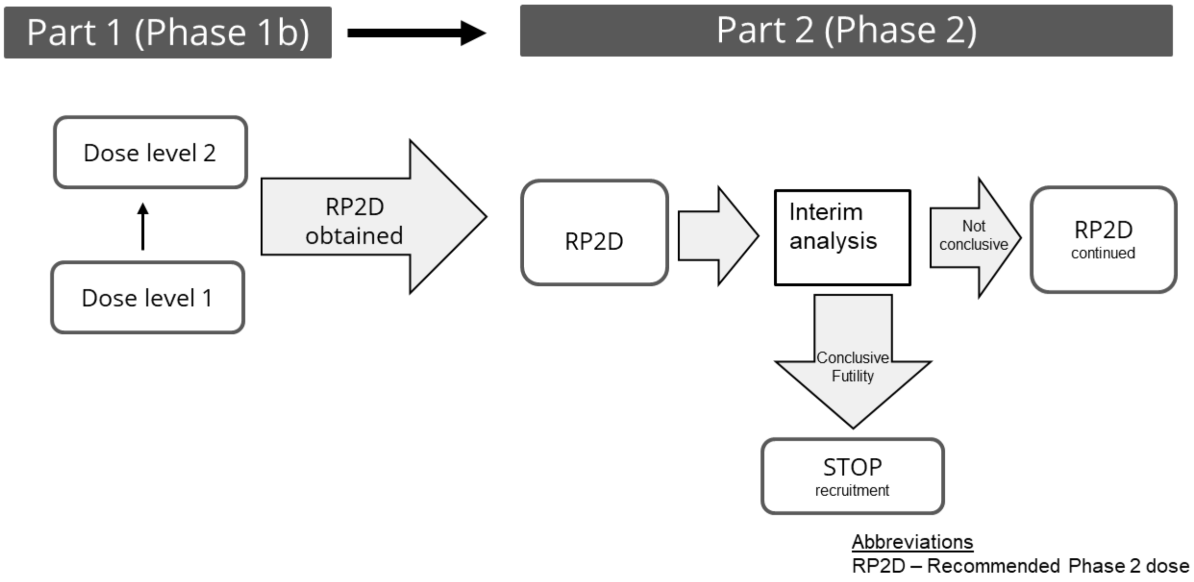

Figure 1 Study overview

In part 1 there will be staggered dosing with at least 11 days between the first dose of mitazalimab administered to the first patient and the first dose of mitazalimab administered to the second patient at each dose level, see Section 8.5 Staggered dosage of patients.

All patients will be monitored for at least 4 hours after the end of the first infusion of mitazalimab, and for at least 2 hours after the second mitazalimab infusion. If infusion-related reactions have not been observed at the latest infusion (the 2nd or later infusion), the monitoring of the patient can be reduced to 1 hour for subsequent infusions. The monitoring period may be prolonged for all patients by the Data Review Committee (DRC) based on emerging safety data, see Section 6.
Referring to Figure 2 below, in case the lowest planned dose level of mitazalimab (450 µg/kg) in combination with mFOLFIRINOX in Part 1 is found not safe or tolerable, mFOLFIRINOX will be exchanged to gemcitabine plus nab-paclitaxel, and the same mitazalimab dose escalation schedule will be followed. If the lowest planned dose level of mitazalimab in combination with gemcitabine plus nab-paclitaxel in Part 1 is found not safe or tolerable, the study will terminate.

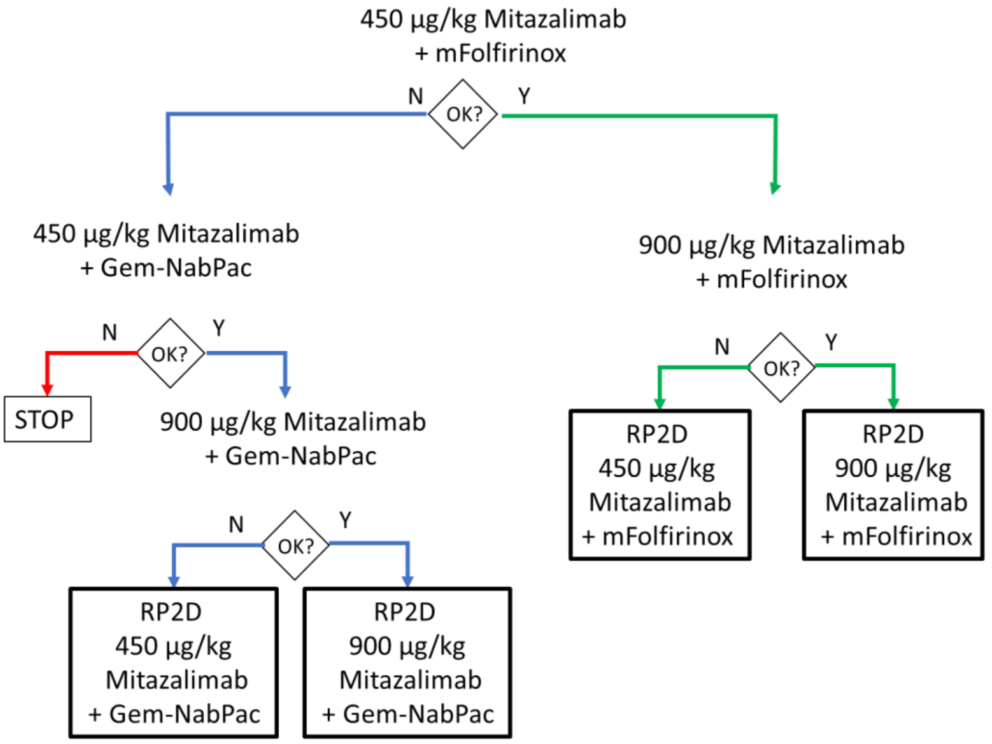

Figure 2 Dose escalation schedule

When the last patient in Part 1 has completed the DLT evaluation period, and data has been collected and reviewed by the DRC, a decision to continue to Part 2 can be taken.
Up to 60 patients are planned to be enrolled in Part 2.

An interim analysis to allow stopping for futility will be performed when efficacy data is available for a total of 23 evaluable patients at RP2D (Part 1 and Part 2). The interim analysis will be based on objective response rate (ORR) observed for all patients dosed at the mitazalimab RP2D.
The interim analysis will include efficacy data up to 17 weeks of study treatment (8 cycles for mFOLFIRINOX and 4 cycles for gemcitabine plus nab-paclitaxel). Depending on the outcome of the

interim analysis, it will be decided if the study should stop further enrollment or continue to enroll 37 additional patients. For details on rational and procedure for the statistical analysis, see Section 12 STATISTICS.
The dosage schedule and visit assessment schedules are the same for Part 1 and Part 2 in the study for each respective combination of chemotherapy, see Section 5.3.1 Doses and dosage schedule and Section 1 VISIT ASSESSMENT SCHEDULE for mitazalimab and mFOLFIRINOX treatment combination. Dosage schedule for mitazalimab and gemcitabine plus nab-paclitaxel treatment combination (if applicable) can be found in APPENDIX 3.

5.2 Duration of treatment

The anticipated duration of study treatment with mitazalimab and chemotherapy for each patient is approximately 6 months, (i.e. 12 treatment cycles of mitazalimab in combination with mFOLFIRINOX and 6 treatment cycles for mitazalimab in combination with gemcitabine plus nab-paclitaxel) as long as no treatment discontinuation or study withdrawal criteria are met, see Section 9.1 and Section 9.3. Thereafter, patients who, in the opinion of the investigator, are deriving clinical benefit (without confirmed disease progression) may continue the study treatment until disease progression, unacceptable toxicity or until any other treatment discontinuation or study withdrawal criteria are met (see Section 9.1 and Section 9.3), whichever comes first.

5.3 Doses, dosage schedule and dose escalation in Part 1

Both mFOLFIRINOX and gemcitabine plus nab-paclitaxel are well-established and effective chemotherapy regimens for treatment of advanced pancreatic cancer. A central principle in the dose-finding portion of the study is to never compromise the intensity of this standard chemotherapy. If an adverse event is considered related to chemotherapy based on the investigators' clinical judgement, treatment will be adjusted in the standard manner as summarized in the dose adjustment sections in APPENDIX 2 (for mFOLFIRINOX) and APPENDIX 3 (for gemcitabine plus nab-paclitaxel) to this protocol. However, when an unexpected toxicity or toxicity of unexpected intensity with regard to these chemotherapies is encountered during the escalation portion of this study and the investigator considers that this toxicity could undermine the effectiveness of the chemotherapy, the investigator will discontinue mitazalimab. If this occurs during the DLT period of the study and the adverse event is not considered a DLT, the patient will be replaced.

5.3.1 Doses and dosage schedule

Two dose levels, 450 µg/kg and 900 µg/kg, of mitazalimab are planned to be administered in combination with mFOLFIRINOX in Part 1.

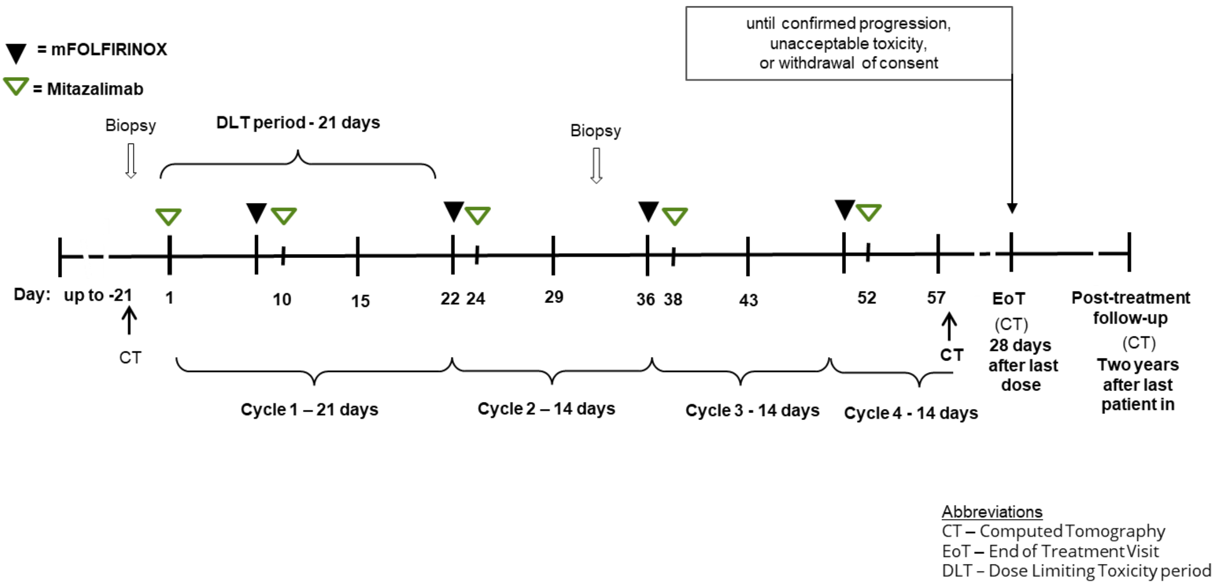

Figure 3 Dosage schedule for mitazalimab and mFOLFIRINOX

During the first treatment cycle (the first 21 days) for each patient, mitazalimab will be administered on Day 1 and Day 10 and mFOLFIRINOX infusion will start on Day 8. During the following 14-day treatment cycles, mFOLFIRINOX will be administered on Day 1 and mitazalimab on Day 3 of each cycle, see Figure 3.

In case the number of DLTs for a dose level fulfill the stop criteria, see Table 6 Escalation rules based on number of patients with DLTs, at the lowest dose level of mitazalimab, the chemotherapy mFOLFIRINOX will be exchanged to the chemotherapy gemcitabine plus nab-paclitaxel, as illustrated in Figure 2.

If changing the chemotherapy from mFOLFIRINOX to gemcitabine plus nab-paclitaxel, see dosage schedule in APPENDIX 3.

5.3.2 Dose escalation procedure

The dose escalation in Part 1 follows a BOIN design with at least 3 patients evaluable for DLT per dose level. According to the BOIN design each time a cohort of patients has been completed, a decision is made whether the next cohort should remain on the same dose, escalate to the next higher dose level or de-escalate to the next lower dose level, according to the decision rules presented in Table 6 below. A range of 3-9 patients may be included at each dose level. In this

study, the de-escalation from 450 µg/kg mitazalimab in combination with mFOLFIRINOX will be a change of chemotherapy to gemcitabine plus nab-paclitaxel as previously described.

Table 6 Escalation rules based on number of patients with DLTs

**Decision, based on the number of patients with DLTs (*NDLT*)**	**Number of patients evaluable for DLT at the current dose level**						
	**3**	**4**	**5**	**6**	**7**	**8**	**9**
Escalate, if ***NDLT*** ≤	0	0	1	1 a	1	1	2
Remain on dose- level, if ***NDLT*** =	1	1	NA	2	2	2	3
De-escalate, if ***NDLT*** ≥	2	2	2	3	3	3	4
Disallow dose level, if ***NDLT*** ≥	3	3	4	4	5	5	5

NA = Not applicable

a Stop if higher dose level has been evaluated and the number of DLTs on that higher dose level lead to a de-escalation

The dose escalation will be determined by the DRC upon review of safety data obtained during the DLT evaluation period as well as available data beyond the DLT evaluation period from all patients in the study. Dose escalation is allowed when the DRC has defined the dose and the dosage schedule for the next dose level.

Based on available data, the DRC can decide to introduce intermediate dose levels in between the pre-planned dose level as well as to change the dosing frequency.

For more details regarding the DRC, see Section 6 Data Review Committee.

5.3.3 Dose-limiting toxicity (DLT) evaluation period

Mitazalimab in combination with mFOLFIRINOX: The DLT evaluation period is defined as the time from the first dose of mitazalimab (Day 1) until Day 21 in the first treatment cycle *(*Cycle 1).

Mitazalimab in combination with gemcitabine plus nab-paclitaxel: The DLT evaluation period is defined as the time from the first dose of mitazalimab (Day 1) until Day 28 within the first treatment cycle of 35 days *(*Cycle 1).

This period will enable the DRC to evaluate for early safety of mitazalimab together with the chemotherapy combination.

5.3.4 Dose-limiting toxicity (DLT) criteria

A DLT is defined as one of the following toxicities (i.e., drug-related AEs) graded by CTCAE version 5.0:

- Grade 4 neutropenia lasting for more than 7 days
- Grade 4 thrombocytopenia lasting for more than 7 days
- Grade 4 infusion-related reaction

- Grade 3 infusion-related reaction which does not resolve to a lower grade within 24 hours after onset
- Grade 4 AST, ALT and/or bilirubin
- Grade 3 AST, ALT and/or bilirubin not resolved to a lower grade within 7 days
- Any grade ≥3 non-hematologic toxicity except for:
    - Laboratory abnormalities that have no clinical consequence and resolve to grade ≤2 within 14 days (including electrolyte abnormalities responding to medical intervention
    - Fatigue
    - Nausea and/or vomiting lasting less than 48 hours
    - Diarrhea which does resolve to less than grade 3 by the end of the DLT period

The same DLT criteria will apply for mitazalimab in combination with mFOLFIRINOX and gemcitabine plus nab-paclitaxel.
A DLT will be considered related to study treatment unless there is a clear, well-documented, alternative explanation for the AE. AEs that meet the above criteria but occur after the DLT evaluation period will not be defined as DLTs, unless the onset of the event is during the DLT evaluation period. The event should be reported as AEs/Serious adverse events (SAEs), as applicable, see Section 11 ADVERSE EVENT REPORTING.
In case of suspicion of a DLT, the Investigator must inform the Medical Monitor/Sponsor immediately. The DLT will be confirmed by the Medical Monitor/Sponsor and a decision must be taken by the Sponsor regarding if an *ad hoc* DRC meeting is required.
Patients who are tolerating study treatment will not have to discontinue dosing prematurely due to the occurrence of DLTs in another patient in the same cohort, unless decided by the DRC. A patient that experiences a DLT may continue study treatment as decided by the DRC.

5.3.5 Intra-patient dose escalation

Intra-patient dose-escalation for mitazalimab will not be applied for this study.

5.4 Doses and dosage schedule in Part 2

The RP2D of mitazalimab in combination with mFOLFIRINOX or gemcitabine plus nab-paclitaxel, as obtained in Part 1, will be administered to all patients in Part 2. Modifications in administration of mFOLFIRINOX or gemcitabine plus nab-paclitaxel, are allowed as described in APPENDIX 2 and APPENDIX 3 respectively.
The same dosage schedule for mitazalimab in combination with each respective chemotherapy applies for both Part 1 and Part 2 of the study, see Section 5.3.1 for dosage schedule for mitazalimab in combination with mFOLFIRINOX and APPENDIX 3 for dosage schedule for mitazalimab in combination with gemcitabine plus nab-paclitaxel.

6 DATA REVIEW COMMITTEE (DRC)

Safety reviews and oversight of study data will be performed during Part 1 (Phase 1b) of the study under the direction of a Data Review Committee (DRC).

The DRC will be composed of the Investigators, Sponsor's medical representative and the Medical Monitor. Additional independent experts may be invited to participate in relevant DRC meetings. During dose escalation, the DRC will assess the safety and tolerability after each dose level before deciding on the subsequent dose level or if to change chemotherapy from mFOLFIRINOX to gemcitabine plus nab-paclitaxel. The DLT evaluation period will be 21 days for mFOLFIRINOX and 28 days for gemcitabine and nab-paclitaxel combination.

The DRC will review data obtained during the DLT evaluation period for all patients treated at the dose level. The DRC will also review available data beyond the DLT evaluation period for all patients in the study (i.e., both from the dose level under review and from previous dose level) before a decision on the next dose level can be taken.

The DRC will review the following data as a minimum:

- Demographics and Medical history
- AEs
- Laboratory assessments
- Vital signs
- Concomitant medications, surgical and medical procedures
- Other data as applicable, e.g., immunogenicity, PK and cytokines

The DRC will, based on the data listed above, decide whether to:

- Continue dose escalation, and decide the next higher mitazalimab dose level
- Expand number of patients at a given mitazalimab dose level
- Change the chemotherapy from mFOLFIRINOX to gemcitabine plus nab-paclitaxel
- Decrease the mitazalimab dose for the next dose level
- Adjust the study treatment dosage schedule
- Introduce a pre-dose of mitazalimab (see Section 8.4)
- Prolong the duration of infusion of mitazalimab
- Prolong the post-infusion monitoring period after mitazalimab administration
- Postpone the next dose level
- Prolong the DLT evaluation period based on emerging data
- Decide to halt further enrolment/dosing if treatment emergent toxicity

In case of a safety concern during dosage of a cohort, the DRC must convene before any further dosing within the cohort. The DRC can decide that staggered dosage should apply for subsequent patients in a cohort and/or can introduce extra safety measurements as applicable (see Section 8.5 Staggered dosage of patients). The DRC can also take decisions regarding premedication.

The DRC may convene *ad hoc* upon the development of emerging safety findings. An *ad hoc* DRC meeting can be on initiative from Medical Monitor/Sponsor based on the Medical Monitor/ Sponsors regular surveillance of the safety data and/or upon request from an Investigator.

The decision of the DRC will be taken in consensus and will be communicated in writing to the Investigators/sites. In case of a decision to stop dosing, this will be communicated in writing to the investigational site.

The functions and responsibilities of the DRC will be further described in the DRC Data Review Plan.

7 STUDY POPULATION

7.1 Inclusion criteria

A patient is eligible to be included in the study if all the following criteria apply:

1. Has provided written informed consent
2. Is ≥18 years of age at the time of signing the informed consent form (ICF)
3. Has an Eastern Cooperative Oncology Group (ECOG) performance status of 0 or 1
4. Has a diagnosis of previously untreated metastatic pancreatic ductal adenocarcinoma (histologically documented)
5. Has measurable disease per RECIST v. 1.1
6. Has not received previous chemotherapy for pancreatic ductal adenocarcinoma
7. Has not received prior abdominal radiotherapy (except for palliative radiotherapy to non-target lesions)
8. Has a life expectancy of ≥ 3 months
9. Has acceptable hematologic laboratory values defined as:
    a. Neutrophils ≥ 1.5 x 109/L without growth factor stimulation within 3 weeks prior to the blood test
    b. Platelets ≥100 x 109/L
    c. Hemoglobin ≥6.2 mmol/L (~100 g/L) (may be after transfusion)
10. Has acceptable clinical chemistry laboratory values defined as:
    a. Bilirubin ≤1.5 x ULN (biliary drainage is permitted)
    b. AST ≤3 x ULN (irrespective of hepatic metastases)
    c. ALT ≤3 x ULN (irrespective of hepatic metastases)
    d. Creatinine ≤1.5 x ULN or glomerular filtration rate (GFR) of ≥45 mL/min (see APPENDIX 4 for calculation of GFR)
    e. INR ≤1.5 x ULN
    f. Albumin ≥28 g/L
11. For women of childbearing potential2:
    a. Has a negative highly sensitive serum (β-human chorionic gonadotropin [β-hCG]) pregnancy test at screening
    b. Is willing to use highly effective contraception methods (defined in APPENDIX 5) during study treatment and for at least six months thereafter

---

2 A woman is considered of childbearing potential (WOCBP), i.e. fertile, following menarche and until becoming post- menopausal unless permanently sterile. Permanent sterilisation methods include hysterectomy, bilateral salpingectomy and bilateral oophorectomy. A postmenopausal state is defined as no menses for 12 months without an alternative medical cause. A high follicle stimulating hormone (FSH) level in the postmenopausal range may be used to confirm a post-menopausal state in women not using hormonal contraception or hormonal replacement therapy. However in the absence of 12 months of amenorrhea, a single FSH measurement is insufficient.

12. Fertile men must practice effective contraceptive methods (i.e. surgical sterilization, or a condom used with a spermicide) during study treatment and for at least six months thereafter
13. Is willing to comply with all study procedures

7.2 Exclusion criteria

A patient is excluded if any of the following criteria apply:

1. Has other types of non-ductal tumor of the pancreas, including endocrine tumors or acinar cell adenocarcinoma, cyst adenocarcinoma and ampullary carcinoma
2. Has other current cancer or history of cancer in the prior 3 years before signing the ICF other than *in situ* cervical cancer, or basal cell or squamous cell carcinoma treated with local excision only
3. Has known CNS metastases or carcinomatous meningitis
4. Has contraindication to any constituent of study treatment (mitazalimab and applicable chemotherapy)
5. Has a history of chronic diarrhea, inflammatory disease of the colon or rectum, or unresolved partial or complete intestinal obstruction
6. Has a history of myocardial infarction within 12 months of the first administration of mitazalimab, uncontrolled angina pectoris, unstable cardiac arrhythmias, or congestive heart failure of New York Heart Association class II or greater
7. Has QTc >450 msec
8. Has uncontrolled intercurrent illness, including active infection
9. Has a known history of HIV, hepatitis B or active hepatitis C infection
10. Is a female patient who is pregnant or nursing
11. Has received attenuated vaccine within 28 days before the first dose of study treatment
12. Any condition that, in the opinion of the Investigator, would place the patient at increased risk or preclude the patient's compliance with the study
13. Participates in another investigational drug or device study with any intervention within the previous 4 weeks prior to first dose of mitazalimab

Additional exclusion criteria only applicable for mFOLFIRINOX treatment:

14. Has received prior treatment with irinotecan or platinum-containing chemotherapy
15. Has pre-existing peripheral neuropathy greater than grade 1
16. Has known Gilbert's disease
17. Has known genotype UGT1A1 * 28 / * 28
18. Has known fructose intolerance (malabsorption)
19. Has complete dihydropyrimidine dehydrogenase (DPD) deficiency

Additional exclusion criteria only applicable for gemcitabine plus nab-paclitaxel treatment:

14. Has a history of slowly progressive dyspnea and unproductive cough, or of conditions such as sarcoidosis, silicosis, idiopathic pulmonary fibrosis, pulmonary hypersensitivity, pneumonitis or multiple allergies
15. Has a history of Peripheral Artery Disease (eg, claudication, Leo Buerger's disease)
16. Has a history of connective tissue disorders (eg, lupus, scleroderma, arteritis nodosa)

7.3 Restrictions

The following restrictions apply during the study:

1. Patients should abstain from taking non-allowed medication during the study according to Section 8.9.7.1, Contraindicated medication and medication with interactions.

7.4 Patient registration

Patients fulfilling all inclusion and exclusion criteria will be registered on the study by using an Interactive Web Response System (IWRS) automated patient registration system (see the Study Operations Manual for specific instructions).
Patient registration is controlled by accrual limits assigned to the active cohort. Registration is automatically stopped once the accrual limit has been reached.
Changes to accrual limits or cohort escalation are determined by the DRC. It will only be possible for sites to register patients to a dose level which has been approved by the DRC.

7.5 Screen failures and rescreening

Screen failures are defined as patients who consent to participate in the study but do not meet all inclusion and exclusion criteria.
The minimal set of information to be reported for screen failures, and for patients who withdraw consent prior to first dose, includes demography, assessment of eligibility criteria (reason for screen failure) and any untoward medical occurrences caused by a protocol-mandated intervention (e.g., invasive procedures such as biopsies) that meet the criteria for being serious.
A screen-failure patient can only be rescreened once. All screening procedures must be repeated, including re-assessment of all eligibility criteria with the following exceptions:

- CT scanning does not need to be repeated if the scan is performed within 28 days before the first dose of mitazalimab.

- Biopsy does not need to be repeated, unless the patient had a new cancer-related treatment (for other indications than PDAC) in between the initial screening and rescreening.
- DPD test does not need to be repeated

In addition, patients that miss all doses of study treatment (mitazalimab and chemotherapy) within the first treatment cycle (Cycle 1) are allowed to be rescreened, see Section 8.8 Handling of delayed and missed doses.
In case of rescreening, the patient will get a new screening number. If the ICF has been updated since signing of the first ICF, the patient will need to reconsent and sign the new ICF for the study before screening can start.

8 TREATMENT

8.1 Investigational Medicinal Product (IMP) - Mitazalimab

The IMP mitazalimab is an agonistic human monoclonal (IgG1) antibody targeting CD40, developed for cancer immunotherapy.

8.1.1 Manufacturing

Mitazalimab is manufactured by Biogen (USA) according to GMP. The product is manufactured using a stable Chinese Hamster Ovarian (CHO) cell line in a fed batch bioreactor. The product does not contain any components of animal origin and has been reviewed to be safe from an adventitious agent perspective.

8.1.2 Formulation, packaging and labelling

Mitazalimab is supplied as a lyophilized cake in single-use glass vials. It must be reconstituted using sterile water for injection (WFI) prior to use. Each vial requires the addition of 2 mL sterile WFI to fully dissolve the cake. Each vial contains 40 mg mitazalimab, and the resulting concentration after reconstitution will be 20 mg/mL. The exact concentration will be stated on the primary vial.

The packaging, labelling and distribution to the clinical sites will be performed by Fisher Clinical Services (FCS). The packaging and labelling will be done at FCS site in Allentown, Pennsylvania USA and the distribution to the sites will be done by FCS in Germany according to Good Manufacturing Practice (GMP) under the responsibility of the Sponsor.

For further details regarding formulation, packaging and labelling, please refer to the Mitazalimab Investigational Medicinal Product Dossier (IMPD).

8.1.3 Handling, preparation and storage

The mitazalimab vials must be stored refrigerated at the local pharmacy at a temperature of 2-8ºC and protected from light in a secure area according to local regulations. The vials must not be used after the expiry date.

The preparation of mitazalimab solution for infusion will be carried out at the local pharmacy according to instructions in the Pharmacy Manual.

After reconstitution, mitazalimab is to be diluted in 5% dextrose in water. The prepared mitazalimab solution for infusion should be kept at room temperature protected from light in a secure area according to local regulations. The mitazalimab solution for infusion must be infused within 16 hours from the reconstitution of the lyophilized cake.

For detailed guidance on study drug preparation, handling and storage, please refer to the Pharmacy Manual.

8.2 Drug accountability procedures

The Investigator or designated study personnel must maintain an accurate record of dispensed mitazalimab in a Drug Accountability Log according to the Pharmacy Manual.
The Drug Accountability Log will be reviewed by the Clinical Research Associate (CRA) during monitoring visits and at the End of Trial.
The log must be forwarded to the Sponsor at the End of Trial.

8.3 Administration of mitazalimab

Mitazalimab will be administered intravenously during a 2-hour rate-controlled infusion. Weight at baseline will be used to calculate the mitazalimab dose throughout the study. If the pre-dose assessment of weight assessed in the beginning of each treatment cycle changes >10% from baseline, the mitazalimab dose must be recalculated. Detailed instruction on mitazalimab administration is given in a separate document "Instruction for intravenous administration of mitazalimab".
All patients will be monitored for at least 4 hours after the end of the first infusion of mitazalimab, and for at least 2 hours after the second mitazalimab infusion. If infusion-related reactions have not been observed at the latest infusion (the 2nd or later infusion), the monitoring of the patient can be reduced to 1 hour for subsequent infusions. The monitoring period may be prolonged for all patients by the DRC based on emerging safety data, see Section6 Data Review Committee.
Pre- and post-medication to be given in connection with mitazalimab administration is described in Section 8.9.1.

8.4 Mitazalimab dose modifications

The dose and dosing frequency of mitazalimab for an individual patient may be modified in the following ways:

- The dose may be reduced due to an AE:
    - If an adverse event is possibly, probably or definitely related to mitazalimab and results in more than two weeks of treatment delay, the dose of mitazalimab will be reduced by 50%. If the dose has already been reduced once, mitazalimab treatment will stop
    - If an adverse event is serious or greater than grade 2 and is possibly, probably or definitely related to mitazalimab, mitazalimab will be held until the AE resolves to the greater of the baseline grade or grade 1 or less; at that time mitazalimab may be resumed at the investigator's discretion either at the previous dose, or if not already dose reduced, at a 50% dose reduction; the investigator may also elect to forego rechallenge

- A pre-dose may be introduced due to an AE, e.g., infusion-related AEs (see Section 8.6 Handling of Infusion-Related Reactions)

8.5 Staggered dosage of patients

In Part 1 (Phase 1b) of the study, there will be at least 11 days between the first mitazalimab dose administered to the first patient and the first mitazalimab dose administered to the second patient at each dose level (i.e., staggered dosage). This allows ample time for observation of AEs and communication between sites and Sponsor in case of AEs, e.g., infusion-related reactions to mitazalimab. It also allows for the Sponsor to take any required actions.

After the 11 days, the CRO will contact the sites by telephone to discuss the treated patient and ascertain if any safety concerns have been observed after dosing. This information will be communicated to the Medical Monitor/Sponsor. If there are no safety concerns, in the opinion of the Investigator and the Medical Monitor/Sponsor, the subsequent patients can start treatment at the same dose level. This will immediately be communicated to the Investigator(s) via e-mail. In this way, the dosing of subsequent patients at a dose level will be strictly regulated.

In case of any safety concerns, a DRC meeting is required before any further dosing at the indicated dose level, see Section 6 Data Review Committee.

8.6 Handling of infusion-related reactions

If clinically significant symptoms of an infusion-related reaction occur, the infusion should be temporarily stopped (interrupted). Upon recovery, the infusion should be resumed at 50% of the rate at which the reaction occurred for at least 30 minutes. If there is no reccurrence of clinically significant symptoms after 30 minutes, the infusion rate can be increased according to the original infusion plan as described in Section 8.3 Administration of mitazalimab. Lower infusion rates may be selected if clinically indicated.

Depending on the time of occurrence and the severity of the reaction, the Investigator may consider administering supportive medication, e.g., antihistamines, acetaminophen or corticosteroids, in addition to the mandatory pre- and post-medications described in Section 8.9.1.

If severe infusion-related reactions are observed, the DRC may introduce changes to the mitazalimab infusion.

If AEs which are considered related to the infusion, e.g., infusion-related reactions, are not well controlled by the above schedule, the DRC can decide to introduce a pre-dose of mitazalimab on the day prior to first administration. The pre-dose will be a maximum of 10% of the full doses. A pre-dose could be set forth for all patients enrolled after the decision by the DRC.

Cytokine release syndrome may be indistinguishable from infusion-related reactions if the symptoms occur in relation to the infusion. Cytokine release syndrome may also have many symptoms that resemble an infection or even sepsis. Cytokine release syndrome as reported with CD3 targeting antibodies or CAR T cell infusion has not been observed with mitazalimab.
The preferred term when reporting reactions considered related to the infusion will be infusion-related reaction rather than cytokine release syndrome.
Infusion-related reactions of grade 2 or higher should be reported as AESIs according to Section 11.1.4 Adverse events of special interest (AESIs), and Section 11.2.5 Reporting of SAEs and AESIs.

8.7 Handling of impaired liver parameters

Hepatic injury is defined by the following alterations of liver parameters:

- For patients with normal liver function (ALT, AST and bilirubin within normal limits) at baseline an elevation of AST and/or ALT >3-fold ULN combined with an elevation of bilirubin >2-fold ULN measured in the same blood draw sample.
- For patients with impaired liver function at baseline, i.e., above ULN for AST or ALT, an elevation of AST and/or ALT >5-fold ULN combined with an elevation of total bilirubin >2-fold ULN measured in the same blood draw sample.

If laboratory values consistent with hepatic injury as defined above are observed the following laboratory tests must be repeated within 48 to 72 hours: ALT, AST, and bilirubin (total and direct). If ALT and/or AST >3 fold ULN combined with an elevation of total bilirubin >2-fold ULN are confirmed (if normal values at baseline/screening), or ALT and/or AST >5-fold ULN combined with an elevation of total bilirubin >2-fold ULN are confirmed (if elevated values at baseline/screening) these results must be reported to the Investigator, Medical Monitor, and to the Sponsor as soon as possible.
If hepatobiliary toxicity is observed, treatment should be held to allow evaluation of alternative causes, e.g., biliary obstruction/stent malfunction. Other causes may need to be followed up according to APPENDIX 7 Drug-induced Liver Injury (DILI).
Increased AST or ALT of grade 3 or higher, and bilirubin of grade 2 or higher should be reported as AESIs according to Section 11.1.4 Adverse events of special interest (AESIs), and Section 11.2.5 Reporting of SAEs and AESIs.

8.8 Handling of delayed and missed doses

This section describes handling of delayed or missed doses. For handling of changes in dosing during infusion, please refer to Section 8.4 and Appendix 2 and 3 for dose modifications of mFOLFIRINOX and gemcitabine plus nab-paclitaxel respectively.

In the first treatment cycle, Cycle 1, if the first dose of mitazalimab cannot be given (i.e., protocol Day 1), treatment should be aborted and the patient should be considered for rescreening if applicable, see Section 7.5 Screen failures and rescreening. Otherwise, the intention is to preserve the dose-intensity of standard chemotherapy as much as possible, but to avoid giving chemotherapy any earlier than five days after first dose of mitazalimab. Also, mitazalimab should not be dosed more frequently than once a week. Consequently, if chemotherapy is delayed, mitazalimab should be similarly delayed to maintain the fixed relationship of administering mitazalimab two days after the start of chemotherapy infusion.

In Cycle 2 and subsequent cycles, if mitazalimab is delayed more than a day, it should be given one week later than scheduled (±1 day). Note that the next cycle should similarly be pushed back by one week.

If mitazalimab is further delayed the dose should be omitted and considered missed. The visit at which the dose should have been administered will be considered a missed visit (unless some assessments have been performed at that visit) and the patient will be scheduled for the next visit according to Section 1 VISIT ASSESSMENT SCHEDULE.

If no mitazalimab dose has been administered in a 4 week period due to mitazalimab-related toxicity, the patient should discontinue treatment and perform the End of treatment visit (see Table 2/Table 3 for combination with mFOLFIRINOX and Table 21 in APPENDIX 3 for combination with gemcitabine plus nab-paclitaxel) and continue to post-treatment follow up (see Table 4 for combination with mFOLFIRINOX and Table 21 in APPENDIX 3 for combination with gemcitabine plus nab-paclitaxel). Any delayed or missed dose as well as the reason for delayed/missed dose should be recorded in the eCRF as well as in the medical source documents.

8.9 Non-Investigational Medicinal Products

8.9.1 Mitazalimab pre- and post-medication

Pre- and post-medications are to be given in connection with each mitazalimab administration. The premedication will start 3 days prior to mitazalimab infusion. The mandatory pre- and post-medications are listed in Table 7 below and includes type of medication and timing of intake in relation to mitazalimab infusion.

Antihistamine H1 and leukotriene inhibitor will be given as premedication, starting 3 days prior to mitazalimab administration, and may be given at the site or provided to the patient at a prior visit for intake in the patient's home. Patients will not be allowed to take any pre-medications prior to screening assessments are completed, and the patient has been confirmed to be eligible for participation in the study.

Information about intake of premedication before dosing of mitazalimab should be recorded in the eCRF, including name of premedication, dose and day/time of intake.

Table 7 Pre- and post-medication to be administered together with mitazalimab

Type of medication	Agent and dose	Schedule
Premedication:		
Antihistamine (H1)	E.g., Cetirizine 10 mg PO	Daily morning intake starting 3 days prior to start of mitazalimab infusion. On day of dosing 1-3 hours prior to start of mitazalimab infusion.
Leukotriene inhibitor	Montelukast 10 mg PO	Daily morning intake starting 3 days prior to start of mitazalimab infusion. On day of dosing 1-3 hours prior to start of mitazalimab infusion.
Antihistamine (H2)	E.g., Famotidine 40 mg PO (optional)	At least 30 minutes prior to start of mitazalimab infusion.
Antipyretic	E.g., Acetaminophen 650-1000 mg PO	At least 30 min prior to start of mitazalimab infusion.
Antiemetic	E.g., Ondansetron 8 mg PO or IV (optional)	At least 30 minutes prior to start of mitazalimab infusion.
Post-medication:		
Leukotriene inhibitor	Montelukast 10 mg PO	Morning intake on the two following days after mitazalimab administration.
Antihistamine (H1)	E.g., Cetirizine 10 mg PO	Morning intake on the 2 following days after mitazalimab administration.

8.9.2 mFOLFIRINOX

The mFOLFIRINOX used in this study is comprised of the constituents oxaliplatin, leucovorin, irinotecan and 5-fluorouracil (5-FU) and is considered standard of care for first line treatment of advanced pancreatic cancer by the European Society of Medical Oncology (ESMO) and the National Comprehensive Cancer Network (NCCN) based on evidence from large phase 3 trials.
The original "full" FOLFIRINOX regimen defined in ACCORD 11/0402, a 342-patient phase 3 study, consisted of a 400 mg bolus of 5-FU followed by 2400 mg/m2 5-FU over 46 hours, 400 mg/m2 leucovorin, 85 mg/m2 oxaliplatin, and 180 mg/m2 irinotecan. Over the last decade, it has become common practice to administer less intense versions of this regimen, known collectively as mFOLFIRINOX, to reduce side effects. Current practice guidelines place mFOLFIRINOX regimens on par with full FOLFIRINOX as a preferred regimen for first line treatment of patients with metastatic pancreatic cancer and good performance status3.

---

3 NCCN Clinical Practice Guidelines in Oncology. Pancreatic Adenocarcinoma. Version 1.2021. (https://www.nccn.org/professionals/physician_gls/pdf/pancreatic.pdf, accessed 30 OCT 2020).

Based on phase 2 trials, other variations of FOLFIRINOX have been recognized as acceptable alternatives that retain comparable clinical efficacy with an improved side effect profile. These have included, for example, regimens reducing all components by 20% [30] or reducing the 5-FU bolus to 300 mg and reducing the irinotecan to 135 mg/m2 [31].

Other clinical trials that have sought to combine novel agents with FOLFIRINOX have opted for similar modifications of FOLFIRINOX, for example, no 5-FU bolus was administered in a study combining FOLFIRINOX with Hedgehog inhibitor IPI-926 [32]. In the AVENGER 500 trial combining FOLFIRINOX with CPI-613, the 5-FU component was kept at full strength including the bolus, but oxaliplatin was reduced to 65 mg/m2 and irinotecan to 140 mg/m2 [33].

The mFOLFIRINOX regimen employed in the current study is identical to the one used in the phase 3 PRODIGE 24 trial adjuvant pancreatic study and differs from the original FOLFIRINOX regimen in that the 5-FU bolus is omitted and the irinotecan dose is 150 mg/m2 [34].

Participants should, prior to study treatment, be informed to seek advice about donation and cryopreservation of germlines because of the possibility of irreversible infertility caused by treatment with the constituents in the mFOLFIRINOX regimen.

mFOLFIRINOX will be supplied/obtained according to Clinical Study Agreements and in accordance with local guidelines.

The constituents comprising mFOLFIRINOX will be stored and handled according to package inserts and stored in a secure place under appropriate storage conditions.

8.9.2.1 Administration of mFOLFIRINOX

Mitazalimab will be administered in combination with the chemotherapy regimen mFOLFIRINOX as first choice. mFOLFIRINOX is comprised of the constituents oxaliplatin, leucovorin, irinotecan and 5-FU. The recommended parameters for timing and sequence of infusion of constituents of mFOLFIRINOX are described in Table 8 below.

Leucovorin is one of several folinates that can be used in combination with 5-FU in cytotoxic therapy. Leucovorin potentiates 5-FU’s inhibition of thymidylate synthase, enhancing 5-FU’s antimetabolic activity.

Alternative folinates to leucovorin may be utilized based on local availability including but not limited to calcium folinate, calcium levofolinate, disodium folinate and disodium levofolinate. Dosing based on folinic acid as defined in the relevant SmPC’s should be followed. Where leucovorin is referenced within this Clinical Study Protocol it is acknowledged that similar approved folinates can be used.

Variations in the administration are permitted, provided drug dosing and modification guidelines in APPENDIX 2 are followed.

Weight and height at baselines will be used to calculate the doses of mFOLFIRNOX constituents. Doses should be re-adjusted if the patient's body surface area (BSA) changes by >10% from baseline. If the patient's BSA changes by $\leq$10% no adjustment is necessary unless the site has a standard procedure to adjust doses based upon current BSA according to institutional guidelines.
Table 8 mFOLFIRINOX administration

**mFOLFIRINOX regimen**			
**Agent**	**Dose**	**Route**	**Schedule**
Oxaliplatin	85 mg/m2	IV, 2h infusion	Every 14 days, (Day 8 in Cycle 1, and Day 1 in subsequent cycles)
Leucovorin	400 mg/m2	IV, 2h infusion	
Irinotecan	150 mg/m2	IV, 90 minute infusion started 30 minutes after end of the leucovorin infusion	
5-FU	2400 mg/m2	46-48 hour infusion IV	Every 14 days (Day 8-10 in Cycle 1 and Day 1-3 in subsequent cycles)

Recommended infusion timing and sequence:

- Oxaliplatin IV over 2 hours immediately followed by
- Leucovorin or similar approved folinates over 2 hours
- Irinotecan administered over 90 minutes (starting 30 minutes after start of the leucovorin infusion), followed by
- 5-FU infusion over 46-48 hours

If one of the mFOLFIRINOX constituents is stopped, the other constituents may be given.

Pre- and post-medication to be given in connection with mFOLFIRINOX administration is described in Section 8.9.3 below.
In case mFOLFIRINOX is not deemed safe and tolerable in combination with the lowest planned dose level of mitazalimab (450 µg/kg) by the DRC during Part 1, mFOLFIRINOX will be exchanged to gemcitabine plus nab-paclitaxel. The dosage schedule and dosing modifications allowed for gemcitabine plus nab-paclitaxel are described in APPENDIX 3.

8.9.3 mFOLFIRINOX pre- and post-medications

Pre- and post-medications are to be given in connection with each mFOLFIRINOX administration. The suggested premedication will start an hour prior to administration of oxaliplatin, the first constituents of mFOLFIRINOX. The suggested pre- and post-medications are listed below.

The suggested premedication can include one or more of the following medications given 30-60 minutes prior to the infusion:

- NK1-receptor antagonist, e.g., Aprepritant, 125 mg PO, 60 min prior to infusion and can be continued during days with chemotherapy. Aprepitant is a cytochrome 2C9 inducer and may inactivate some oral contraceptives. Therefore, women of child-bearing potential should use an alternative means of contraception if Aprepitant is administered.
- 5-HT3 receptor antagonist, e.g., Ondansetron, 8 mg PO, 30 min prior to infusion and can be continued during days with chemotherapy
- Corticosteroid, e.g., dexamethasone 8 mg IV or PO, 30 min prior to infusion.

The post-medication can include the following medications

- G-CSF, e.g., Neulasta, 6 mg sc, on the fourth day following start of mFOLFIRINOX regimen (at least 24 h after the end of the continuous 5-FU iv infusion).

Alternative pre- and post-medications to mFOLFIRINOX are allowed and can be discussed with the Medical Monitor.
Information about intake of premedication before dosing of mFOLFIRINOX should be recorded in the eCRF, including name of premedication, dose and day/time of intake.

8.9.4 Gemcitabine and nab-paclitaxel (if applicable)

Gemcitabine and nab-paclitaxel are considered standard of care for first line treatment of advanced metastatic pancreatic cancer by the European Society of Medical Oncology (ESMO) and the National Comprehensive Cancer Network (NCCN) based on evidence from large, randomized phase 3 trials.
Participants should, prior to study treatment, be informed to seek advice about donation and cryopreservation of germlines because of the possibility of irreversible infertility caused by treatment with gemcitabine or nab-paclitaxel.
Gemcitabine plus nab-paclitaxel will be supplied/obtained according to Clinical Study Agreements and in accordance with local guidelines.
Gemcitabine and nab-paclitaxel will be stored and handled according to package inserts and stored in a secure place under appropriate storage conditions

8.9.4.1 Administration of gemcitabine and nab-paclitaxel

Gemcitabine plus nab-paclitaxel will be given in combination with mitazalimab in case mFOLFIRINOX is found not viable. The recommended parameters for timing and sequence of infusion for gemcitabine and nab-paclitaxel, respectively, are listed in Table 9 Gemcitabine and nab-paclitaxel administration below. Variations in the administration are permitted, provided drug dosing and modification guidelines are followed, see APPENDIX 3.

Table 9 Gemcitabine and nab-paclitaxel administration

Agent	Dose	Route	Schedule
**Nab-paclitaxel**	125 mg/m2	IV	Day 8, 15 and 22 in Treatment cycle 1 (35-day treatment cycle) and Day 1, 8, 15 of subsequent treatment cycles (28-day treatment cycles)
**Gemcitabine**	1000 mg/m2	IV	

Recommended infusion timing and sequence:

- Nab-paclitaxel is infused over 30-40 min
- Gemcitabine is infused over 30 min, immediately after completion of nab-paclitaxel infusion

8.9.5 Gemcitabine and nab-paclitaxel pre- and post-medications

No pre- or post-medications are mandatory in connection with gemcitabine and nab-paclitaxel administration but allowed as judged by the Investigator.

8.9.6 Rescue therapy

Rescue medication in terms of an antidote to reverse the action of mitazalimab, mFOLFIRINOX, gemcitabine or nab-paclitaxel is not available.
Potential side effects of mitazalimab, mFOLFIRINOX, gemcitabine and nab-paclitaxel must be treated symptomatically.
Handling of treatment related reactions is described in Section 8.6 Handling of infusion-related reactions, Section 8.7 Handling of impaired liver parameters (mitazalimab) and in APPENDIX 2 (mFOLFIRINOX) and APPENDIX 3 (gemcitabine plus nab-paclitaxel).

8.9.7 Concomitant medication

All medications taken up to 14 days before the first administration of mitazalimab and all concomitant therapies administered during the study should be recorded on the Concomitant medication eCRF page.
The information to be recorded includes a description of the drug name or treatment, start date, stop date, dose, route, reason for use and, if applicable, the AE Number related to reason for use.
During the post-treatment follow-up, only subsequent cancer-related therapies will be collected and should be recorded on the concomitant medication/procedure eCRF page.

8.9.7.1 Contraindicated medication and medication with interactions

The following medications are either contraindicated or interact with the chemotherapy agents.

Contraindicated medication (not to be used)

General

- Warfarin or oral direct-acting anticoagulants (i.e., direct anti-thrombin and anti-factor X inhibitors). Use heparin-based anticoagulation instead.
- Pimozide (Orap®) and cisapride (Prepulsid®) are contraindicated due to risk ventricular arrythmia (torsades de pointes).
- Live vaccines are contraindicated due to risk of potentially fatal infection in immunocompromised patients.

Contraindicated concomitant medications must not be given.

Medication to be avoided or only used with caution due to interactions with mFOLFIRINOX

5-FU interaction

- Metronidazole as it enhances toxicity of 5-FU by decreasing the clearance of 5-FU.

Irinotecan interaction

- St. John's Wort, an alternative medicine, is contraindicated because it decreases the serum concentration of SN-38, the active metabolite of irinotecan.
- Irinotecan is metabolized by CYP3A4, therefore CYP3A4 inhibitors such as cimetidine, macrolide antibiotics (azithromycin, clarithromycin, erythromycin), fluoroquinolone antibiotics (ciprofloxacine, norfloxacin), azolated antifungal agents (fluconazole, ketoconazole, itraconazole), grapefruit juice, and the calcium channel blockers that inhibit CYP3A4 (verapamil, diltiazem, nifedipine) could increase the irinotecan toxicity.

Oxaliplatin interaction

- Phenytoin, which increases the risk for seizure in combination with oxaliplatin.

Folinate interaction

- Anti-epileptica, e.g., phenobarbital, primidone, phenytoin and succinimides may have diminished effect and hence increase the risk for seizures
- Folic acid antagonist, e.g., cotrimoxazole and pyrimethamine, may have reduced or completely neutralized effect.

Medication to be avoided or only used with caution due to interactions with gemcitabine and nab-paclitaxel treatment

- The metabolism of nab-paclitaxel is catalysed, in part, by cytochrome P450 isoenzymes CYP2C8 and CYP3A4. Therefore, caution should be exercised when administering paclitaxel concomitantly with medicines known to inhibit either CYP2C8 or CYP3A4 (e.g., ketoconazole and other imidazole antifungals, erythromycin, fluoxetine, gemfibrozil, clopidogrel, cimetidine, ritonavir, saquinavir, indinavir, and nelfinavir) because toxicity of

paclitaxel may be increased due to higher paclitaxel exposure. Administering paclitaxel concomitantly with medicines known to induce either CYP2C8 or CYP3A4 (e.g., rifampicin, carbamazepine, phenytoin, efavirenz, nevirapine) is not recommended because efficacy may be compromised because of lower paclitaxel exposures.

Prior to use of any medication with known interactions, the Investigators should discuss the use with the Medical Monitor, unless the urgency of the situation precludes such advance discussion; in that case, notification can be provided afterwards. In all cases, this decision should be documented.

8.9.7.2 Restricted medication

The use of corticosteroids should be restricted as much as possible in relation to mitazalimab administration due to the presumed mode of action of mitazalimab to stimulate the dendritic cells for antigen presentation and avoid compromising patient’s immune response. Therefore, it is recommended to limit the use of corticosteroids as prophylaxis (e.g., anti-emetics) to the first day of the chemotherapy, whenever possible. For urgent indications the use of corticosteroids will be at the Investigator’s or treating physician’s discretion.

9 TREATMENT DISCONTINUATION, STUDY WITHDRAWAL AND STUDY TERMINATION

9.1 Treatment discontinuation criteria

A patient will be discontinued from study treatment for any of the following reasons:

- Progressive disease (PD) (see Section 10.12 Assessment of anti-tumor activity)
- Clear clinical deterioration where continued study treatment would not be in the best interest of the patient, as judged by the Investigator
- Unacceptable toxicity as judged by the Investigator or the patient
- The patient's general condition or an unrelated AE that contraindicates continued study treatment, as judged by the Investigator
- Permanent discontinuation of mitazalimab treatment (for any reason)
- The patient has to receive or receives contraindicated medication (according to Section 8.9.7.1 Contraindicated medication and medications with interactions)
- Delay in dosing for more than 4 weeks due to mitazalimab related toxicity
- Pregnancy

9.2 Treatment discontinuation

Patients discontinued from study treatment should complete the End of treatment visit, preferably 28 days after the last dose of study treatment, where this is feasible. At this visit, information should be obtained about the reason(s) for discontinuation, AEs should be recorded and, as far as possible, a complete set of data at the point of discontinuation should be collected.

Patients discontinued from study treatment and recommended to receive a new cancer-related treatment or to continue to receive chemotherapy backbone alone, should complete the End of Treatment visit before the new cancer-related treatment starts.

Patients that have discontinued study treatment should enter the post-treatment follow-up period after the End of treatment visit has been performed unless any of the study withdrawal criteria in Section 9.3 below applies. Assessments to be performed during post-treatment follow-up, including disease/survival status and subsequent cancer-related therapy, are detailed in Table 4 for mitazalimab in combination with mFOLFIRINOX, and in Table 22 for mitazalimab in combination with nab-paclitaxel plus gemcitabine.

The post-treatment follow-up period will continue until the patient has died, is lost to follow-up or withdraws consent, or until any of the other study withdrawal criteria apply (see Section 9.3) for up to a maximum of 2 years after the LPI or until disease progression or clinical deterioration if study treatment continues past the 2 years after LPI. Patients who discontinue study treatment prior to the end of the DLT evaluation period (as defined in Section 5.3.3 Dose-limiting toxicity (DLT)

evaluation period) will be replaced unless they have experienced a DLT. If a patient is replaced, both the patient and the replacement patient are counted in the total number of study patients.
The primary reason for treatment discontinuation will be documented in the eCRF. If a patient wishes to discontinue due to an AE (or AEs), the reason for discontinuation will be Unacceptable toxicity or Unrelated AE, depending on the causality of the AE(s).

9.3 Study withdrawal criteria

A patient will be withdrawn from the study for any of the following reasons:

- Death
- The patient is lost to follow-up
- Withdrawal of consent. The patients can at any time discontinue their participation in the study
- Non-compliance with this Clinical Study Protocol as judged by the Investigator and/or Sponsor
- The study is terminated by the Sponsor (according to Section 9.5 Termination of the Study)

9.4 Study withdrawal

If a patient is withdrawn from the study during study treatment, the treatment with mitazalimab and chemotherapy will be discontinued. The patient should complete the End of treatment visit preferably 28 days after the last dose of study treatment, where this is feasible, (see Section 9.2 above), and End of study visit assessments.
The assessments at the End of treatment visit, should always be performed when a patient is withdrawn from the study unless they have already been performed, i.e., the patient is withdrawn during post-treatment follow-up.
Patients who are withdrawn from the study and recommended to receive a new cancer-related treatment or to continue to receive chemotherapy backbone alone, should complete the End of Treatment and End of study visit assessments before the new cancer-related treatment starts, if feasible.
For patients lost to follow-up (i.e., patients that fail to appear for study visits without stating an intention to withdraw consent), efforts must be made by the study site personnel to contact the patient and determine the reason for discontinuation/withdrawal. The measures taken to follow up should be documented. Every effort will be made to undertake protocol-mandated follow-up procedures.
Any patient withdrawn from the study will be given appropriate care under medical supervision until the symptoms of any AE resolve or become stable.

In all cases of withdrawal, the Sponsor should be informed as soon as possible.

9.4.1 Treatment plan for patients who have shown clinical benefit

Patients may continue study treatment for 12 treatment cycles of mitazalimab in combination with mFOLFIRINOX or 6 treatment cycles of mitazalimab in combination with gemcitabine plus nab-paclitaxel. Upon completion of 12 or 6 treatment cycles, the Investigator will review and assess each patient´s health and determine the best treatment options available. If the Investigator deems that a patient has shown clinical benefit (without confirmed disease progression) from the combination treatment with mitazalimab and mFOLFIRINOX or gemcitabine plus nab-paclitaxel and recommends the patient to continue with study treatment, the patient may continue until disease progression, unacceptable toxicity or until any other treatment discontinuation or study withdrawal criteria are met (see Section 9.1 and Section 9.3), whichever comes first.

9.5 Premature Termination of study

The Sponsor reserves the right to terminate the study at any time for any reason at the sole discretion of the Sponsor but intends only to exercise this right for valid scientific or administrative reasons. The Investigator or Sponsor may initiate study site closure at any time, provided there is a reasonable cause and sufficient notice is given in advance. Conditions that may warrant termination of the study at an individual site or overall, include, but are not limited to:

- Finding that the lowest planned dose of mitazalimab is not safe and tolerable in combination with at least one of the planned chemotherapy regimens (mFOLFIRINOX or gemcitabine plus nab-paclitaxel) in Part 1 of this study
- DRC recommendation to not proceed to Part 2 after conclusion of Part 1 of the study
- The discovery of an unexpected, significant, or unacceptable risk to the patients enrolled in the study
- Failure of the Investigators to enter patients at an acceptable rate
- Insufficient adherence to protocol requirements (non-compliance)
- Lack of evaluable and/or complete data
- Decision to modify the development plan of the drug, and/or
- A decision on the part of the Sponsor to suspend or discontinue development of the drug

In case patients should stop study treatment, the Investigator must call in the relevant patients and inform them of the decision as well as to perform relevant study assessments.

9.6 End of Trial

The End of Trial (completion) is defined as the last study assessment for the last patient on study.

Additionally, approximately two years after last patient in or, once a sufficient number of overall survival events for the study population have been recorded (whichever comes first), the Sponsor may decide to declare the study as completed.

10 STUDY ASSESSMENTS

Please refer to Section 1 VISIT ASSESSMENT SCHEDULE, for detailed timings of the different assessments mentioned below for mitazalimab administered in combination with mFOLFIRINOX and to APPENDIX 3 for mitazalimab administered in combination with gemcitabine plus nab-paclitaxel.

When unscheduled visits/assessments are performed, the reason for the visit/assessment should be recorded in the eCRF.

10.1 Demographics

Age, gender, race and ethnic origin of study participants will be recorded.

10.2 Medical history

Relevant past medical conditions, as judged by the Investigator, and all concurrent medical conditions will be recorded by date and diagnosis on the Medical History eCRF page.

Clinically significant abnormal findings observed during the physical examination and non-serious AEs occurring pre-treatment (i.e., after signing the ICF but before first dose) should be recorded as medical history. See also Section 11.2.1 Time period for collection of AEs.

10.2.1 Cancer disease status

The date of initial histopathological diagnosis of pancreatic adenocarcinoma, will be recorded as well as the disease stage at time of diagnosis and the current disease stage.

Surgery for the cancer should be recorded, including information if the surgery was of curative intention.

Radiotherapy should be recorded, also when given for palliative purpose.

10.2.2 Other cancer than study disease

Any prior anti-cancer treatment for cancer disease other than pancreatic adenocarcinoma (if applicable) should be recorded in the eCRF, including:

- Treatment identity, including surgery, radiotherapy and chemotherapy as applicable
- Start and end date of treatment
- Best response (CR, PR, SD, PD and NE)
- Reason for discontinuation (for treatments stopped before initially planned)

10.3 Body weight and height

Body weight (without overcoat and shoes) will be measured at screening and during treatment and recorded on the eCRF page rounded to the nearest kilogram.

The dose calculation of mitazalimab is based on the body weight. In case the body weight changes more than 10% from baseline measurement, the mitazalimab dose needs to be re-calculated, see Section 8.3.

The dose calculation for constituents of mFOLFIRINOX, gemcitabine and nab-paclitaxel is based on BSA. In case the BSA changes more than 10% based on calculation using baseline measurement of body weight, the dose of constituents of mFOLFIRNOX, gemcitabine and nab-paclitaxel needs to be re-calculated.

Additional body weight assessments may be performed, and recorded in the eCRF, based on Investigator's judgement at regular visits or at additional (Unscheduled) visits.

Height (without shoes) will be measured at screening and recorded on the eCRF page rounded to the nearest centimeter.

10.4 Vital signs

The vital sign measurements include systolic and diastolic blood pressure, pulse rate, oxygen saturation and body temperature.

Blood pressure determinations should, preferably using the same equipment within each visit, be made after the patient has rested for 10 minutes.

Clinically significant abnormal findings collected pre-treatment (i.e., after the patient signed the ICF but before first dose) should be reported on the Medical History eCRF page.

Any new or aggravated clinically significant abnormal findings as compared with the pre-treatment assessment will be reported as an AE (see Section 11.2 ADVERSE EVENT REPORTING).

Additional vital sign assessments may be performed, and recorded in the eCRF, based on Investigator's judgement at regular visits or at additional (Unscheduled) visits.

10.5 Physical examination

Physical examination will as a minimum include general appearance and examination of the following body systems: eyes, mouth and throat, lymph node regions, respiratory, cardiovascular system, abdomen, extremities, and skin. An examination of the neurological system may be done based on the investigator's judgement.

Clinically significant abnormal findings collected pre-treatment (i.e., after the patient signed the ICF but before first dose) should be reported on the Medical History eCRF page.
Any new or aggravated clinically significant abnormal medical findings as compared with the pre-treatment assessment will be reported as an AE, see Section 11 ADVERSE EVENT REPORTING.
Additional assessments of physical examination may be performed, and recorded in the eCRF, based on Investigator's judgement at regular visits or at additional (Unscheduled) visits.

10.6 Electrocardiogram (ECG)

Standard 12-lead ECGs will be recorded, and an overall interpretation of the ECGs will be performed by the Investigator or, if applicable, the Investigator may delegate this task to a cardiologist. The ECG printout must be signed and dated following review and interpretation.
For the ECG recordings, the patients must be resting and in horizontal or half laid position for at least 10 minutes. The same method of assessment should be used throughout the study. Any irregularity observed or occurring during the ECGs (e.g., vomiting, cough) should either induce a repeat of the ECG or be annotated on the eCRF with the description and time of the occurrence.
For evaluation of exclusion criteria number 7, the QTc is assessed using Bazett's formula [35].
Clinically significant abnormal ECG findings collected pre-treatment (i.e., after the patient signed the ICF but before first dose) should be reported on the Medical History eCRF page.
Any new or aggravated clinically significant abnormal ECG findings as compared with the pre-treatment assessment will be reported as an AE, see Section 11 ADVERSE EVENT REPORTING.
Additional ECGs recordings may be performed, and recorded in the eCRF, based on Investigator's judgement at regular visits or at additional (Unscheduled) visits.

10.7 ECOG performance status

The Eastern Cooperative Oncology Group (ECOG) performance status scale [36] provided in Table 10 below, will be used by the Investigator to grade the patients' performance status of daily living activities. The result of the ECOG assessment should be recorded in the eCRF.

Table 10 ECOG performance status

Grade	ECOG
0	Fully active, able to carry on all pre-disease performance without restriction.
1	Restricted in physically strenuous activity but ambulatory and able to carry out work of a light or sedentary nature, e.g., light housework, office work.
2	Ambulatory and capable of all self-care but unable to carry out any work activities. Up and about more than 50% of waking hours.
3	Capable of only limited self-care, confined to bed or chair more than 50% of waking hours.
4	Completely disabled. Cannot carry on any self-care. Totally confined to bed or chair.
5	Dead

10.8 Concomitant medical procedures

Concomitant medical and surgical procedures should be discussed in advance with the study Medical Monitor with the intention of identifying any issues that could affect conduct or interpretation of the study. In the event that a procedure needs to be performed urgently, it should be discussed as soon afterwards as is practical. This discussion, the procedure and indications for the procedure will be documented.

10.9 Clinical laboratory tests

The time points for the clinical laboratory assessments are specified in Section 1 VISIT ASSESSMENT SCHEDULE. However, more frequent tests may be performed if indicated by the clinical condition of the patient or by abnormalities that warrant more frequent monitoring as judged by the Investigator.

The Investigator must review the laboratory results, document this review and enter the laboratory results in the relevant eCRF pages. The screening laboratory results must be available and reviewed by the Investigator before the first dose of mitazalimab.

The results from the clinical laboratory tests will be summarized in the Clinical Study Report. Therefore, all clinically significant abnormal laboratory results (as determined by the investigator), even if performed outside the protocol schedule, must be reported in the relevant Clinical Chemistry, Hematology or Coagulation eCRF pages. Deterioration as compared to pre-treatment in a laboratory test must be reported as an AE if it meets the criteria for a SAE/AESI or is the reason for modification of any study treatment (e.g., dose delay, dose reduction). Other laboratory abnormalities that the investigator determines should be reported as an AE may also be reported. Deterioration of a laboratory value that is unequivocally due to disease progression will not be reported as an AE.

The procedures for blood and urine sample collection, preparation and handling will be performed as per local procedures. The DPD test taken at screening will be analysed at a central laboratory or per local procedures.
The clinical laboratory tests to be performed are listed in Table 11.

All tests should be performed by the local laboratory, except for the urinalysis tests which will be performed and assessed by the site personnel.
If an abnormal urinalysis result (urine dipstick) is regarded as clinically significant, microscopy will be used *to measure sediment, i.e., red blood cells, white blood cells, epithelial cells, crystals, casts, and culture of bacteria.*
Table 11 Clinical laboratory tests

**Dihydropyrimidine dehydrogenase (DPD)**	DPD assessed during screening only when mitazalimab is given in<br>combination with mFOLFIRINOX. Analysed at central laboratory or per local procedures.	
**Pregnancy testing** (for women of childbearing potential only)	Serum β-human chorionic gonadotropin (β-hCG) pregnancy test is required at screening, at the other time points either serum or urine pregnancy tests can be used.<br>The results of the pregnancy test must be available prior to each drug administration.	
**Clinical chemistry**	• Creatinine<br>• Total bilirubin<br>• Alanine aminotransferase (ALT)<br>• Aspartate aminotransferase (AST)<br>• Alkaline phosphatase<br>• Albumin<br>• Total protein<br>• Total calcium<br>• Glucose<br>• Creatine kinase<br>• C-reactive protein	• Lactate dehydrogenase (LDH)<br>• Urea or Blood urea nitrogen (BUN)<br>• Uric acid<br>• Phosphorus<br>• Bicarbonate<br>• Sodium<br>• Potassium<br>• Magnesium<br>• Chloride (if feasible)
**Hematology**	• White blood cell count with differential (absolute) counts (neutrophils, eosinophils, lymphocytes, and monocytes, basophils)	• Hemoglobin<br>• Platelet count
**Coagulation**	• International normalized ratio (INR)<br>• aPTT	• D-dimer<br>• Fibrinogen
**Urinalysis** (dipstick)	• Protein<br>• Hemoglobin/blood<br>• Glucose<br>• Specific gravity<br>• pH	• Ketones<br>• Bilirubin<br>• Urobilinogen<br>• Nitrite<br>• Leukocyte esterase

10.10 Immunogenicity (anti-drug antibodies)

Blood samples (serum) will be taken for immunogenicity testing according to Section 1 VISIT ASSESSMENT SCHEDULE for mitazalimab in combination with mFOLFIRINOX and according to visit assessment tables in APPENDIX 3 for mitazalimab in combination with gemcitabine and nab-paclitaxel. Samples may also be collected at additional time points, at regular visits or at additional (Unscheduled) visits, based on Investigator's judgement.

If the infusion of mitazalimab is interrupted due to an AE, a sample for immunogenicity should be collected (except during the first infusion) at the time of interruption or as soon as feasible considering patient safety together with a PK sample.

The samples for immunogenicity testing will be used for anti-drug antibody (ADA) analysis (i.e., antibodies to mitazalimab). The samples analysed for immunogenicity and confirmed positive will be tested for neutralizing antibodies. Other analyses may be performed to further characterize the immunogenicity of mitazalimab. Immune response analysis may be conducted on PK samples collected at other timepoints noted in the visit assessment schedule if deemed necessary.

Details regarding sample collection and processing will be provided in the Laboratory Manual.

10.11 Pharmacokinetics (PK)

Blood samples (serum) will be taken for analysis of mitazalimab concentrations and PK analysis according to Section 1 VISIT ASSESSMENT SCHEDULE for mitazalimab in combination with mFOLFIRINOX and APPENDIX 3 for mitazalimab in combination with gemcitabine and nab-paclitaxel. PK samples may be collected at additional time points, at regular visits or at additional (Unscheduled) visits, based on Investigator's judgement.

The samples for PK analysis must be taken from a peripheral vein contralateral to the arm into which mitazalimab is infused.

If the infusion of mitazalimab is interrupted due to an AE, a PK sample and a sample for immunogenicity should be collected (except during the first infusion when no sample for immunogenicity needs to be taken) at the time of interruption or as soon as feasible considering patient safety.

Date and time of PK samples should always be collected in the eCRF.

Details regarding sample collection and processing will be provided in the Laboratory Manual. The following PK parameters will be derived for mitazalimab:

- Cmax
- Tmax
- AUC(0-T)

Other PK parameters may be derived if data allows such as:

- AUC0-∞
- AUCτ
- Elimination half-life (T1/2)
- Total serum clearance (CL)
- Volume of distribution (Vd)

10.12 Assessment of anti-tumor activity

10.12.1 Computed tomography (CT) scan

A CT scan of chest/abdomen/pelvis should be taken according to local practice. Other body areas may also be CT scanned if needed to assess the tumor(s) (e.g., a CT scan of neck would be needed for a patient having cervical nodes or a head and neck tumor). Additional CT scans may be taken based on Investigator's judgement at regular visits or at additional (Unscheduled) visits.

The use of intravenous contrast is at the discretion of the radiologist performing the scanning, but imaging must be consistent per patient throughout the study.

If a CT scan is considered not feasible, as judged by the Investigator, a Magnetic Resonance Imaging (MRI) may be performed. The same scanning modality must be used throughout the study.

The Investigator and/or radiologist will identify the tumors to be followed throughout the study. These will be recorded on the relevant eCRF page(s).

A CT scan is performed at screening (within 28 days of first dose), 9 weeks after start of study treatment (typically end of cycle 4 for mFOLFIRINOX combination and at end of Cycle 2 for gemcitabine plus nab-paclitaxel treatment) and thereafter every 8th week irrespective of any treatment delays. Additional CT scans may be performed at the discretion of the Investigator at unscheduled visits.

The CT scans will be evaluated according to RECIST v. 1.1 according to Section 10.12.2 Tumor response evaluation. Patients with response (PR or CR) should have a confirmatory CT scan at least 4 weeks later to confirm the response. If the patients have progressive disease, the patients should discontinue treatment).

10.12.2 Tumor response evaluation

The evaluation of tumor response will be done according to RECIST v. 1.1 by the investigational sites using CT scans according to APPENDIX 1 RECIST v. 1.1 guideline.

The tumor responses will be recorded on the Cycle Response Assessment eCRF pages.

Patients with PD should discontinue study treatment (see Section 9.1). However, patients with suspected progression are allowed to continue on treatment if they are considered to be clinically stable in the opinion of the Investigator until clinical or radiological progression is documented.

10.13 Pharmacodynamics

The aim with the pharmacodynamic biomarkers is to characterize changes in intratumoral and systemic immune activation associated with mode of action of mitazalimab and correlate pharmacodynamic effects with clinical response. Disease progression and treatment response will also be followed with liquid biopsies, analyzing biomarkers such as Carbohydrate antigen 19-9 (CA19-9) and circulating tumor DNA (ctDNA).
All biomarker analyses in blood and tumor biopsies are exploratory in nature. All exploratory analyses will be performed at a fit-for-purpose laboratory, as the data from the analyses are for scientific use and decision making only. The data may be included in a scientific publication but will not be included in the clinical study report. However, in case where an analytical method is fully validated and performed at a GCP compliant laboratory prior to sample testing, results may be included in the clinical study database and reported in the Clinical Study Report.

10.13.1 Blood

The blood samples will be taken according to Section 1 Visit Assessment Schedule, for mitazalimab in combination with mFOLFIRINOX, and according to APPENDIX 3 for mitazalimab in combination with gemcitabine plus nab-paclitaxel.
Date and time of the samples should be collected in the eCRF.

Details regarding sample collection and processing will be provided in the Laboratory Manual.

Different types of blood samples will be taken and the following pharmacodynamic biomarkers may be evaluated:
Cytokines and chemokines: Serum samples are to be analyzed for levels of cytokines and chemokines involved in the immune activation of mitazalimab, including but not limited to IFN-γ, TNF-α, IL12p70, IL-6, MCP-1, IP-10, MIP-1α, MIP-1β and IL-8; e.g., using a 30-plex kit with Luminex, MSD or similar.
Immune phenotyping: Whole blood samples will be used for immunophenotyping of whole blood for quantification of immune cell populations and immune cell activation directly or indirectly involved with immune activation of mitazalimab using flow cytometry. The following biomarker panels may be analyzed:

- T/NK/NKT cells (e.g., CD45, CD3, CD8, CD4, CD16, CD56, CCR7, CD45RA + activation markers CD25, Ki67)
- B cells (e.g., CD45, CD19, CD27, IgD + activation markers CD86, CD83, CD54, HL-DR)

- Monocytes and DCs (CD45, CD14, CD16, CD11c, CD123 + activation markers CD54, CD86, HLA-DR, CD83)

Whole blood RNA: Whole blood RNA samples will be collected for analyzation of gene signatures involved in the immune activation with mitazalimab (including but not limited to: immunoscore, IFNg genes profile, APC gene profile and T cell gene profile) and prognostic tumor gene profiles (e.g., TMB and MSI-high) and can be analyzed with e.g., Nanostring IO 360, RNAseq. or similar.
Whole blood DNA: Cell free whole blood DNA samples will be collected for evaluation of circulating tumor DNA (ctDNA). Changes in tumor specific genes e.g., KRAS will be followed as a biomarker to monitor treatment response.
Whole blood DNA can be collected for evaluation of TCRb clonality.

CA19-9: Changes in the levels of pancreatic serum protein CA19-9 will be followed as a biomarker for monitoring disease progression and treatment response. Locally measured CA19-9 will also be collected in the eCRF, if available.

10.13.2 Tumor biopsy

Tumor biopsies will be collected according to Section 1 Visit Assessment Schedule, for mitazalimab in combination with mFOLFIRINOX, and according to APPENDIX 3 for mitazalimab in combination with gemcitabine plus nab-paclitaxel.
Collection of biopsies may be omitted in case the tumor is inaccessible or the biopsy procedure expose the patient to an increased risk, as judged by the Investigator. If a biopsy cannot be taken at screening, archival biopsy material (i.e., tissue slides or tissue block, preferably from the most recently collected tumor biopsy prior to enrollment) should be collected if possible. If no biopsy is obtained (fresh nor archival) at screening, no further biopsies will be collected during the study. Date of the biopsy collection should be documented in the eCRF.

Biopsies (core biopsy, 18-gauge needle) will be taken under local anesthesia. The tumor biopsies should preferably be taken from the same tumor throughout the study. Imaging can be used to guide the biopsies. Tumor tissue collected by punch biopsy or excisional biopsy are acceptable alternatives for cutaneous tumors.
Anticoagulation medication must be paused as medically indicated prior to a tumor biopsy is performed.
The biopsies will be formalin-fixed and paraffin-embedded. The procedures for biopsy collection, preparation and handling will be provided in the Laboratory Manual.
Tumor biopsy analysis may include:

Immunohistochemistry: Archival or freshly collected tumor biopsies may be analyzed for CD40 target expression and immune cell infiltration at baseline and correlate with clinical response. On treatment biopsies will be compared with baseline biopsies for analyzation of immune cell infiltration and immune activation induced by mitazalimab for proof of mechanism. The following immunohistochemistry (IHC) panels may be included:

- CD40 target expression
- T cell infiltration and activation status
- Macrophage infiltration with M1/M2 differentiation and activation status
- Cell surface markers related to immune regulation e.g., PD-L1
- Other markers describing proof of mechanism such as e.g., fibrotic markers and stroma (e.g., fibronectin and collagen type I) macrophage functionality related to macrophage functionality

Gene profiling: Baseline and on treatment tumor biopsies will be collected for analyzation of gene signatures involved in the immune activation with mitazalimab (including but not limited to: immunoscore, IFNg genes profile, APC gene profile and T cell gene profile) and prognostic tumor gene profiles (e.g., TMB and MSI-high) and can be analyzed with e.g., Nanostring IO 360, RNAseq. or similar.

Any tumor biopsy material remaining after the analysis described, will be stored in a biobank, for possible future analyses, see Section 14.5.

10.14 Disease/survival status (post treatment follow-up)

During the post-treatment follow-up, disease and survival status will be collected, see Table 4 in Section 1 VISIT ASSESSMENT SCHEDULE for mitazalimab in combination with mFOLFIRINOX and Table 22 in APPENDIX 3 for mitazalimab in combination with gemcitabine plus nab-paclitaxel. The following will be assessed:

- Survival
- Current disease stage

If the patient has died, the date and cause of death will be collected and documented.

Disease/survival status may be followed up via the patient's medical records (as allowed by local regulations) or phone contact. Where allowed by local law, public records may be used to document death for the purpose of obtaining survival status.

Written documentation must be available for review in the source documents.

10.15 Subsequent cancer-related therapy/procedure (post treatment follow-up)

During the post-treatment follow-up, all subsequent anti-cancer therapies/procedures will be collected.

Data on subsequent anti-cancer therapies/procedures may be recorded via the patient’s medical records (as allowed by local regulations) or phone contact. Written documentation must be available for review in the source documents.

11 ADVERSE EVENT REPORTING

11.1 Definitions

11.1.1 Adverse event (AE)

An AE is any untoward medical occurrence in a patient or clinical investigation subject administered a medicinal product and which does not necessarily have a causal relationship with this treatment. An AE can be any unfavorable and unintended sign (including an abnormal laboratory finding), symptom or disease temporally associated with the use of a medicinal product, whether or not considered related to the medicinal product.

11.1.2 Adverse reaction (AR)

All untoward and unintended responses to an investigational medicinal product related to any dose administered.

The phrase "responses to a medicinal product" means that a causal relationship between a medicinal product and an AE is at least a reasonable possibility, i.e., the relationship cannot be ruled out.

11.1.3 Serious adverse event (SAE) or serious adverse reaction (SAR)

A serious AE (SAE) or serious AR (SAR) is any untoward medical occurrence or effect that at any dose:

- Results in death
- Is life-threatening (*Note*: The term "life-threatening" in the definition of "serious" refers to an event in which the patient was at risk of death at the time of the event; it does not refer to an event which hypothetically might have caused death if it were more severe)
- Requires in-patient hospitalization or prolongation of existing hospitalization, unless the hospitalization is for:
    - Routine treatment or monitoring of the disease under study, including hospitalization due to study-related procedures (e.g., administration of medicinal product) or to manage AEs related to signs or symptoms of disease under study
    - Elective treatment (planned before signing the ICF) for a pre-existing condition that is unrelated to the disease under study and has not worsened since signing the ICF
    - Treatment on an emergency outpatient basis for an event not fulfilling any of the definitions for an SAE
    - Social reasons, respite care in the absence of a medical condition
- Results in persistent or significant disability/incapacity or substantial disruption of the ability to conduct normal life functions
- Is or results in a congenital abnormality or birth defect

Medical and scientific judgement should be exercised in deciding whether expedited reporting is appropriate in other situations, such as important medical events that may not be immediately life-threatening or result in death or hospitalization but may jeopardize the patient or may require intervention to prevent one of the other outcomes listed in the definition above. These AEs should also usually be considered serious. Examples of such events are intensive treatment in an emergency room or at home for allergic bronchospasm; blood dyscrasias or convulsions that do not result in hospitalization; or development of drug dependency or drug abuse.

To ensure no confusion or misunderstanding of the difference between the terms “serious” and “severe,” which are not synonymous, the following note of clarification is provided:

The term “severe” is often used to describe the intensity (severity) of a specific event (as in mild, moderate, or severe myocardial infarction); the event itself, however, may be of relatively minor medical significance (such as severe headache). This is not the same as “serious,” which is based on patient/event outcome or action criteria usually associated with events that pose a threat to a patient’s life or functioning. Seriousness (not severity) serves as a guide for defining regulatory reporting obligations.

11.1.4 Adverse events of special interest (AESIs)

An adverse event of special interest (AESI) is any AE, serious or non-serious, that is of scientific and medical concern to the Sponsor’s product or program, for which ongoing monitoring and rapid communication by the Investigator to the Sponsor can be propagated.

The following AEs are considered as AESIs for this Clinical Study Protocol:

- Infusion-related reaction grade 2 or higher (see Section 8.6 Handling of infusion-Related Reactions)
- Cytokine release syndrome grade 2 or higher (see Section 8.6 Handling of infusion-Related Reactions)
- Liver enzyme (AST and/or ALT) elevation of grade 3 or higher
- Bilirubin elevation of grade 2 or higher

11.1.5 Reference Safety Information (RSI)

The Reference Safety Information (RSI) is a list of expected SARs which are classified using preferred terms (PTs) according to the Medical Dictionary for Regulatory Activities (MedDRA). Please refer to the current approved version of the mitazalimab Investigator’s Brochure for the RSI.

11.1.6 Unexpected adverse reaction

An unexpected AR is an AR where the nature or severity of which is not consistent with the applicable product information (e.g., the RSI in the Investigator's Brochure for an unapproved investigational medicinal product).

11.1.7 Suspected Unexpected Serious Adverse Reaction (SUSAR)

A Suspected Unexpected Serious Adverse Reaction (SUSAR) is an adverse reaction which is both serious and unexpected according to the applicable Reference Safety Information (RSI) defined above.

11.1.8 Disease progression

Disease progression can be considered as a worsening of a patient's condition attributable to the disease for which the investigational product is being studied. It may be an increase in the severity of the disease under study and/or increases in the symptoms of the disease.
Deterioration of the disease under study and associated symptoms or findings, including the development of new, or the progression of existing, metastases, should not be regarded as an AE, unless the study medication is considered to have contributed to the progression.

11.1.9 New cancers

New cancers are those that are not the primary reason for the administration of the study treatment and have been identified after the patient's inclusion in this study. They do not include metastases of the original cancer. The development of a new cancer should be regarded as an AE and will generally meet at least one of the serious criteria.

11.1.10 Medication error and overdose

A medication error is an unintended failure in the drug treatment process that leads to, or has the potential to lead to, harm to the patient.
Overdose refers to the administration of a quantity of a medicinal product given per administration or cumulatively, which is more than the assigned dose for a given patient (i.e., the intended dose).
For details regarding reporting of medication error/overdose, see Section 11.2.8 Reporting medication error and overdose.

11.2 Adverse event (AE) reporting procedures

11.2.1 Time period for collection of AEs

AEs will be collected throughout the study, from signing the ICF until 28 days after the last administration of study treatment (i.e., either mitazalimab or chemotherapy), irrespective of

relationship to study treatment. AEs that occur more than 28 days after the last administration of study treatment will only be collected if the Investigator assesses them as related to study treatment. Untoward medical occurrences that occur after the patient signed the ICF but before the first administration of study treatment will be collected as Medical History. However, if such an untoward medical occurrence is assessed as having been caused by a protocol-mandated intervention (e.g., invasive procedures such as biopsies) and meets the criteria for being serious, it will be reported to the Sponsor's representative (Theradex) as if it were an SAE and reported on the AE eCRF page. All untoward medical occurrences that occur after first administration of study treatment will be reported on the AE eCRF page.

Pre-existing conditions will not be reported as AEs. However, if a pre-existing condition worsens during the treatment period it will be reported as an AE.

11.2.2 Follow-up of AEs

All AEs will be followed until they are resolved or until 28 days after the last administration of study treatment, whichever comes first. All AEs that are still ongoing 28 days after the last administration of study treatment, will be followed on a regular basis, according to the Investigator's clinical judgment, until the event has resolved or until the Investigator assesses it as chronic, or until the patient starts a new treatment regimen or the patient is lost to follow-up.

AEs ongoing 28 days after the last administration of study treatment, do not require further recording in the eCRF unless they are serious. When a SAE resolves, the resolved date and outcome (resolved or resolved with sequelae, as appropriate) should be recorded on the eCRF and the SAE/AESI Report Form. If a SAE does not resolve, the Investigator will confirm one of the following outcomes: assessed as chronic, started a new cancer treatment, patient is lost to follow-up. The Sponsor retains the right to request additional information for any patient with ongoing AE(s) at the end of the study, if judged necessary.

11.2.3 AE variables

The following variables will be collected for each AE:

11.2.3.1 AE term

All AEs spontaneously reported by the patient or reported in response to open-ended and non-leading verbal questioning from the study personnel (e.g., "How are you feeling?" "Have you had any health problems since the previous visit/you were last asked?"), or revealed by observation will be collected and recorded in the eCRF.

Where possible a diagnosis will be recorded, rather than recording a list of signs and symptoms. For example, if a patient has cough, fever and pleuritic pain and these are medically confirmed as symptoms of a respiratory infection, the reported AE term will be respiratory infection. Cough,

fever and pleuritic pain will not be recorded as separate AEs. However, if a diagnosis cannot be made, each sign or symptom will be recorded as an AE. Signs or symptoms that are not generally part of a diagnosis will be recorded as AEs.
The results from clinical laboratory tests will be summarized in the Clinical Study Report. Deterioration as compared to pre-treatment in these parameters will therefore only be reported as AEs if they fulfill any of the criteria for a SAE or are the reason for modifying the study treatment.
Deterioration of a laboratory value that is unequivocally due to disease progression will not be reported as an AE.
Clinically significant deteriorations in non-mandated parameters will also be reported as AE(s).

Wherever possible the reporting Investigator will use the clinical, rather than the laboratory term (e.g., anemia rather than low hemoglobin, neutropenia rather than low neutrophil count).
If deterioration in a laboratory value, vital sign, ECG, or other safety assessment is associated with a clinical event (e.g., hypokalemia due to diarrhea or vomiting), the clinical event will be reported as the AE.
Any new or aggravated clinically significant abnormal medical finding at a physical examination as compared with the pre-treatment assessment will be reported as an AE.
Events that are unequivocally due to disease progression should not be reported as AEs during the study.

11.2.3.2 Start/stop date and start/stop time

The date the AE started and the date it stopped, if it resolved completely, will be recorded. If a diagnosis has been made, the start date of the first symptom will be recorded. Complete dates (i.e., day, month and year) are required for all AEs. If an AE changes CTCAE grade, a stop date should be recorded and the AE is started again with the new CTCAE grade.
For AEs of less than 24 hours duration or that start on the same day as study treatment (e.g., allergic reaction), the time the AE started and ended will also be recorded.
If the AE is fatal, the date of death will be recorded as the stop date for the AE. All AEs that were ongoing at the time of death but which did not contribute to the death will be recorded as Not recovered/Not resolved or Recovering/Resolving, as appropriate.

11.2.3.3 CTCAE grade

The grading scales found in the National Cancer Institute Common Terminology Criteria for Adverse Events (CTCAE) version 5.0 will be used to assign the severity for all events. For AEs without assigned CTCAE grades, the general guideline in the CTCAE criteria that describes mild, moderate,

and severe events into CTCAE grades will be used. A copy of the current CTCAE version can be downloaded from the Cancer Therapy Evaluation Program website4.

11.2.3.4 Relationship to study treatment

The Investigator will assess whether or not the AE is related to study treatment. The question, "Do you consider that there is a reasonable possibility that the event may have been caused by the study treatment?" will be answered "yes" or "no".

For SAEs, the causal relationship to other medication and study procedure(s) will also be assessed.

A guide to the interpretation of the causality question is found in APPENDIX 8 Guidance on assessing causal relationship.

11.2.3.5 Action taken with study treatment

The action taken with the study treatment because of the AE will be reported as:

- None
- Infusion interrupted
- Dose delayed
- Dose reduced
- Dose missed
- Dosing permanently stopped
- Not applicable
- Unknown

Not applicable should only be used if the AE occurs before first treatment (e.g., SAE due to protocol-mandated procedure, see Section 11.2.1 Time period for collection of AEs).

11.2.3.6 Outcome

The Outcome of each AE will be assessed as one of the following:

- Recovered/Resolved: The patient has fully recovered (does not mean the condition has returned to pre-treatment grade).
- Recovered/resolved with sequelae: The patient has recovered from the condition, but with lasting effect due to disease, injury, treatment or procedure.
- Recovering/Resolving: The condition is improving and the patient is expected to recover from the event.
- Not recovered/Not resolved: The condition of the patient has not improved and the symptoms are unchanged, or the outcome is not known.

4https://ctep.cancer.gov/protocoldevelopment/electronic_applications/docs/CTCAE_v5_Quick_Reference_8.5x11.pdf

- Fatal: Only applicable if the patient died from the reported AE. An AE with fatal outcome must be reported as an SAE.

Unknown: Only applicable if the patient is lost to follow-up.

11.2.4 Reporting death

All deaths that occur during the study, or within the follow-up period after the administration of the last dose of study treatment, will be reported as follows:

If the death is unequivocally due to progression of the disease under study, this will be communicated to the CRA at the next monitoring visit and will be documented in the eCRF module but will not be reported as an AE or SAE during the study.

If death is not unequivocally due to progression of the disease under study, the AE that caused death will be reported to the Theradex Pharmacovigilance (PV) Group of the Sponsor's representative (Theradex) as an SAE within 24 hours. The report will assign a single primary cause of death together with any contributory causes (including progressive disease, if appropriate).

If the cause of death is unknown this will be reported as a SAE, but every effort will be made to establish a cause of death. A post-mortem may be helpful in the assessment of the cause of death, and if performed a copy of the post-mortem results (with translation of important parts into English) will be reported in an expedited fashion to the Theradex PV Group within 24 hours.

11.2.5 Reporting SAEs and AESIs

All SAEs/AESIs that occur during the timeframe specified in Section 11.2.1 Time period for collection of AEs, must be reported to the Theradex PV Group immediately, or no later than 24 hours of when site personnel became aware of it using the SAE/AESI Report Form. All SAEs/AESIs will also be recorded in the eCRF.

AEs related to sign or symptoms of disease under study that require hospitalization for management, should not be reported as SAEs unless other serious criteria are met.

If important information is missing (e.g., relationship to study treatment), the Theradex PV Group will follow-up with the site immediately.

Follow-up information on a previously reported SAE/AESI must also be reported to the Theradex PV Group immediately, or no later than 24 hours of when site personnel became aware of it.

The SAE/AESI Report Form must be completed and forwarded via fax or email (as a PDF attachment) to the Theradex CRO PV Group within 24 hours of awareness of the event. The fax and telephone numbers listed below may be used during both business and non-business hours. During non-business hours a recorded message will provide the caller with the contact information for the on-call Safety Physician.

SAEs will be reported to:	Theradex PV Group<br><br>Telephone: +44 (0) 1293 510 319<br><br>Fax: +44 (0) 1293 510 322<br><br>Email SafetyDeskEurope@theradex.co.uk

11.2.6 Reporting pregnancy

If a patient becomes pregnant during the course of the study, study treatment should be discontinued immediately. For details on patients being discontinued from study treatment see Section 9.2 Treatment discontinuation.

Pregnancy will not be reported as an AE unless there is a suspicion that the study treatment may have interfered with the effectiveness of a contraceptive medication. Congenital abnormalities/birth defects and spontaneous miscarriages will be reported and handled as SAEs. Elective abortions without complications should not be reported as AEs. The outcome of a pregnancy should be followed up and documented even if the patient was withdrawn from the study.

For any conception occurring during exposure to study treatment or within 6 months after discontinuing study treatment, Investigators or other site personnel must inform the Theradex PV Group immediately, or no later than 24 hours of when he or she becomes aware of it.

The same timelines apply when outcome information is available.

11.2.7 Reporting paternal exposure

Pregnancy of a patient's partner will not be reported as an AE. Congenital abnormalities/birth defects and spontaneous miscarriages in the partner of a male patient will be reported and handled as SAEs. Elective abortions without complications should not be reported as AEs.

Any conception in the partner of a male patient occurring from the first administration of study treatment until 6 months after the last administration of study treatment should be reported to the Theradex PV Group and followed up for its outcome.

11.2.8 Reporting medication error and overdose

Medication errors and overdose will be documented as protocol deviations and will not be reported as AEs. Adverse reactions (ARs) associated with medication errors/overdose should be reported as AEs and reported to the CRO Theradex as SAEs, if any of the serious criteria in Section 11.1.3 Serious adverse event (SAE) or serious adverse reaction (SAR), are met.

11.2.9 Reporting SUSARs

The Sponsor's representative Theradex will inform the Regulatory Authorities of all SUSARS in the study via the EudraVigilance Clinical Trial Module (EVCTM).
Life-threatening or fatal SUSARs will be submitted to Competent Authorities without delay (with submission of a follow-up report if needed within the subsequent 8 days). All other SUSARs will be reported to Competent Authorities no later than 15 days after the event (with submission of a follow-up report if needed within the subsequent 8 days).
The CRO Theradex will provide Investigators with a line listing of all SUSARs every 6 months. In addition, SUSARs that may warrant a change to this Clinical Study Protocol will be provided in real time (i.e., within 7 or 15 days, as appropriate).

11.2.10 Communication of Safety Information

Prompt communication of serious adverse events (SAEs) and suspected unexpected serious adverse reactions (SUSARs) or serious safety-related protocol deviations between the sponsor, all study sites and investigators and study participants is of high importance. The process for reporting of SAEs is described Section 11.2.5 and SUSARs in Section 11.2.9. SUSARs will be conveyed to site investigators by Dear Investigator Letters and through provision of 6-monthly line listings as detailed in the safety management plan. Serious safety-related protocol deviations that may impact all sites will be communicated to the investigators immediately.
In the event of an urgent safety measure, the CRO will inform the Regulatory Authorities, EC and Investigators. The study may be temporarily halted and/or a protocol amendment may be required. Study participants will be notified of important information concerning the trial via their respective sites and investigators.

11.3 Halting rules

Safety data that may temporarily suspend enrollment or study interventions until a safety review is convened includes:

- Overall number of SAEs
- Number of occurrences of a particular type of SAE
- Severe AEs/reactions

Safety reviews and oversight will be under the direction of the DRC, see Section 6 Data Review Committee.

12 STATISTICS

12.1 Study hypotheses

No formal statistical hypothesis is defined for Part 1 (Phase 1b).

For Part 2 (Phase 2), the null hypothesis for the primary endpoint ORR is: H0: ORR=30%
The null hypothesis will be tested against the alternative: H1: ORR>30%

12.2 Sample size considerations

In Part 1, it is estimated that at least 9 patients will be included in two dose levels with at least 3 patients on each dose and 6 patients at the RP2D.
For Part 2 the sample size calculation is based on an assumption of 30% ORR for mFOLFIRINOX [4]. Adding mitazalimab is assumed to increase the ORR to approximately 45%. Gemcitabine plus nab- paclitaxel is assumed to have similar ORR (29%) as mFOLFIRNOX [5] and all other assumptions are the same, hence, the sample size estimation is considered valid for both treatment options.
The sample size estimation is based on an extended Simon's two-stage design with break for futility and efficacy [37], however only the futility stop will be implemented. The optimal design was selected as it minimized the expected sample size. There will be a cut-off for number of responders in the interim analysis for stopping for futility. If the number of responders is above the cut-off the study continues. Table 12 presents the sample sizes.
Table 12 Extended Simon's two-stage design

Design restrictions: ORR ≥30% historic ORR=30%, alpha (one-sided)=0.10											
			First stage				Second stage				
ORR (%)	Power (%)	Design	n1	rfut [a]	reff [b]	ORReff [c]	n [a]	rfut	Est. N given H0	PETH0 (%)	PETH1 (%)
45	80	Optimal	23	7	11	52.2	54	20	34.17	63.96	42.87

[a] If number of responders is equal to or below this number the study is stopped for futility / not conclusive
[b] If number of responders is above this number the study is stopped for efficacy
[c] ORR needed in first stage to stop for efficacy (reff + 1 / n1)

PETH0 = Probability of Early Termination (both for futility and efficacy) given there is **no increase** in response rate when adding Mitazalimab
PETH1 = Probability of Early Termination (both for futility and efficacy) given there is **an increase** in response rate when adding Mitazalimab

Assuming a drop-out rate of 15% (not evaluable for response), the interim analysis will require a total of 27 enrolled patients at RP2D to reach 23 evaluable patients. Patient recruitment will continue during the interim analysis. If the study continues after interim analysis, a total number of 64 needs to be enrolled at the RP2D to reach 54 evaluable patients assuming the same drop- out rate as above.

12.3 Statistical methods

12.3.1 Efficacy analysis

12.3.1.1 Primary efficacy

The primary endpoint is the Objective Response Rate (ORR) defined as the proportion of patients achieving a confirmed complete response (CR) or partial response (PR) on the RECIST v. 1.1 at any time during the study.

For the interim analysis the ORR will be based on the response evaluation after 8 cycles for mitazalimab in combination with mFOLFIRINOX or 4 cycles for mitazalimab in combination with gemcitabine plus nab-paclitaxel (i.e., approximately 4 months after first dose). The response will not have to be confirmed as per RECIST v. 1.1 for the interim analysis.

The primary endpoint ORR will be compared between mitazalimab and historical control using a one-sided exact binomial test performed on the 10% significance level. Two-sided exact binomial 90% confidence intervals will also be presented. In addition, ORR will be presented for each study visit. Patients not evaluable for ORR and who completed at least 2 cycles will be considered as non-responders.

12.3.1.2 Secondary efficacy

All secondary efficacy endpoint will be presented using descriptive statistics.

Best Overall Response (BOR) for each patient will be defined as the best response from RECIST v. 1.1 categories CR, PR, SD and PD at any time during the study.

Disease control rate will be defined as either CR, PR or SD at each visit as determined by RECIST v. 1.1.

The duration of response (DoR) is defined as number of days from initial response of CR or PR to progressive disease or death due to underlying disease, whichever comes first.

Duration of Stable Disease (SD) is defined as number of days from first dose of mitazalimab to progressive disease or death, whichever comes first.

Time to next anti-cancer therapy is defined as number of days from the time from first dose of mitazalimab to next treatment initiated.

Kaplan-Meier curves will be presented for DoR, duration of SD and time to next anti-cancer therapy.
Progression-free survival (PFS) is defined as the number of days from first dose of mitazalimab to progressive disease or death due to any cause, whichever occurs first.
Overall survival (OS) is defined as the number of days from first dose of mitazalimab to date of death from any cause.
Kaplan-Meier curves will be presented for PFS and OS.

In addition, detection and characterization of anti-drug antibody (ADA) titers in serum will be summarized.

12.3.2 Safety analysis

Number or patients experiencing AEs and number of events will be summarized by SOC and preferred term. In addition, the following AEs will be presented separately:

- Incidence of DLT (Part 1)
- AEs Grade 3 or higher
- Serious AEs
- AEs leading to discontinuation of study treatment
- AEs leading to death

12.4 Analysis populations

Patients in Part 1 who are on the same dose regimen as patients in Part 2 will be pooled together with the Part 2 patients for the statistical analyses and data summaries.

12.4.1 Full Analysis Set (FAS)

The FAS includes all patients who received the combination of mitazalimab at the RP2D and chemotherapy for at least two treatment cycles for the mFOLFIRINOX combination or one cycle for the gemcitabine plus nab-paclitaxel combination. The FAS population will be used for all efficacy and explorative endpoints.

12.4.2 Safety Set

The safety set will comprise all patients that received any study treatment (mitazalimab) and will be used for all endpoints related to the safety objectives.

12.4.3 Per Protocol Set (PP)

The PP set is a subset of FAS and will comprise all patients that qualify for FAS and had at least 1 post-baseline efficacy measurement and no critical protocol deviations. PP will be used for all endpoints related to the efficacy objectives in addition to FAS.

12.4.4 Pharmacokinetic (PK) set

Evaluation of pharmacokinetic parameters will be based on the PK set. It will consist of all patients that received at least one infusion of mitazalimab who have no relevant protocol violations that affect the evaluation of PK parameters. More details will be given in the SAP.

12.5 Dose escalation in Part 1

Part 1 follows a Bayesian Optimal Interval (BOIN) design with at least 3 patients evaluable for DLT per dose level. The BOIN is described in statistical detail in reference [38] as well as in clinical application in reference [39]. It can be considered as a generalization of the 3+3, accelerated titration and 3+3+3 designs and is quite similar to these designs.

According to the BOIN design each time a cohort of patients has been completed, it is evaluated whether the next cohort should remain on the same dose, escalate to the next higher dose level above or de-escalate to the next lower dose level, according to the decision rules presented in Table 6.

The BOIN design shares the simplicity of the 3+3 design, which makes the decision of dose escalation/de-escalation by comparing $pp\hat{}$ with 0/3, 1/3, 2/3, 0/6, 1/6, and 2/6. In the BOIN design this decision is based on a comparison of $pp\hat{}$ with two pre-determined fixed boundaries, $\lambda\lambda ee$ and $\lambda\lambda dd$. Where $pp\hat{}$ is the observed DLT-rate (number of patients with DLT/number of patients treated) at the current dose level. In this study, the target toxicity level, i.e., the target DLT rate, is 30% and the

boundaries $\lambda\lambda ee$ =0.236 and $\lambda\lambda dd$ =0.359.

12.6 Interim analysis in Part 2

An interim analysis will be conducted when data from at least 23 evaluable patients at RP2D become available. Patients who have completed at least two treatment cycles of mitazalimab and mFOLFIRINOX combination or one cycle of the mitazalimab and gemcitabine plus nab-paclitaxel combination will be regarded as evaluable for the purpose of interim analysis, that will be performed according to the Simon's two-stage design. The cut-off limit for stopping for futility is 7 or fewer responders (ORR), i.e., if there are 8 responders or more, the study continues.

13 QUALITY CONTROL AND QUALITY ASSURANCE PROCEDURES

13.1 Monitoring of the study and regulatory compliance

The Project Manager, or designee, will make a Site Initiation Visit to each Institution to review the Clinical Study Protocol and its requirements with the Investigator(s), inspect the drug storage area if not performed at a prior study visit at the site, and fully inform the Investigator of his/her responsibilities and the procedures for assuring adequate and correct documentation. During the Site Initiation Visit, the eCRFs will be reviewed. Other pertinent study materials will also be reviewed with the Investigator's research staff.

During the course of the study, the CRA will make regular site visits in order to review protocol compliance, examine eCRFs and individual patient's medical records, drug accountability, and assure that the study is being conducted according to pertinent regulatory requirements. eCRF entries will be verified with source documentation. The review of medical records will be done in a manner to assure that patient confidentiality is maintained. In accordance with applicable national and local regulations, CRAs, Investigators and the Sponsor (or their designees) will only have access to patient information relevant to the study and will not have access to the full patient records.

13.2 Non-compliance to protocol

All instances of non-compliance and all resulting protocol deviations will be reviewed on a quarterly basis by the Sponsor's representatives (Theradex).

13.3 Curricula vitae and financial disclosure of Investigators

All Principal Investigators and all Sub-investigators will be required to provide a current signed and dated *curriculum vitae* and a financial disclosure statement to the Sponsor's representatives (Theradex).

13.4 Protocol modifications

No modification of the Clinical Study Protocol should be implemented without the prior written approval of the Sponsor or Sponsor's representatives (Theradex). Any such changes which may affect a patient's treatment or informed consent, especially those increasing potential risks, must receive prior approval by the ECs and the Competent Authorities. The exception to this is where modifications are necessary to eliminate an immediate hazard to patients, or when the change involves only logistical or administrative aspects of the study (e.g., change in Sponsor personnel, change in address or telephone number).

14 ETHICAL CONSIDERATIONS

14.1 Informed consent

The Investigator will obtain written informed consent from each patient, or their authorized representative, participating in the study. The informed consent form (ICF) must be signed, witnessed and dated. The ICF will contain all the Essential Elements of Informed Consent set forth in Title 21 CFR Part 50, the European Union Directive 2001/20/EC and its associated Detailed Guidance, European Union GCP Directive 2005/28/EC, European Union GDPR 2016/679, the ICH GCP, Section 4.8, and the terms of the Declaration of Helsinki (2013). A copy of the signed document should be given to the patient and the signed original ICF should be retained in the files of the Investigator/Institution in conformance with the Institution's Standard Operating Procedures.

The Investigator must comply with applicable regulatory requirement(s) and adhere to the ICH GCP and the terms of the Declaration of Helsinki (2013) when obtaining and documenting informed consent. The Investigator is responsible for seeking informed consent, and for ensuring that the information discussions prior to consent takes place undisturbed, however the task may be delegated by the Investigator to a medically qualified person (in accordance with local requirements).

The patient will be given verbal and written information in detail about the study, study procedures and risks. The patient should be given enough time to read the ICF and the opportunity to ask questions. The patient must voluntarily sign and date the written ICF before any study-specific procedures are being conducted.

If new information becomes available that may be relevant to the patient's willingness to continue participation in the study, the patient must be informed in a timely manner and a revised written ICF must be provided to the patient and a new written informed consent must be obtained.

The patients may withdraw consent at any time throughout the course of the study. The patients will be informed that the quality of their medical care will not be adversely affected in any way if they decline to participate in this study.

If a patient withdraws consent for disclosure of future information, the Sponsor may retain and continue to use any data collected before such a withdrawal of consent.

Patients who in the opinion of the investigator are deriving clinical benefit (without confirmed disease progression) may continue study treatment after the first 12 cycles and a separate consent to continue treatment will be obtained.

14.1.1 Initial contact with patients

Patients potentially eligible for the study may be offered referral by their treating oncologist to the clinics of the participating sites (if not a patient at the participating site). At these clinics, patients

are offered information about clinical studies and this specific study may be discussed. Patients will be offered the ICF to read. Patients who are currently under the care of the Investigator and patients who have previously participated in clinical studies may also be considered for participation. This may involve reviewing patient registers or medical records or arrangements with the responsible care organization(s).
The Investigator will discuss with the patient their right to have a family member, friend or acquaintance with them at the consultation.

14.1.2 Patient consideration of written and verbal information

The study will be discussed with the patient during a visit at the clinic. If agreeable, the patient will be given the ICF to take away and consider the study for at least 24 hours and to discuss it with anyone else he/she wishes to. Patients will be free to take as much time as they feel necessary to decide. If the study ends or recruitment stops, they will not be able to enroll in this study and the Investigator will discuss their other options. Investigators will not pressure patients to take part. Patients will be asked to return to the clinic sometime later to discuss the study further. If the patient wishes to proceed, he/she will then sign the ICF with an Investigator or appropriately medically qualified person.

14.2 Ethics Committee (EC)

The study will not be initiated without approval of the appropriate EC and compliance with all administrative requirements of the governing body of the Institution. This Clinical Study Protocol, consent procedures, and any Amendments must be approved by the EC in compliance with current regulations of the FDA and the European Union. Title 21 CFR Part 56, the European Union Directive 2001/20/EC and its associated Detailed Guidance, European Union GCP Directive 2005/28/EC, the ICH GCP, Sections 3 and 4, and the terms of the Declaration of Helsinki (2013), will be followed. A letter of approval will be sent to the Sponsor prior to initiation of the study and when any subsequent modifications are made. The EC will be kept informed by the Investigator, the Sponsor or the Sponsor's representative (Theradex), as required by national regulations, as to the progress of the study as well as to any serious and unexpected AEs.

14.3 Patient privacy

In order to maintain patient confidentiality, all eCRF pages, study reports and communications relating to the study will identify patients by assigned patient numbers; patients will not be identified by name. In accordance with local, national or federal regulations, the Investigator will allow the Sponsor or designee personnel access to all pertinent medical records in order to verify the data gathered on the eCRFs and to audit the data collection process. Regulatory agencies such as the French competent authority and the Belgian Competent authority may also request access to all study records, including source documentation for inspection. All clinical information will be handled as outlined, and consented to, by the patient within the ICF.

The applicable national laws on the processing of personal data will be complied to in countries with recruiting sites as well as in all countries to which patients' personal data are transferred to for research purposes. Personal information will be sent abroad for scientific analysis.
The study will be conducted in a manner that respects patients' physical and mental integrity as well as privacy.

14.4 Chain of custody of biological samples

A full chain of custody is maintained for all samples throughout their lifecycle. The Investigator, at each site, keeps full traceability of collected biological samples from the patients while in storage at the site until shipment or disposal (where appropriate). The Investigator will also ensure that access to the samples while in storage at the site will be limited only to those people for whom access is required.
Upon shipment, the sample receiver keeps full traceability of the samples while in storage and during use, until used or disposed of or until further shipment and keeps documentation of receipt of arrival.
The Sponsor keeps oversight of the entire life cycle through internal procedures, monitoring of sites and auditing of external laboratory providers.
All samples collected during the study will be destroyed after analysis or at the End of Trial. For long-term retention of samples, see Section 14.5 Long-term retention of samples for possible future analysis.

14.5 Long-term retention of samples for possible future analysis

Samples for possible future exploratory research will be collected and stored in a biobank, ClinStorage, in Sweden:

- Serum samples (see Section 10.13.1)
- Tumor Biopsy (samples remaining upon completion of the analyses described in Section 10.13.2)

The samples may be stored for up to approximately 5 years after the End of Trial and will only be used to investigate mitazalimab in relation to immuno-oncology, solid malignancies and to identify potential markers for safety and/or efficacy. Samples may also be used to develop test/assays in relation to this. The analysis of stored samples may begin at any time during the study or the post-study storage period.
Stored samples will be pseudonymized throughout the sample storage and analysis process and will not be labelled with personal identifiers (i.e., the samples will be coded with the same patient numbers as allocated in the clinical study).

Patients may withdraw their consent for their samples to be stored for research. If so, stored samples will be destroyed. If samples are already analyzed, the Sponsor is not obliged to destroy the results of this research.
Samples that have not been used approximately 5 years after the End of Trial will be destroyed in accordance with national guidelines.

14.6 Breach of GCP

The Principal Investigator must promptly provide the Sponsor or Sponsor's representatives (Theradex) with reports on any serious breaches of GCP or any change that is likely to significantly affect the safety of the patients, the conduct of the study or the scientific value of the study.

14.7 Finance

This clinical study was initiated and is funded by the commercial company Alligator Bioscience AB (i.e., the Sponsor). No non-commercial assistance providers are involved in this study. Alligator Bioscience AB will cover all costs for the procedures in this study that are not part of standard care. Cost for mFOLFIRINOX will be handled according to local regulations and will be specified in the Clinical Study Agreement.
The Investigators have no financial or other ties to Alligator Bioscience AB. A separate financial agreement (Clinical Study Agreement) will be signed between Alligator Bioscience AB and the Investigators and/or the Institution involved as required. The grants are paid to the Institution.
The patients will not be paid for taking part in the study, however the patients will be reimbursed for expenses in connection with the study visits, such as travel costs.

14.8 Insurance

This study is covered under the Sponsor's Liability Insurance Policy.

15 DATA HANDLING AND RECORD KEEPING

15.1 Recording of data

Data collected during the study will be entered on the patient's eCRF pages in the Electronic Remote Data Capture system, except the external data described in Section 15.2 by authorized study site personnel. The system is a fully validated secure web-based software (Viedoc®) which conform with 21 CFR part 11 requirements. Access to the system is only given after completed training. Electronic data queries will be used to communicate discrepant data with the study sites for inconsistent, not logical or missing data. Written instructions on how to complete the eCRF will be provided along with the training.

All information required by this Clinical Study Protocol should be provided; any omissions or corrections should be explained. All eCRFs should be completed and available within maximum 10 working days after the patient's visit (except prior to DRC meetings when data will need to be entered within a shorter time period) so that the CRA and Data Manager may check the entries for completeness and accuracy.

15.2 External data transfer

All electronic data will be transferred using a secure method accepted by Alligator according to separate Data Transfer Plan.

15.3 Source data

The Investigator must maintain source documents for each patient in the study according to the Source Data Agreement. The source documents are generally maintained in the patient's file. Detailed information about how to handle the source documents are further described in the Monitoring Plan.

The Investigator will provide direct access to his/her original records to permit the CRA to verify the proper transcription of data.

15.4 Coding of data

Medical history and AEs will be coded using MedDRA and CTCAE v. 5.0. SAEs and AESIs will be reconciled between the clinical database and the safety database.

Histopathology will be coded using ICD-O.

Concomitant medication will be coded using WHO Drug Reference List.

15.5 Investigator Site File (ISF)

The study site will be provided with an Investigator Site File (ISF) that should be used to store documentation pertaining to the study. It is the Principal Investigator's responsibility to continuously update the ISF. The ISF must be available to the CRA during monitoring visits.
The ISF will include, but not be limited to, the following documents:

- Signed Clinical Study Protocol and Amendments
- A copy of the Clinical Study Agreement, signed and dated
- EC approvals for the Clinical Study Protocol and Amendments
- Correspondence with ECs, Regulatory Authorities, Sponsor and Sponsor's representatives Theradex.

15.6 Retention of study records

The CRA will instruct the Investigator to maintain source documents and the signed ICF for each patient.
Furthermore, the CRA will instruct the Investigator to archive essential documents for the duration defined in ICH GCP/applicable regulations or for 15 years, whichever is longer.
The duration of archiving defined in ICH GCP is as follows:

Essential documents should be retained until at least 2 years after the last approval of a marketing application in an ICH region and until there are no pending or contemplated marketing applications in an ICH region or at least 2 years have elapsed since the formal discontinuation of clinical development of the investigational product. These documents should be retained for a longer period however if required by the applicable regulatory requirements or by an agreement with the Sponsor. The Sponsor will notify the Investigator when retention of the study-related records is no longer required to be retained.
At the end of the study, the site will be provided with all data related to the site (including eCRF data, queries and audit trail) using a secure media. It is the responsibility of the investigator to secure the storage of these data at site. When confirmation of receipt of the data, the access to the eCRF will be revoked. If the data is not readable for the full retention, the Investigator may request that the data be re-sent.

16 CLINICAL STUDY REPORT AND PUBLICATION POLICY

16.1 Clinical Study Report

A Clinical Study Report, in compliance with ICH E3; Structure and content of Clinical Study Reports, will be prepared describing the conduct of the study, the statistical analysis performed and the results obtained.

The Clinical Study Report, or where required the Clinical Study Report Synopsis, will be submitted to Competent Authorities and ECs within 12 months from the End of Trial.

16.2 Publication policy

The Sponsor endeavors to publish the results of clinical studies and is committed to ensure that the data are reported in a responsible and coherent manner. Positive, negative and inconclusive results will all be published.

The first publication will be based on data from all sites, analyzed as stipulated in this Clinical Study Protocol by Sponsor or Sponsor's representative statisticians. The publication of data collected from this study will be considered as a joint publication by the Investigators and the appropriate personnel of the Sponsor. The Sponsor will manage the publication of the study results in partnership with the authors; the principal author will take a leading role in this process. Co- authorship will be decided by the Sponsor and the Coordinating Investigator and will be limited to the number of Investigators who have contributed substantially to the study and have included more than 5% of the eligible patients. Investigators participating in the study agree not to present data gathered from one site (or a group of sites) before the initial multicenter publication. If a publication concerns the analyses of sub-sets of data from the study, the publication shall make reference to the relevant multicenter publication(s). If the multicenter publication is not submitted within 12 months after conclusion, abandonment or termination of the study at all sites, or after the Sponsor confirms that there will be no multicenter publication, the Institution/Principal Investigator may publish the data/results from the Institution individually in accordance with this section.

The data collected during the study are confidential and proprietary to the Sponsor. Any publications or abstracts arising from this study require approval by the Sponsor prior to publication or presentation and must adhere to the Sponsor's publication requirements as set forth in the approved Clinical Study Agreement. All draft publications, including abstracts or detailed summaries of any proposed presentations, must be submitted to the Sponsor at the earliest practicable time for review, but at any event not less than sixty (60) days before submission or presentation unless otherwise set forth in the Clinical Study Agreement. The Sponsor will review for accuracy (thus avoiding potential discrepancies with submissions to Regulatory Authorities), verify that confidential information is not being inadvertently disclosed, and provide any relevant

supplementary information. Sponsor may provide comments on the scientific conclusions of the study as it relates to mitazalimab in which case such comments will be discussed and considered in good faith by Investigator. The Sponsor will have the right to delete any confidential or proprietary information contained in any proposed publication, abstract or presentation and may delay publication for up to six (6) months, unless otherwise set forth in the Clinical Study Agreement, for purposes of filing a patent application.

17 STUDY AGREEMENTS

The Principal Investigator at the investigational site must comply with all the terms, conditions, and obligations of the Clinical Study Agreement for this study. In the event of any inconsistency between the Clinical Study Protocol and the Clinical Study Agreement, the Clinical Study Agreement shall prevail.

The Clinical Study Agreement will be filed in the ISF and the TMF.

18 REFERENCES

1. Puckett, Y. and K. Garfield, *Pancreatic Cancer*, in *StatPearls*. 2020: Treasure Island (FL).
2. Rawla, P., T. Sunkara, and V. Gaduputi, *Epidemiology of Pancreatic Cancer: Global Trends, Etiology and Risk Factors.* World J Oncol, 2019. **10**(1): p. 10-27.
3. Ducreux, M., et al., *Cancer of the pancreas: ESMO Clinical Practice Guidelines for diagnosis, treatment and follow-up.* Ann Oncol, 2015. **26 Suppl 5**: p. v56-68.
4. Conroy, T.e.a., *FOLFIRINOX versus Gemcitabine for Metastatic Pancreatic Cancer.* N Engl J Med, 2011(364): p. 1817-1825.
5. Von Hoff, D.D., et al., *Increased survival in pancreatic cancer with nab-paclitaxel plus gemcitabine.* N Engl J Med, 2013. **369**(18): p. 1691-703.
6. Elgueta, R., et al., *Molecular mechanism and function of CD40/CD40L engagement in the immune system.* Immunol Rev, 2009. **229**(1): p. 152-72.
7. Korniluk, A., H. Kemona, and V. Dymicka-Piekarska, *Multifunctional CD40L: pro- and anti-neoplastic activity.* Tumour Biol, 2014. **35**(10): p. 9447-57.
8. Peters, A.L., L.L. Stunz, and G.A. Bishop, *CD40 and autoimmunity: the dark side of a great activator.* Semin Immunol, 2009. **21**(5): p. 293-300.
9. Eliopoulos, A.G. and L.S. Young, *The role of the CD40 pathway in the pathogenesis and treatment of cancer.* Curr Opin Pharmacol, 2004. **4**(4): p. 360-7.
10. Vonderheide, R.H. and M.J. Glennie, *Agonistic CD40 antibodies and cancer therapy.* Clin Cancer Res, 2013. **19**(5): p. 1035-43.
11. Ma, D.Y. and E.A. Clark, *The role of CD40 and CD154/CD40L in dendritic cells.* Semin Immunol, 2009. **21**(5): p. 265-72.
12. Moran, A.E., M. Kovacsovics-Bankowski, and A.D. Weinberg, *The TNFRs OX40, 4-1BB, and CD40 as targets for cancer immunotherapy.* Curr Opin Immunol, 2013. **25**(2): p. 230-7.
13. Piechutta, M. and A.S. Berghoff, *New emerging targets in cancer immunotherapy: the role of Cluster of Differentiation 40 (CD40/TNFR5).* ESMO Open, 2019. **4**(Suppl 3): p. e000510.
14. Zarnegar, B., et al., *Unique CD40-mediated biological program in B cell activation requires both type 1 and type 2 NF-kappaB activation pathways.* Proc Natl Acad Sci U S A, 2004. **101**(21): p. 8108-13.
15. Rakhmilevich, A.L., K.L. Alderson, and P.M. Sondel, *T-cell-independent antitumor effects of CD40 ligation.* Int Rev Immunol, 2012. **31**(4): p. 267-78.
16. Calvo, E., et al., *A phase I study to assess safety, pharmacokinetics (PK), and pharmacodynamics (PD) of JNJ-64457107, a CD40 agonistic monoclonal antibody, in patients (pts) with advanced solid tumors.* Journal of Clinical Oncology, 2019. **37**(15_suppl): p. 2527-2527.
17. Vonderheide, R.H., et al., *Clinical activity and immune modulation in cancer patients treated with CP-870,893, a novel CD40 agonist monoclonal antibody.* J Clin Oncol, 2007. **25**(7): p. 876- 83.
18. Vonderheide, R.H., *CD40 Agonist Antibodies in Cancer Immunotherapy.* Annu Rev Med, 2020.

71: p. 47-58.

19. Beatty, G.L., et al., *A phase I study of an agonist CD40 monoclonal antibody (CP-870,893) in combination with gemcitabine in patients with advanced pancreatic ductal adenocarcinoma.* Clin Cancer Res, 2013. **19**(22): p. 6286-95.
20. O'Hara, M.H., et al., *Abstract CT004: A Phase Ib study of CD40 agonistic monoclonal antibody APX005M together with gemcitabine (Gem) and nab-paclitaxel (NP) with or without nivolumab (Nivo) in untreated metastatic ductal pancreatic adenocarcinoma (PDAC) patients.* Cancer Research, 2019. **79**(13 Supplement): p. CT004-CT004.

21. Yao, W., A. Maitra, and H. Ying, *Recent insights into the biology of pancreatic cancer.* EBioMedicine, 2020. **53**: p. 102655.
22. Vonderheide, R.H., *The Immune Revolution: A Case for Priming, Not Checkpoint.* Cancer Cell, 2018. **33**(4): p. 563-569.
23. Nowak, A.K., B.W. Robinson, and R.A. Lake, *Synergy between chemotherapy and immunotherapy in the treatment of established murine solid tumors.* Cancer Res, 2003. **63**(15): p. 4490-6.
24. Byrne, K.T. and R.H. Vonderheide, *CD40 Stimulation Obviates Innate Sensors and Drives T Cell Immunity in Cancer.* Cell Rep, 2016. **15**(12): p. 2719-32.
25. Beatty, G.L., et al., *CD40 agonists alter tumor stroma and show efficacy against pancreatic carcinoma in mice and humans.* Science (New York, NY), 2011. **331**.
26. Beatty, G.L., Y. Li, and K.B. Long, *Cancer immunotherapy: activating innate and adaptive immunity through CD40 agonists.* Expert Rev Anticancer Ther, 2017. **17**(2): p. 175-186.
27. Long, K.B., et al., *IFNgamma and CCL2 Cooperate to Redirect Tumor-Infiltrating Monocytes to Degrade Fibrosis and Enhance Chemotherapy Efficacy in Pancreatic Carcinoma.* Cancer Discov, 2016. **6**(4): p. 400-413.
28. Byrne, K.T., et al., *CSF-1R-Dependent Lethal Hepatotoxicity When Agonistic CD40 Antibody Is Given before but Not after Chemotherapy.* J Immunol, 2016. **197**(1): p. 179-87.
29. Mayes, P.A., K.W. Hance, and A. Hoos, *The promise and challenges of immune agonist antibody development in cancer.* Nat Rev Drug Discov, 2018. **17**(7): p. 509-527.
30. Lowery, M.A., et al., *Activity of front-line FOLFIRINOX (FFX) in stage III/IV pancreatic adenocarcinoma (PC) at Memorial Sloan-Kettering Cancer Center (MSKCC).* Journal of Clinical Oncology, 2012. **30**(15_suppl): p. 4057-4057.
31. Stein, S.M., et al., *Final analysis of a phase II study of modified FOLFIRINOX in locally advanced and metastatic pancreatic cancer.* Br J Cancer, 2016. **114**(7): p. 737-43.
32. Ko, A.H., et al., *A Phase I Study of FOLFIRINOX Plus IPI-926, a Hedgehog Pathway Inhibitor, for Advanced Pancreatic Adenocarcinoma.* Pancreas, 2016. **45**(3): p. 370-5.
33. Philip, P.A., et al., *Avenger 500, a phase III open-label randomized trial of the combination of CPI-613 with modified FOLFIRINOX (mFFX) versus FOLFIRINOX (FFX) in patients with metastatic adenocarcinoma of the pancreas.* Journal of Clinical Oncology, 2019. **37**(4_suppl): p. TPS479- TPS479.
34. Conroy, T., et al., *FOLFIRINOX or Gemcitabine as Adjuvant Therapy for Pancreatic Cancer.* N Engl J Med, 2018. **379**(25): p. 2395-2406.
35. Dahlberg, P., et al., *QT correction using Bazett's formula remains preferable in long QT syndrome type 1 and 2.* Ann Noninvasive Electrocardiol, 2021. **26**(1): p. e12804.
36. Oken, M.M., et al., *Toxicity and response criteria of the Eastern Cooperative Oncology Group.*
Am J Clin Oncol, 1982. **5**(6): p. 649-55.
37. Mander, A.P. and S.G. Thompson, *Two-stage designs optimal under the alternative hypothesis for phase II cancer clinical trials.* Contemp Clin Trials, 2010. **31**(6): p. 572-8.
38. Liu, S. and Y. Yuan, *Bayesian optimal interval designs for phase I clinical trials.* J R Stat Soc Ser C Appl Stat, 2015(64): p. 507–23.
39. Yuan, Y., et al., *Bayesian Optimal Interval Design: A Simple and Well-Performing Design for Phase I Oncology Trials.* Clin Cancer Res, 2016. **22**(17): p. 4291-301.
40. Boulanger, J., et al., *Management of the extravasation of anti-neoplastic agents.* Support Care Cancer, 2015. **23**(5): p. 1459-71.

19 APPENDICES

APPENDIX 1

Response Evaluation Criteria in Solid Tumors - RECIST v. 1.1 guideline

The revised RECIST v. 1.1 guideline are available at
https://ctep.cancer.gov/protocoldevelopment/docs/recist_guideline.pdf

Definitions

Response and progression will be evaluated in this trial using the international criteria (version 1.1) proposed by the Response Evaluation Criteria in Solid Tumors (RECIST) Committee [Eur J Cancer. 45 (2009) 228-247]. Changes in only the largest diameter (unidimensional measurement) of the tumor lesions are used in the RECIST v. 1.1 criteria. Note: Lesions are either measurable or non-measurable using the criteria provided below. The term "evaluable" in reference to measurability will not be used because it does not provide additional meaning or accuracy.

1. ***Measurable Disease***

Measurable disease is defined by the presence of at least one measurable lesion. Measurable lesions are defined as those that can be accurately measured in at least one dimension [longest diameter (LD) in the plane of measurement to be recorded] with a minimum size of:

- 10 mm by CT scan (CT scan slice thickness no greater than 5 mm)
- 10 mm caliper measurement by clinical exam (lesions which cannot be accurately measured with calipers should be recorded as non-measurable)
- 20 mm by chest x-ray

Malignant lymph nodes: To be considered pathologically enlarged and measurable, a lymph node must be ≥ 15 mm in short axis when assessed by CT scan (CT scan slice thickness no greater than 5 mm).

2. ***Non-measurable Disease***

All other lesions (or sites of disease), including small lesions (longest diameter < 10 mm or pathological lymph nodes with ≥ 10 to < 15 mm short axis) are considered non-measurable disease. Lesions considered truly non-measurable include: leptomeningeal disease, ascites, pleural/pericardial effusions, lymphangitis cutis/pulmonis, inflammatory breast disease, abdominal masses/abdominal organomegaly identified by physical exam and not followed by CT or MRI.
Bone lesions, cystic lesions and lesions previously treated with local therapy must be considered as follows:

Bone lesions:

- Bone scan, positron-emission tomography (PET) scan or plain films are not considered adequate imaging techniques to measure bone lesions. However, these techniques can be used to confirm the presence or disappearance of bone lesions.
- Lytic bone lesions or mixed lytic-blastic lesions, with identifiable soft tissue components, that can be evaluated by cross sectional imaging techniques (i.e., CT or MRI) can be considered as measurable lesions if the soft tissue component meets the definition of measurability described above.
- Blastic bone lesions are non-measurable. Cystic lesions:
- Lesions that meet the criteria for radiographically defined simple cysts should not be considered malignant lesions (neither measurable or non-measurable) since they are, by definition, simple cysts.
- 'Cystic lesions' thought to represent cystic metastases can be considered measurable lesions, if they meet the definition of measurability described above. However, if non-cystic lesions are present in the same subject, these are preferred for selection as target lesions.

Lesions with prior local treatment:

- Tumor lesions situated in a previously irradiated area, or in an area subjected to other loco-regional therapy, are usually not considered measurable unless there has been demonstrated progression in the lesion.

3. ***Target Lesions***

All measurable lesions up to a maximum of two lesions per organ and five lesions in total, representative of all involved organs, should be identified as target lesions and recorded and measured at baseline. Target lesions should be selected on the basis of their size (lesions with the longest diameter) and their suitability for accurate repeated measurements (either by imaging techniques or clinically). A sum of the diameters (longest for non-nodal lesions, short axis for nodal lesions) for all target lesions will be calculated and reported as the baseline sum diameters. The baseline sum diameters will be used as reference by which to characterize the objective tumor response.

4. ***Lymph Node Assessment***

For lymph nodes, measurements should be made of the short axis, which is defined as perpendicular to the LD of node assessed in the plane of measurement:

- Target lesion if short axis ≥ 15 mm
- Non-target lesion if short axis is ≥ 10 but < 15 mm
- Normal if short axis < 10 mm

For baseline, add the actual short axis measurement to the sum of LD of non-nodal lesions.

5. ***Non-target Lesions***

All other lesions (or sites of disease) including pathological lymph nodes should be identified as non-target lesions and should also be recorded at baseline. Measurements of these lesions are not required and these lesions should be followed as “present,” “absent,” or in rare cases “unequivocal progression.” In addition, it is possible to record multiple non-target lesions involving the same organ as a single item on the case report form (e.g., ‘multiple enlarged pelvic lymph nodes’ or ‘multiple liver metastases’).

Guidelines for Evaluation of Measurable Disease

All measurements should be taken and recorded in metric notation using a ruler or calipers. All baseline evaluations should be performed as closely as possible to the beginning of treatment and never more than 4 weeks before the beginning of the treatment.

The same method of assessment and the same technique should be used to characterize each identified and reported lesion at baseline and during follow-up. Imaging-based evaluation is preferred to evaluation by clinical examination when both methods have been used to assess the antitumor effect of a treatment.

Clinical lesions. Clinical lesions will only be considered measurable when they are superficial and ≥ 10 mm diameter as assessed using calipers (e.g., skin nodules). In the case of skin lesions, documentation by color photography, including a ruler to estimate the size of the lesion, is recommended. When lesions can be evaluated by both clinical exam and imaging, imaging evaluation should be undertaken since it is more objective and may be reviewed at the end of the trial.

Chest x-ray. Chest CT is preferred over chest x-ray, particularly when progression is an important endpoint. Lesions on chest x-ray may be considered measurable if they are clearly defined and surrounded by aerated lung.

Conventional CT and MRI. This guideline has defined measurability of lesions on CT scan based on the assumption that CT slice thickness is 5 mm or less. When CT scans have slice thickness > 5 mm, the minimum size for a measurable lesion should be twice the slice thickness. MRI is acceptable in certain situations (e.g., for body scans).

Ultrasound (US). US should not be used to measure tumor lesions. US examinations cannot be reproduced in their entirety for independent review at a later date because they are operator dependent. If new lesions are identified by US, confirmation by CT or MRI is advised. If there is concern about radiation exposure at CT, MRI may be used instead of CT.

Endoscopy, Laparoscopy. The utilization of these techniques for objective tumor evaluation is not advised. However, such techniques can be useful to confirm complete pathological response when

biopsies are obtained or to determine relapse in trials where recurrence following complete response or surgical resection is an endpoint.

Tumor markers. Tumor markers alone cannot be used to assess objective tumor response. If markers are initially above the upper normal limit, they must normalize for a subject to be considered in complete clinical response.

Cytology, Histology. These techniques can be used to differentiate between partial responses (PR) and complete responses (CR) in rare cases (e.g., residual lesions in tumor types such as germ cell tumors, where known residual benign tumors can remain).

Response Criteria

Evaluation of Target Lesions

Complete Response (CR): Disappearance of all target lesions. Any pathological lymph nodes (whether target or non-target) must have reduction in short axis to
< 10 mm.

Partial Response (PR): At least a 30% decrease in the sum of diameters of target lesions, taking as reference the baseline sum diameters.

Progressive Disease (PD): At least a 20% increase in the sum of diameters of target lesions, taking as reference the smallest sum on trial (this includes the baseline sum if that is the smallest). In addition to the relative increase of 20%, the sum must also demonstrate an absolute increase of at least 5 mm. The appearance of one or more new lesions is also considered progression.

Stable Disease (SD): Neither sufficient shrinkage to qualify for PR nor sufficient increase to qualify for PD, taking as reference the smallest sum diameters while in the trial.

a) Assessment of Target Lymph Nodes

Lymph nodes identified as target lesions should always have the actual short axis measurement recorded (measured in the same anatomical plane as the baseline exam), even if the nodes regress to below 10 mm in the trial. In order to qualify for CR, each node must achieve a short axis < 10 mm. For PR, SD and PD, the actual short axis measurement of the nodes is to be included in the sum of target lesions.

b) Target Lesions that Become “too small to measure”

All lesions (nodal and non-nodal) recorded at baseline should have their actual measurements recorded at each subsequent evaluation, even when very small (e.g., 2 mm). If it is the opinion of the radiologist that the lesion has disappeared, the measurement should be recorded as 0 mm. If

the lesion is believed to be present and is faintly seen but too small to measure, a default value of 5 mm should be assigned.

c) Lesions that Split or Coalesce on Treatment

When non-nodal lesions fragment, the longest diameters of the fragmented portions should be added together to calculate the target lesion sum. Similarly, as lesions coalesce, a plane between them may be maintained that would aid in obtaining diameter measurements of each individual lesion. If the lesions have truly coalesced such that they are no longer separable, the vector of the longest diameter should be the maximal longest diameter for the 'coalesced lesion.'

1. ***Evaluation of Non-Target Lesions***

Complete Response (CR): Disappearance of all non-target lesions and normalization of tumor marker level. All lymph nodes must be non-pathological in size (< 10 mm short axis).
Non-CR/Non-PD: Persistence of one or more non-target lesion(s) and/or maintenance of tumor marker level above the normal limits.
Progressive Disease (PD): Unequivocal progression of existing non-target lesions. (The appearance of one or more new lesions is also considered progression.) To achieve 'unequivocal progression' on the basis of the non-target disease, there must be an overall level of substantial worsening in non-target disease such that, even in the presence of SD or PR in target disease, the overall tumor burden has increased sufficiently to merit discontinuation.

2. ***New Lesions***

The finding of a new lesion should be unequivocal (i.e., not attributed to differences in scanning technique, change in imaging modality, or findings thought to represent something other than tumor, such as a 'new' healing bone lesion). A lesion identified on a follow-up trial in an anatomical location that was not scanned at baseline is considered a new lesion and will indicate disease progression. If a new lesion is equivocal, continued therapy and follow-up evaluation will clarify if it represents truly new disease. If repeat scans confirm this is definitely a new lesion, then progression should be declared using the date of the initial scan.

3. ***Evaluation of Best Overall Response***

The best overall response is the best response recorded from the start of the treatment until disease progression/recurrence (taking as reference for progressive disease the smallest measurements recorded since the treatment started). The subject's best overall response assignment will depend on findings of both target and non-target disease and will also take into consideration the appearance of new lesions. Furthermore, depending on the nature of the trial,

it may also require confirmatory measurement. Specifically, in non-randomized trials where response is the primary endpoint, confirmation of PR or CR is needed to deem either one the "best overall response."

It is assumed that at each protocol-specified time point, a response assessment occurs. Table 13 provides a summary of the overall response status calculation at each time point for subjects who have measurable disease at baseline.

Table 13 Time Point Response: Subjects with Target (+/- Non-target) Disease

Target Lesions	Non-target Lesions	New Lesions	Overall Response
CR	CR	No	CR
CR	Non-CR / non-PD	No	PR
CR	Not evaluated	No	PR
PR	Non-PD or not all evaluated	No	PR
SD	Non-PD or not all evaluated	No	SD
Not all evaluated	Non-PD	No	NE
PD	Any	Yes or No	PD
Any	PD	Yes or No	PD
Any	Any	Yes	PD
	CR=complete response, PR=partial response, SD=stable disease PD=progressive disease, NE=inevaluable		

Best response determination for studies where confirmation of CR or PR is required: Complete or partial responses may be claimed only if the criteria for each are confirmed by a repeat assessment at least 4 weeks later. In this circumstance, the best overall response can be interpreted as in Table 14.

Table 14 Best Overall Response when Confirmation of CR and PR Required

Overall response First time point	Overall response Subsequent time point	BEST overall response
CR	CR	CR
CR	PR	SD, PD or PR*
CR	SD	SD provided minimum criteria for SD duration met, otherwise PD

Overall response First time point	Overall response Subsequent time point	BEST overall response
CR	PD	SD provided minimum criteria for SD duration met, otherwise PD
CR	NE	SD provided minimum criteria for SD duration met, otherwise NE
PR	CR	PR
PR	PR	PR
PR	SD	SD
PR	PD	SD provided minimum criteria for SD duration met, otherwise PD
PR	NE	SD provided minimum criteria for SD duration met, otherwise NE
NE	NE	NE
CR=complete response; PR=partial response; SD=stable disease; PD=progressive disease; NE=inevaluable<br>* If CR is truly met at first time point, then any disease seen at a subsequent time point, even disease meeting PR criteria relative to baseline, makes the disease PD at that point (since disease must have reappeared after CR). Best response would depend on whether minimum duration for SD was met. However, sometimes 'CR' may be claimed when subsequent scans suggest small lesions were likely still present and in the fact subject had PR, not CR, at the first time point. Under<br>these circumstances, the original CR should be changed to PR and the best response is PR.		

Confirmatory Measurement/Duration of Response

1. ***Confirmation***

To be assigned a status of PR or CR, changes in tumor measurements must be confirmed by repeat assessments that should be performed 4 weeks after the criteria for response are first met. In the case of SD, follow-up measurements must have met the SD criteria at least once after trial entry at a minimum interval of 7 weeks.

2. ***Duration of Overall Response***

The duration of overall response is measured from the time measurement criteria are met for CR or PR (whichever is first recorded) until the first date that recurrent or progressive disease is objectively documented (taking as reference for progressive disease the smallest measurements recorded since the treatment started).

The duration of overall CR is measured from the time measurement criteria are first met for CR until the first date that recurrent disease is objectively documented.

3. ***Duration of Stable Disease***

Stable disease is measured from the start of the treatment until the criteria for progression are met, taking as reference the smallest measurements recorded since the treatment started.

APPENDIX 2 mFOLFIRINOX and permitted dose modifications

The guidelines for the permitted dose modification of mFOLFIRINOX during the study are described in this appendix. All other information related to mFOLFIRINOX treatment is described throughout the protocol.

DOSE MODIFICATIONS

Rules for Dose Omissions and Modified Schedules

The dose modifications for the mFOLFIRINOX treatment is outlined below. Toxicities are graded based upon CTCAE v 5.0, see protocol Section 11.2.3.3. Dose adjustments are to be made according to the system showing the greatest degree of toxicity.

Doses will be reduced, one level at a time, for hematologic and non-hematological toxicities.

- Three levels of dose modifications are permitted, for each constituent of mFOLFIRINOX, according to the criteria below (Table 15 Dose modifications for mFOLFIRINOX).
- If a toxicity requiring dose modification occurs following the third dose reduction of any constituent, additional dose reductions are not permitted. However, further treatment should be discussed with the Medical Monitor.
- Dose re-escalation is not permitted

Table 15 Dose modifications for mFOLFIRINOX

**Dose Level/ Constituent**	**Planned Dose**	**Dose modification steps**		
		**Dose Level - 1**	**Dose Level - 2**	**Dose Level - 3**
**Oxaliplatin**	85 mg/m2	60 mg/m2	50 mg/m2	40 mg/m2
**Leucovorin**	400 mg/m2	400 mg/m2	400 mg/m2	400 mg/m2
**Irinotecan**	150 mg/m2	120 mg/m2	100 mg/m2	80 mg/m2
**5-FU**	2400 mg/m2	1920 mg/m2	1600 mg/m2	1360 mg/m2

If treatment is held for more than 4 consecutive weeks for a mitazalimab related toxicity, patients should discontinue study treatment. However, if a patient is clinically benefitting at the end of a 4-week hold, the Investigator may contact the Medical Monitor to potentially continue protocol- based therapy. Modifications to the individual components of mFOLFIRINOX are allowed based on investigator's judgement in case the patient is continuing the study treatment beyond 12 cycles. Patients who discontinue mitazalimab treatment should perform the End of treatment visit and proceed to the post-treatment follow-up period. During the post-treatment follow-up period, patients will receive recommendation on standard of care treatment from Investigator.

Determination regarding the need for dose modification of 5-FU, irinotecan, oxaliplatin, and/or leucovorin should be made based on the guidelines according to system outlined below. Questions regarding adverse reactions, dose modifications, or toxicity management should be directed to the Medical Monitor. Management of toxicities and supportive care, except for where indicated below, can be performed as judged by the Investigator.

1. *Dose Modifications for Neutropenia and/or Thrombocytopenia at the Start of a Cycle or Within a Cycle*

**Blood count on the first day of mFOLFIRINOX dosing**	**Dose Delay**	**Dose reduction**		
		**Irinotecan**	**Oxaliplatin**	**5-FU and leucovorin**
Neutrophils ≥1.5 x 109/L<br>Thrombocytes ≥100 x 109/L	No delay	No dose reduction		
Neutrophils <1.5 x 109/L	Delay treatment until neutrophils ≥1.5 x 109/L	1st episode: dose reduction to 120 mg/m2<br>2nd episode: maintain dose at 120 mg/m2<br>3rd episode: discuss treatment stop or maintain only 5-FU and leucovorin	1st episode: no dose reduction<br>2nd episode: dose reduction to 60 mg/m2<br>3rd episode: discuss treatment stop or maintain only 5-FU and leucovorin	1st episode: no dose reduction
Thrombocytes <100 x 109/L	Delay treatment until thrombocytes ≥100 x 109/L<br>If no recovery within 14 days discuss treatment stop	1st episode: no dose reduction<br>2nd episode: dose reduction to 120 mg/m2<br>3rd episode: discuss treatment stop or maintain only 5-FU and leucovorin	1st episode: dose reduction to 60 mg/m2<br>2nd episode: maintain 60 mg/m2<br>3rd episode: discuss oxaliplatin stop	1st episode: no dose reduction<br>2nd episode: reduce the dose of iv continuous infusion by 25%

The use of prophylactic G-CSF is advised when there is a delay in treatment due to hematologic toxicity involving neutrophils (see also Section 5.3.4 Dose limiting toxicity criteria), Section 3.7 Potential risks associated with mitazalimab treatment and Section 8.6 Handling of infusion related reactions). G-CSF should first be started 24 hours after end of the cytotoxic chemotherapy.

Other hematologic toxicities do not require dose modification. However, red blood cell transfusion should be considered for hemoglobin <9.5 g/dL or significant symptoms of anemia or per institutional guidelines.

Measures for hematologic toxicity during the cycle (nadir values)

Events	Dose reduction for mFOLFIRINOX
Febrile neutropenia<br>Grade 4 neutropenia >7 days<br>Infection with grade 3-4 neutropenia	1st episode: dose reduction of irinotecan to 120 mg/m2 and add G-CSF if applicable<br>2nd episode: in addition reduce oxaliplatin to 60 mg/m2<br>3rd episode: discuss growth factor or further treatment reduction, maintain only 5-FU and leucovorin if necessary
Thrombocytopenia grade 3-4	1st episode: dose reduction of oxaliplatin to 60 mg/m2<br>2nd episode: in addition reduce irinotecan to 120 mg/m2 and reduce the dose of 5-FU iv continuous infusion by 25%<br>3rd episode: stop oxaliplatin and irinotecan, continue 5-FU and leucovorin

2. ***Dose Modifications for Diarrhea***

Events	Dose reduction for mFOLFIRINOX
Diarrhea grade 1-2	No dose modification; Initiate/optimize supportive care
Diarrhea grade 3-4 or<br>Diarrhea + fever and/or grade 3-4 neutropenia	1st episode: dose reduction of irinotecan to 120 mg/m2<br>2nd episode: dose reduction of oxaliplatin to 60 mg/m2 and reduce the dose of 5-FU iv continuous infusion by 25%<br>3rd episode: stop irinotecan
Recurrent diarrhea (>48 h) despite high doses of loperamide	No dose reduction for irinotecan, oxaliplatin and 5-FU after recovery except if Diarrhea grade 3-4 or diarrhea + fever and/or grade 3-4 neutropenia

For symptoms of diarrhea (and/or abdominal cramping) that occur at any time during a treatment cycle, it is suggested that patients should be instructed to take an anti-diarrheal, such as loperamide (2 mg every 2 hours until diarrhea resolves for 12 hours; 4 mg 4 hours at night is allowed) or diphenoxylate/atropine (Lomotil) as treatment for diarrhea.

For persisting diarrhea (i.e., lasting more than 48 hours) treatment with broad spectrum antibiotics, fluoroquinolone orally for 7 days. If severe diarrhea, hospitalization for parenteral rehydration and change to iv antibiotics should be considered.

Acute diarrhea and abdominal cramps, developing during or within 24 hours after irinotecan administration, may occur as part of a cholinergic syndrome. For irinotecan-related cholinergic reactions, the infusion time may be increased to mitigate these symptoms and prophylactic atropine per institutional guidelines is permitted.

3. *Dose Modifications for Drug Related Hepatic Toxicity*

For all hepatobiliary toxicity, hold treatment and evaluate for non-drug causes, e.g., biliary obstruction/stent malfunction (see also APPENDIX 7 DILI).

Once the underlying etiology is corrected and improving, resume therapy (5-FU and oxaliplatin only without irinotecan) at the previous dose level and add irinotecan once toxicity improved to ≤ Grade 1.

Use the following dose modification guidelines for hyperbilirubinemia:

1) Grade 2 and Grade 3 hyperbilirubinemia: Omit irinotecan until Grade ≤ 1 and resume at the same dose level
2) Grade 4 hyperbilirubinemia: Hold therapy until ≤ Grade 1 and resume at the next dose level once underlying etiology is corrected*

*Note: If the etiology of hyperbilirubinemia is from biliary obstruction (i.e., reversible and non-therapy related), discussion with the Medical Monitor may permit the option to continue irinotecan at original levels once liver function tests are resolved to ≤ Grade 1.

4. *Dose Modifications for Mucositis*

Mucositis as a toxicity is caused by 5-FU. If grade 3-4 toxicity occurs, continuous 5-FU iv infusion should be reduced by 25% for the remaining courses.

5. *Dose Modifications for Peripheral Neuropathy*

Only oxaliplatin is modified in the case of peripheral neuropathy:

1) Grade peripheral neuropathy: Continue monitoring at same dose level
2) Grade 2 peripheral neuropathy persisting more than 14 days: Decrease oxaliplatin by one dose level
3) Grade 3 peripheral neuropathy: Decrease oxaliplatin dose to 65 mg/m^2 ; if G3 peripheral neuropathy persists for more than 14 days, discontinue oxaliplatin.
4) Grade 4 peripheral neuropathy: Discontinue oxaliplatin, continue 5-FU, irinotecan and leucovorin at same dose level, if resolved to < Grade 1, therapy can be resumed on case-by- case basis after discussion with Medical Monitor

6. *Hand-Foot syndrome*

Hand-Foot syndrome as a toxicity is caused by 5-FU. If grade 3-4 toxicity occurs, continuous 5-FU iv infusion should be reduced by 25% for the remaining courses.

7. ***Dose Modifications for Other Clinically Significant Non-Hematologic* Toxicities (except alopecia and Grade 3 nausea and vomiting responding to medical treatment within 72 hours)***

**Toxicity and Grade**	**Suggested Dose Modification**				
	**Occurence**	**5-FU**	**Irinotecan**	**Oxaliplatin**	**Leucovorin**
Grade 3<br><br>Hold treatment until AE resolves to ≤ Grade 1.	First	Reduce the suspected offending agent by one dose level			
	Second	Reduce the suspected offending agent by one or more dose level(s)			
	Third	Discontinue the suspected offending agent			
	Fourth	Discuss with Medical Monitor			
Grade 4<br>Hold treatment until AE resolves to ≤ Grade 1.	First	Discontinue the suspected offending agent			
	Second	Discuss with Medical Monitor			
*Determination of "clinically significant" AEs and "offending drug" is at the discretion of the treating physician and/or the Medical Monitor.					

Gastrointestinal ulceration, regardless of whether it is hemorrhagic: hold 5-FU until symptoms resolved.
Acute laryngopharyngeal dysesthesia is due to oxaliplatin and should be handled by institutional practices which may include prolonged oxaliplatin infusion duration to 6 hours and infusion of 1g of calcium gluconate and 1g of magnesium sulfate over 15 min before all subsequent oxaliplatin infusions.

8. ***Dose Modifications for Infusion-Related Reactions***

Either institutional guidelines or those described below should be followed in case of infusion-related reactions to any chemotherapy component given per protocol. Infusion reactions will be defined according to the National Cancer Institute CTCAE (version 5.0) definitions of an allergic reaction or anaphylaxis as noted below.

**Toxicity and Grade**	**Suggested Dose Modification (any constituent of mFOLFIRINOX)**
Grade 1:<br>Transient flushing or rash, drug fever <38°C (100.4°F); intervention not indicated	• Reduce infusion rate of offending agent by 50%<br>• Monitor patient every 15 minutes for worsening of condition<br>• Future infusions may be administered at a reduced rate (e.g., over 60-120 minutes for irinotecan), at the discretion of the Investigator

**Toxicity and Grade**	**Suggested Dose Modification (any constituent of mFOLFIRINOX)**
	NOTE: Premedication with a combination of diphenhydramine hydrochloride 25-50 mg IV, dexamethasone 10-20 mg IV, and acetaminophen 650 mg orally or per institutional guidelines may be provided as part of subsequent treatments.
Grade 2:<br>Intervention or infusion interruption indicated; responds promptly to symptomatic treatment (e.g., antihistamines, NSAIDS, narcotics); prophylactic medications indicated for ≤24 hours.	• Stop infusion of offending agent<br>• Administer diphenhydramine hydrochloride 25-20 mg IV, acetaminophen 650 mg orally, and oxygen<br>• Resume infusion at 50% of the prior rate once infusion reaction has resolved<br>• Monitor patient every 15 minutes for worsening of condition<br>• For all subsequent infusions, pre-medicate with diphenhydramine hydrochloride 25-50 mg IV, dexamethasone 10-20 mg IV, and acetaminophen 650 mg orally<br>• Future infusions may be administered at a reduced rate (e.g., over 60-120 minutes for irinotecan), at the discretion of the Investigator.
Grade 3:<br>Prolonged (e.g., not rapidly responsive to symptomatic medication and/or brief interruption of infusion); recurrence of symptoms following initial improvement; hospitalization indicated for clinical sequelae (e.g., renal impairment, pulmonary infiltrates).	• Stop infusion and disconnect infusion tubing from patient<br>• Administer diphenhydramine hydrochloride 25-50 mg IV, dexamethasone 10-20 mg IV, bronchodilators for bronchospasm, and other medications or oxygen as medically necessary<br>• No further treatment will be permitted during this visit<br>• Consider consultation with allergist prior to subsequent treatment; de-sensitization to oxaliplatin as per institutional guidelines with premedication with a combination of diphenhydramine hydrochloride 25-50 mg IV, dexamethasone 10-20 mg IV, monteleucast 10 mg and acetaminophen 650 mg orally
Grade 4:<br>Life-threatening consequences; urgent intervention indicated.	• Stop the infusion and disconnect infusion tubing from patient<br>• Administer epinephrine, bronchodilators or oxygen as indicated for bronchospasm<br>• Administer diphenhydramine hydrochloride 50 mg IV, dexamethasone 10-20 mg IV and other medications as medically necessary<br>• Consider hospital admission for observation<br>• No further treatment will be permitted during this visit

9. *Cardiac toxicity*

In case of cardiac pain, e.g., angina pectoris or myocardial infarction, 5-FU treatment shall be stopped. The reintroduction of 5-FU is not allowed.

10. *Extravasation*

Severe reactions due to irinotecan or oxaliplatin extravasation have been reported [40]. General recommendations in case of extravasation are as follow:

- stop infusion immediately,
- do not remove the needle or the catheter,
- suck/aspirate the maximum of infiltrated product through the needle,
- apply ice on the infiltrated area for 15 to 20 minutes every 4 to 6 hours for a period of 72 hours,
- apply local corticotherapy,

check regularly the infiltrated site during the following days, to verify whether more treatment is needed. Do not hesitate to require a surgical consultation in case of doubt.

APPENDIX 3

Gemcitabine plus nab-paclitaxel administration including dosing schedule, visit assessment tables and potential dose modifications (if applicable)

Please note that gemcitabine plus nab-paclitaxel treatment only applies for this study in case the Data Review Committee (DRC) has taken a formal decision during Part 1 of the study to change the chemotherapy mFOLFIRINOX to gemcitabine plus nab-paclitaxel as described in Section 5.1.

DOSE MODIFICATIONS

Dose adjustments are to be made according to the system showing the greatest degree of toxicity. Doses will be reduced, one level at a time, for hematologic and non-hematological toxicities.

- Two levels of dose modifications are permitted, for each drug, according to the criteria below, see Table 16. If a toxicity requiring dose modification occurs following the second dose gemcitabine and nab-paclitaxel reduction of either drug, additional dose reductions are not permitted. However, further treatment should be discussed with the Medical Monitor.
- Dose re-escalation is not permitted.

Table 16 Dose modifications for gemcitabine and nab-paclitaxel

Dose Level/ Agent	Planned Dose	Dose modification steps	
		**Dose Level -1**	**Dose Level -2**
**Nab-paclitaxel**	125 mg/m2	100 mg/m2	75 mg/m2
**Gemcitabine**	1000 mg/m2	800 mg/m2	600 mg/m2

If treatment is held for >3 consecutive weeks for a treatment related toxicity, patients should stop all study treatment. However, if a patient is clinically benefitting at the end of a 3-week hold, treating physicians may contact the Medical Monitor to potentially extend therapy. Patients who discontinue study treatment should perform End of treatment visit and enter post-treatment follow up period, please see Table 21 and Table 22 respectively. After the End of treatment visit, patients will receive recommendation on standard of care treatment from Investigator.

In all situations where toxicity justifies discontinuation of an agent, only the individual offending agent should be removed from the regimen and treatment should continue otherwise per protocol.

Determination regarding the need for dose modifications of nab-paclitaxel and/or gemcitabine should be made based on the following guidelines. Questions regarding adverse reactions, dose modifications, or toxicity management should be directed to the Medical Monitor.

Table 17 Dose Modifications for Neutropenia and/or Thrombocytopenia at the start of a cycle or within a cycle

Cycle Day	Neutrophils		Thrombocytes	Nab-paclitaxel Dose	Gemcitabine Dose
**Day 1**	≥ 1.5 x 109/L	AND	≥ 100 x 109/L	Treat on time at current dose levels	
	< 1.5 x 109/L	OR	< 100 x 109/L	Delay doses until recovery	
**Day 8**	≥ 1 x 109/L	AND	≥ 75 x 109/L	Treat on time at current dose levels	
	≥ 0.5 x 109/L to < 1 x 109/L	OR	≥ 50 x 109/L to < 100 x 109/L	Reduce to DL-1	
	< 0.5 x 109/L	OR	< 50 x 109/L	Withhold doses	
**Day 15, if Day 8 doses were given without modification or reduced:**					
**Day 15**	≥ 1 x 109/L	AND	≥75 x 109/L	Treat on time at current dose levels	
	≥ 0.5 x 109/L to < 1 x 109/L	OR	≥ 50 x 109/L to 75 x 109/L	Reduce 1 dose level from Day 8	
	< 0.5 x 109/L	OR	< 50 x 109/L	Withhold doses	
**Day 15:**	**If Day 8 doses were withheld**				
	> 1 x 109/L	AND	≥75 x 109/L	Reduce 1 dose level from Day 8	
	≥ 0.50 x 109/L to ≤ 1 x 109/L	OR	≥ 50 x 109/L to < 75 x 109/L	Reduce 2 dose levels from Day 1	
	< 0.5 x 109/L	OR	< 50 x 109/L	Withhold doses	

The use of prophylactic G-CSF is advised when there is a delay in treatment due to hematologic toxicity involving neutrophils. G-CSF should first be started 24 hours after end of the cytotoxic chemotherapy.
If hematologic toxicity is restricted to platelet counts alone, dose modification of only gemcitabine could be considered after discussion with the Medical Monitor.
Other hematologic toxicities do not require dose modification. However, red blood cell transfusion should be considered for hemoglobin <9.5 g/dL or significant symptoms of anemia or per institutional guidelines.

Table 18 Dose Modifications for Other Clinically Significant Non-Hematologic* Toxicities

Adverse Drug Reaction	Nab-paclitaxel Dose	Gemcitabine Dose
**Febrile Neutropenia: Grade 3 or 4**	Withhold doses until fever resolves and ANC is ≥ 1.5 x 109/L resume at next lower dose level for both agents	
**Peripheral Neuropathy: Grade 3 or 4**	Withhold dose until improvement to ≤ Grade 1; Resume at next lower dose level	Treat with same dose
**Cutaneous Toxicity: Grade 2 or 3**	Reduce doses to next lower dose level; discontinue treatment if toxicity persists	
**Gastrointestinal Toxicity: Grade 3 mucositis or diarrhea**	Withhold until improves to ≤ Grade 1; Resume at next lower dose level	

For all other ≥ Grade 3 non-hematologic toxicities (*except nausea, vomiting, alopecia and pulmonary embolism and Adverse events of special interest (AESIs) described below.

- Withhold dose of either or both agent(s) until improvement to ≤ Grade 1
- Resume at next lower dose level

Hepatotoxicity and dose reduction for Gemcitabine plus nab-paclitaxel

- If the AST or ALT or both increase is less than 5 x ULN, gemcitabine will be continued without dose reduction
- If the AST or ALT or both increase is more than 5 x ULN, but less than 20 x ULN, gemcitabine dose must be reduced by 25%
- If the AST or ALT or both increase is more 20 x ULN, gemcitabine must be stopped

DOSAGE SCHEDULE

The first treatment cycle with mitazalimab and gemcitabine plus nab-paclitaxel will last 35-days, mitazalimab will be administered on Day 1, Day 10 and 24 and gemcitabine plus nab-paclitaxel on Day 8, 15 and 22. During the following 28-day treatment cycles, mitazalimab will be administered on Day 3 and 17 and gemcitabine plus nab-paclitaxel on Day 1, 8 and 15.

A maximum of 6 treatment cycles will be allowed for mitazalimab and gemcitabine plus nab-paclitaxel combination, see Figure 4.

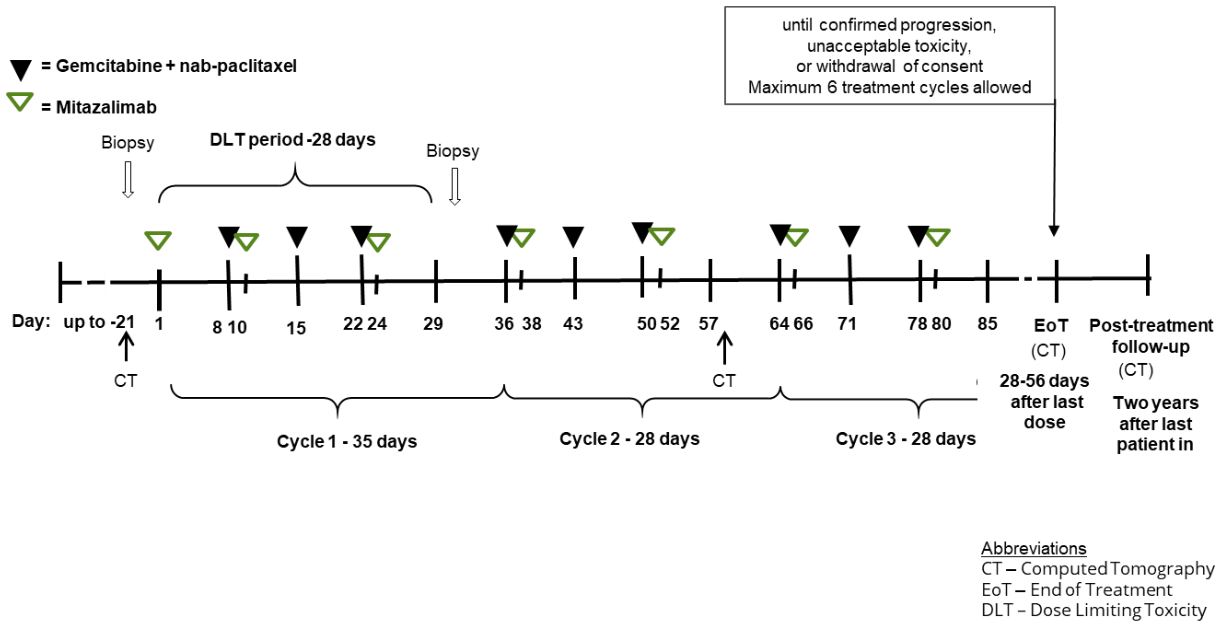

Figure 4 Dosage schedule for mitazalimab and gemcitabine plus nab-paclitaxel

Visit assessment schedules to be used if gemcitabine plus nab-paclitaxel are to replace mFOLFIRINOX in the study as decided by DRC.

Table 19 Assessment schedule during screening period and up to Day 14 of Treatment cycle 1

**Activity/Visit**	**Screening**	**Cycle 1 (35-day treatment cycle)10 – Day 1 to14**									
**Day**	**-21 to -1**	**1**			**2**	**8±1**		**10±1**			**11±1**
**Time in relation to study drug**		**Pre**	**Mitazalimab**	**Post1**	**24±2h1**	**Pre**	**Gem-NabPac**	**Pre**	**Mitazalimab 2**	**Post1**	**24±2h1**
**Informed Consent** (Section 14.1*)	X3										
**Eligibility criteria** (Section 7.1 and 7.2)	X										
**Demographics** (Section 10.1)	X										
**Medical history** (Section 10.2)	X										
**Body weight** (Section 10.3)	X	X4									
**Height** (Section 10.3)	X										
**Vital signs** (Section 10.4)	X	X		1h±10min<br>2h±10min<br>4h±15min		X				1h±10min<br>2h±10min	
**Physical examination** (Section 10.5)	X	X4				X					
**12-lead ECG** (Section 10.6)	X	X4		1h±10min<br>4h±15min						1h±10min	
**ECOG** (Section 10.7)	X	X4									

**Treatment discontinuation criteria** (Section 9.1)		X5				X5		X5			
**Pre- and postmedication**		**Note**: Premedications are to be given up to 3 days prior to administration of mitazalimab. For detailed information of timing of pre-and<br>postmedications for mitazalimab, please see protocol Section 8.9.3.									
**Mitazalimab administration** (Section 8.3)			X6						X6		
**Gem-NabPac administration** (See Section 8.9.4.1)											

**Activity/Visit**	**Screening**	**Cycle 1 (35-day treatment cycle)10 – Day 1 to14**									
**Day**	**-21 to -1**	**1**			**2**	**8±1**		**10±1**			**11±1**
**Time in relation to study drug**		**Pre**	**Mitazalimab**	**Post1**	**24±2h1**	**Pre**	**Gem-NabPac**	**Pre**	**Mitazalimab 2**	**Post1**	**24±2h1**
Nab-paclitaxel							X				
Gemcitabine							X				
**Adverse events** (Section 11.2)	Continuous from signing ICF until 28 days after last dose of IMP										
**Concomitant medication** (Section 8.9.7)	Continuous from signing of ICF until End of treatment visit										
**Concomitant medical procedures** (Section 10.8)	Continuous from signing of ICF until End of treatment visit										
**Clin laboratory tests** (Section 10.9)											
Pregnancy test	X	X7									
Clinical chemistry, hematology and coagulation	X	X4				X4		X			
Urinalysis	X	X4				X4					
**Pharmacokinetics** (Section 10.1110.10)		X4		5min, 1h±10min 2h±10min 4h±20min	X	X		X		5min, 1h±10min 2h±10min 4h±20min	X
**Immunogenicity** (Section 10.10)		X4									
**CT scan/Tumor response evaluation** (Section 10.12)	X8										
**CA19-9** (Section 10.13.1)	X	X4									X

**Immunophenotyping** (Section 10.13.1)		X4			X	X					X
**Cytokines and chemokines** (Section 10.13.1)		X4		1h±10min, 4h±20min	X	X		X		1h±10min, 4h±20min	X
**Whole blood RNA** (Section 10.13.1)		X4		1h±10min, 4h±20min	X	X		X		1h±10min, 4h±20min	

**Activity/Visit**	**Screening**	**Cycle 1 (35-day treatment cycle)10 – Day 1 to14**									
**Day**	**-21 to -1**	**1**			**2**	**8±1**		**10±1**			**11±1**
**Time in relation to study drug**		**Pre**	**Mitazalimab**	**Post1**	**24±2h1**	**Pre**	**Gem-NabPac**	**Pre**	**Mitazalimab2**	**Post1**	**24±2h1**
**Whole blood DNA** (Section 10.13.1)	X										
**Tumor biopsy** (Section 10.13.2)	X9										

* The sections referred to in the table are the sections in the main body of the protocol.

1. Assessments to be performed at specified time after End of infusion. The End of infusion is defined as when the infusion of IMP is completed/stopped (i.e., before rinsing if applicable). For example, 4h post-dose means 4 hours after end of infusion.
2. Mitazalimab should be administered 2 days following gemcitabine administration.
3. Informed consent must be obtained before or at screening prior to performing any screening assessments.
4. The assessment may be performed within 72 hours (up to 3 days) prior to administration of study treatment. If the screening assessment were taken Day -1 to -3 prior to dosing, it does not need to be repeated at Day 1.
5. If any of the treatment discontinuation criteria apply, the patient should be discontinued from treatment and the End of treatment visit should be performed, see End of treatment visit in Table 21 Assessment schedule for Treatment cycle 2 and subsequent cycles.
6. If the infusion of mitazalimab is interrupted due to an AE, a PK sample and a sample for immunogenicity should be taken at the time of interruption, or as soon as is feasible considering the patient safety. An immunogenicity sample do not need to be taken if it is first administration of mitazalimab that is interrupted.
7. The result of the pregnancy test must be available prior to dosing.
8. The CT scan at screening may be obtained 28 days prior to first dose of mitazalimab.
9. The baseline biopsy may be collected up to 28 days before first dose. If a fresh biopsy cannot be taken during screening period, archival biopsy material (most recent) could be used.
10. The DLT evaluation period includes Day 1 to 28 of the full 35-day treatment cycle.

Table 20 Assessment schedule for Day 15-35 of Treatment cycle 1

**Activity/Visit**	**Cycle 1 (35-day treatment cycle)11 – Day 15 to 35**								**Unscheduled visit1**
**Day**	**15±1**		**22±1**		**24±1**			**29±4**	
**Time in relation to study drug**	**Pre**	**Gem-NabPac**	**Pre**	**Gem-NabPac**	**Pre**	**Mitazalimab 3**	**Post2**	**24±2h2**	
**Body weight** (Section 10.3)	X4								X
**Height** (Section 10.3)									
**Vital signs** (Section 10.4)	X		X				1h±10min		X
**Physical examination** (Section 10.5)	X4		X4						X
**12-lead ECG** (Section 10.6)							1h±10min		X
**ECOG** (Section 10.7)									X
**Treatment discontinuation criteria** (Section 9.1)	X5		X5		X5				X5
**Pre- and postmedication**	**Note**: Premedications are to be given up to 3 days prior to administration of mitazalimab. For detailed information of timing of pre-and postmedications for mitazalimab, please see Section 8.9.1.								
**Mitazalimab administration** (Section 8.3)						X6			
**Gem-NabPac administration** (Section 8.9.4.1)									
Nab-paclitaxel		X		X					
Gemcitabine		X		X					
**Adverse events** (Section 11.2)	Continuous from signing ICF until 28 days after last dose of study treatment								
**Concomitant medication** (Section	Continuous from signing of ICF until End of treatment visit								

8.9.7)									
**Concomitant medical procedures**<br>(Section 10.8)	Continuous from signing of ICF until End of treatment visit								
**Clin laboratory tests**<br>(Section 10.9)									
Pregnancy test									X
Clinical chemistry, hematology and coagulation	X4		X4		X				X

**Activity/Visit**	**Cycle 1 (35-day treatment cycle)11 – Day 15 to 35**								**Unscheduled visit1**
**Day**	**15±1**		**22±1**		**24±1**			**29±4**	
**Time in relation to study drug**	**Pre**	**Gem-NabPac**	**Pre**	**Gem-NabPac**	**Pre**	**Mitazalimab 3**	**Post2**	**24±2h2**	
Urinalysis	X4		X4						X
**Pharmacokinetics** (Section 10.11)					X		5min<br>1h±10min<br>2h±10min<br>4h±20min	X	X
**Immunogenicity** (Section 10.10)									X
**CT scan/Tumor response evaluation** (Section 10.12)									X
**CA19-9** (Section 10.13.1)									X
**Immunophenotyping** (Section 10.13.1)	X								X
**Cytokines and chemokines** (Section 10.13.1)	X								X
**Whole blood RNA** (Section 10.13.1)	X								
**Whole blood DNA** (Section 10.13.1)									
**Tumor biopsy** (Section 10.13.2)								X7	X 7, 8

* The sections referred to in the table are the sections in the main body of the protocol.

1. The assessments to be performed during the Unscheduled visit should be based on Investigator's judgement.

2. Assessments to be performed at specified time after End of infusion. The End of infusion is defined as when the infusion of IMP is completed/stopped (i.e., before rinsing if applicable). For example, 4h post-dose means 4 hours after end of infusion.
3. Mitazalimab should be administered 2 days after gemcitabine administration
4. The assessment may be performed within 72 hours (up to 3 days) prior to administration of study treatment.
5. If any of the treatment discontinuation criteria apply, the patient should be discontinued from treatment and the End of treatment visit should be performed, see End of treatment visit in Table 21 Assessment schedule for Treatment cycle 2 and subsequent cycles.
6. If the infusion of mitazalimab is interrupted due to an AE, a PK sample and a sample for immunogenicity should be taken at the time of interruption, or as soon as is feasible considering the patient safety. An immunogenicity sample do not need to be taken if it is first administration of mitazalimab that is interrupted.

7. Biopsy to be taken only if baseline biopsy (fresh or archival) was obtained at screening and second biopsy not obtained during Treatment cycle 1.
8. Biopsy not to be taken if already obtained in Cycle 1

Table 21 Assessment schedule for Treatment cycle 2 and subsequent cycles

**Activity/Visit**	**Cycle 2 and subsequent cycles (28-day treatment cycles)**													**Unscheduled visit[1]**	**End of treatment visit[2]**
**Cycle day**	**1[3]**		**3±1**			**8±1**		**15±1**		**17±1**			**24 ±2**		
**Time in relation to study drug administration**	**Pre**	**Gem-NabPac**	**Pre**	**Mitazalimab[4]**	**Post[5]**	**Pre**	**Gem-NabPac**	**Pre**	**Gem-NabPac**	**Pre**	**Mitazalimab[4]**	**Post[5]**			**28-56 days after last dose**
**Body weight** (Section 10.3*)	X[6]													X	
**Vital signs** (Section 10.4)	X		X		1h±10min	X		X		X		1h±10min	X	X	X
**Physical examination** (Section 10.5)	X[6]		X[6]			X[6]		X[6]		X[6]			X	X	X
**12-lead ECG** (Section 10.6)					1h±10min[12]									X	X
**ECOG** (Section 10.7)	X[6]													X	X
**Treatment discontinuation criteria** (Section 9.1)	X[7]		X[7]			X[7]		X[7]		X[7]				X[7]	
**Pre- and postmedication**	**Note:** Premedications are to be given up to 3 days prior to administration of mitazalimab. For detailed information of timing of pre-and postmedications for mitazalimab, please see protocol Section 8.9.1.														
**Gem-NabPac administration** (See Section 8.9.4.1)															
Nab-paclitaxel		X					X		X						
Gemcitabine		X					X		X						

**Mitazalimab administration** (Section 8.3)				X8							X8				
**Adverse events** (Section 11.2)	Continuous from signing ICF until 28 days after last dose of study treatment														
**Concomitant medication** (Section 8.9.7)	Continuous from signing of ICF until End of treatment visit														
**Concomitant medical procedures** (Section 10.8)	Continuous from signing of ICF until End of treatment visit														
**Clin laboratory tests** (Section 10.9)															
Pregnancy test	X9													X	X

**Activity/Visit**	**Cycle 2 and subsequent cycles (28-day treatment cycles)**													**Unscheduled visit1**	**End of treatment visit2**
**Cycle day**	**13**		**3+1**			**8±1**		**15±1**		**17±1**			**24 ±2**		
**Time in relation to study drug administration**	**Pre**	**Gem-NabPac**	**Pre**	**Mitazalimab4**	**Post5**	**Pre**	**Gem-NabPac**	**Pre**	**Gem-NabPac**	**Pre**	**Mitazalimab4**	**Post5**			**28-56 days after last dose**
Clinical chemistry, hematology and coagulation	X6		X6			X6		X6		X6			X	X	X
Urinalysis	X6												X	X	X
**Pharmacokinetics** (Section10.11)	X				1h±10min									X	X
**Immunogenicity** (Section10.10)	X													X	X
**CT scan/Tumor response evaluation** (Section 10.12)													X (Cycle 2, 4, 6)	X	X
**CA19-9** (Section 10.13.1)	X													X	X
**Immunophenotyping** (Section 10.13.1)	X													X	
**Cytokines and chemokines** (Section 10.13.1)	X													X	
**Whole blood RNA** (Section 10.13.1)	X														

**Whole blood DNA** (Section 10.13.1)	X														
**Tumor biopsy** (Section 10.13.2														X10, 11	X 10, 11

* The sections referred to in the table are the sections in the main body of the protocol.

1. The assessments to be performed during the Unscheduled visit should be based on Investigator's judgement.
2. After completion of the End of treatment visit, the patient will either enter the post-treatment follow-up period (see Table 22), or if any of the study withdrawal criteria in Protocol Section 9.3 apply, the patient will be withdrawn from the study and have the End of treatment visit and End of study visit assessments performed, see End of study visit in Table 22).

3. Day 1 must be at least 14 days after the last gemcitabine plus nab-paclitaxel administration.
4. Mitazalimab should be administered 2 days after gemcitabine administration.
5. Assessments to be performed at specified time after End of infusion. The End of infusion is defined as when the infusion of study treatment is completed/stopped (i.e., before rinsing if applicable). For example, 1h post-dose means 1 hour after end of infusion.
6. The assessment may be performed within 72 hours (i.e., up to 3 days) prior to administration of study treatment.
7. If any of the treatment discontinuation criteria apply, the patient should be discontinued from treatment and the End of treatment visit should be performed
8. If the infusion of mitazalimab is interrupted due to an AE, a PK sample and a sample for immunogenicity should be taken at the time of interruption, or as soon as is feasible considering patient safety.
9. The result of the pregnancy test must be available prior to dosing.
10. Biopsy to be taken only if baseline biopsy (fresh or archival) was obtained at screening.
11. Biopsy not to be taken if already obtained in Cycle 1 or 2.
12. Assessment to be obtained in Cycle 2 only

Table 22 Assessment schedule for Post-treatment follow-up period and End of study visit

**Activity/Visit**	**Post-treatment follow-up**		**End of study visit4**
**Time after End of treatment visit**	**1 month**	**3 months, and thereafter every 3rd month ± 14 days**	**2 years after LPI**
**Disease/Survival status** (Section 10.14)	X1	X1	X1
**Subsequent cancer-related therapy** (Section 10.15)	X1	X1	X1
**CT scan/Tumor response evaluation** (Section 10.12)		X2	X
**Study withdrawal criteria** (Section 9.3)	X3	X3	X

* The sections referred to in the table are the sections in the main body of the protocol.

1. Disease/Survival status and subsequent cancer related therapy may be followed up via the patient's medical records or phone contact.
2. Assessment to be performed until new cancer-related therapy/continued treatment with chemotherapy backbone alone.
3. If any of the study withdrawal criteria in Protocol Section 9.3 apply, the patient will be withdrawn from the study and have the End of study visit performed.
4. End of study visit will be performed 2 years after LPI, i.e., there will be a variation of duration of the treatment follow-up period for individual patients

APPENDIX 4

Calculation of glomerular filtration rate

Glomerular filtration rate (GFR) may be estimated based on commonly used and accepted formulae, i.e., one of the below formula.

Cockroft Gault formula:

$$GFR = \frac{(140 - age) \times weight \times F_S}{Serum\ Creatinine \times 72}$$

Units: GFR [ml/min], age [years], weight [kg], serum creatinine [mg/dl], FS is a correction Factor for Sex: in males FS = 1, in females FS = 0.85

Modification of Diet in Renal Disease (MDRD) formula:

$$GFR = 170 \times Serum\ Creatinine^{-0.999} \times Age^{-0,176} \times BUN^{-0.170} \times Albumin^{+0.318} \times F_S$$

Units: GFR [ml/min], age [years], serum creatinine [mg/dl], FS is a correction Factor for Sex: in males FS
= 1, in females FS = 0.762

Variations of the MDRD formula:

$$GFR = 186 \times Serum\ Creatinine^{-1.154} \times Age^{-0,203} \times F_S$$

Units: GFR [ml/min], age [years], serum creatinine [mg/dl], FS is a correction Factor for Sex: in males FS = 1, in females FS = 0.742

Highly effective forms of contraception.

APPENDIX 5

Clinical Trials Facilitation and Coordination Group

CTFG

Recommendations related to contraception and pregnancy testing in clinical trials

Version 1.1

Introduction and scope

The aim of this document is to supplement existing guidelines related to embryofetal risk mitigation and to provide practical guidance on contraception use and pregnancy testing in clinical trials. It is not the aim of this document to discuss when women of childbearing potential may be included in clinical trials or to discuss treatment of pregnant women with investigational medicinal products (IMPs) in clinical trials. In this guidance document it is assumed that treatment with the IMP will be interrupted in case of pregnancy. For this reason, the relevant data for risk assessment cover risks in the early stages of pregnancy only. The recommendations in this document are intended for sponsors of clinical trials seeking to meet regulatory expectations for submission of application dossiers for clinical trials with IMPs in accordance with Directive 2001/20/EC. Deviations from these recommendations should be justified by the sponsor. This guidance applies to all IMPs, with the exception of advanced therapy medicinal products (ATMP). For ATMP products, embryofetal risk assessment and the need for contraception and pregnancy testing recommendations should be considered on a case-by-case basis.

This document should be read in conjunction with published guidelines and in particular the following:

- Non-Clinical Safety Studies for the Conduct of Human Clinical Trials and Marketing Authorization for Pharmaceuticals (ICH M3 (R2)), EMA/CPMP/ICH/286/95
- Preclinical Safety Evaluation of Biotechnology-derived Pharmaceuticals (ICH S6 (R1)), EMA/CHMP/ICH/731268/1998
- Nonclinical Evaluation for Anticancer Pharmaceuticals (ICH S9), EMA/CHMP/ICH/646107/08
- Guidance on Genotoxicity Testing and Data Interpretation for Pharmaceuticals Intended for Human Use (ICH S2 (R1)), EMEA/CHMP/ICH/126642/2008
- General Considerations for Clinical Trials (ICH E8), CPMP/ICH/291/95
- Clinical Investigation of Medicinal Products in the Paediatric Population (ICH E11 (R1)), CPMP/ICH/2711/99
- Guideline on Risk Assessment of Medicinal Products on Human Reproduction and Lactation: from Data to Labelling, EMEA/CHMP/203927/2005
- Guideline on the Summary of Product Characteristics – SmPC (September 2009). In EUDRALEX – Volume 2C - Regulatory Guidelines in Notice to applicants and regulatory guidelines for medicinal products for human use
- Guideline for Good Clinical Practice (ICH E6 (R1)), CPMP/ICH/135/95
- Note for Guidance on Development Safety Update Reports (ICH E2F), EMA/CHMP/ICH/309348/2008
- U.S. Medical Eligibility Criteria for Contraceptive Use, 2015; Adapted from the World Health Organization (WHO) August 2015 – with special regard to table 2.1. (Part II Using the recommendations)
- Guideline on the Exposure to Medicinal Products during Pregnancy: Need for Post- Authorisation Data, EMEA/CHMP/313666/2005
- Guideline on the Investigation of Drug Interactions, CPMP/EWP/560/95/Rev.1 Corr.
- U.S. Selected Practice Recommendations for Contraceptive Use, 2016

Clinical Trials Facilitation and Coordination Group

CTFG

- Guideline on Strategies to Identify and Mitigate Risks for First-in-Human and Early Clinical Trials with Investigational Medicinal Products, EMEA/CHMP/SWP/28367/07 Rev.1
- Assessment and Control of DNA Reactive (Mutagenic) Impurities in Pharmaceuticals to Limit Potential Carcinogenic Risk (ICH M7 (R1)) CHMP/ICH/83812/2013
- Guideline on reproductive toxicology: Detection of Toxicity to Reproduction for Human Pharmaceuticals (ICH S5 (R3)) EMA/CHMP/ICH/544278/1998

Clinical Trials Facilitation and Coordination Group

CTFG

1 Definitions

1.1 Definition of women of childbearing potential and of fertile men

For the purpose of this document, a woman is considered of childbearing potential (WOCBP), i.e. fertile, following menarche and until becoming post-menopausal unless permanently sterile. Permanent sterilisation methods include hysterectomy, bilateral salpingectomy and bilateral oophorectomy.

A postmenopausal state is defined as no menses for 12 months without an alternative medical cause. A high follicle stimulating hormone (FSH) level in the postmenopausal range may be used to confirm a post-menopausal state in women not using hormonal contraception or hormonal replacement therapy. However in the absence of 12 months of amenorrhea, a single FSH measurement is insufficient.

For the purpose of this document, a man is considered fertile after puberty unless permanently sterile by bilateral orchidectomy.

1.2 Definition of end of relevant systemic exposure

For the purpose of this document the end of relevant systemic exposure is defined as the time point where the IMP, including any active or major metabolites, has decreased to a concentration that is no longer considered relevant for human teratogenicity/fetotoxicity. In case reproductive toxicity studies are available, this systemic exposure level should include a sufficient exposure margin to the no-observed adverse effect level (NOAEL) in the non- clinical reproductive toxicity studies. In the absence of reproductive toxicity studies, such considerations may be based on the principles of a minimal anticipated biological effect level (MABEL) or other accepted principles. In case of a genotoxic IMP the principle of threshold of toxicological concern (TTC) should be considered.

2 How to proceed from risk assessment to practical contraception recommendations

2.1 Risk Assessment

2.1.1 IMPs with Marketing Authorisation

In case of clinical trials with authorised IMPs, the appropriate labelling (the SmPC, for medicinal products approved in the EU) should be reviewed when assessing contraception recommendations. In case of existing contraception recommendations, these should form the basis for the contraception recommendation with the IMP, but their relevance for the specific clinical trial needs to be assessed and justified by the applicant. In case of no contraception recommendations, the principles for IMPs without marketing authorisation (MA) should be applied.

2.1.2 IMPs without Marketing Authorisation

In case of clinical trials with IMPs that have not yet received MA, there is usually limited or no information about the outcome of pregnancies in humans following in utero or gonadal exposure. Depending on the stage of clinical development there may also be limited or no information from non-clinical reproduction toxicity studies.

The general recommendation in the ICH M3(R2) guideline is that "all female reproduction toxicity studies and the standard battery of genotoxicity tests should be completed prior to the inclusion, in any clinical trial, of WOCBP not using highly effective birth control or whose pregnancy status is unknown".

The following non-clinical toxicological studies for risk assessment during preconception and early stages of pregnancy are considered necessary in order to allow a conclusion that non- clinical toxicological studies do not indicate a risk to the unborn that would necessitate the requirement for highly effective methods of contraception in clinical trials (the timings of these studies are included in the appropriate guidelines):

- A standard battery of genotoxicity testing (if applicable)

- Repeated dose toxicity of adequate duration
- Embryofetal development
- Fertility and early embryonic development

Given that it is assumed that treatment with the IMP will be interrupted in case of pregnancy, the pre- and postnatal development study is not considered necessary for assessment of risk to the unborn, except for IMPs with exceptionally long half-lives. Since the focus of this guidance is on the early stages of pregnancy, the main concern relates to evidence of teratogenicity.

Risk assessment should be based on all relevant available non-clinical and clinical data, including pharmacology and pharmacokinetic data, in accordance with the CHMP "Guideline on risk assessment of medicinal products on human reproduction and lactation: from data to labelling". In order to specify the duration of the risk mitigation measures after discontinuation of treatment with the IMP, the risk assessment should include an estimation of the end of relevant systemic exposure (see section 1.2).

In the present guidance document the following three main risk categories for the early stages of pregnancy have been adapted from the risk categories set in table 1 of the above CHMP guideline:

- Demonstrated or suspected human teratogenicity/fetotoxicity
- Possible human teratogenicity/fetotoxicity
- Unlikely human teratogenicity/fetotoxicity

In case of insufficient or unavailable non-clinical data, the impact on the risk categorization should be evaluated. Unavailable or insufficient non-clinical data should be considered as "effects detected", and the highest possible risk category assumed .

Genotoxicity / genetic damage at the level of the germ cells and/or conceptus may deserve particular attention due to its potential irreversible nature. If genotoxic effects take place in the germ cells that are undergoing or completing meiosis (spermatocytes, preovulatory oocytes), but not in the primordial spermatogonia or in the oocytes that are arrested in the first meiotic prophase, such effects may be considered reversible in the sense that new spermatocytes or arrested oocytes are unaffected. It is recommended that, for male subjects with pregnant or non-pregnant WOCBP partner, a minimum one sperm cycle (here defined as 90 days) should be awaited after the relevant systemic exposure to the medicinal product has ended. In case of women of childbearing potential, a minimum of one menstruation cycle (30 days/'1 month'), for aneugenic compounds, or one folliculogenesis cycle (here defined as 6 months), in case of other types of genotoxicants, should be awaited after the relevant systemic exposure to the medicinal product has ended (see section 1.2).

Concerning the embryo-fetal risk posed from treatment of male subjects with IMPs capable of provoking embryo-fetal harm, there is a theoretical risk of human teratogenicity/fetotoxicity in a pregnant WOCBP partner through exposure to the ejaculate. Exposure levels in the WOCBP partner are, however, much smaller from exposure to semen compared with direct intake of the IMP by the WOCBP. Estimated exposure levels in WOCBP are three or more orders of magnitude lower than the plasma concentrations in the male subject (Klemmt & Scialli, The Transport of Chemicals in Semen. Birth Defects Research 2005; 74: 119-31).

A concern may, therefore, only apply to IMPs with demonstrated or suspected human teratogenicity/fetotoxicity in the early pregnancy (see section 2.2.2) at sub-therapeutic systemic exposure levels.

2.2 Birth Control and Pregnancy Testing Recommendations for WOCBP

2.2.1 General considerations

Clinical Trials Facilitation and Coordination Group

CTFG

WOCBP should only be included after a confirmed menstrual period and a negative highly sensitive urine or serum pregnancy test, except for IMPs where an absence of risk of human teratogenicity/fetotoxicity can be justified by human pregnancy data.

The recommendations below, with respect to contraception and pregnancy testing, are provided in relation to the risk categories that have been adapted from the "Guideline on risk assessment of medicinal products on human reproduction and lactation: from data to labelling", and concern both authorized and unauthorized IMPs.

2.2.2 Contraception and pregnancy testing recommendations for IMPs with demonstrated or suspected human teratogenicity/fetotoxicity

This refers to IMPs where a malformative effect has been demonstrated in humans or is suspected on the basis of class effects, IMPs with genotoxic potential, or IMPs where there is a strong suspicion of human teratogenicity/fetotoxicity based on non- clinical data.

- The inclusion of WOCBP requires use of a highly effective contraceptive measure (see sections 4.1 and 4.3). Contraception methods with low user dependency (see section 4.1, footnote 2) should preferably be used, in particular when contraception is introduced as a result of participation in the clinical trial.

- Additional pregnancy testing should be performed at monthly intervals.

- The above mentioned risk mitigation measures (contraception and pregnancy testing) should be maintained during treatment and until the end of relevant systemic exposure (see section 1.2). This period should be extended by 1 month, in case of purely aneugenic compounds, or by 6 months, in case of other type of genotoxicity (see section 2.1.2).

2.2.3 Contraception and pregnancy testing recommendations for IMPs with possible human teratogenicity/fetotoxicity

This refers to IMPs, where human data on pregnancies is limited or not available, there is no suspicion of human teratogenicity based on class effects or genotoxic potential, and non- clinical reproductive toxicity studies of relevance for early human pregnancy show positive findings that do not generate a strong suspicion of human teratogenicity/fetotoxicity.

- The inclusion of WOCBP requires use of a highly effective contraceptive measure (see sections 4.1 and 4.3). Contraception should be maintained during treatment and until the end of relevant systemic exposure (see section 1.2).

- Additional pregnancy testing should be considered taking into account, amongst others, the duration of the trial. As a minimum, a pregnancy test should be performed at the end of relevant systemic exposure.

- In each case of delayed menstrual period (over one month between menstruations) confirmation of absence of pregnancy is strongly recommended. This recommendation also applies to WOCBP with infrequent or irregular menstrual cycles.

2.2.4 Contraception and pregnancy testing recommendations for IMPs with unlikely human teratogenicity/fetotoxicity

This refers to IMPs where assessment of the completed necessary non-clinical studies (see section 2.1.2) does not indicate teratogenicity/fetotoxicity and human data are not available or do not contradict these findings or there is already sufficient evidence for lack of risk based on human data.

- The inclusion of WOCBP is possible using at least an acceptable effective contraceptive measure unless an absence of risk of human teratogenicity/ fetotoxicity in early pregnancy can be justified by human pregnancy data (see sections 4.1, 4.2 and 4.3 for methods considered acceptable and section 4.4 for methods considered unacceptable). As a minimum contraception should be maintained until treatment

discontinuation.

- Unless a woman is suspected to have become pregnant, additional pregnancy testing during the clinical trial is not necessary.

2.2.5 Other factors to consider

The choice of contraceptive methods for WOCBP and the frequency of pregnancy testing may need to be adapted to special circumstances, which should be justified by the sponsor. Factors to consider when adapting the need for a specific clinical trial may include e.g. exposure to IMP, study duration, fertility of study population, and seriousness of the treated medical condition.

2.3 Recommendations for male subjects with pregnant or non-pregnant WOCBP partner

For IMPs with possible or unlikely risk of human teratogenicity/fetotoxicity (see sections 2.2.3 and 2.2.4), no contraception measures are needed for male subjects with pregnant or non-pregnant WOCBP partner. Also for non-genotoxic IMPs with demonstrated, or suspected human teratogenicity/fetotoxicity (see section 2.2.2), only at therapeutic or supratherapeutic systemic exposure levels, no contraception measures are needed. For non-genotoxic IMPs with demonstrated or suspected human teratogenicity/fetotoxicity (see section 2.2.2), at subtherapeutic systemic exposure levels, where it is theoretically possible that relevant systemic concentrations may be achieved in WOCBP from exposure to seminal fluid, male contraception (condom) is recommended in order to avoid exposure of an existing embryo/fetus. Contraception should be continued until the end of relevant systemic exposure in WOCBP (see section 1.2).
For genotoxic IMPs, the male subject should use condom during treatment and until the end of relevant systemic exposure in the male subject (see section 1.2), plus a further 90-day period (see section 2.1.2). For a non-pregnant WOCBP partner, contraception recommendations should also be considered.

3 Provision of information in the IB/appropriate label and trial protocol

3.1 Information to be provided in the IB/appropriate label

For clinical trials with IMPs that have not yet received MA the analysis of embryofetal risk should be provided in the Investigator's Brochure (IB). The "Summary of data and guidance for the investigator", or equivalent section as part of the reference safety information should contain the above mentioned risk assessment (see section 2.1) and the recommendations for the level of contraception and frequency of pregnancy testing (see sections 2.2 and 2.3). The information should be sufficiently detailed to indicate the duration of the need for contraceptive measures and pregnancy testing.

Regarding the content of this information, reference is made to the SmPC guideline. For clinical trials with authorised IMPs the SmPC is the basis for the analysis of embryofetal risk (see section 2.1.1).

Where hormonal contraception methods are recommended, birth control methods assessment should be made of the likelihood of possible interaction with IMP (see section 4.3).

3.2 Information to be provided in the trial protocol

The specific recommendations for contraception and pregnancy testing for a clinical trial in the study protocol should be adequate in relation to the information provided in the IB/appropriate label and any other factors to consider. They should encompass all IMPs as well as non-investigational medicinal products, e.g. background therapy and the measures to be followed should be based on the medicinal product with highest risk. The study protocol should contain detailed information on the level of contraception and the possibility for an interaction between the IMP or the non-investigational medicinal products and hormonal contraceptives, the frequency of pregnancy testing, and the duration of the need for contraceptive measures and pregnancy testing. The need for sexual counseling of study subjects, e.g. in adolescents, should be reflected in the protocol, as well as, information to seek advice about donation and cryopreservation of germ cells in line with this guidance prior treatment if applicable.

Clinical Trials Facilitation and Coordination Group

CTFG

4 Birth control methods

4.1 Birth control methods which may be considered as highly effective

For the purpose of this guidance, methods that can achieve a failure rate of less than 1% per year when used consistently and correctly are considered as highly effective birth control methods. Such methods include:

- combined (estrogen and progestogen containing) hormonal contraception associated with inhibition of ovulation 1:
  - oral
  - intravaginal
  - transdermal
- progestogen-only hormonal contraception associated with inhibition of ovulation 1:
  - oral
  - injectable
  - implantable 2
- intrauterine device (IUD) 2
- intrauterine hormone-releasing system (IUS) 2
- bilateral tubal occlusion 2
- vasectomised partner 2,3
- sexual abstinence 4

1 Hormonal contraception may be susceptible to interaction with the IMP, which may reduce the efficacy of the contraception method (see section 4.3)

2 Contraception methods that in the context of this guidance are considered to have low user dependency.

3 Vasectomised partner is a highly effective birth control method provided that partner is the sole sexual partner of the WOCBP trial participant and that the vasectomised partner has received medical assessment of the surgical success.

4 In the context of this guidance sexual abstinence is considered a highly effective method only if defined as refraining from heterosexual intercourse during the entire period of risk associated with the study treatments. The reliability of sexual abstinence needs to be evaluated in relation to the duration of the clinical trial and the preferred and usual lifestyle of the subject.

Clinical Trials Facilitation and Coordination Group

CTFG

4.2 Acceptable birth control methods which may not be considered as highly effective

Acceptable birth control methods that result in a failure rate of more than 1% per year include:

- progestogen-only oral hormonal contraception, where inhibition of ovulation is not the primary mode of action
- male or female condom with or without spermicide 5
- cap, diaphragm or sponge with spermicide 5

3 A combination of male condom with either cap, diaphragm or sponge with spermicide (double barrier methods) are also considered acceptable, but not highly effective, birth control methods

4.3 Assessment of pharmacokinetic interaction between the IMP and hormonal contraceptives and recommendations on the use of hormonal contraceptives

For hormonal contraception methods, caution should be taken to possible interaction with a (non- biologic) IMP. Interaction with the IMP leading to reduced efficacy of the hormonal contraception method can occur due to e.g. increased metabolism (enzyme induction).

A potential human teratogen needs to be studied in vivo for effects on contraceptive steroids if the drug is intended for use in fertile women, regardless on the in vitro induction study results (see Guideline on the Investigation of Drug Interactions). For the purpose of this guidance, an IMP with demonstrated or suspected human teratogenicity/fetotoxicity in early pregnancy (see section 2.2.2) is a potential human teratogen. For these IMPs, data from a clinical pharmacokinetic interaction study between the IMP and contraceptive steroids, if available, allow to conclude whether the efficacy of hormonal contraception is reduced. In the absence of such a clinical pharmacokinetic interaction study, any recommendation for use of hormonal contraceptives should be thoroughly justified by the sponsor.

For all other IMPs, recommendations should take into account both the evidence of the non- clinical reproductive toxicity data and available information related to the potential risk for interaction, e.g. in vitro enzyme induction results, signs of autoinduction and results from clinical interaction studies.

As a general rule, use of hormonal contraception is not recommended if a clinically relevant interaction with contraceptive steroids has been observed or is suspected. If an interaction with contraceptive steroids has been observed or is suspected, but the effect is considered to be of limited clinical significance, the hormonal contraception method must be supplemented with a barrier method (preferably male condom).

An assessment of the potential for interaction between the IMP and hormonal contraceptives should be provided in the IB, including a scientific rationale for the use of hormonal contraception methods with or without a supplementary barrier method (preferably male condom).

4.4 Birth control methods which are considered unacceptable in clinical trials

Periodic abstinence (calendar, symptothermal, post-ovulation methods), withdrawal (coitus interruptus), spermicides only, and lactational amenorrhoea method (LAM) are not acceptable methods of contraception. Female condom and male condom should not be used together.

Clinical Trials Facilitation and Coordination Group

CTFG

Decision Trees - Recommendations Related to Contraception and Pregnancy Testing in Clinical Trials

Women of Childbearing Potential (WOCBP)

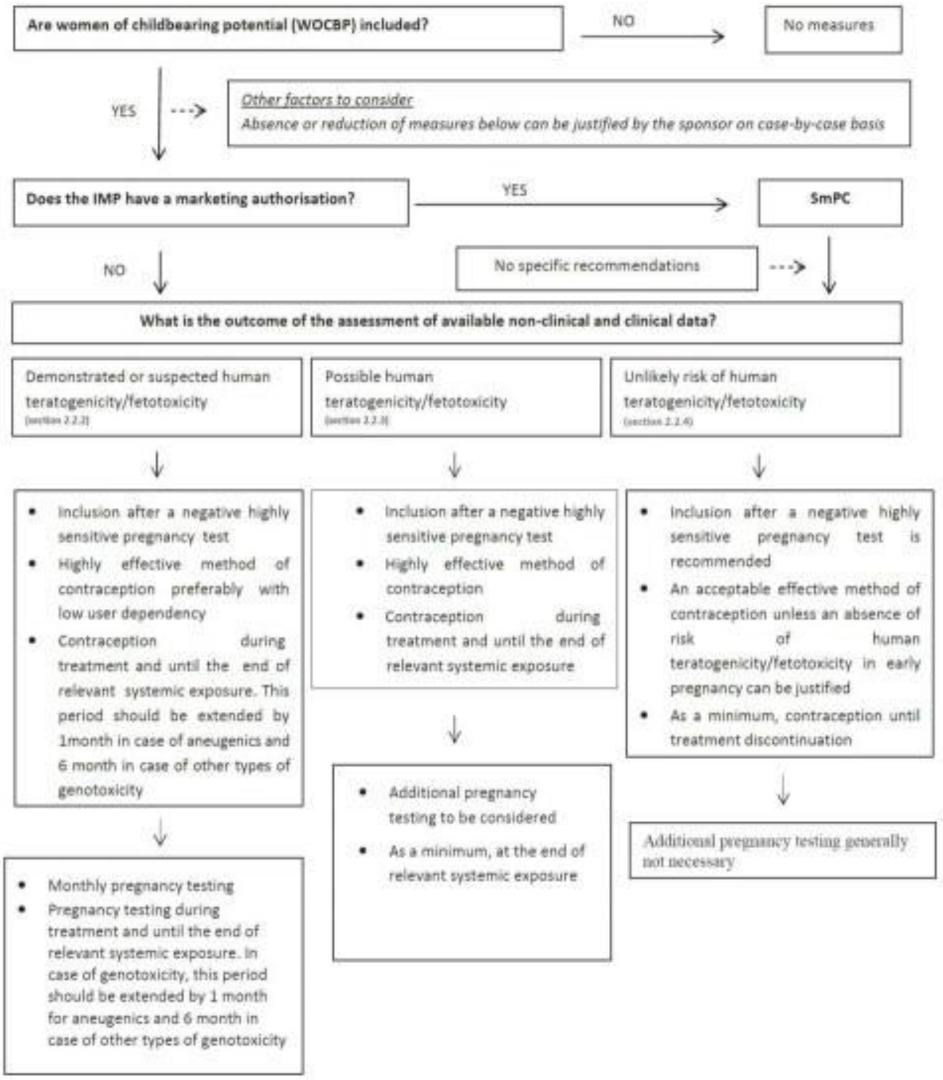

Clinical Trials Facilitation and Coordination Group

CTFG

Males with WOCBP Partners

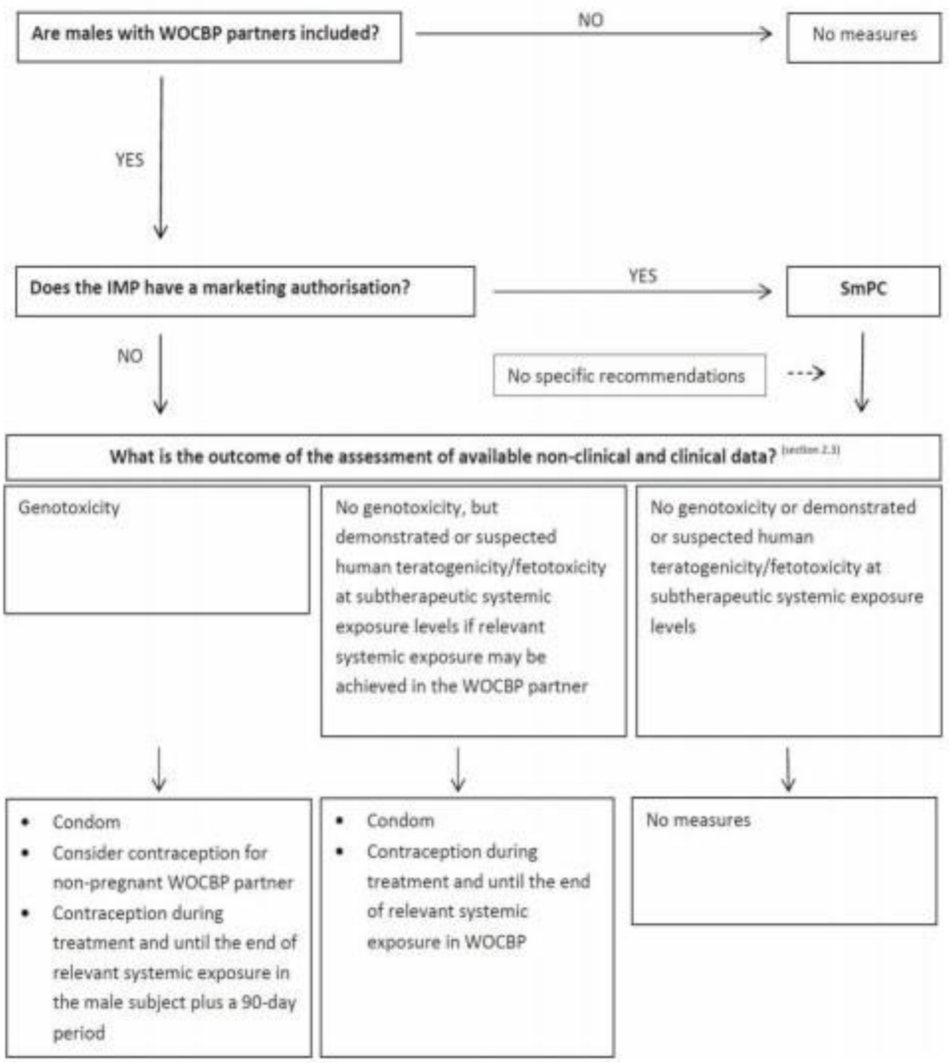

CTFG 21/09/2020 Version 1.1 10

APPENDIX 6

New York Heart Association criteria

Class	Patient Symptoms	Objective Assessment
I	No limitation of physical activity. Ordinary physical activity does not cause undue fatigue, palpitation, dyspnea (shortness of breath).	No objective evidence of cardiovascular disease. No symptoms and no limitation in ordinary physical activity.
II	Slight limitation of physical activity. Comfortable at rest. Ordinary physical activity results in fatigue, palpitation, dyspnea (shortness of breath).	Objective evidence of minimal cardiovascular disease. Mild symptoms and slight limitation during ordinary activity. Comfortable at rest.
III	Marked limitation of physical activity. Comfortable at rest. Less than ordinary activity causes fatigue, palpitation, or dyspnea.	Objective evidence of moderately severe cardiovascular disease. Marked limitation in activity due to symptoms, even during less- than-ordinary activity. Comfortable only at rest.
IV	Unable to carry on any physical activity without discomfort. Symptoms of heart failure at rest. If any physical activity is undertaken, discomfort increases.	Objective evidence of severe cardiovascular disease. Severe limitations. Experiences symptoms even while at rest.

APPENDIX 7

Drug Induced Liver Injury (DILI)

Alterations of liver laboratory parameters, as described in Section 11.1.4 Adverse event of special interest (AESI), are to be further evaluated using the following procedures:

Procedures

Repeat the following laboratory tests:

ALT, AST, and bilirubin (total and direct) - within 48 to 72 hours. If ALT and/or AST >3 fold ULN combined with an elevation of total bilirubin >2 fold ULN are confirmed (if normal values at baseline/screening), or ALT and/or AST >5 fold ULN combined with an elevation of total bilirubin >2 fold ULN are confirmed (if elevated values at baseline/screening) results of the laboratory parameters described below must be made available to the investigator and to the Sponsor as soon as possible.

In addition, obtain and report these via the eCRF

- a detailed history of current symptoms and concurrent diagnoses and medical history
- a history of concomitant drug use (including non-prescription medications, herbal and dietary supplement preparations), alcohol use, recreational drug use, and special diets
- a history of exposure to environmental chemical agents (consider home and workplace exposure)

Provide abdominal ultrasound or other appropriate imaging to rule out biliary tract, pancreatic or intrahepatic pathology, e.g., bile duct stones or neoplasm.

Clinical chemistry

alkaline phosphatase, albumin, PT or INR, CK, CK-MB, ceruloplasmin, α-1 antitrypsin, transferrin, amylase, lipase, fasting glucose, cholesterol, triglycerides, glutamate-dehydrogenase, D-dimers, C-reactive protein, gamma-glutamyl-transpeptidase

Serology

Hepatitis A (RNA), Hepatitis B (HbsAg, Anti-HBs, DNA), Hepatitis C (Anti-HCV, RNA), Hepatitis D (Anti- IgM, Anti-IgG), Hepatitis E (Anti-HEV, Anti-HEV IgM, RNA if Anti-HEV IgM positive), Cytomegalovirus (repeat CMV DNA), Anti-Smooth Muscle antibody (titer), Anti-nuclear antibody (titer), Anti-LKM (liver-kidney microsomes) antibody, Anti-mitochondrial antibody

Hormone

Thyroid stimulating hormone

Hematology

White blood count + differential, hemoglobin, thrombocytes

In case AST/ALT remain elevated and the previous testing does not provide a likely cause for the elevation, the following tests should be performed: Epstein Barr Virus (VCA IgG, VCA IgM), herpes simplex virus (IgG, IgM), varicella (IgG, IgM), parvovirus (IgG, IgM), toxoplasmosis (IgG, IgM).

Initiate close observation of patients by repeat testing of ALT, AST, and total bilirubin (with fractionation by total and direct) at least weekly until the laboratory ALT and/or AST abnormalities stabilize or return to normal, then according to the protocol. Depending on further laboratory changes or additional parameters identified, follow-up should be based on medical judgement and Good Clinical Practice (GCP).

APPENDIX 8

Guidance on causal relationship

The following factors should be considered when deciding if there is a "reasonable possibility" that an AE may have been caused by the study treatment.

1. Time course/exposure to suspect IMP: has the patient actually received the suspect Investigational Medicinal Product (IMP)? Did the AE occur in a reasonable temporal relationship to the administration of the suspect IMP?
2. Consistency with known drug profile: was the AE consistent with the previous knowledge of the suspect IMP (pharmacology and toxicology) or drugs of the same pharmacological class? Or, could the AE be anticipated from its pharmacological properties?
3. Dechallenge experience: did the AE resolve or improve on stopping or reducing the dose of the suspect IMP?
4. No alternative cause: the AE cannot be reasonably explained by another etiology such as the underlying disease, other drugs, other host or environmental factors.
5. Rechallenge experience: if the suspect IMP was reintroduced after having been stopped, did the AE reoccur? Rechallenge is not normally recommended or supported.
6. Laboratory tests: has a specific laboratory investigation confirmed the relationship?

When one or more of the above factors apply, the question, "is there a reasonable possibility that a causal relationship to the suspect IMP exists?" should usually be answered "Yes". If none of the above factors apply or where there is evidence of exposure and a reasonable time course but any dechallenge (if performed) is negative or ambiguous or there is another more likely cause of the AE, the answer to the question should be "No".

In difficult cases, other factors could be considered such as:

- Is this a recognized feature of overdose of the IMP?
- Is there a known mechanism?

Ambiguous cases should be considered as a "reasonable possibility" of a causal relationship to the suspect IMP until/if evidence becomes available to refute this. Causal relationship in cases where the disease under study has deteriorated due to lack of effect should be classified as no reasonable possibility.

DocuSign

Certificate Of Completion

Envelope Id: 9D33A835AA3642358B37B8C1C0FD5A6E Status: Completed Subject: Complete with DocuSign: OPTIMIZE-1_CSP_v.6.0_ final_05OCT 2022 acrobat DC.pdf
Source Envelope:
Document Pages: 154 Signatures: 3 Envelope Originator:
Certificate Pages: 5 Initials: 0 Annette Viegas Lopes Gustavsson
AutoNav: Enabled aeg@alligatorbioscience.com IP Address: 193.42.21.182
EnvelopeId Stamping: Disabled
Time Zone: (UTC-08:00) Pacific Time (US & Canada)

Record Tracking

Status: Original<br>10/6/2022 5:26:27 AM	Holder: Annette Viegas Lopes Gustavsson<br>aeg@alligatorbioscience.com	Location: DocuSign

Signer Events	Signature	Timestamp
Karin Nordbladh knh@alligatorbioscience.com Alligator Bioscience AB<br>Security Level: Email, Account Authentication (Required)<br>Signature ID: C25714D6-81C1-4564-B2C5-1699D25B4FFC<br>Using IP Address: 155.4.242.104	Karin Nordbladh<br>Signature Adoption: Pre-selected Style<br><br>With Signing Authentication via DocuSign password With Signing Reasons (on each tab):<br>I approve this document	Sent: 10/6/2022 5:58:53 AM Viewed: 10/6/2022 6:01:07 AM Signed: 10/7/2022 5:53:42 AM

Electronic Record and Signature Disclosure:
Not Offered via DocuSign

Sumeet Ambarkhane<br>sua@alligatorbioscience.com Chief Medical Officer Alligator Bioscience AB<br>Security Level: Email, Account Authentication (Required)	Tommy Schyman<br>Tommy.schyman@4pharma.com<br>Security Level: Email, Account Authentication (Required)	SAmbarkhane<br>Signature Adoption: Drawn on Device<br>Signature ID: 9F668ED3-6F39-4C19-A457-C05E7D71E9F8<br>Using IP Address: 95.90.195.180<br><br>With Signing Authentication via DocuSign password With Signing Reasons (on each tab):<br>I approve this document

Electronic Record and Signature Disclosure:
Not Offered via DocuSign

Electronic Record and Signature Disclosure:

Signature Adoption: Pre-selected Style Signature ID:
64F03265-48B3-4DFD-BA12-6CF6AE89812C
Using IP Address: 31.208.27.197

With Signing Authentication via DocuSign password With Signing Reasons (on each tab):
I approve this document

Sent: 10/6/2022 5:58:52 AM Viewed:

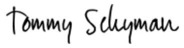

10/6/2022 6:00:21 AM Signed:
10/6/2022 6:01:19 AM

Sent: 10/6/2022 5:58:53 AM Viewed:
10/6/2022 11:25:44 PM Signed:
10/6/2022 11:26:37 PM

Signer Events	Signature	Timestamp
Accepted: 10/2/2022 11:51:33 PM<br>ID: c2d596d6-b9e6-4aee-9c47-932c4274ddb9		

In Person Signer Events	Signature	Timestamp

Editor Delivery Events	Status	Timestamp

Agent Delivery Events	Status	Timestamp

Intermediary Delivery Events	Status	Timestamp

Certified Delivery Events	Status	Timestamp

Carbon Copy Events	Status	Timestamp
Tina Hjort<br>tih@alligatorbioscience.com<br>CTM Consultant<br>Security Level: Email, Account Authentication (Required)<br>**Electronic Record and Signature Disclosure:**<br>Not Offered via DocuSign	COPIED	Sent: 10/6/2022 5:58:54 AM<br>Viewed: 10/7/2022 5:54:55 AM

Witness Events	Signature	Timestamp

Notary Events	Signature	Timestamp

Envelope Summary Events	Status	Timestamps
Envelope Sent	Hashed/Encrypted	10/6/2022 5:58:54 AM
Certified Delivered	Security Checked	10/6/2022 11:25:44 PM
Signing Complete	Security Checked	10/6/2022 11:26:37 PM
Completed	Security Checked	10/7/2022 5:53:42 AM

Payment Events	Status	Timestamps

Electronic Record and Signature Disclosure

Electronic Record and Signature Disclosure created on: 2/27/2022 11:03:25 PM Parties agreed to: Tommy Schyman

ELECTRONIC RECORD AND SIGNATURE DISCLOSURE

From time to time, Alligator Bioscience (we, us or Company) may be required by law to provide to you certain written notices or disclosures. Described below are the terms and conditions for providing to you such notices and disclosures electronically through the DocuSign system.
Please read the information below carefully and thoroughly, and if you can access this information electronically to your satisfaction and agree to this Electronic Record and Signature Disclosure (ERSD), please confirm your agreement by selecting the check-box next to 'I agree to use electronic records and signatures' before clicking 'CONTINUE' within the DocuSign system.

Getting paper copies

At any time, you may request from us a paper copy of any record provided or made available electronically to you by us. You will have the ability to download and print documents we send to you through the DocuSign system during and immediately after the signing session and, if you elect to create a DocuSign account, you may access the documents for a limited period of time (usually 30 days) after such documents are first sent to you. After such time, if you wish for us to send you paper copies of any such documents from our office to you, you will be charged a $0.00 per-page fee. You may request delivery of such paper copies from us by following the procedure described below.

Withdrawing your consent

If you decide to receive notices and disclosures from us electronically, you may at any time change your mind and tell us that thereafter you want to receive required notices and disclosures only in paper format. How you must inform us of your decision to receive future notices and disclosure in paper format and withdraw your consent to receive notices and disclosures electronically is described below.

Consequences of changing your mind

If you elect to receive required notices and disclosures only in paper format, it will slow the speed at which we can complete certain steps in transactions with you and delivering services to you because we will need first to send the required notices or disclosures to you in paper format, and then wait until we receive back from you your acknowledgment of your receipt of such paper notices or disclosures. Further, you will no longer be able to use the DocuSign system to receive required notices and consents electronically from us or to sign electronically documents from us.

All notices and disclosures will be sent to you electronically

Unless you tell us otherwise in accordance with the procedures described herein, we will provide electronically to you through the DocuSign system all required notices, disclosures, authorizations, acknowledgements, and other documents that are required to be provided or made available to you during the course of our relationship with you. To reduce the chance of you inadvertently not receiving any notice or disclosure, we prefer to provide all of the required notices and disclosures to you by the same method and to the same address that you have given us. Thus, you can receive all the disclosures and notices electronically or in paper format through the paper mail delivery system. If you do not agree with this process, please let us know as described below. Please also see the paragraph immediately above that describes the consequences of your electing not to receive delivery of the notices and disclosures electronically from us.

How to contact Alligator Bioscience:

You may contact us to let us know of your changes as to how we may contact you electronically, to request paper copies of certain information from us, and to withdraw your prior consent to receive notices and disclosures electronically as follows:
To contact us by email send messages to: grd@alligatorbioscience.com

To advise Alligator Bioscience of your new email address

To let us know of a change in your email address where we should send notices and disclosures electronically to you, you must send an email message to us at grd@alligatorbioscience.com and in the body of such request you must state: your previous email address, your new email address. We do not require any other information from you to change your email address.

If you created a DocuSign account, you may update it with your new email address through your account preferences.

To request paper copies from Alligator Bioscience

To request delivery from us of paper copies of the notices and disclosures previously provided by us to you electronically, you must send us an email to grd@alligatorbioscience.com and in the body of such request you must state your email address, full name, mailing address, and telephone number. We will bill you for any fees at that time, if any.

To withdraw your consent with Alligator Bioscience

To inform us that you no longer wish to receive future notices and disclosures in electronic format you may:

i. decline to sign a document from within your signing session, and on the subsequent page, select the check-box indicating you wish to withdraw your consent, or you may;

ii. send us an email to grd@alligatorbioscience.com and in the body of such request you must state your email, full name, mailing address, and telephone number. We do not need any other information from you to withdraw consent.. The consequences of your withdrawing consent for online documents will be that transactions may take a longer time to process..

Required hardware and software

The minimum system requirements for using the DocuSign system may change over time. The current system requirements are found here: https://support.docusign.com/guides/signer-guide- signing-system-requirements.

Acknowledging your access and consent to receive and sign documents electronically

To confirm to us that you can access this information electronically, which will be similar to other electronic notices and disclosures that we will provide to you, please confirm that you have read this ERSD, and (i) that you are able to print on paper or electronically save this ERSD for your future reference and access; or (ii) that you are able to email this ERSD to an email address where you will be able to print on paper or save it for your future reference and access. Further, if you consent to receiving notices and disclosures exclusively in electronic format as described herein, then select the check-box next to 'I agree to use electronic records and signatures' before clicking 'CONTINUE' within the DocuSign system.
z
By selecting the check-box next to 'I agree to use electronic records and signatures', you confirm that:

- You can access and read this Electronic Record and Signature Disclosure; and
- You can print on paper this Electronic Record and Signature Disclosure, or save or send this Electronic Record and Disclosure to a location where you can print it, for future reference and access; and
- Until or unless you notify Alligator Bioscience as described above, you consent to receive exclusively through electronic means all notices, disclosures, authorizations, acknowledgements, and other documents that are required to be provided or made available to you by Alligator Bioscience during the course of your relationship with Alligator Bioscience.
